# Chronic alcohol-induced dysbiosis of the gut microbiota and gut metabolites impairs sperm quality in mice

**DOI:** 10.3389/fmicb.2022.1042923

**Published:** 2022-12-01

**Authors:** Hui Li, Ningshan Li, Qudong Lu, Jun Yang, Jiang Zhao, Qiong Zhu, Shanhong Yi, Weihua Fu, Tingting Luo, Jiawei Tang, Yi Zhang, Guoliang Yang, Zheng Liu, Jie Xu, Wei Chen, Jingzhen Zhu

**Affiliations:** ^1^Department of Ultrasound, Second Affiliated Hospital, Army Medical University, Chongqing, China; ^2^Department of Urology, Second Affiliated Hospital, Army Medical University, Chongqing, China

**Keywords:** chronic alcohol, gut microbiota, fecal microbiota transplantation, sperm quality, testicular inflammation

## Abstract

Studies have indicated that the ethanol exposure impairs the gut microbiota, At the same time, high levels of alcohol exposure damage sperm in mice. However, whether the gut microbiota is involved in mediating the effects of alcohol on sperm quality remains unclear. This study aimed to assess the effect of chronic alcohol consumption on intestinal microbiota in mice and analyze the potential pathophysiological effect of altered intestinal microbiota on sperm quality. We established a mouse model of chronic alcohol consumption by allowing male C57 mice to freely ingest 10% ethanol for 10 weeks, and collected the fecal microbiota of the male mice in the chronic drinking group (alcohol) and the control group (control) and transplanted the specimens into the transplant groups (the alcohol-fecal microbiota transplantation [FMT] group and the control-FMT group). Sperm quality was significantly decreased in the alcohol-FMT group compared with the control-FMT group. Gut microbiota analysis revealed that the abundance of 11 operational taxonomic units (OTUs) was altered in the alcohol-FMT group. Nontargeted metabolomics identified 105 differentially altered metabolites, which were mainly annotated to amino acids, lipids, glycerophosphoethanolamine, organic oxygenic compounds, organic acids and their derivatives, steroids, and flavonoids. In particular, the oxidative phosphorylation pathway, which is the key to spermatogenesis, was significantly enriched in the alcohol-FMT group. Moreover, compared with the control-FMT group, the alcohol-FMT group presented significantly higher serum endotoxin and inflammatory cytokine levels, with more pronounced T cell and macrophage infiltration in the intestinal lamina propria and elevated levels of testicular inflammatory cytokines. In addition, RNA sequencing showed significant differences in the expression of testis-related genes between the alcohol-FMT group and the control-FMT group. In particular, the expression of genes involved in gamete meiosis, testicular mitochondrial function, and the cell division cycle was significantly reduced in alcohol-FMT mice. In conclusion, these findings indicated that intestinal dysbiosis induced by chronic alcohol consumption may be an important factor contributing to impaired sperm quality. Chronic alcohol consumption induces intestinal dysbiosis, which then leads to metabolic disorders, elevated serum endotoxin and inflammatory cytokine levels, testicular inflammation, abnormal expression of related genes, and ultimately, impaired sperm quality. These findings are potentially useful for the treatment of male infertility.

## Introduction

Infertility has become a global problem, with an incidence of approximately 15% worldwide, as the third most common condition after cancer and cardiovascular and cerebrovascular diseases (Sharlip et al., 2002). Approximately 50% of infertility cases are male infertility (WHO, 2001; Agarwal and Prabakaran, 2005; Gaur et al., 2007). Over the past few decades, sperm concentrations have decreased by more than 50%, with no effective method to slow this trend (Safe, 2013; Sermondade et al., 2013; Levine et al., 2017). The decrease in sperm quality is caused by both environmental and genetic factors, and the sharp decrease in sperm quality over such a short period is most likely due to environmental factors, such as environmental pollution, stress, diet changes, smoking, and drinking (Ramlau-Hansen et al., 2010; Nordkap et al., 2016; Skakkebaek et al., 2016; Virtanen et al., 2017). With the rapid social and economic development in China, the fast pace of modern life, serious environmental pollution, dietary changes due to the Westernization of diets and lifestyles, unfavorable sexual behaviors, and increased abortion rates have contributed to the growing issue of infertility in China, and the incidence in China is as high as or even exceeds the global average (Zhang, 2020).

Studies have demonstrated that chronic alcohol consumption is an important factor modulating male spermatogenesis and motility (Srikanth et al., 1998; Oliva et al., 2006; Talebi et al., 2011; Rahimipour et al., 2013; Condorelli et al., 2015). In China, alcohol consumption is significant. The overall drinking rate is 30.5% among Chinese adults, with rates of 53.8% for men and 12.2% for women (Li et al., 2018). Chronic alcohol consumption causes testicular abnormalities and sexual dysfunction in humans and animals (El-Sokkary, 2001; Muthusami and Chinnaswamy, 2005). It causes local testicular and epididymal inflammation that leads to spermatocyte injury or death and subsequently affects sperm count and motility (Carlsen et al., 2003). Inflammation induces the production of toxic reactive oxygen species (ROS) and other toxins, as well as a variety of inflammatory cytokines, biologically active lipids, and some enzymes, which damage cells and blood vessels and stimulate immune cells, thereby resulting in physical damage and even male infertility (Yan and Han, 2012).

In recent years, the gut microbiota has become a topic of major interest in the field of biomedicine. Numerous studies have found that the gut microbiota is closely related to the status of all major organs and systems in the body (Jandhyala et al., 2015; Kataoka, 2016; Quigley, 2017; Fan and Pedersen, 2021). The gut microbiota is related to many factors, including the living environment, dietary structure, antibiotics, birthing method, breastfeeding, circadian rhythm and host genetics (Kumbhare et al., 2019; Vandenplas et al., 2020; Venneri et al., 2022). Alcohol consumption is a very important factor modulating the gut microbiota, and chronic drinking reduces the amount of bacteria with anti-inflammatory activity, leading to intestinal damage and leaky gut (Capurso and Lahner, 2017; Vujkovic-Cvijin et al., 2020). These changes in the gut microbiota play an important role in alcohol-related diseases (Bajaj, 2019). Studies have demonstrated that alcohol-induced changes in the composition and metabolic function of the gut microbiota mediate alcohol-induced oxidative stress, promote intestinal inflammation, increase endotoxin levels, and lead to intestinal hyperpermeability, systemic inflammation, and tissue damage/organ disease (Engen et al., 2015).

The factors affecting sperm quality are complex and varied, Researchers are discovering the role of the microbiota in male reproduction, including the gut microbiota and the reproductive tract microbiota (Venneri et al., 2022). This study mainly examines the effect of gut microbiota on sperm quality. Studies have described an important role for the gut microbiota in male spermatogenesis. For example, intestinal dysbiosis due to a high-fat diet (HFD) causes a significant decrease in sperm count and motility (Ding et al., 2020). In animal studies, busulfan affects sperm quality and causes intestinal dysbiosis, while alginate oligosaccharide (AOS) negates the adverse effect of busulfan on sperm quality by improving the gut microbiota. Moreover, improvements in the gut microbiota induced by AOS transplantation improve the quality of damaged sperm (Zhao et al., 2020; Zhang et al., 2021). In a sheep model of diet-induced metabolic disorders, intestinal dysbiosis leads to a decrease in bile acid levels, which affects the transport of fat-soluble vitamin A in the intestine-testis axis, and the low level of vitamin A in the testis affects spermatogenesis (Zhang et al., 2021). In summary, methods designed to regulate the gut microbiota and subsequently improve fertility may become a new hot topic (Yurtdaş and Akdevelioğlu, 2020).

Alcohol affects spermatogenesis and sperm quality. However, researchers have not clearly determined whether alcohol affects sperm quality through the gut microbiota. We hypothesized that chronic alcohol consumption causes intestinal dysbiosis, leading to the production of a large amount of toxic and harmful substances that damage the intestinal mucosa, increase intestinal permeability, and then enter the blood circulation and reach different sites, resulting in elevated levels inflammatory cytokines, an imbalance of inflammatory cytokines, the inflammatory response, and testicular damage. Varying degrees of pathological changes in testicular tissue ultimately lead to spermatocyte apoptosis, impaired sperm quality, and a low sperm count. In this study, we established a mouse model of chronic alcohol consumption and tested our hypothesis with fecal microbiota transplantation to investigate the effect of chronic drinking-induced intestinal dysbiosis on sperm quality.

## Materials and methods

### Animals

Three-week-old male C57 mice from the Experimental Animal Centre of Army Medical University were housed in a specific pathogen-free (SPF) environment at 18°C–26°C and 50%–60% humidity, with a 12-h-on/12-h-off light cycle. The mice had free access to feed and distilled water.

After 2 weeks of co-feeding (keep in the same cage), the mice were randomly divided into two groups with12 mice each for the alcohol treatment experiment: the control group (control; free access to feed and distilled water for 10 weeks), and the chronic drinking group (alcohol; free access to feed and 10% ethanol [99%, vol/vol] for 10 weeks). The average weekly alcohol intake of per mouse in the alcohol group is shown in Supplementary Table 1. All mice were sacrificed after anesthesia induction with 1% pentobarbital sodium solution by intraperitoneal injection.

Fecal microbiota transplantation (FMT) experiment: Three-week-old male C57 mice were co-fed (in the same cage) and then randomly divided into two groups with five mice each 2 weeks later: the alcohol-fecal microbiota transplantation (FMT) group (alcohol-FMT; fecal microbiota from the alcohol group was transplanted into normal mice; free access to feed and distilled water for 10 weeks), and the control-FMT group (fecal microbiota from the control group was transplanted into normal mice; free access to feed and distilled water for 10 weeks). All mice were sacrificed after anesthesia induction with 1% pentobarbital sodium solution by intraperitoneal injection.

### Fecal microbiota transplantation

**Figure 1:**
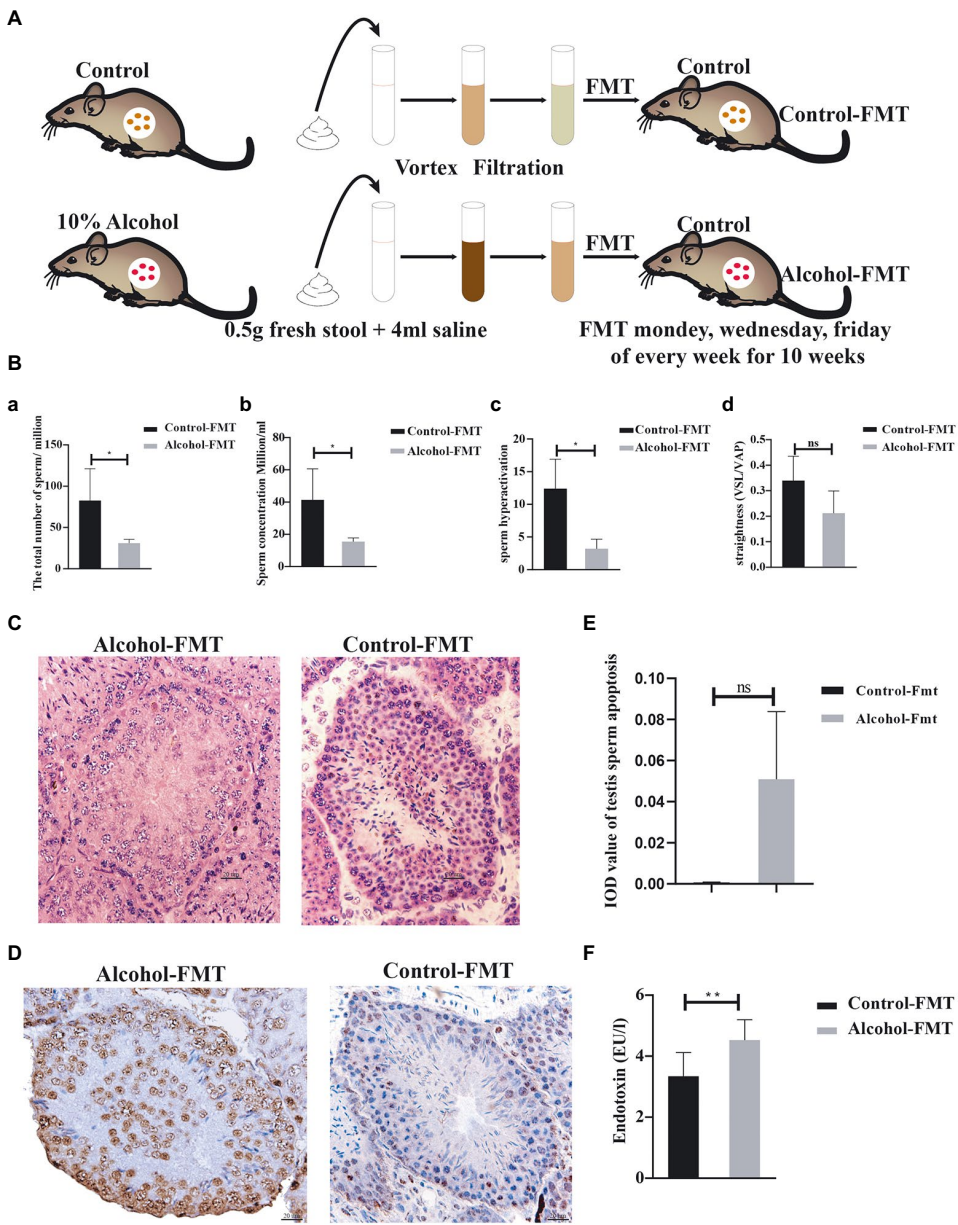
Fecal microbiota transplanted from alcohol-treated mice reduced sperm quality in recipient mice. **(A)** Schematic diagram of the FMT experiment. **(B)**—**(a)** The total number of sperm, **(b)** the concentration of sperm, **(c)** hyperactivation of sperm, and **(d)** the straightness of sperm. **(C)** Representative images of H&E staining showing impairment of the seminiferous tubules in the testis. Scale bar = 50 μm. **(D)** TUNEL assay of seminiferous tubules in the control-FMT group and alcohol-FMT group. Cells stained dark brown are apoptotic cells, while normal cells are stained blue (200X). **(E)** The mean integrated optical density (IOD) of TUNEL staining in the two groups. **(F)** Analysis of endotoxin levels in blood from the control-FMT group and alcohol-FMT group. ^*^p < 0.05 and ^**^p < 0.01.

Five hundred milligrams of fresh donor stool samples were continuously collected from the alcohol (five mice) and control (five mice) groups immediately upon defecation, resuspended in 4 ml of saline, vortexed for 5 min, and filtered with a 360-mesh sterile strainer. Transplantation into the recipient mice was achieved by gavage with 200 μl of the supernatant from the fecal sample every Monday, Wednesday, and Friday for 10 weeks (Figure 1A). Fecal samples from donors were collected during the whole fecal microbiota transplantation process.

### Sperm count and motility measurement

The cauda epididymis was minced in 100 ml of phosphate-buffered saline (PBS, pH 7.2) and incubated for 20 min at 37°C in a 5% CO_2_ incubator to release sperm. Sperm were observed under a Nikon N200 microscope and measured with a Suiplus semen analysis (SSA) system.

### Hematoxylin and eosin staining

Mouse testes and small intestines were harvested. The entire testis was fixed with a special testis solution (Servicebio Cat. No.: G1121) for 24 h. The small intestine was cut into small sections of approximately 1 cm, which were then cleaned and fixed with paraformaldehyde (Servicebio Cat. No.: G1101) for 24 h. Hematoxylin and eosin (H&E) staining was performed after dehydration in graded ethanol solutions, paraffin embedding, and sectioning (2.5 μm). Three random fields of sections on each slide were observed under an Olympus upright microscope (×200).

### Immunohistochemistry

The deparaffinized and rehydrated intestine and testis sections were blocked with 5% bovine serum albumin (BSA) containing 0.1% Triton X-100 for 1 h and incubated with a primary antibody against F4/80 (1:500, ab111101, Abcam, Cambridge, MA, United States) at 4°C overnight. Then, the sections were incubated with the secondary antibody (BA1039, Boster, Wuhan, Hubei, China) at room temperature for 1 h. After color development, the sections were observed under an Olympus microscope, and the proportions of positively stained cells were analyzed. Protein expression was quantified using ImageJ software (National Institutes of Health; http://www.imagej.softonic.de).

### Confocal microscopy and immunofluorescence staining

For immunofluorescence staining, paraffin sections of the intestines (2.5 μm thick) were assayed with 4′,6-diamidino-2-phenylindole (DAPI) as a counterstain. The primary antibody used in this experiment was rabbit anti-mouse CD3e (1:500; ab231775, Abcam, Cambridge, MA, United States). The secondary antibody (1:500; A11034, Invitrogen, United States) was an Alexa Fluor 488-conjugated donkey anti-rabbit antibody. Sections were examined under a ZEISS 880 confocal microscope. Protein expression was quantified using ImageJ software (National Institutes of Health; http://www.imagej.softonic.de).

### TUNEL staining

DNA fragmentation indicative of apoptosis was examined using the terminal deoxynucleotidyl transferase-mediated dUTP nick end labeling (TUNEL) method. The TUNEL assay was performed using the *In Situ* Cell Death Detection Kit (Cat. No. 11684817910, Roche Molecular Biochemicals, Germany) according to the manufacturer’s instructions. Sections (4 μm thick) were transferred to polylysine-coated slides and incubated overnight at 60°C. Then, they were deparaffinized in xylene. Sections were rehydrated in a graded series of 75%, 80%, 90%, and 100% ethanol and washed with PBS. Slides immersed in 0.1 M citrate buffer were microwaved at 350 W for 5 min for antigen retrieval. PBS was used for washing. Sections were incubated with the TUNEL reaction mixture (labeling solution + terminal deoxynucleotidyl transferase (TdT) enzyme solution) at 37°C for 1 h in a humidified chamber and washed twice with PBS. After this step, Converter-POD was added, and the slides were incubated for 30 min at 37°C and then rinsed twice with PBS. Next, the sections were treated with 3,3-diaminobenzidine at room temperature and rinsed twice with PBS. Sections were counterstained with Harris’ hematoxylin for 10 min. The sections were dehydrated with a series of graded alcohol concentrations. Xylene was used as a clearing agent, and then, the sections were coverslipped with Entellan for light microscopy examination.

### Transmission electron microscopy

Mouse small intestine was harvested, cut into sections of 1 mm × 1 mm × 2 mm, and placed in a 1.5-ml Eppendorf tube (Servicebio Cat. No. G1101) containing 2.5% glutaraldehyde. The samples were then embedded, sectioned (2.5 μm), stained, and observed and imaged by transmission electron microscopy (HITACHI HT7800/HT7700). Image-Pro Plus 6.0 (Media Cybernetics, Inc., Rockville, MD, United States) software was used to measure the intercellular space (nm) at five equal positions with an intact intercellular space on each slide [reference: (El-Sokkary, 2001) 0.0 k, 500 nm ruler].

### Measurement of serum endotoxin levels

Mouse serum endotoxin levels were measured using ELISA kits (Quanzhou Ruixin Biological Technology Co. Ltd., China) according to the manufacturer’s protocols.

### Antibody arrays

RayBio Mouse Cytokine Antibody Arrays (QAM-TH17-1-1, RayBiotech, Norcross, GA) were employed to evaluate chrysin-mediated inhibition of signaling in the serum and testis according to the manufacturer’s specification. Eighteen different cytokines were evaluated: interleukin 1 beta (IL-1β), IL-2, IL-4, IL-5, IL-6, IL-10, IL-12p70, IL-13, IL-17, IL-17F, IL-21, IL-22, IL-23, IL-28, interferon gamma (IFN-γ), macrophage inflammatory protein 3 alpha (MIP-3α), transforming growth factor beta 1 (TGF β1), and tumor necrosis factor alpha (TNF-α). Each sample was prepared in triplicate. An Axon scanner 4000B with GenePix software was used to record fluorescence intensities.

### 16S rRNA sequencing and analysis

DNA from intestinal contents was prepared from animals in the alcohol, control, alcohol-FMT, and control-FMT groups using a fecal DNA extraction kit (DP712, Tiangen Company, Beijing, China); three biological replicates per group were analyzed. DNA quality was monitored on 1% agarose gels. The hypervariable V3–V4 region of the 16S-rDNA gene was amplified by PCR using the primers 341F: CCTACGGGNGGCWGCAG and 806R: GGACTACHVGGGTATCTAAT, where the barcode was an eight-base sequence unique to each sample. PCR was performed in 30-μL reactions composed of 15 μl of Phusion® High-Fidelity PCR Master Mix (New England Biolabs), 0.2 μM forward and reverse primers, and 10 ng of template DNA. Thermal cycling consisted of an initial denaturation step at 98°C for 1 min, followed by 30 cycles of denaturation at 98°C for 10 s, annealing at 50°C for 30 s, and elongation at 72°C for 30 s. Finally, the reactions were incubated at 72°C for 5 min. PCR products were detected on 2% agarose gels by electrophoresis and then purified using the GeneJET Gel Extraction Kit (Thermo Scientific). Sequencing libraries were generated using the Illumina TruSeq DNA PCR-Free Library Preparation Kit (Illumina, United States) according to the manufacturer’s recommendations, and index codes were added. Sequencing was performed using the Illumina NovaSeq 6,000 platform (Genecloud Co. Ltd., Chongqing, China). Sequences were analyzed using QIIME software package 2 (version 2020.2). The effective tags were clustered into operational taxonomic units (OTUs) of ≥97% similarity using the MOTHUR pipeline. The tag sequence with the highest abundance was selected as the representative sequence within each cluster. Bacterial alpha diversity was determined by performing a sampling-based OTU analysis and presented as the observed OTUs, Chao index, Shannon index, and Simpson index, which were calculated using the R program package “vegan.” Principal coordinate analysis (pCoA) was conducted using the R package^1^ to display and compare the beta diversity of the microbiome between samples. The Bray–Curtis metric distances, unweighted UniFrac distances, and weighted UniFrac distances were calculated with the phyloseq package. Bacterial taxonomic analyses and comparisons, including bacterial phyla, classes, orders, families, and genera, were conducted between two groups using the Wilcoxon rank-sum test. The predominance of bacterial communities between groups was analyzed using the linear discriminant analysis (LDA) effect size (LEfSe) method.^2^ Based on the normalized relative abundance matrix, features with significantly different abundance levels between assigned taxa were determined using LEfSe with the Kruskal–Wallis rank-sum test (*p* < 0.05), and LDA was used to assess the effect size of each feature [LDA score (log10) = 3.5 as the cutoff value]. Correlation analysis was performed by calculating Pearson’s correlation coefficient using the R language mixOmics package to calculate the correlation coefficient r^2^ and the *p* value of differentially intestinal flora and differentially metabolites. All 16S rRNA datasets are publicly available (NCBI SRA accession numbers: SRP789987 for mouse intestinal content samples).

### Nontargeted metabolomics analysis

Tissues (100 mg) were individually ground in liquid nitrogen, and the homogenate was resuspended in prechilled 80% methanol by thoroughly vortexing the mixture. The samples were incubated on ice for 5 min and then centrifuged at 15,000 × *g* for 20 min at 4°C. Some of the supernatant was diluted with liquid chromatography–mass spectrometry (LC–MS)-grade water to a final concentration of 53% methanol. The samples were subsequently transferred to a fresh Eppendorf tube and then centrifuged at 15,000 × *g* for 20 min at 4°C. Finally, the supernatant was injected into a liquid chromatography tandem mass spectrometry (LC–MS/MS) system. Ultrahigh-performance liquid chromatography (UHPLC)-MS/MS analyses were performed using a Vanquish UHPLC system (Thermo Fisher, Germany) coupled with an Orbitrap Q Exactive™ HF-X mass spectrometer (Thermo Fisher, Germany) at Novogene Co., Ltd. (Beijing, China). The raw data files generated by the UHPLC–MS/MS system were processed using the Compound Discoverer 3.1 platform (CD3.1, Thermo Fisher) to perform peak alignment, peak picking, and quantitation of each metabolite. The normalized data were used to predict the molecular formula based on additive ions, molecular ion peaks, and fragment ions. Then, peaks were matched with the mzCloud,^3^ mzVault, and MassList databases to obtain accurate qualitative and relative quantitative results. Statistical analyses were performed using the statistical software R (R version R-3.4.3), Python (Python version 2.7.6), and CentOS (CentOS release 6.6). When data were not normally distributed, normal transformations were attempted using the area normalization method.

These metabolites were annotated using the Kyoto Encyclopedia of Genes and Genomes (KEGG) database,^4^ Human Metabolome Database (HMDB),^5^ and LIPID MAPS database.^6^ Principal component analysis (PCA) and partial least squares discriminant analysis (PLS-DA) were performed using metaX (a flexible and comprehensive software for processing metabolomics data). We employed a univariate analysis (*t* test) to calculate statistical significance (*p*-value). Metabolites with a variable importance in projection (VIP) value > 1, *p*-value < 0.05, and fold change (FC) ≥ 2 or ≤ 0.5 were considered differentially altered metabolites. Volcano plots were constructed to filter the metabolites of interest based on the log2 (FC) and log10 (*p*-value) of metabolites. The functions of these metabolites and metabolic pathways were analyzed using the KEGG database. Next, we evaluated the metabolic pathway enrichment of the differentially altered metabolites. When the ratios were satisfied by x/n > y/N, the metabolic pathway was considered enriched, while when the p value of the metabolic pathway was <0.05, the enrichment of the metabolic pathway was considered statistically significant. Correlation analysis was performed by calculating Pearson’s correlation coefficient using the R language mixOmics package to calculate the correlation coefficient r^2^ and the *p*-value of differentially expressed genes and differentially expressed metabolites. All differentially expressed genes and differentially altered metabolites obtained in the alcohol-FMT group compared with the control-FMT group were mapped to the KEGG database to obtain their common pathway information and for statistical analysis.

### Transcriptome sequencing and data analysis

Total RNA was extracted from the testicular tissues of animals in the alcohol-FMT and control-FMT groups, with three replicates in each group. RNA integrity was assessed using an RNA Nano 6000 Assay Kit and a Bioanalyzer 2100 system (Agilent Technologies, Santa Clara, CA, United States). The cDNA libraries were prepared using an Illumina TruSeq RNA Sample Prep Kit. The average size of the library cDNAs was 150 bp (excluding adapters). The integrity and quality of cDNA libraries were assessed using an Agilent 2100 Bioanalyzer and an ABI StepOne Plus real-time PCR system. RNA sequencing (RNA-seq) was performed by Novogene Company (Tianjin, China). RNA-seq reads from the FASTQ files were mapped to the mouse reference genome using TopHat2. The accession number for the RNA-seq data is GEO GSE192522.

The original data were filtered to obtain clean data and ensure the quality and reliability of the analysis. Trinity software was used for transcript assembly. Corset hierarchical clustering was performed for splicing. BUSCO software was used to evaluate the splicing quality of Trinity.fasta, unigene.fa, and cluster.fasta files. The gene function was annotated using the following databases: Nr [National Center for Biotechnology Information (NCBI nonredundant protein sequences), Nt (NCBI nonredundant nucleotide sequences), Pfam (protein family), KOG/COG (clusters of orthologous groups of proteins), Swiss-Prot (a manually annotated and reviewed protein sequence database), KO (KEGG Orthology database), and GO (Gene Ontology)]. The differential expression analysis of the two conditions/groups was performed using the DESeq2 R package (1.20.0). DESeq2 provides statistical routines for determining differential expression in digital gene expression data using a model based on a negative binomial distribution. The resulting *p* values were adjusted using the Benjamini–Hochberg approach to control the false discovery rate. Genes with an adjusted *p*-value < 0.05 identified by DESeq2 were considered differentially expressed. The GO enrichment analysis of differentially expressed genes was performed using the clusterProfiler R package, and gene length bias was corrected. We used the clusterProfiler R package to test the statistical enrichment of differentially expressed genes in KEGG pathways.

### Statistical analysis

The data are expressed as the mean ± standard error of the mean (SEM). Statistical comparisons between two measurements were analyzed by independent-samples t tests using SPSS 26.0 software (SPSS, Inc., Chicago, IL, United States). When analyzing gut microbiota sequencing data, we performed a two-tailed Wilcoxon rank-sum test by R Project; *p* < 0.05 was considered statistically significant.

## Results

### Mouse intestinal dysbiosis and impaired sperm quality induced by 10% ethanol

**Figure 2:**
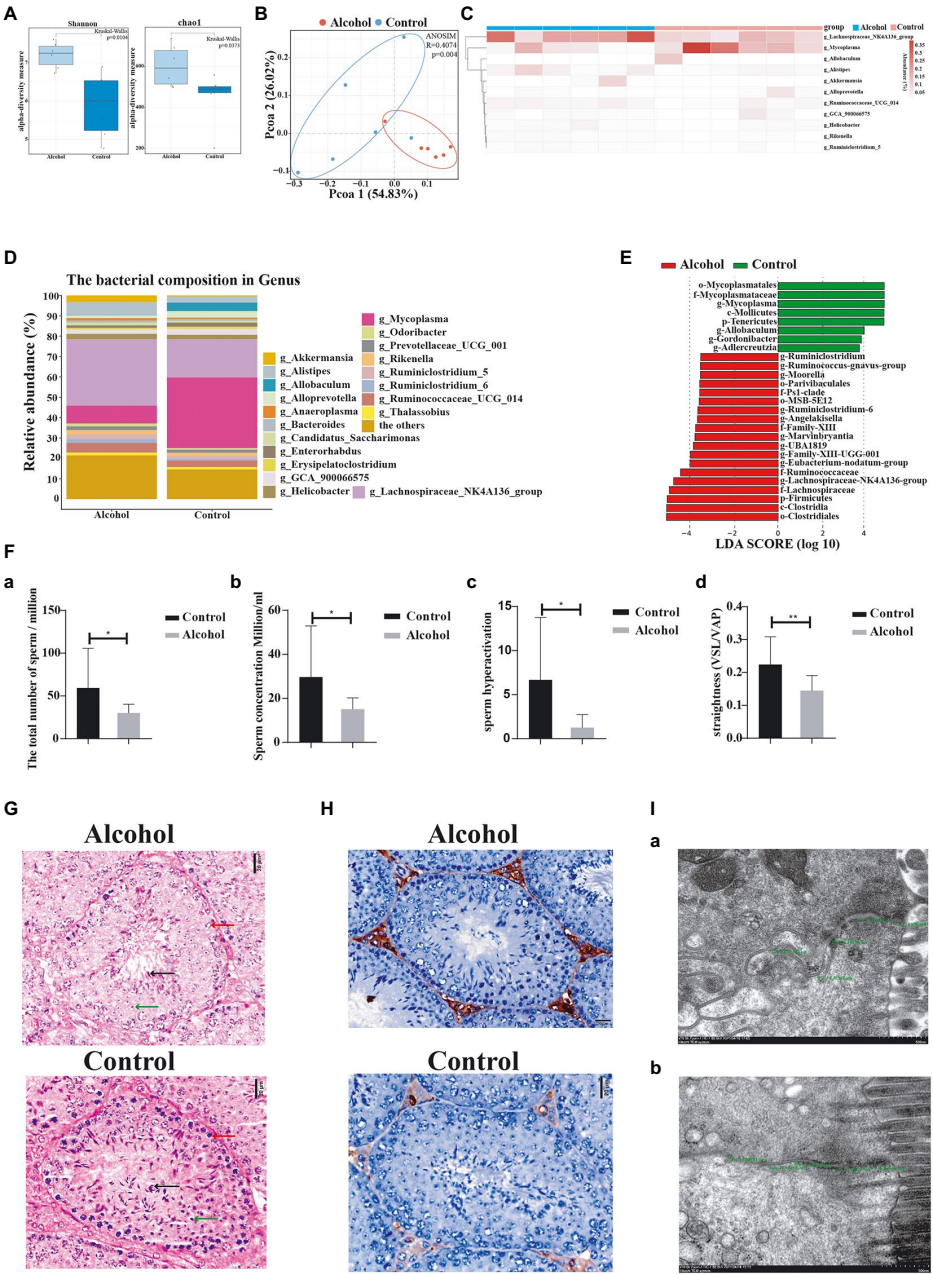
Chronic alcohol consumption (alcohol) altered the gut microbiota and impaired sperm quality. **(A)** Alpha diversity (based on the Shannon and Chao1 index). **(B)** pCoA of beta diversity. **(C)** Heatmap of selected differentially abundant features. **(D)** Relative abundance of the indicated genus. **(E)** LEfSe indicating differences in the bacterial taxa between alcohol and control group mice. Green bars indicate taxa enriched in control mice, and red bars indicate taxa enriched in alcohol-treated mice. **(F)**—**(a)** The total number of sperm, **(b)** the concentration of sperm, **(c)** hyperactivation of sperm, and **(d)** the straightness of sperm. **(G)** Representative images of H&E staining showing impairment of the seminiferous tubules in the testis. Scale bar = 20 μm. **(H)** Immunohistochemical determination of macrophage infiltration in the testis of alcohol-treated mice. Scale bar = 50 μm. **(I)** Transmission electron microscopy images measuring the intercellular space. **(a)** Alcohol and **(b)** control groups. ^*^p < 0.05 and ^**^p < 0.01.

Through its interaction with the host, the gut microbiota plays an important role in human diseases. We established a mouse model of chronic drinking (10% ethanol; alcohol) to investigate the effect of chronic drinking-induced intestinal dysbiosis on sperm quality. No significant difference was observed in body weight between the alcohol group and the control group (*p* = 0.479, Supplementary Table 2). We harvested the intestinal contents from each group for 16S rDNA sequencing and found that chronic drinking induced significant changes in the gut microbiota. We measured alpha diversity using the Shannon index, Chao1 index, ACE index, Simpson index, observed features, ENSPIE, and Fisher’s alpha (Figure 2A; Supplementary Figure S1A). Most of these indices manifested similar tendencies, as the gut microbiota of mice in the alcohol group exhibited a higher alpha diversity than that of control mice (*p* = 0.0104, *p* = 0.0373, *p* = 0.0373, *p* = 0.0249, *p* = 0.0373, *p* = 0.0249, and *p* = 0.0373, respectively). The beta diversity based on weighted PCoA clearly showed a difference in the microbiota composition between the control group and the alcohol group, with a significant difference along the PCO1 axis (accounting for 54.83% of the overall variation, Figure 2B). Next, we compared the species composition of the gut microbiota between the alcohol group and the control group at different levels. Chronic drinking induced significant differences in the gut microbiota at different levels (Supplementary Figure S1B). The relative abundance of the dominant phyla (> 0.5%) Bacteroides and Tenericutes decreased significantly, while that of the phylum Firmicutes increased significantly. The relative abundance levels of the dominant classes (> 0.5%) Bacteroidia, Mollicutes, and Erysipelotrichia decreased significantly, while those of the classes Clostridia and Verrucomicrobia increased significantly. The relative abundance of the dominant orders (> 0.5%) Bacteroidales, Mycoplasmatales, and Erysipelotrichales decreased significantly, while that of the orders Clostridiales and Verrucomicrobiales increased significantly. At the family level, the relative abundance of the dominant taxa (> 0.5%) Erysipelotrichaceae, Muribaculaceae, and Mycoplasmataceae decreased significantly, while that of Akkermansiaceae, Lachnospiraceae, Rikenellaceae, and Ruminococcaceae increased significantly. At the genus level, the relative abundance of the dominant taxa (˃0.5%) *Allobaculum*, *Alloprevotella, Adlercreutzia*, *Gordonibacter*, and *Mycoplasma* decreased significantly, while that of *Alistipes*, *Eubacterium_nodatum*_group, *Lachnospiraceae*_NK4A136_group, Family_XIII_UCG_001, *Angelakisella*, *Ruminiclostridium*_5, *Ruminiclostridium*_6, UBA1819, *Moorella*, *Marvinbryantia*, *Ruminococcus__gnavus* _ group, and *Ruminococcaceae*_UGG_014 increased significantly (Figure 2C). The heatmap shows the most differentially abundant features selected at the genus level between mice in the alcohol and control groups (Figure 2D). We performed high-dimensional class comparisons using LEfSe (LDA score > 3.5) to identify bacterial biomarkers of the two groups (Figure 2E). Collectively, the gut microbiota differed between mice in the alcohol and control groups.

We also investigated the effect of chronic drinking on male sperm quality. Sperm count, sperm concentration, sperm hyperactivation, and forward progression were significantly reduced in the alcohol group (Figure 2F), along with significantly decreased numbers of spermatocytes (red arrow), round spermatids (green arrow), and sperm (black arrow) in the seminiferous tubules (Figure 2G), and the infiltration of a largenumber of macrophages in the testicular interstitium (Figure 2H). These findings are consistent with previous reports (Anderson et al., 1982; Talebi et al., 2011; Ezzatabadipour et al., 2012; Chen and Li, 2014).

Furthermore, we observed intestinal mucosal epithelial cells and cell junction complexes with transmission electron microscopy. The small intestinal mucosa was significantly more permeable in the alcohol group than in the control group (Figure 2I; Supplementary Table 3), which manifested as a widened gap junction. Intestinal dysbiosis increases the permeability of the intestinal mucosa, which aggravates intestinal dysbiosis and causes gut metabolites to enter the blood circulation through the intestinal barrier. In summary, chronic alcohol consumption induces intestinal dysbiosis and significantly affects sperm quality in mice.

### Significantly elevated serum endotoxin levels and impaired sperm quality in the alcohol-FMT group

We investigated the effect of chronic drinking-induced intestinal dysbiosis on sperm quality by transplanting the fecal microbiota from the alcohol group into the intestine of normal mice by gavage and then analyzed the sperm from the alcohol-FMT group and control-FMT group. At the end of FMT, no significant difference was observed in the mean body weight between the alcohol-FMT group and the control-FMT group (21.58 ± 3.87 g vs. 23.42 ± 3.49 g, *p* > 0.05; Supplementary Table 4). Sperm counts, sperm concentrations and sperm hyperactivation were significantly lower in the alcohol-FMT group than in the control-FMT group. The straightness (straight-line (rectilinear) velocity/average path velocity) of sperm declined but not significantly (Figure 1B), along with significantly decreased numbers of spermatocytes and sperm in the seminiferous tubules (Figure 1C). The cell apoptosis analysis (TUNEL) results further showed increased apoptosis of spermatocytes in the seminiferous tubules of the alcohol-FMT group compared with the control-FMT group, although the difference did not reach statistical significance (Figures 1D,E). Moreover, serum endotoxin levels were significantly higher in the alcohol-FMT group than in the control-FMT group (Figure 1F). Based on these findings, FMT from the alcohol group into normal mice increases serum endotoxin levels and impairs sperm quality in otherwise normal mice.

### Significant post-FMT differences in the gut microbiota between the alcohol-FMT group and the control-FMT group

**Figure 3:**
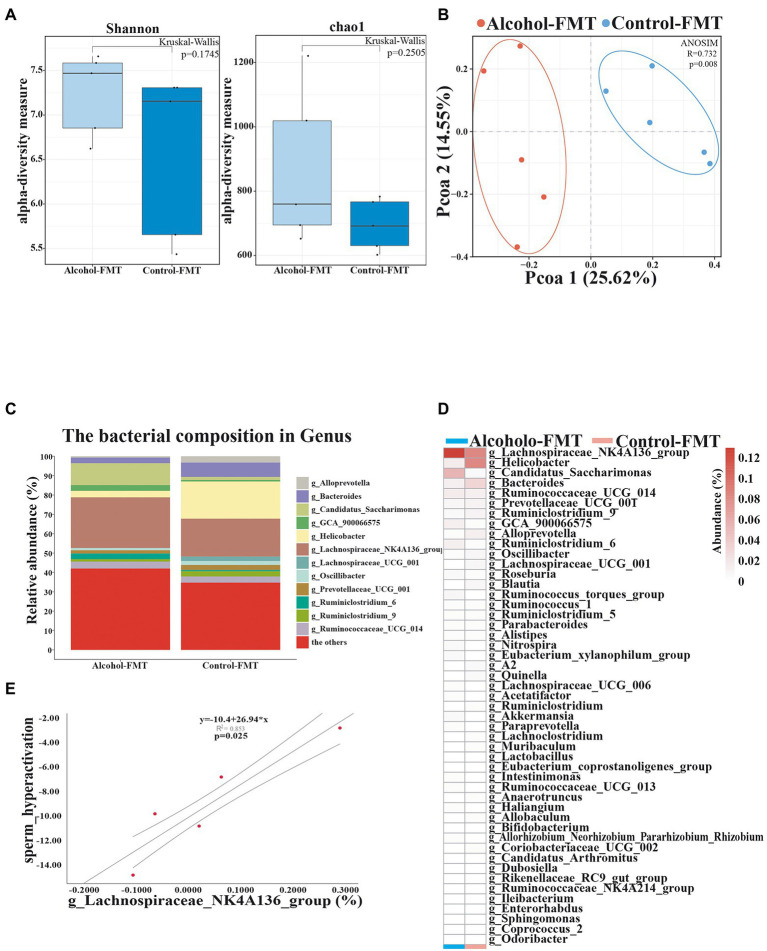
Transferring microbiota from alcohol-treated mice alters the gut microbiota community of normal mice. **(A)** Alpha diversity (based on the Shannon and Chao1 index). **(B)** PCoA of the beta diversity metric distance. **(C)** Relative abundance of the indicated genus. **(D)** Heatmap of selected differentially abundant genera. **(E)** The correlation between sperm hyperactivation and *Lachnospiraceae*_NK4A136_group abundance. ^*^p < 0.05 and ^**^p < 0.01.

The intestinal contents were harvested from the alcohol-FMT group and control-FMT group for high-throughput 16S rDNA sequencing. We measured alpha diversity using the Shannon index, Chao1 index, ACE index, Simpson index, observed features, ENSPIE, and Fisher’s alpha (Figure 3A; Supplementary Figure S3A). Most of the indices exhibited similar tendencies, although the gut microbiota of the alcohol-FMT group displayed higher alpha diversity than that of the control-FMT group, but the difference did not reach statistical significance (*p* = 0.1745, *p* = 0.2505, *p* = 0.2505, *p* = 0.4647, *p* = 0.2505, *p* = 0.4647, and *p* = 0.2505, respectively), which was consistent with the results described above. A distinct clustering of the microbiota composition for the two groups was observed using Bray–Curtis-based pCoA (Figure 3B). Next, we analyzed the microbiota composition and observed intergroup differences in species composition at different levels (Supplementary Figure S3B). At the phylum level, the relative abundance levels of the dominant taxa (˃0.5%) Bacteroides and Epsilonbacteraeota decreased, while those of Patescibacteria and Cyanobacteria increased. At the class level, the relative abundance of the dominant taxa (> 0.5%) Bacteroidia, Campylobacter, and Erysipelotrichia decreased, while that of Anaerolineae, Oxyphotobacteria, and Saccharimonadia increased. The relative abundance of the dominant orders (> 0.5%) Bacteroidales, Campylobacterales, and Erysipelotrichales decreased, while that of the orders Chloroplast, Clostridiales, and Saccharimonadales increased. At the family level, the relative abundance of the dominant taxa (> 0.5%) Bacteroidaceae, Erysipelotrichaceae, Helicobacteraceae, Muribaculaceae, and Prevotellaceae decreased, while that of Lachnospiraceae and Saccharimonadaceae increased. At the genus level, differential clustering and features were observed between the alcohol-FMT group and the control-FMT group. The relative abundance of the dominant genera (> 0.5%) Helicobacter, Bacteroides, Oscillibacter, Lachnospiraceae_UCG_001, Blautia, and Alloprevotella decreased, while that of the genera Lachnospiraceae_NK4A136_group, Ruminococcaceae_UCG_014, Ruminiclostridium_6, Ruminococcus_torques_group, and GCA_900066575 increased (Figure 3C). The heatmap shows the most differentially abundant features selected at the genus level between the alcohol-FMT and control-FMT mice (Figure 3D). Some of the changes described above were also observed between the alcohol group and the control group.

As described above, serum endotoxin levels were significantly elevated in the alcohol-FMT group. Pearson’s correlation analysis showed that serum endotoxin levels were correlated with the relative abundance of *Lachnospiraceae*_NK4A136_group, *Helicobacter*, *Bacteroides*, *Candidatus_Saccharimonas*, and *Akkermansia*, although the intergroup difference did not reach statistical significance (Pearson’s correlation coefficient, *p* ≥ 0.05). Pearson’s correlation analysis of sperm quality and the relative abundance of gut microbiota showed a significant negative correlation between sperm motility and the abundance of *Lachnospiraceae*_NK4A136_group (*p* = 0.025) (Figure 3E). These findings indicate that transplantation of the fecal microbiota from the alcohol group to normal mice also altered the gut microbiota and led to impaired sperm quality in otherwise normal mice. We suggest that *Lachnospiraceae*_NK4A136_group may be one of the most important species causing impaired sperm quality, although further research is needed to investigate the mechanism.

### Significant differences in gut metabolites between the alcohol-FMT group and the control-FMT group are due to the differences in the gut microbiota

**Figure 4:**
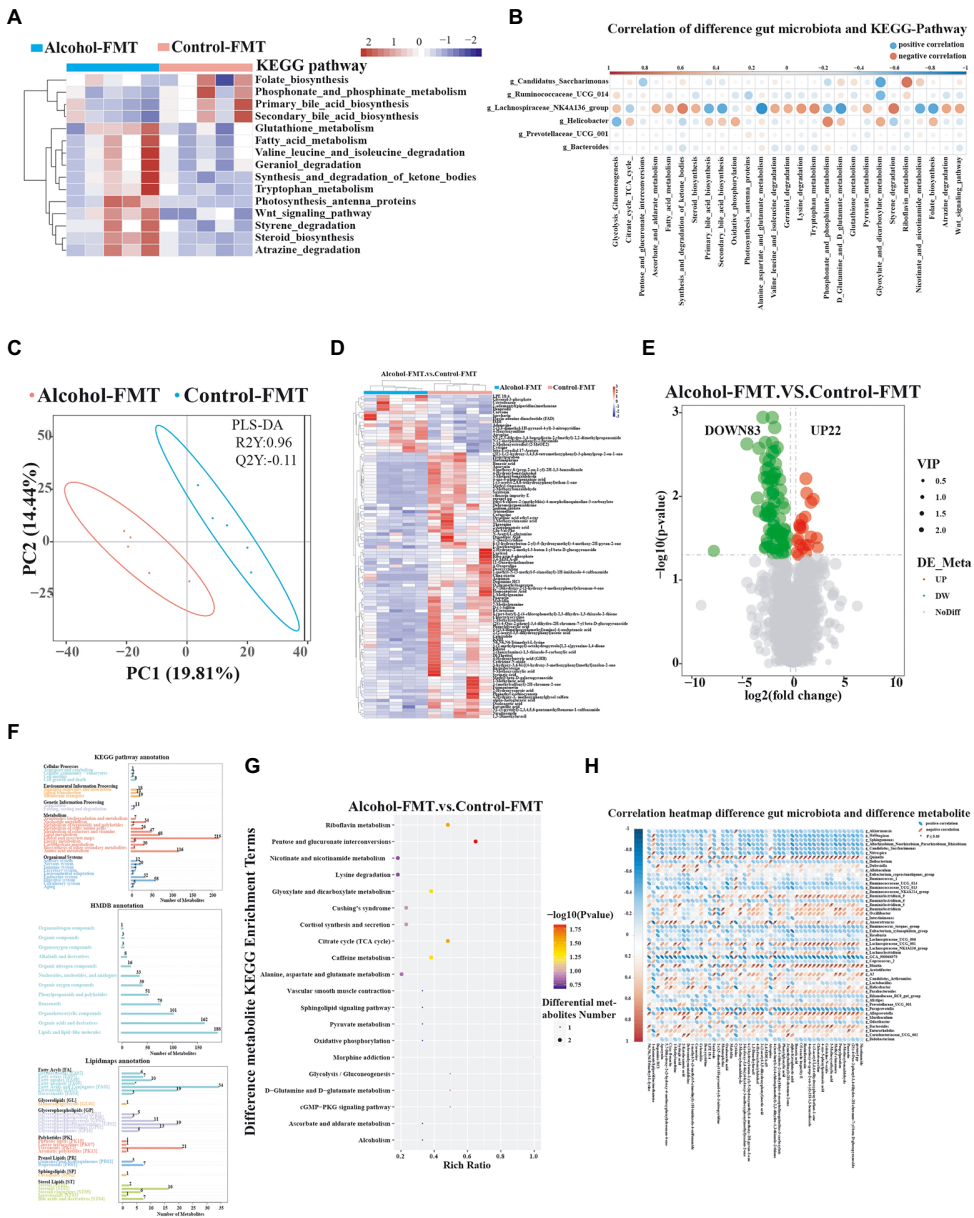
The alteration in the gut microbiota of the alcohol-FMT group was closely associated with differences in intestinal metabolites. **(A)** KEGG pathway analysis (top 15 different pathways). **(B)** Correlation map of differentially enriched KEGG pathways and differentially abundant bacteria. **(C)** PLS-DA score plot discriminating the intestine digesta metabolome from the control-FMT and alcohol-FMT groups. **(D)** Heatmaps of the differentially altered metabolites. **(E)** Volcano plot showing the changes in the intestine digesta metabolome (log2 fold change ≥ 1.5). **(F)** Metabolite pathways annotated for all metabolites based on the KEGG database, HMDB, and LIPID MAPS database. **(G)** KEGG pathway enrichment bubble diagram of differentially altered metabolites. **(H)** Correlation heatmap of differentially abundant gut microbiota and differentially altered metabolites from the alcohol-FMT and control-FMT groups. Blue represents a positive correlation, red represents a negative correlation, and a darker color corresponds to a stronger correlation. ^*^p < 0.05 and ^**^p < 0.01.

As described above, the difference in the gut microbiota may be an important factor contributing to impaired sperm quality in the alcohol-FMT group. We adopted KEGG pathway analysis to detect the relative abundance levels of metabolic pathways related to the gut microbiota to investigate how chronic drinking-induced intestinal dysbiosis affects spermatogenesis. Fifteen KEGG pathways differed between alcohol-FMT and control-FMT mice (Figure 4A). In particular, the primary and secondary bile acid biosynthesis pathways were significantly less enriched in the alcohol-FMT group, although the intergroup difference did not reach statistical significance (*p* = 0.081 and *p* = 0.73, respectively). The correlation analysis result of differentially enriched KEGG pathways with the species at the genus level showed that differentially abundant microbiota species were enriched in a series of metabolic pathways (Figure 4B), such as steroid biosynthesis, Wnt signaling pathway, primary bile acid biosynthesis, secondary bile acid biosynthesis, folate biosynthesis, and oxidative phosphorylation, all of which have been shown to be critical for spermatogenesis (Ding et al., 2020; Zhang et al., 2021). Next, we performed UHPLC–MS/MS to analyze the nontargeted metabolomics of the intestinal contents from the alcohol-FMT group and the control-FMT group (Supplementary Table 5). PLS-DA showed apparent metabolite clustering in both groups (Figure 4C). Transplantation of the fecal microbiota from the alcohol group to normal mice caused extensive changes in the gut metabolites in otherwise normal mice (Figure 4D). A total of 105 differentially altered metabolites were identified, including 22 upregulated metabolites and 83 downregulated metabolites in the alcohol-FMT group compared with the control-FMT group (Figure 4E). These metabolites were annotated to metabolic pathways related to amino acids, lipids and lipid molecules, fatty acids and conjugates, steroids, and flavonoids using the KEGG database, HMDB, and LIPID MAPS database (Figure 4F). The differentially altered metabolites were enriched in 20 KEGG pathways, including pentose and glucuronate interconversions (*p* = 0.0150), riboflavin metabolism (*p* = 0.0287), nicotinate and nicotinamide metabolism (*p* = 0.1638), morphine addiction (*p* = 0.0739), lysine degradation (p = 0.1638), glyoxylate and dicarboxylate metabolism (*p* = 0.0456), cortisol synthesis and secretion (*p* = 0.1115), citrate cycle (tricarboxylic acid (TCA) cycle) (p = 0.0287), caffeine metabolism (p = 0.0456), alanine, aspartate, and glutamate metabolism (*p* = 0.1370), and oxidative phosphorylation (*p* = 0.2065; Figure 4G), which was consistent with the KEGG analysis results of the 16S sequencing data. The significantly differentially altered metabolites enriched in these metabolic pathways included alpha-ketoglutaric acid, oxaloacetic acid, ribitol, riboflavin-5-phosphate, 1-methylxanthine, and 1-methyluric acid (Supplementary Table 6). Analyses of the 16S rDNA sequencing data and nontargeted metabolomics data identified a positive or negative correlation between 49 differentially abundant microbiota species and 50 differentially altered metabolites (Figure 4H), indicating that changes in the gut microbiota caused changes in the gut metabolites. Interestingly, the differentially altered metabolites were mainly lipids and amino acids, with a significantly less enriched bile acid metabolic pathway in the alcohol-FMT group. In summary, the differences in the gut microbiota between the alcohol-FMT group and the control-FMT group caused significant differences in gut metabolites, which is one possible explanation for the difference in sperm quality, although further research is needed to validate the results.

### In the alcohol-FMT group, intestinal dysbiosis-induced intestinal inflammation affects the host testes

**Figure 5:**
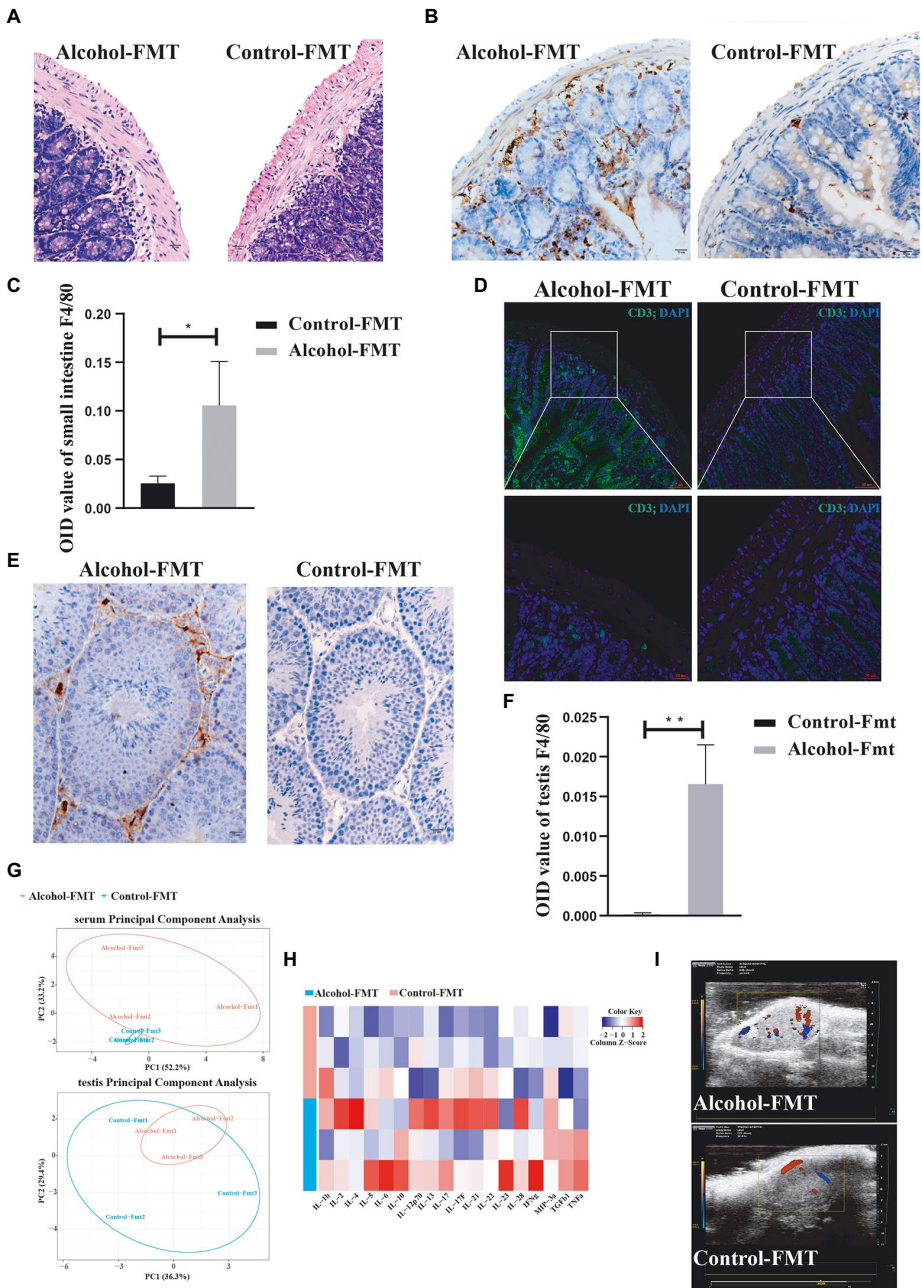
Alcohol**-**FMT mice showed dysbiosis of the gut microbiota, which led to intestinal CD3+ T-cell and macrophage aggregation and testis inflammation. **(A)** Histological examination of FMT-treated intestines. Scale bar = 50 μm. **(B)** Immunohistochemical determination of macrophage infiltration in the small intestines. Scale bar = 50 μm. **(C)** F4/80 histological score. **(D)** Immunofluorescence staining for CD3 (green) was performed in the small intestine samples. Nuclei were stained with DAPI (blue). The lower panels show higher magnification images of the upper panels. Scale bar = 20 μm. **(E)** Immunohistochemical determination of macrophage infiltration in the testis. Scale bar = 50 μm. **(F)** F4/80 histological score. **(G)** PCA of the serum and testis inflammatory factors. **(H)** Heatmap of the abundance of 18 inflammatory factors. **(I)** Ultrasonic evaluation of testicular blood flow using the Vevo 2,100 imaging system. Red and blue represent blood flow; red represents positive blood flow, and blue represents reverse blood flow. ^*^p < 0.05 and ^**^p < 0.01.

As described above, serum endotoxin levels were elevated in the alcohol-FMT group, which was correlated with certain microbiota, especially the dominant phyla Firmicutes and Bacteroides. We first analyzed intestinal inflammation in the recipient mice. H&E staining showed pronounced lymphocyte infiltration in the lamina propria of the small intestine in the alcohol-FMT group compared with the control-FMT group (Figure 5A). Immunohistochemistry and immunofluorescence staining also showed that a large amount of macrophages (Figures 5B,C) and T cells (Figure 5D) had infiltrated the lamina propria of the small intestine in alcohol-FMT recipient mice, suggesting that chronic drinking induced changes in the gut microbiota to promote inflammation. The testes and epididymis are where sperm are produced, mature, and are mobilized (Cornwall, 2009; Mäkelä et al., 2019), and they are the foundation of male fertility and reproductive health. Elevated endotoxin levels cause testicular and epididymal inflammation and affect spermatogenesis and sperm motility (Cao et al., 2010; Wang et al., 2019). Testicular immunohistochemistry also revealed that a large number of macrophages infiltrated the testicular interstitium in the alcohol-FMT group compared with the control-FMT group (Figures 5E,F), indicating the presence of inflammation. The results described above confirmed that FMT from the alcohol group to normal mice increased serum endotoxin levels and caused chronic inflammation in otherwise normal mice. We used antibody array technology to analyze the serum levels of 18 specific inflammatory cytokines in the alcohol-FMT and control-FMT groups. PCA showed differential clustering, with a significant difference along the PCO2 axis (responsible for 52.2% of the overall change rate, Figure 5G). All 18 inflammatory cytokines were present at higher levels in the alcohol-FMT group than in the control-FMT group, although the difference did not reach statistical significance in most cases (Figure 5H). In particular, serum IL-12p70 (*p* = 0.0148) and IL-22 (*p* = 0.0246) levels were significantly higher in the alcohol-FMT group, indicating significantly increased inflammation in this group compared with the control-FMT group. We also analyzed inflammatory cytokine levels in the testes and detected higher levels of the proinflammatory cytokines IL-2 and IL-Ib and lower levels of the anti-inflammatory cytokines TGF β1, IL-12p70, and IL-22 in the alcohol-FMT group than in the control-FMT group, although the differences did not reach statistical significance (*p* > 0.05).

Studies have demonstrated that chronic inflammation causes sustained activation of endothelial cells, which promotes angiogenesis, increased blood flow, and macrophage aggregation (Cao et al., 2010). We observed the testicular blood flow in each group using the Vevo 2,100 imaging system (Visual sonic Inc., Toronto ON, Canada) with a 30-MHz phased array transducer (MS400) at a frame rate of 235/s. The results indicated increased testicular blood flow in the alcohol-FMT group compared with the control-FMT group (Figure 5I), which, along with ultrasound images, confirmed that FMT from the alcohol group to normal mice increased testicular blood flow and the level of testicular inflammation in otherwise normal mice. In summary, FMT from the alcohol group to normal mice induced changes in the gut microbiota, which caused intestinal and testicular inflammation and ultimately impaired sperm quality in the alcohol-FMT group.

### Chronic drinking-induced intestinal dysbiosis alters testicular gene expression

**Figure 6:**
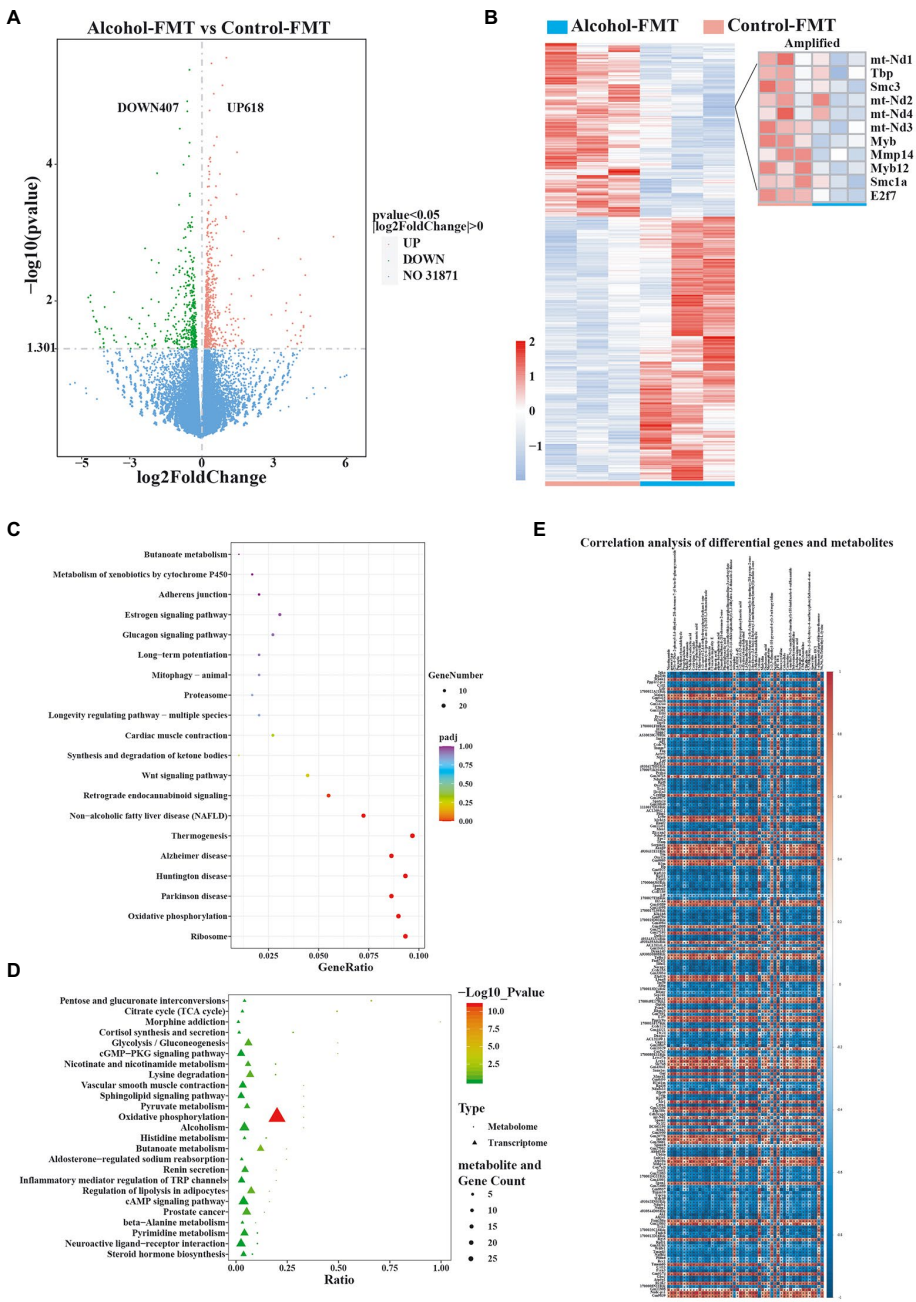
Alterations in gene expression in alcohol-FMT-treated testes. **(A)** Volcano plot showing the changes in the expression of testis-related genes (*p* value<0.05). **(B)** Heatmap of differentially expressed genes in the testis. **(C)** KEGG pathway enrichment analysis of differentially expressed genes. **(D)** The KEGG coenrichment bubble map was drawn using the common enriched pathways of differentially altered metabolites and differentially expressed genes. **(E)** Correlation analysis of differentially expressed genes and metabolites. The results of the correlation analysis showing the top 50 differentially altered metabolites (*p*-values ranging from low to high) and the top 100 differentially expressed genes (*p* values ranging from low to high). Red represents a positive correlation, blue represents a negative correlation, and a darker color corresponds to a stronger correlation. ^*^p < 0.05 and ^**^p < 0.01.

As described above, FMT from the alcohol group impaired sperm quality. We next investigated the mechanism of impaired sperm quality in the alcohol-FMT group by collecting testis samples from the alcohol-FMT group and the control-FMT group for transcriptome sequencing, with fragments per kilobase per million (FPKM) ≥ 1 as the threshold for gene expression. Among the eligible genes, 1,025 genes showed significant differences in expression (*p* ≤ 0.05, or |log2 FC| > 0), including 618 upregulated genes and 407 downregulated genes. We then used the H-cluster method with log2(FPKM + 1) to analyze differential expression and centralization correction to observe clustering (Figures 6A,B). Moreover, KEGG pathway analysis revealed a series of enriched pathways, such as ribosome (*p* = 3.14E-12), oxidative phosphorylation (*p* = 1.16E-11), Wnt signaling pathway (*p* = 0.0077), synthesis and degradation of ketone bodies (*p* = 0.0091), proteasome (*p* = 0.0403), thermogenesis (*p* = 1.52E-07), and retrograde endocannabinoid signaling (*p* = 0.0003) (Figure 6C), all of which play important roles in spermatogenesis and sperm quality. In particular, the expression levels of the mitochondrial genes MT-ND1 (mitochondrially encoded NADH:ubiquinone oxidoreductase core subunit 1), MT-ND2, MT-ND3, and MT-ND4, which encode different subunits of the mitochondrial dehydrogenase complex; oncogenes Myb and Myb12, which regulate cell growth and differentiation; E2F transcription factor 7 (E2F7) and matrix metallopeptidase 14 (MMP14), which regulate cell cycle progression; chromosomal structure maintenance protein genes structural maintenance of chromosomes 1A (Smc1a) and Smc3, which are involved in the regulation of meiosis; and the spermatocyte-specific regulatory factor TATA-binding protein (Tbp) were significantly decreased (Figure 6B).

Next, we performed differential KEGG pathway coenrichment analysis using metabolomics and transcriptomics data with KEGG pathways as the entry and the R language ggplot2 package to plot a KEGG coenrichment bubble chart of the differentially altered metabolites and the differentially expressed genes. Oxidative phosphorylation, the key pathway required for spermatogenesis, was significantly enriched in both transcriptomics and metabolomics data (*p* = 1.16E-11) (Figure 6D). Oxidative phosphorylation was also a differentially enriched metabolic pathway in the KEGG pathway analysis of the gut microbiota data. Next, we calculated the correlation coefficient r^2^ and *p*-value to identify the correlations between the differentially expressed genes and the differentially altered metabolites with the R language mixOmics package and Pearson’s correlation analysis. The results indicated a significant correlation between 200 differentially expressed genes and 50 differentially altered metabolites (Figure 6E). In summary, FMT from the alcohol group to normal mice affected sperm quality by inducing intestinal dysbiosis and abnormal expression of testis-related genes in otherwise normal mice.

## Discussion

**Figure 7:**
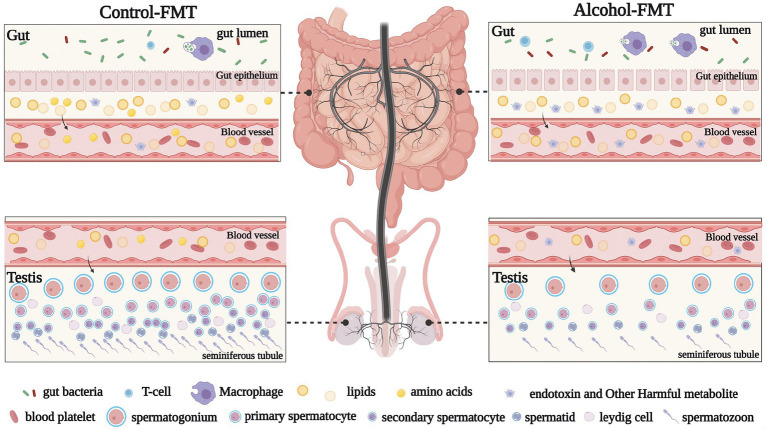
Graphical abstract. Diagram illustrating that alcohol affects sperm quality via the gut microbiota-gut-testis axis. Notably, the effect of chronic drinking on the sperm of male mice can be transferred to the testes of new male mice via gut microbiota exposed to chronic drinking, which leads to abnormal sperm quality in the alcohol-FMT model.

As demonstrated in the present study, chronic drinking-induced intestinal dysbiosis may be one of the most important mechanisms by which chronic alcohol consumption affects male sperm quality. Specifically, intestinal dysbiosis reduces the amount of bacteria with anti-inflammatory activity; increases the amount of proinflammatory bacteria; increases bacterial endotoxin levels; causes intestinal inflammation, intestinal mucosal injury, intestinal hyperpermeability, and leaky gut; causes a large amount of bacterial metabolites to enter the blood circulation; alters serum cytokine levels; and ultimately results in testicular inflammation and abnormal expression of testis-related genes. To the best of our knowledge, this study is the first to investigate the functional connection between chronic drinking-induced intestinal dysbiosis and impaired male fertility. Further research is needed to investigate the role of the gut-testicular axis in the etiology of male infertility (Figure 7).

Chronic drinking disrupts the intestinal microecosystem (Engen et al., 2015; Capurso and Lahner, 2017; Wang et al., 2020; Calleja-Conde et al., 2021). We established a mouse model of chronic drinking, performed 16S sequencing of the gut microbiota from the alcohol group to investigate the effect of chronic drinking on the gut microbiota, and found significant intergroup differences in the gut microbiota at different taxonomic levels. At the genus level, the relative abundance of the dominant taxa (> 0.5%) *Allobaculum*, *Alloprevotella*, *Adlercreutzia*, *Gordonibacter*, and *Mycoplasma* decreased significantly, while that of *Alistipes*, *Eubacterium_nodatum*_group, *Lachnospiraceae*_NK4A136_group, Family_XIII_UCG_001, *Angelakisella*, *Ruminiclostridium*_5, *Ruminiclostridium*_6, UBA1819, *Moorella*, *Marvinbryantia*, *Ruminococcus__gnavus*_group, and *Ruminococcaceae*_UGG_014 increased significantly in the alcohol group. Further analysis revealed that *Lachnospiraceae*_NK4A136_group, a dominant genus of Firmicutes, and *Helicobacter*, a dominant genus of Bacteroides, may be the key intestinal bacterial species modulating sperm quality. Further studies are needed to confirm the specific mechanism of their effect on sperm quality. Sperm analysis indicated that the sperm count, sperm concentration, and sperm motility were significantly reduced in the alcohol group compared with the control group. Electron microscopy, H&E staining, and immunohistochemistry showed intestinal hyperpermeability and increased intestinal inflammation in the alcohol group. TUNEL staining revealed more apoptotic spermatocytes in the seminiferous tubules in the alcohol group than in the control group. Based on these findings, chronic drinking induces changes in the gut microbiota and affects sperm quality in mice.

We investigated whether the aforementioned findings were caused by chronic drinking-induced intestinal dysbiosis by collecting fresh feces from the alcohol group and the control group and then transplanting their gut microbiota into normal mice by gavage to establish a mouse model of alcohol-FMT and a mouse model of control-FMT. Analysis of the gut microbiota and sperm of these two groups reproduced the results from the alcohol group, such as changes in the gut microbiota and impaired sperm quality, suggesting that the gut microbiota probably mediates the effect of chronic drinking on sperm quality. Interestingly, *Lachnospiraceae*_NK4A136_group, a dominant genus of Firmicutes, and *Helicobacter*, a dominant genus of Bacteroides, exhibited similar trends in both the alcohol-FMT and alcohol groups. In addition, the correlation analysis of sperm quality with the relative abundance of *Lachnospiraceae*_NK4A136_group and *Helicobacter* demonstrated strong correlations between decreased sperm motility and these two genera. Even if the *Lachnospiraceae*_NK4A136_group exerts a direct effect on sperm quality, this result should be considered in the context of chronic drinking-induced alterations in the intestinal ecosystem. Other microbial species may also interact dynamically with these bacteria. Thus, chronic drinking-induced intestinal dysbiosis, particularly alterations in Firmicutes and Bacteroides (with *Lachnospiraceae*_NK4A136_group and *Helicobacter* representing some of the potential ‘culprits’) may result in impaired sperm quality. KEGG pathway analysis revealed a series of pathways with differential enrichment, such as steroid biosynthesis, Wnt signaling pathway, primary bile acid biosynthesis, secondary bile acid biosynthesis, folate biosynthesis, and oxidative phosphorylation, all of which are all related to sperm quality. We performed a nontargeted metabolomics analysis of intestinal contents to further investigate how intestinal dysbiosis may affect sperm quality.

Nontargeted metabolomics of intestinal contents identified significantly differentially altered metabolites between the alcohol-FMT group and the control-FMT group. In particular, the oxidative phosphorylation pathway, a pathway closely related to spermatogenesis and sperm quality, overlapped with the 16S sequencing KEGG pathway analysis. The correlation analysis of 16S rDNA sequencing data and nontargeted metabolomics data indicated that changes in the gut microbiota caused extensive changes in metabolites. Interestingly, most of the differentially altered metabolites were lipids and amino acids, and the bile acid metabolic pathway was significantly less enriched in the alcohol-FMT group. These differentially altered metabolites and metabolic pathways may be one cause of differences in sperm quality in mice, although further research is needed to verify the results.

Extensive studies conducted over the past few decades have demonstrated that the gut microbiota plays an important role in the metabolism and circulation of nitrogen-containing compounds (including amino acids). The microbiota actively metabolizes a large amount of dietary amino acids, which enter the portal vein for systemic use to improve or prevent metabolic syndrome and regulate various signaling molecules involved in sperm quality, oocyte fertilization, and embryo implantation (Bergen and Wu, 2009; Dai et al., 2015). A sufficient amount of circulating amino acids is essential for sperm production, differentiation, and maturation, all of which affect sperm count and sperm quality (Eskiocak et al., 2006; Wu, 2009). As shown in the present study, amino acid metabolites were present at significantly lower levels, and amino acid-related anabolic pathways were significantly less enriched in the alcohol-FMT group than in the control-FMT group, suggesting that chronic drinking affects the metabolites produced by the gut microbiota. In particular, reduced amino acid synthesis affects male reproduction. Moreover, the bile acid metabolic pathways were less enriched in the alcohol-FMT group. Studies have reported that low bile acid levels affect the intestinal absorption of vitamin A, while a disorder in vitamin A metabolism affects testicular cells through the blood circulation, resulting in sperm abnormalities (Zhang et al., 2021). In summary, chronic drinking-induced intestinal dysbiosis leads to changes in gut metabolites, especially reduced amino acid and bile acid synthesis, which may be an important cause of impaired sperm quality.

Chronic drinking-induced intestinal dysbiosis triggers host immune responses (Hedger, 2011a; Atarashi et al., 2015; Rizzetto et al., 2018; Tanoue et al., 2019), including chronic inflammation (Wang et al., 2015). A study revealed that dysbiosis of the gut microbiota induced by a high-fat diet was one of the primary causes of impaired sperm production and motility. This phenotype is likely mediated by elevated blood endotoxin levels, epididymal inflammation, and dysregulation of testicular gene expressions (Ding et al., 2020). In the present study, after FMT from the alcohol group to the control group, serum endotoxin and inflammatory cytokine levels were significantly higher in the alcohol-FMT group than in the control-FMT group. Chronic alcohol consumption may result in elevated serum endotoxin levels (Slevin et al., 2020), which may be mediated by the gut microbiota, as studies have documented a positive correlation between intestinal dysbiosis and elevated levels of circulating endotoxins (Ramezani and Raj, 2014). Endotoxin exerts its effects through Toll-like receptor 4 (TLR4) and myeloid differentiation factor 88 (MyD88)-dependent pathways (Płóciennikowska et al., 2015; Ding et al., 2020). Elevated endotoxin levels induce strong systemic inflammatory responses and reduce testicular and serum androgen (testosterone) levels, thereby causing serious damage to spermatocytes and male reproduction (Gerdprasert et al., 2002; Liew et al., 2007; Zhan et al., 2013). In this study, we observed apparent infiltration/aggregation of T cells and macrophages in the lamina propria of the small intestine in the alcohol-FMT group, indicating strong local inflammatory responses, although further research is needed to confirm the association between local intestinal inflammation and impaired sperm quality. Furthermore, immunohistochemistry of testicular cells showed significant macrophage infiltration and elevated IL-2 and IL-1b levels in the testes and elevated IL-12p70, IL-22, and IL-17 levels in the serum of the alcohol-FMT group, indicating that intestinal microbiota dysbiosis may drive an inflammatory storm in the body, which induces a testicular inflammatory response and oxidative stress (Liu J. B. et al., 2022). which may explain the low sperm count and motility in the alcohol-FMT group. Several studies have found that testicular inflammation exerts a negative effect on sperm quality (Abu et al., 2008; Cao et al., 2010; Hedger, 2011a,b; Azenabor et al., 2015; Wang et al., 2019; Liu J. B. et al., 2022; Liu R. et al., 2022). Dybiosis of the intestinal microbiota causes elevated lipopolysaccharide (LPS), which increases the permeability of the intestinal barrier, potentially allowing bacterial pathogens to enter the systemic circulation; simultaneously, LPS stimulation upregulates the expression of proinflammatory cytokines and chemokines, which recruit and activate white blood cells (such as macrophages and lymphocytes) and induce inflammation to prevent pathogen infection, which may affect the secretion of hormones in the body (Liu R. et al., 2022). These immune responses play a role in fighting a pathogen in the early stage. However, a sustained or prolonged response may affect the testes and the reproductive tract, resulting in orchitis and epididymitis (Rival et al., 2006), and may even induce apoptosis (Sundaresan et al., 2007, 2008). This study also revealed more severe testicular cell apoptosis and impaired sperm quality in the alcohol-FMT group than in the control-FMT group.

Previous animal and human studies have confirmed that elevated endotoxin levels cause orchitis and epididymitis, resulting in low sperm motility and spermatogenic dysfunction (Abu et al., 2008; Cao et al., 2010; Hedger, 2011a,b; Azenabor et al., 2015; Wang et al., 2019; Liu J. B. et al., 2022). An intraperitoneal injection of LPS increases the expression of proinflammatory cytokines such as IL-1β, IL-6, and chemotactic and angiogenic factor (CXCLi2) in chicken epididymis and testes (Liu R. et al., 2022), as well as IL-18 in mouse testes (Abu et al., 2008). In addition, incubating human sperm with LPS may cause low sperm motility (Sahnoun et al., 2017). Bacterial LPS exposure induces inflammatory responses, followed by testicular damage and male infertility in mice (Fijak et al., 2018). These data are consistent with our hypothesis that FMT from the alcohol group to the control group resulted in elevated serum endotoxin levels, followed by systemic chronic inflammation (especially testicular inflammation) and impaired sperm quality in otherwise normal mice.

Sperm quality in mammals is regulated by many factors, and the expression of spermatogenic cells in the testes plays a key role in sperm quality (Gunawan et al., 2011; Guo et al., 2021). Chronic drinking-induced intestinal dysbiosis affects sperm quality, which manifests as differential expression of testicular genes at the molecular level. Abnormal expression of testis-related genes may be an important cause of impaired sperm motility. RNA-seq revealed significantly decreased expression of the chromosomal structure maintenance protein gene Smc1a, which is involved in the regulation of meiosis; oncogenes Myb and Myb12, which regulate cell growth and differentiation; E2F7 and MMP14, which regulate cell cycle progression; and the spermatocyte-specific regulatory factor Tbp in the alcohol-FMT group. Similarly, decreased expression of some mitochondrial genes (such as MT-ND1, MT-ND2, MT-ND3, and MT-ND4), as evidenced by sequencing, may also lead to impaired sperm motility in the alcohol-FMT group. These genes encode NADH dehydrogenase, the core subunit of the mitochondrial membrane respiratory chain, which plays an important role in oxidative phosphorylation and sperm motility (DiMauro and Schon, 2003; Chai et al., 2017). In summary, the abnormal expression of testis-related genes caused by chronic drinking-induced intestinal dysbiosis may lead to spermatogenic dysfunction and impaired sperm quality.

In this study, we performed FMT directly without antibiotic pretreatment. Numerous studies have indicated that the use of antibiotics may cause intestinal dysbiosis in humans (Becattini et al., 2016; Hou et al., 2019; Mu and Zhu, 2019). Moreover, antibiotic pretreatment before FMT may lead to glucose intolerance (Lai et al., 2018) and induce steroidogenesis disturbances *via* mitochondrial dysfunction in Leydig cells, decreasing testosterone levels and ultimately affecting sperm quality (Hou et al., 2019). These metabolic side effects may contribute to spermatogenesis defects (Ding et al., 2020) and can complicate the interpretation of the effect of the gut microbiota on sperm (Lai et al., 2018; Ding et al., 2020). In the present study, the intestinal bacteria used for FMT were prepared from feces, not intestinal contents. Some differences in the transplantation process and colonization efficiency exist between these two approaches (Zmora et al., 2018). We increased the frequency of transplantation (Monday, Wednesday, and Friday of each week, for a total of 10 weeks) to improve intestinal colonization with donor intestinal bacteria. Despite the difference in colonization efficiency between the fecal microbiota and intestinal mucosal microbiota, the transplanted fecal microbiota still exerted a negative effect on spermatogenesis and sperm quality in recipient mice in the alcohol-FMT group. Further research is needed to investigate the effect of the intestinal mucosal microbiota on spermatogenesis and sperm quality.

For many years, alcohol has been considered an important factor contributing to low sperm counts and motility in males. Many researchers have been investigating and achieving considerable progress in determining the mechanism by which alcohol affects sperm quality; however, most studies focus on the direct effect of alcohol on sperm. We focused on a new perspective and confirmed the association between alcohol-induced intestinal dysbiosis and impaired sperm quality. As shown in the present study, chronic drinking causes intestinal dysbiosis that in turn alters gut metabolites, and the effect is transferred to the testes through the blood circulation, resulting in testicular inflammation, abnormal expression of testis-related genes, and ultimately impaired sperm quality in mice. Given the complex interaction between the gut microbiota and host, more research is needed to further investigate how the gut microbiota affects sperm quality. Based on the current data, a potentially useful approach may be to investigate how to treat male infertility by improving the gut microbiota and correcting alcohol-induced impairments in sperm quality.

## Data availability statement

The 16S rRNA raw sequence data reported in this paper have been deposited in the Genome Sequence Archive (Genomics, Proteomics & Bioinformatics 2021) in National Genomics Data Center (Nucleic Acids Res 2022), China National Center for Bioinformation / Beijing Institute of Genomics, Chinese Academy of Sciences (GSA: CRA009014 and CRA009015) that are publicly accessible at https://ngdc.cncb.ac.cn/gsa.

## Ethics statement

The animal study was reviewed and approved by the Laboratory Animal Welfare and Ethics Committee of the Third Military Medical University.

## Author contributions

HL, JZZ, NL, and JZ conceived, designed, and directed the study. HL, QL, JZZ, and QZ collected the mouse samples and sperm measurement data. HL and TL performed the FMT experiments. HL and JZZ obtained and analyzed the microbiota data. HL, JZZ, and NL interpreted the results and drafted the manuscript. HL, WC, JY, SY, WF, YZ, GY, JT, JX, and ZL reviewed and edited the manuscript. All authors contributed to the article and approved the submitted version.

## Funding

This study was supported by grants from the National Natural Science Foundation of China (nos. 81900690 and 82102077) and the Natural Science Foundation of Chongqing (nos. cstc2020jcyj-msxmX0065 and cstc2021jcyj-msxmX0056).

## Conflict of interest

The authors declare that the research was conducted in the absence of any commercial or financial relationships that could be construed as a potential conflict of interest.

## Publisher’s note

All claims expressed in this article are solely those of the authors and do not necessarily represent those of their affiliated organizations, or those of the publisher, the editors and the reviewers. Any product that may be evaluated in this article, or claim that may be made by its manufacturer, is not guaranteed or endorsed by the publisher.

## Footnotes

^1^ http://www.R-project.org/

^2^ http://huttenhower.sph.harvard.edu/lefse/

^3^ https://www.mzcloud.org/

^4^ https://www.genome.jp/kegg/pathway.html

^5^ https://hmdb.ca/metabolites

^6^ http://www.lipidmaps.org/
