# Supplementary material for: Chronic alcohol-induced dysbiosis of the gut microbiota and gut metabolites impairs sperm quality in mice
Source: Front Microbiol. 2022 Dec 1;13:1042923. doi: 10.3389/fmicb.2022.1042923 (PMC9751024; doi:10.3389/fmicb.2022.1042923)
Supplement: Supplementary file 1 [file Data_Sheet_1.ZIP › supplemental/Supplemental Table 2.docx]

**Supplemental Table 2 Effect of Alcohol on intestinal mucosa**

| Group | Sample ID | Intercellular distance of intestinal mucosa（nm）  Mean±SD | | | P-value |
| --- | --- | --- | --- | --- | --- |
| Control | C1-01 | 29.70297±8.04 | 18.66949±12.36 | 23.10231±9.16 | ˂0.001 |
|  | C1-02 | 19.32978±13.45 | 17.234426±9.14 | 12.862596±4.68 |  |
|  | C1-07 | 15.999976±7.82 | 16.901354±3.99 | 18.77248±12.26 |  |
| Alcohol | A2-04 | 39.370484±20.24 | 32.87218±14.85 | 32.597468±16.27 | ˂0.001 |
|  | A2-05 | 65.527514±37.77 | 65.527514±15.45 | 77.975698±36.67 |  |
|  | A2-06 | 48.494536±40.90 | 50.367224±52.99 | 105.274756±112.18 |  |

Difference between Control and Alcohol group.
