# Supplementary figures and images for: Chronic alcohol-induced dysbiosis of the gut microbiota and gut metabolites impairs sperm quality in mice
Source: Front Microbiol. 2022 Dec 1;13:1042923. doi: 10.3389/fmicb.2022.1042923 (PMC9751024; doi:10.3389/fmicb.2022.1042923)

**alpha-diversity measure**

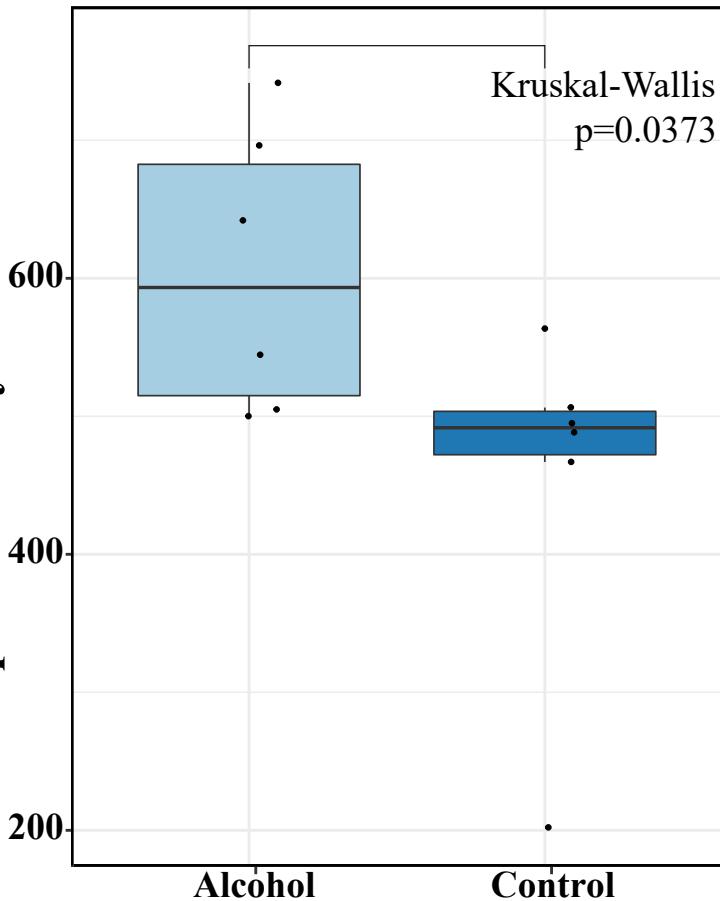

Supplement: Supplementary file 1 [file Data_Sheet_1.ZIP › supplemental/supplemental Figure. S1a ace.pdf]

**alpha-diversity measure**

200

400

600

**Alcohol**

**Control**

Kruskal-Wallis  
 $p=0.0373$

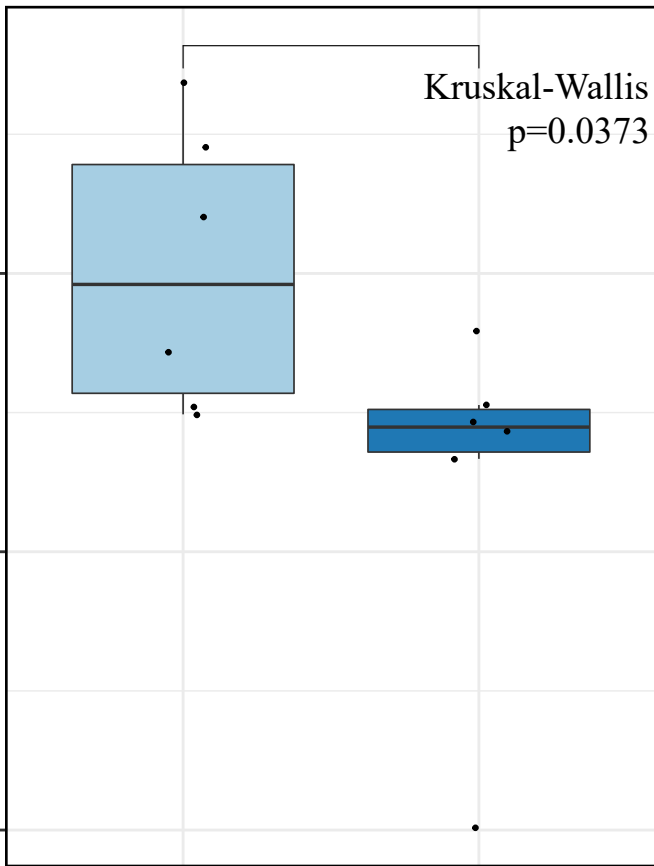

Supplement: Supplementary file 1 [file Data_Sheet_1.ZIP › supplemental/supplemental Figure. S1a chao1.pdf]

**alpha-diversity measure**

**Kruskal-Wallis**  
**p=0.0249**

75

50

25

**Alcohol**

**Control**

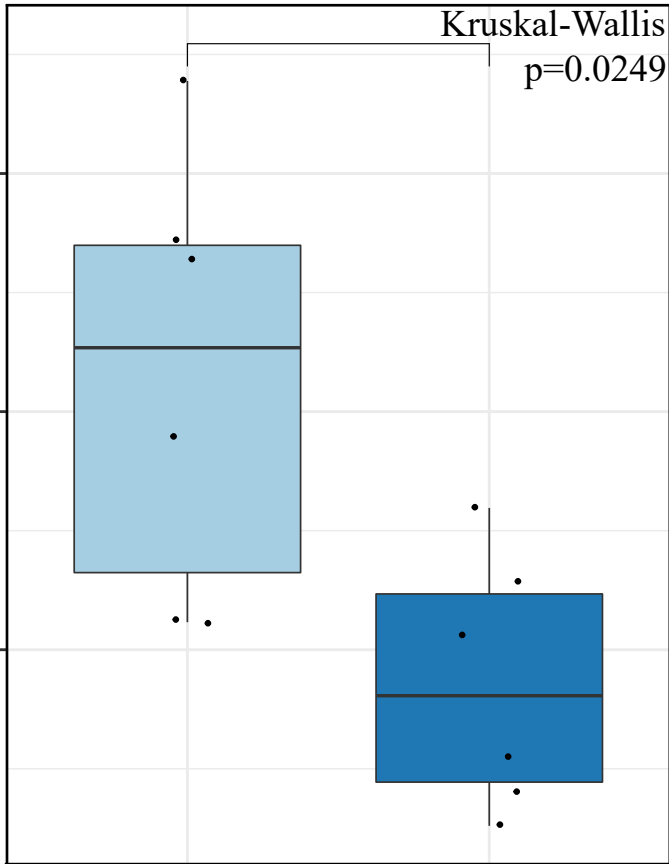

Supplement: Supplementary file 1 [file Data_Sheet_1.ZIP › supplemental/supplemental Figure. S1a enspie.pdf]

# fisher\_alpha

alpha-diversity measure

Kruskal-Wallis  
p=0.0373

Alcohol

Control

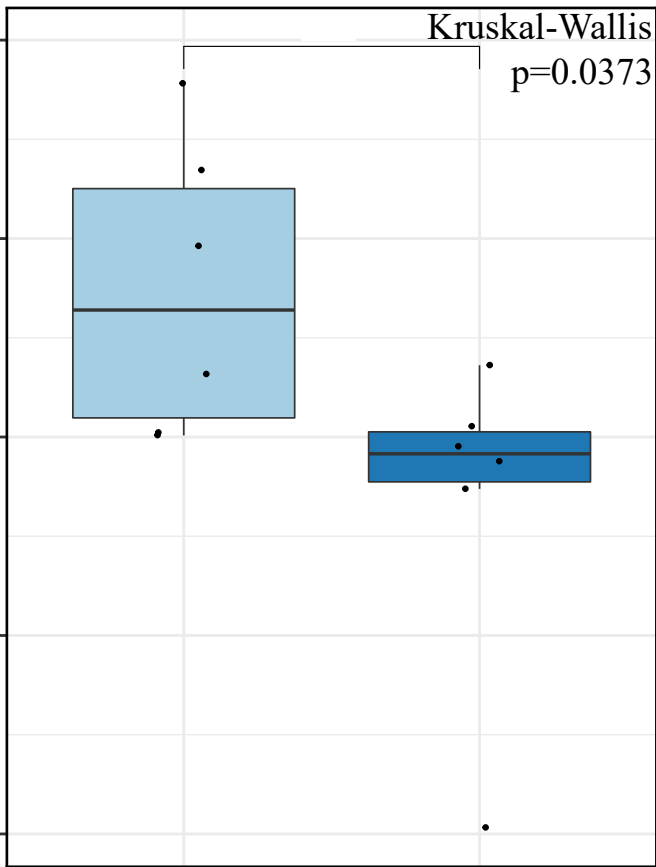

Supplement: Supplementary file 1 [file Data_Sheet_1.ZIP › supplemental/supplemental Figure. S1a fisher_alpha.pdf]

**alpha-diversity measure**

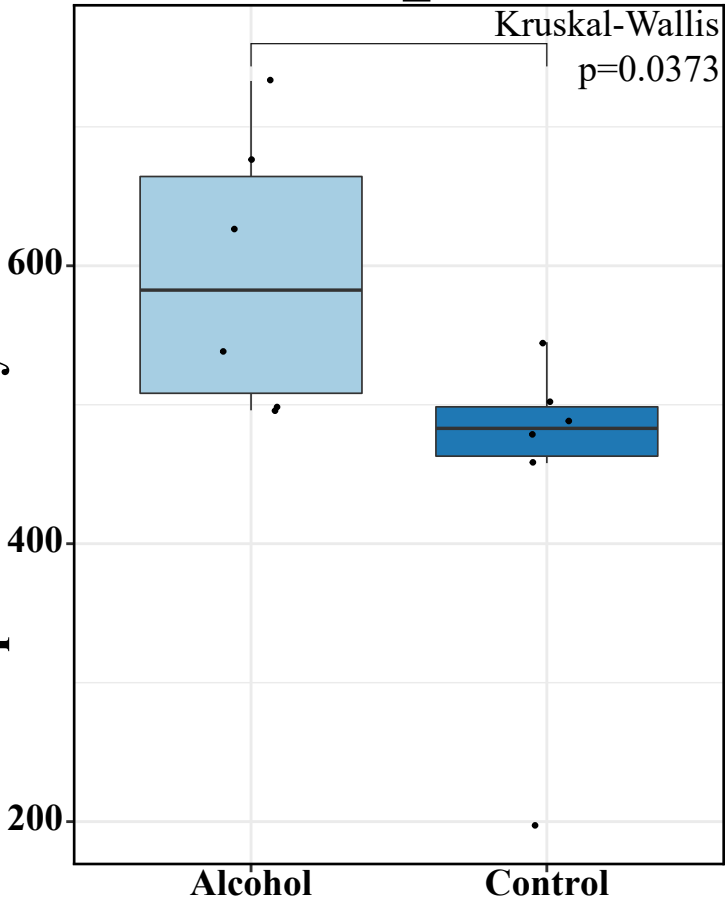

Supplement: Supplementary file 1 [file Data_Sheet_1.ZIP › supplemental/supplemental Figure. S1a observed_features.pdf]

**alpha-diversity measure**

**Kruskal-Wallis**  
**p=0.0249**

1.00

0.95

0.90

0.85

**Alcohol**

**Control**

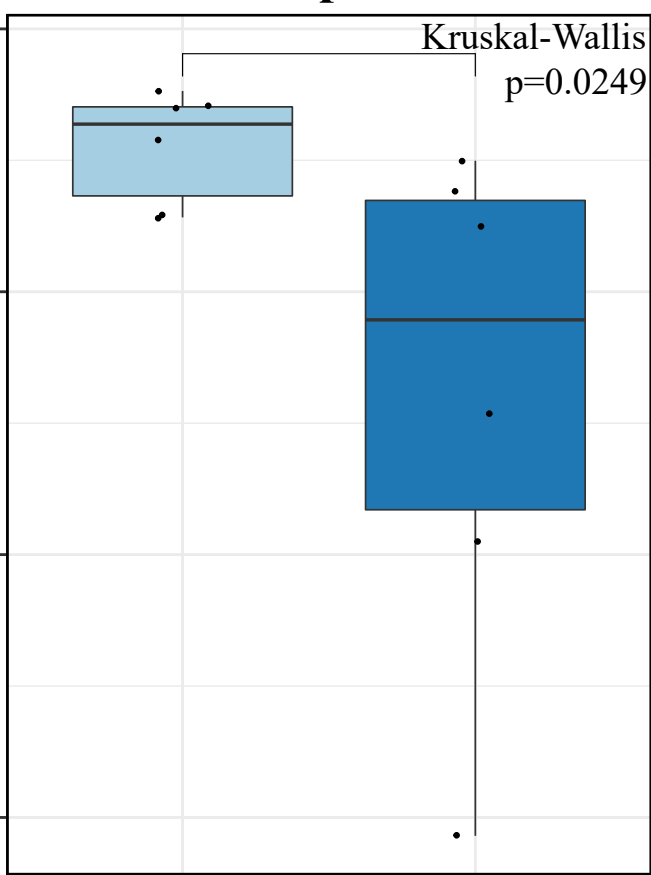

Supplement: Supplementary file 1 [file Data_Sheet_1.ZIP › supplemental/supplemental Figure. S1a simpson.pdf]

# The bacterial composition in Class

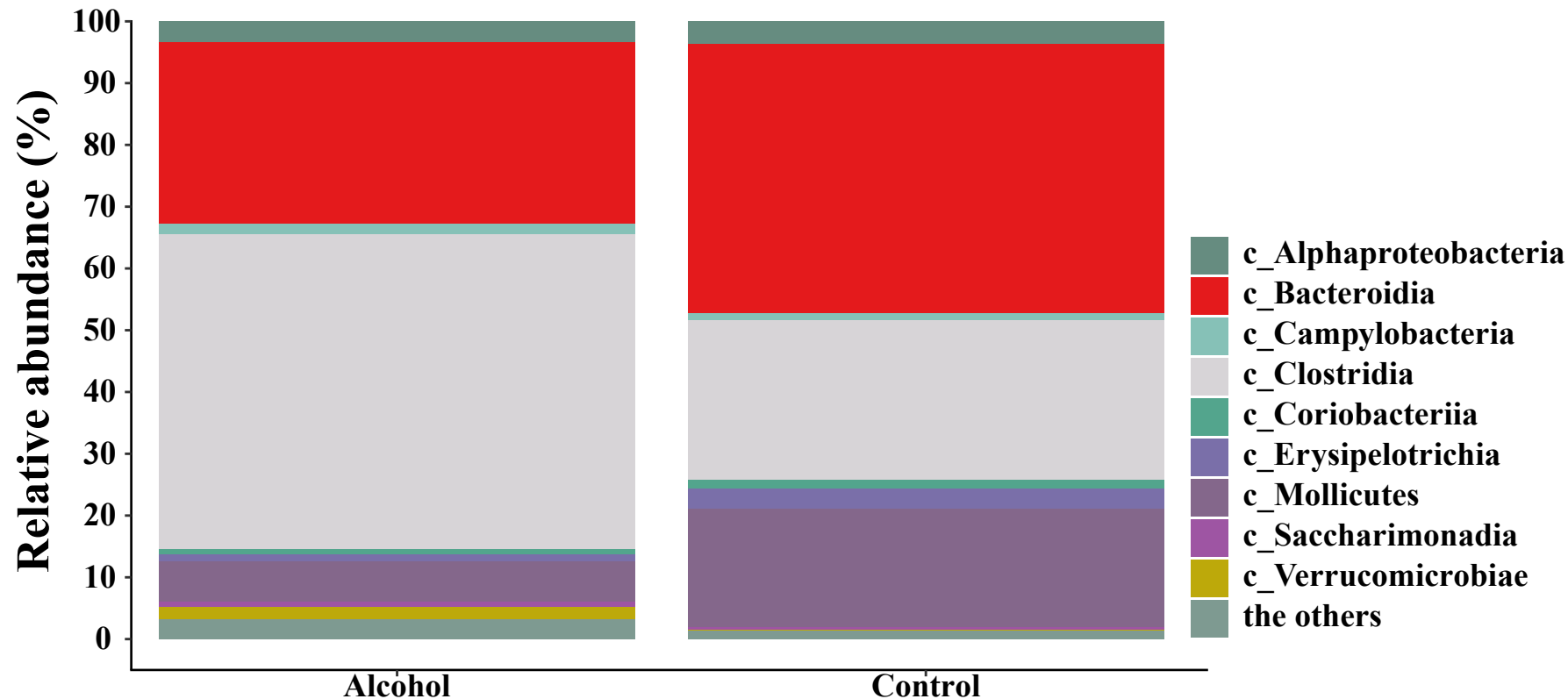

Supplement: Supplementary file 1 [file Data_Sheet_1.ZIP › supplemental/supplemental Figure. S1b Class.pdf]

# The bacterial composition in Family

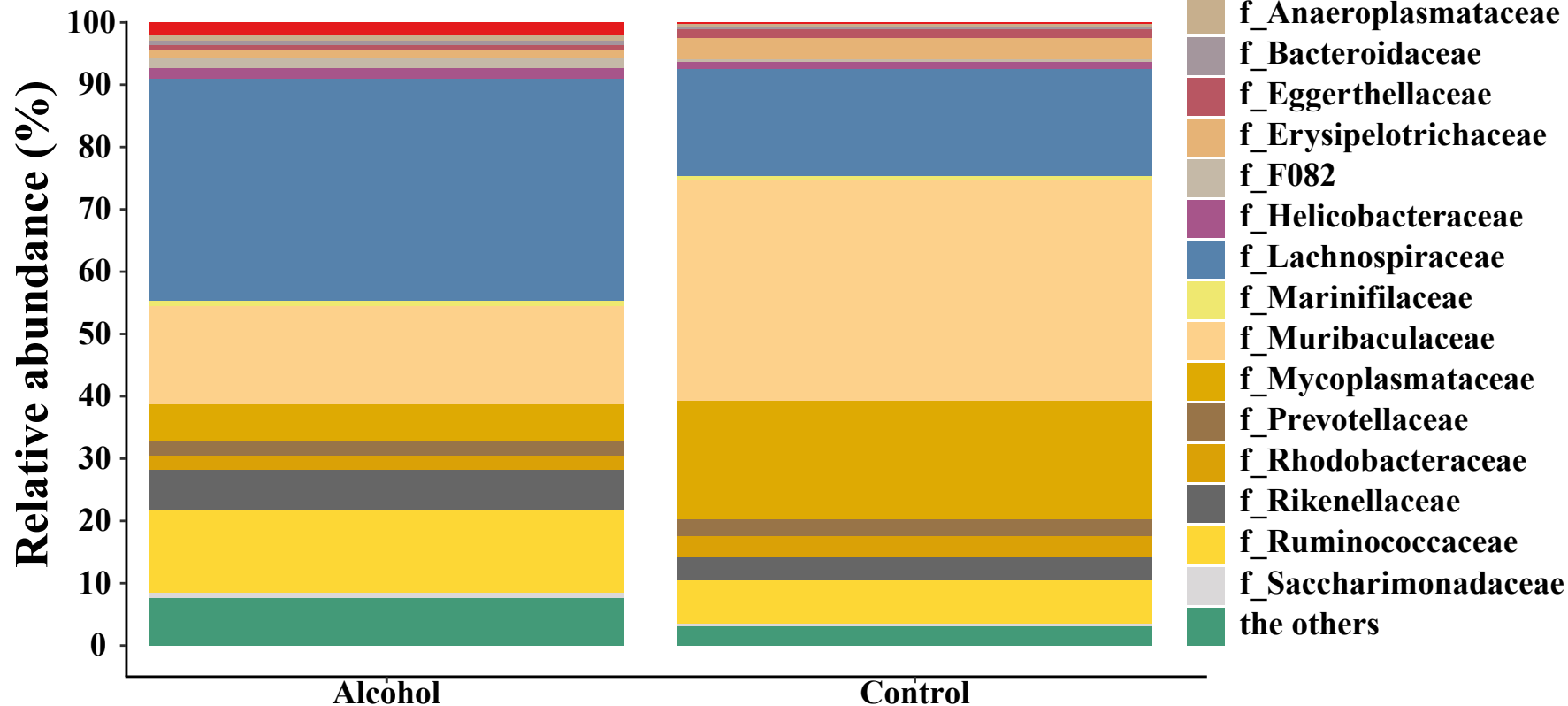

Supplement: Supplementary file 1 [file Data_Sheet_1.ZIP › supplemental/supplemental Figure. S1b Family.pdf]

# The bacterial composition in Order

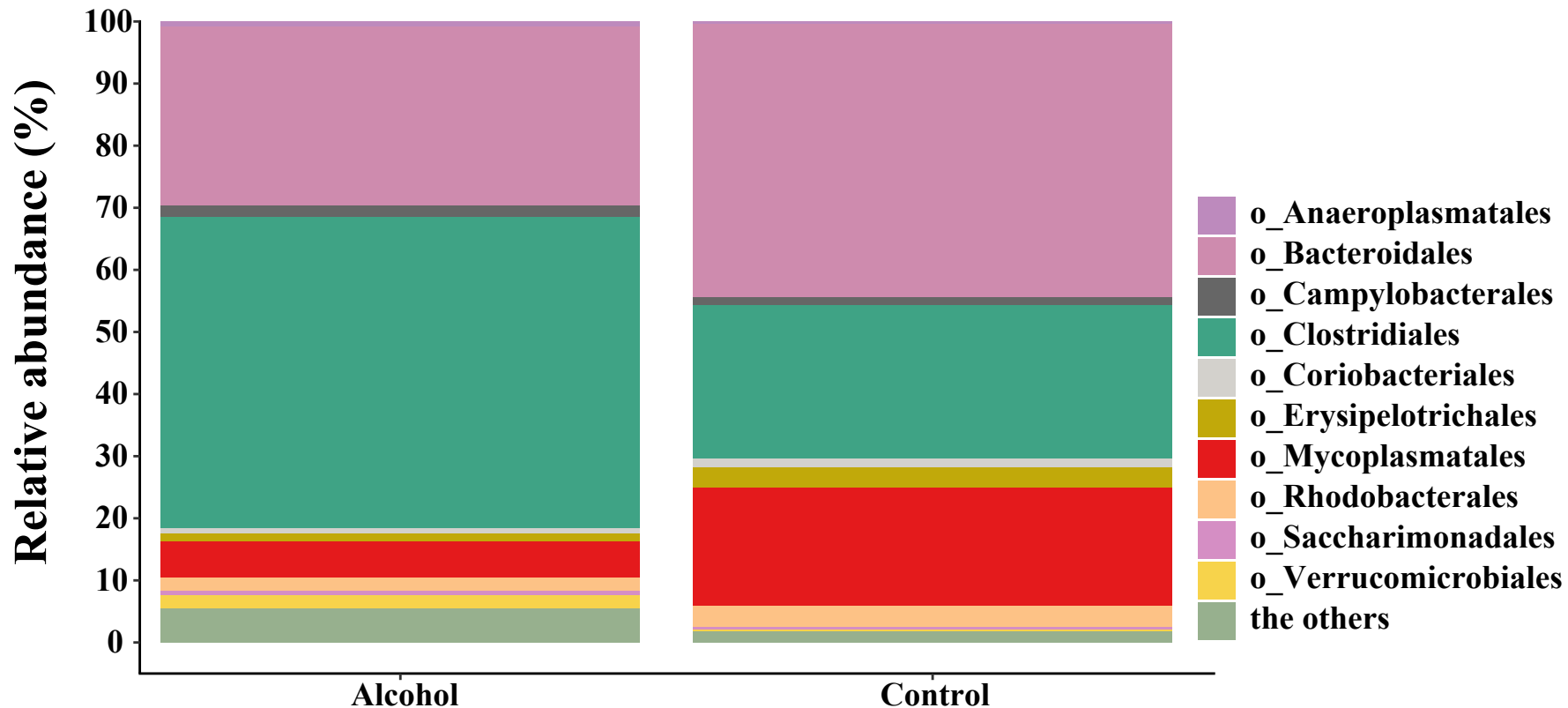

Supplement: Supplementary file 1 [file Data_Sheet_1.ZIP › supplemental/supplemental Figure. S1b Order.pdf]

# The bacterial composition in Phylum

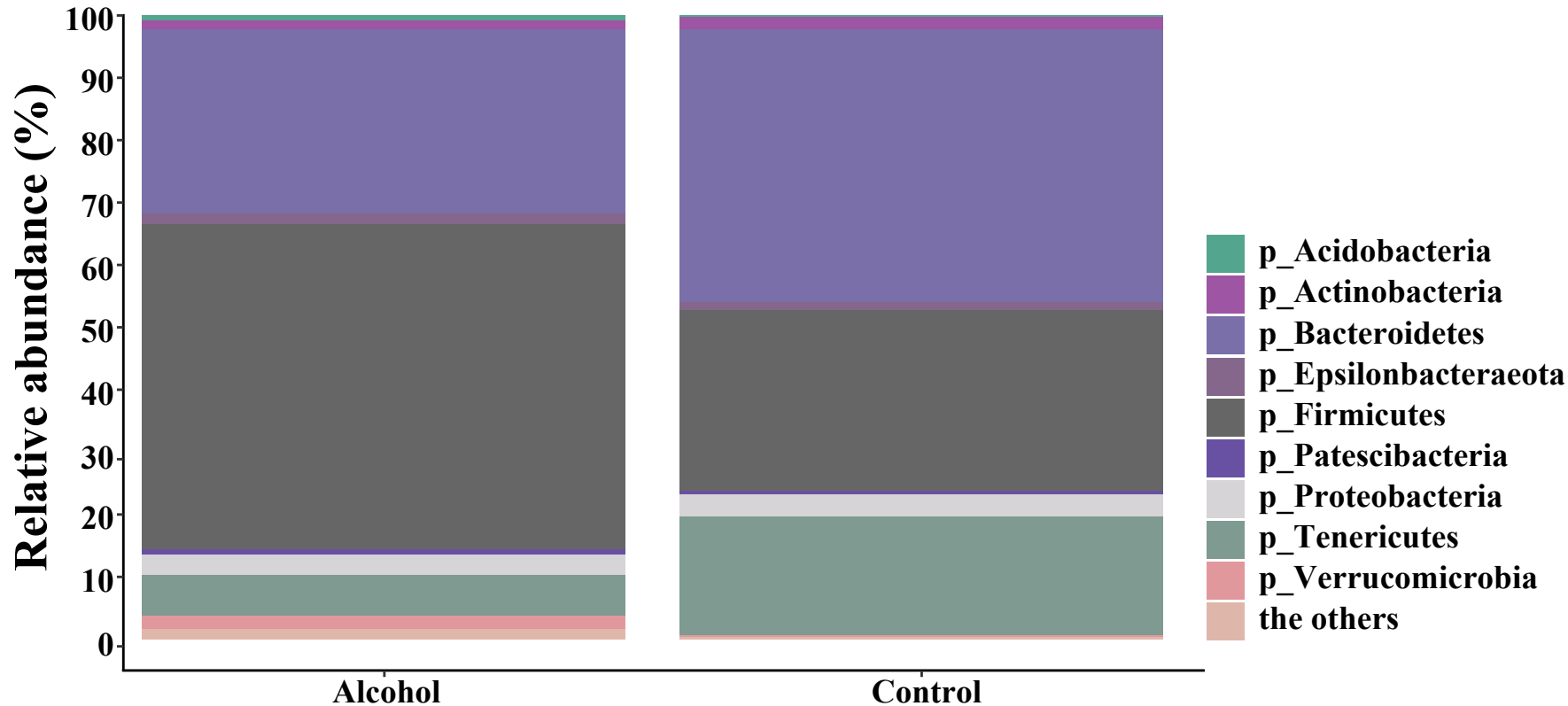

Supplement: Supplementary file 1 [file Data_Sheet_1.ZIP › supplemental/supplemental Figure. S1b Phylum.pdf]

# ACE

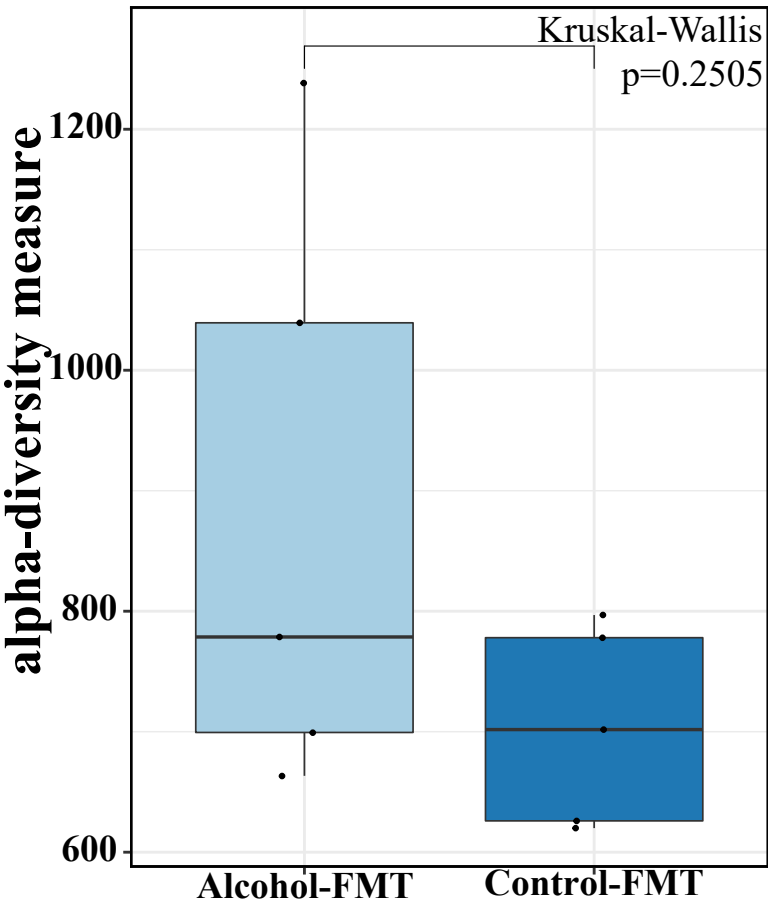

Supplement: Supplementary file 1 [file Data_Sheet_1.ZIP › supplemental/supplemental Figure.S3a ace.pdf]

# chao1

alpha-diversity measure

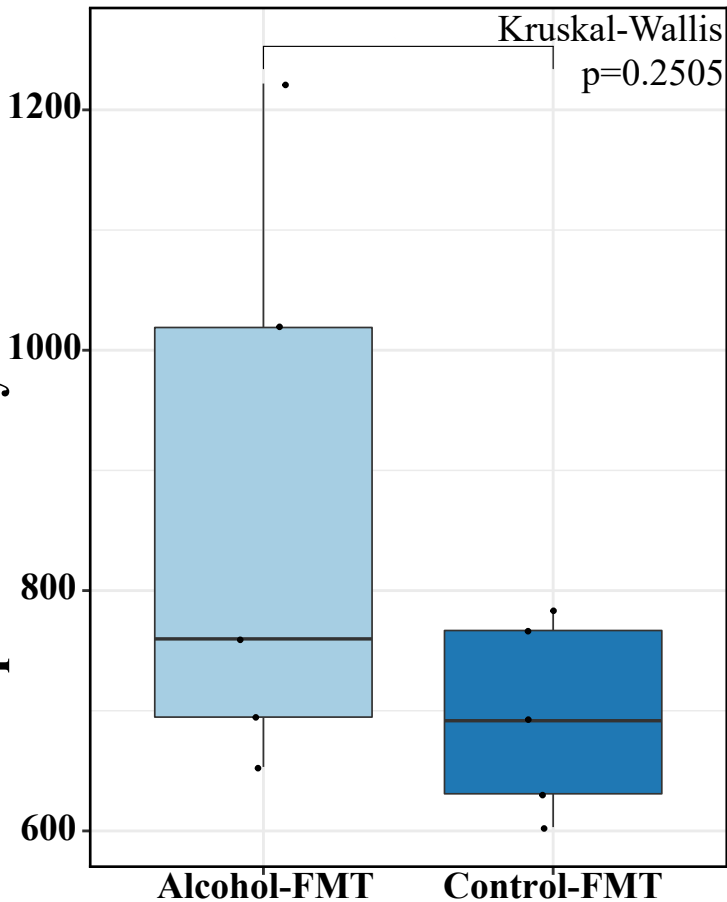

Supplement: Supplementary file 1 [file Data_Sheet_1.ZIP › supplemental/supplemental Figure.S3a chao1.pdf]

# enspie

alpha-diversity measure

Kruskal-Wallis  
p=0.4647

100

75

50

25

Alcohol-FMT

Control-FMT

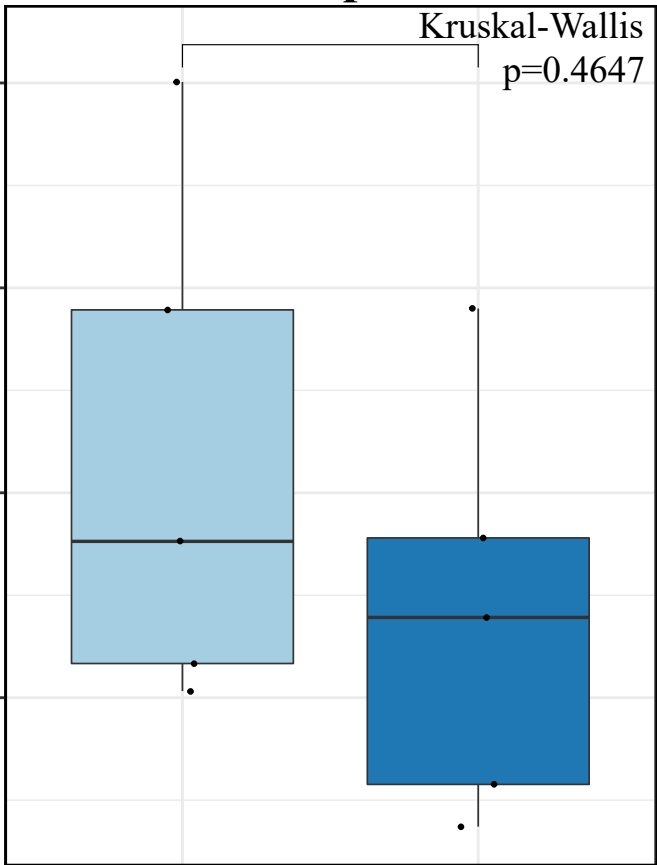

Supplement: Supplementary file 1 [file Data_Sheet_1.ZIP › supplemental/supplemental Figure.S3a enspie.pdf]

# fisher\_alpha

alpha-diversity measure

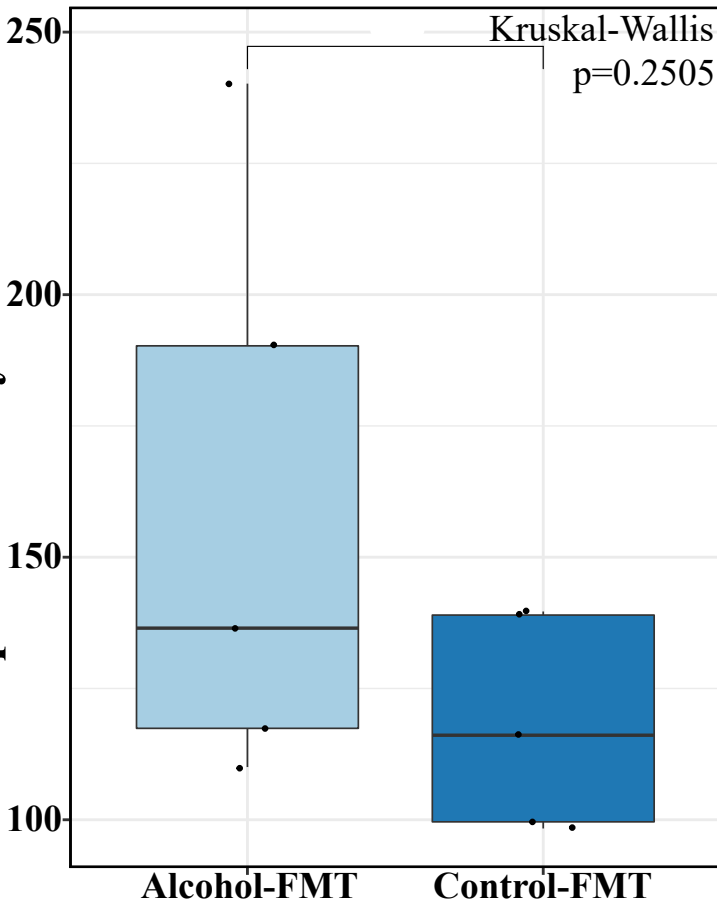

Supplement: Supplementary file 1 [file Data_Sheet_1.ZIP › supplemental/supplemental Figure.S3a fisher_alpha.pdf]

# observed\_features

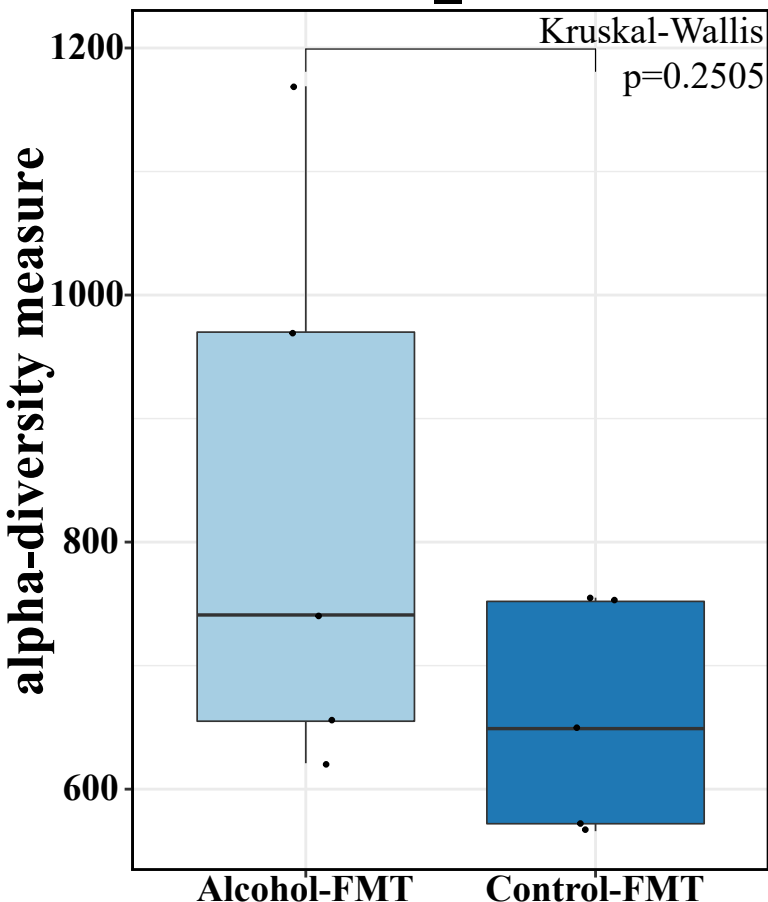

Supplement: Supplementary file 1 [file Data_Sheet_1.ZIP › supplemental/supplemental Figure.S3a observed_features.pdf]

# simpson

alpha-diversity measure

1.000

0.975

0.950

0.925

0.900

Kruskal-Wallis

p=0.4647

Alcohol-FMT

Control-FMT

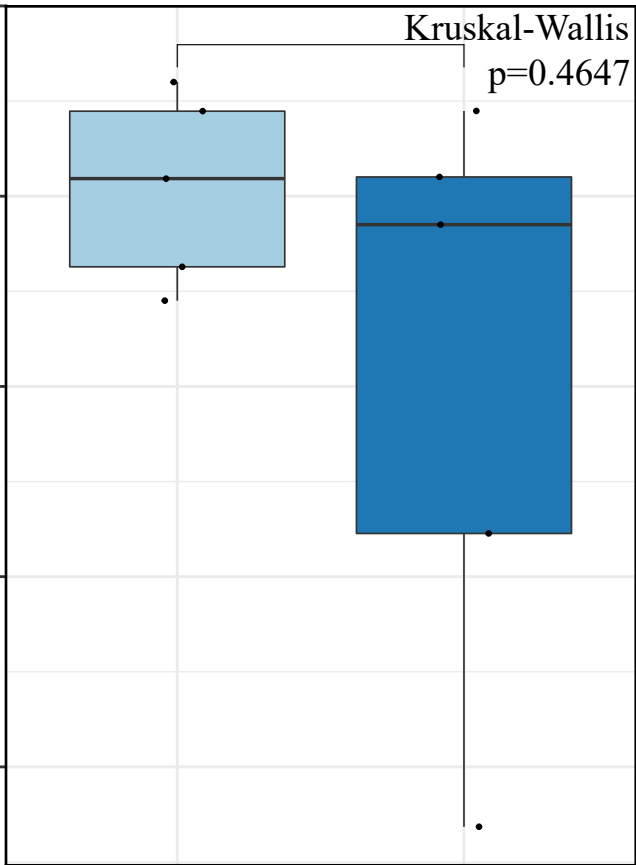

Supplement: Supplementary file 1 [file Data_Sheet_1.ZIP › supplemental/supplemental Figure.S3a simpson.pdf]

# The bacterial composition in Class

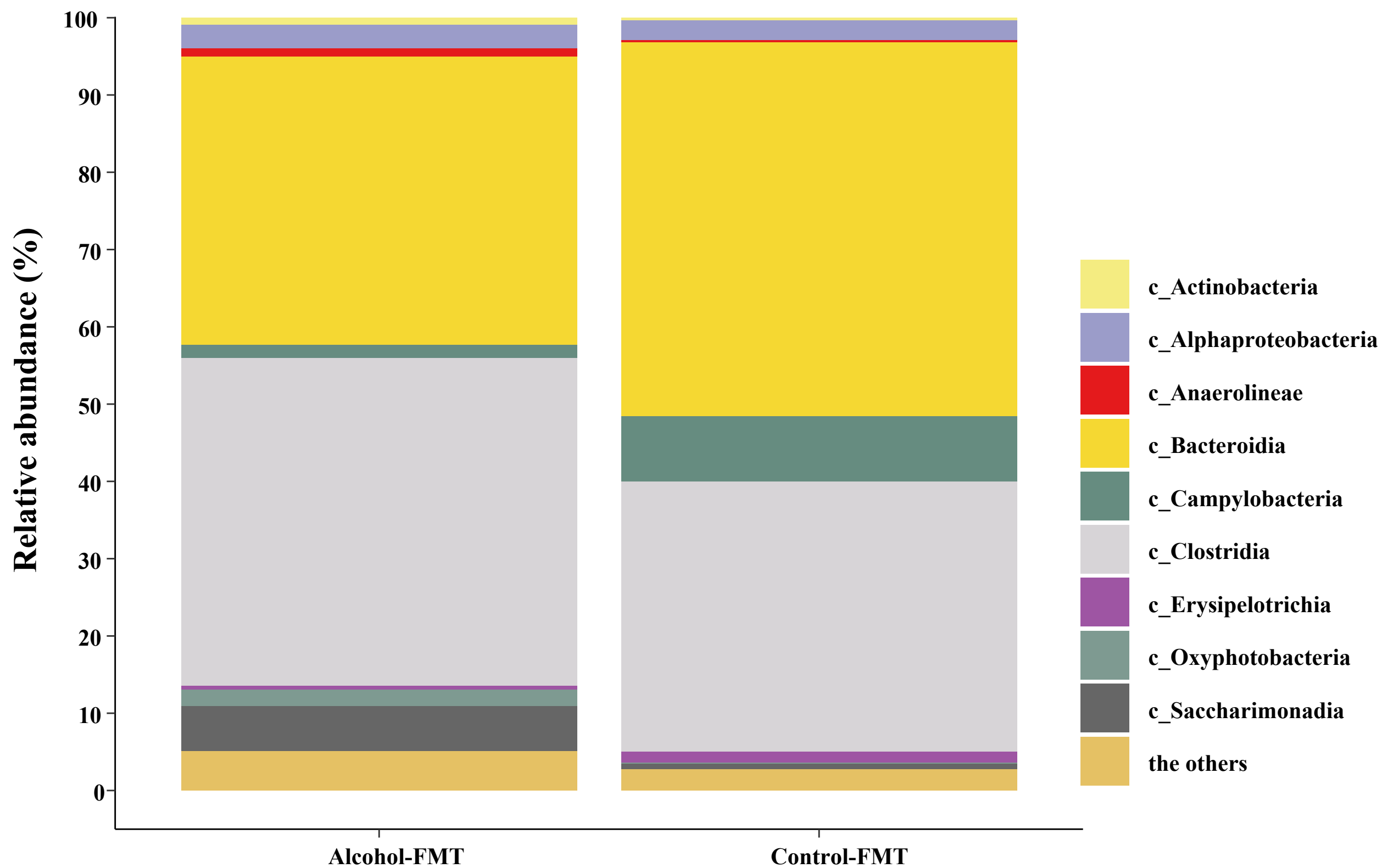

Supplement: Supplementary file 1 [file Data_Sheet_1.ZIP › supplemental/supplemental Figure.S3b Class.pdf]

The bacterial composition in Family

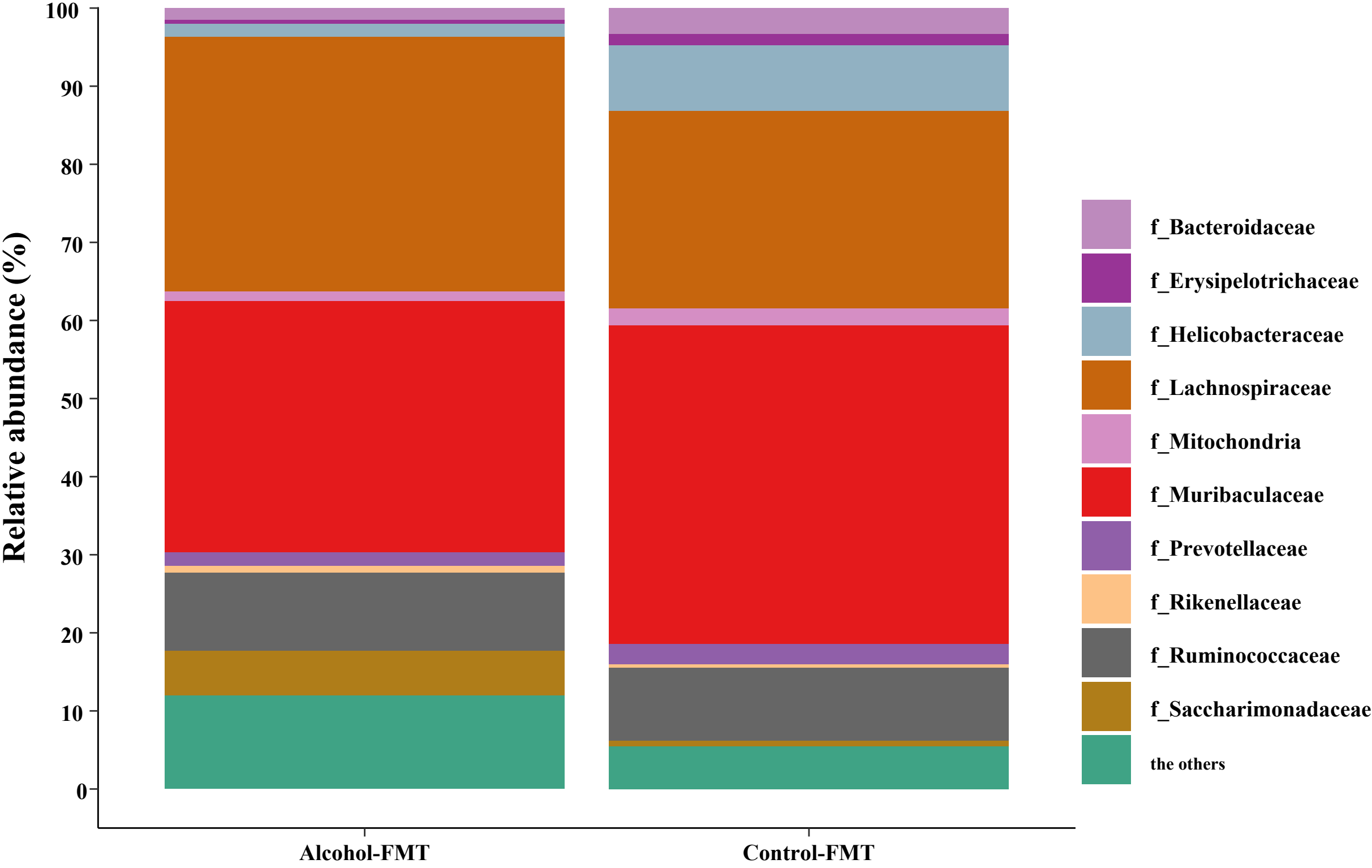

Supplement: Supplementary file 1 [file Data_Sheet_1.ZIP › supplemental/supplemental Figure.S3b Family.pdf]

**The bacterial composition in Order**

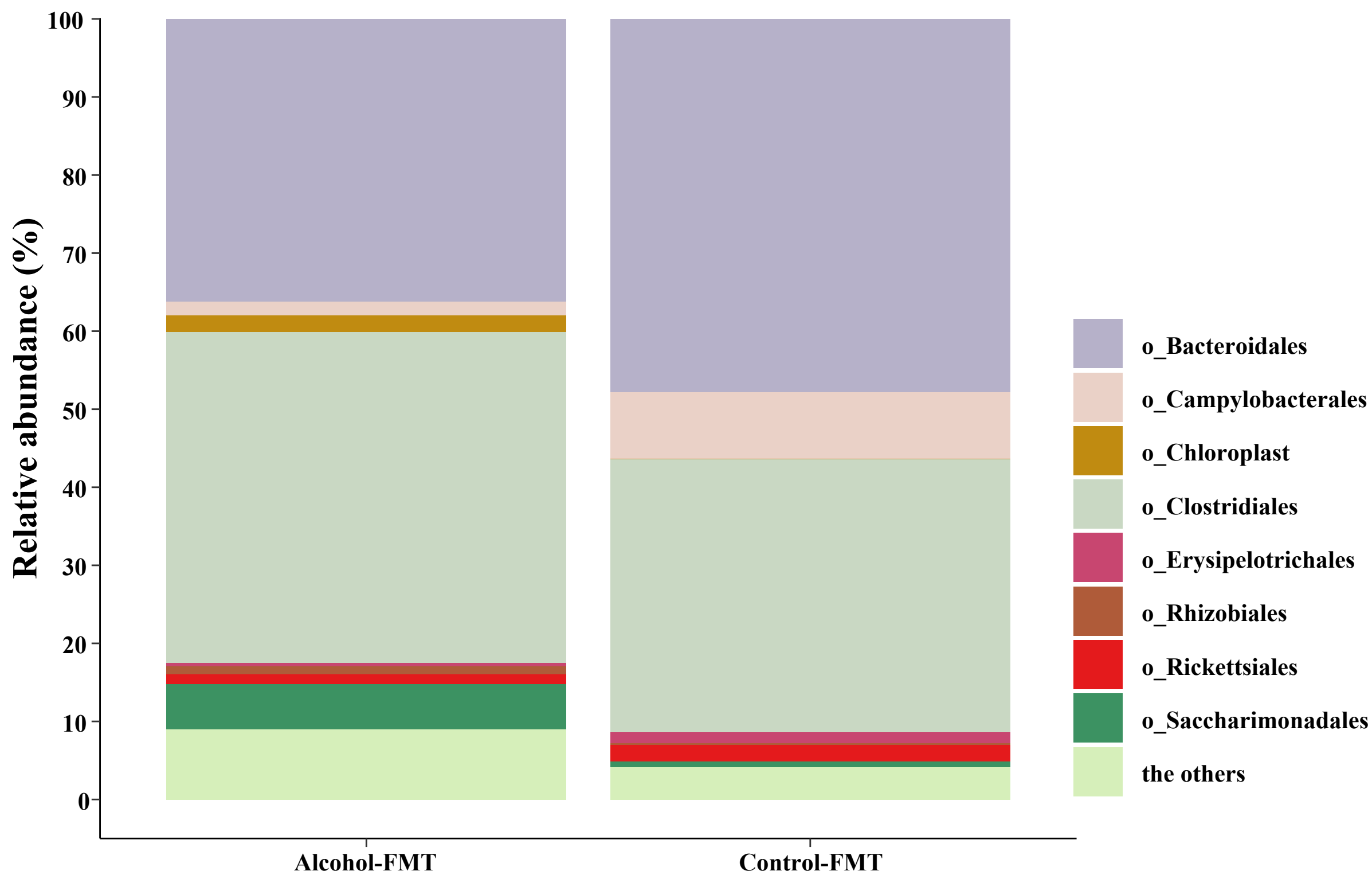

Supplement: Supplementary file 1 [file Data_Sheet_1.ZIP › supplemental/supplemental Figure.S3b Order.pdf]

**The bacterial composition in Phylum**

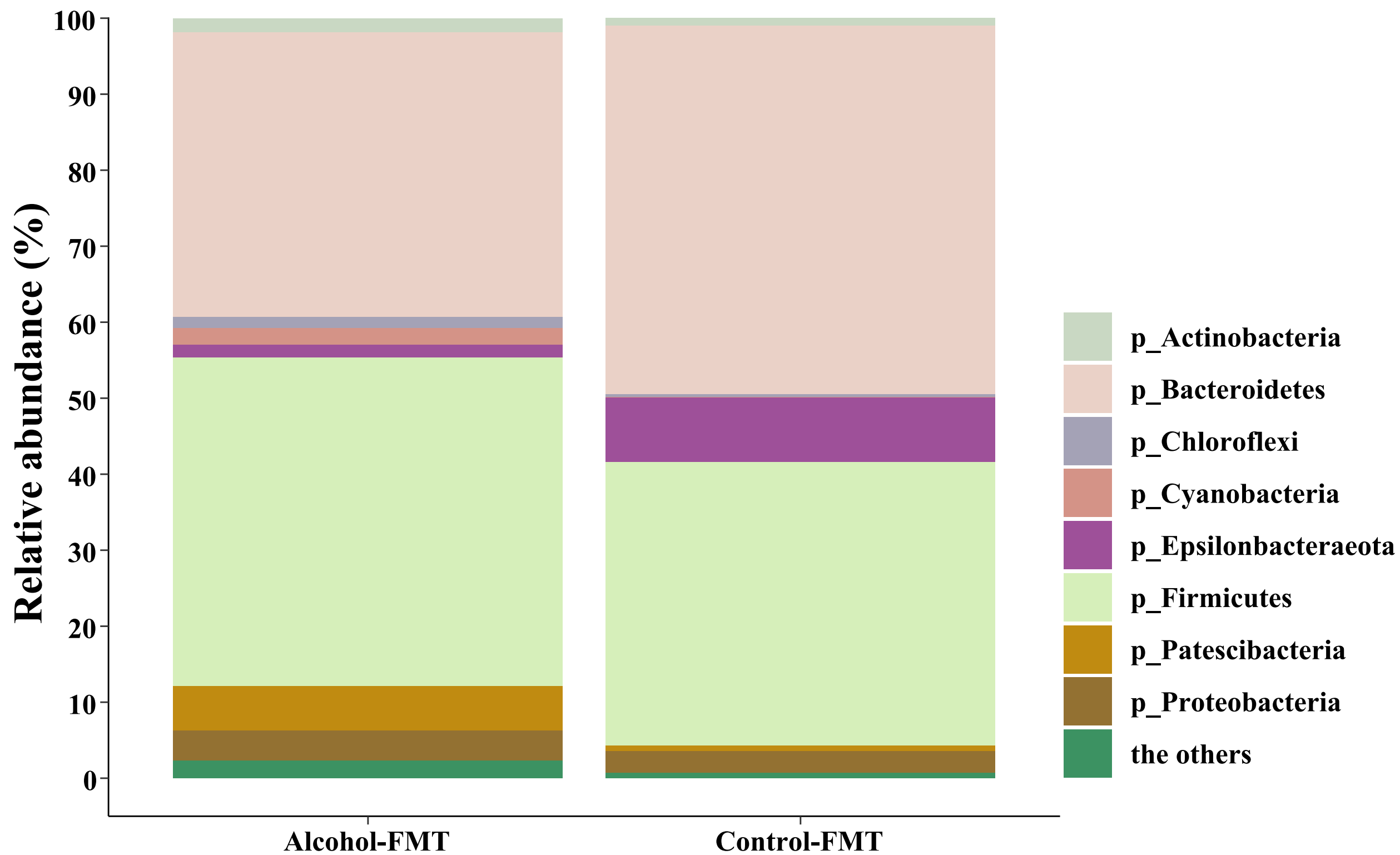

Supplement: Supplementary file 1 [file Data_Sheet_1.ZIP › supplemental/supplemental Figure.S3b Phylum.pdf]
