# Supplementary material for: Chronic alcohol-induced dysbiosis of the gut microbiota and gut metabolites impairs sperm quality in mice
Source: Front Microbiol. 2022 Dec 1;13:1042923. doi: 10.3389/fmicb.2022.1042923 (PMC9751024; doi:10.3389/fmicb.2022.1042923)
Supplement: Supplementary file 2 [file Data_Sheet_2.ZIP › supplemental/Supplemental Table 1.docx]

**Supplemental Table 1 Data on** **Average weekly 10% alcohol intake of Alcohol Group**

| **Group** | **Sample**  **ID** | **weekly 10% alcohol intake** (ml/week) | | | | | | | | | | **Total 10% alcohol intake** (ml) | **Average** **weekly 10% alcohol intake** (ml) | **Average weekly 10% alcohol intake / per mice** (ml) |
| --- | --- | --- | --- | --- | --- | --- | --- | --- | --- | --- | --- | --- | --- | --- |
|  |  | 1w | 2w | 3w | 4w | 5w | 6w | 7w | 8w | 9w | 10w |  |  |  |
| **Alcohol** | A1-01 | 245.15 | 177.43 | 327.58 | 324.41 | 261.90 | 429.87 | 344.12 | 265.42 | 296.83 | 232.18 | 2904.89 | 290.49 | 24.21 |
|  | A1-02 |  |  |  |  |  |  |  |  |  |  |  |  |  |
|  | A1-03 |  |  |  |  |  |  |  |  |  |  |  |  |  |
|  | A1-04 |  |  |  |  |  |  |  |  |  |  |  |  |  |
|  | A1-05 |  |  |  |  |  |  |  |  |  |  |  |  |  |
|  | A1-06 |  |  |  |  |  |  |  |  |  |  |  |  |  |
|  | A2-01 |  |  |  |  |  |  |  |  |  |  |  |  |  |
|  | A2-02 |  |  |  |  |  |  |  |  |  |  |  |  |  |
|  | A2-03 |  |  |  |  |  |  |  |  |  |  |  |  |  |
|  | A2-04 |  |  |  |  |  |  |  |  |  |  |  |  |  |
|  | A2-05 |  |  |  |  |  |  |  |  |  |  |  |  |  |
|  | A2-06 |  |  |  |  |  |  |  |  |  |  |  |  |  |
