# Supplementary material for: Chronic alcohol-induced dysbiosis of the gut microbiota and gut metabolites impairs sperm quality in mice
Source: Front Microbiol. 2022 Dec 1;13:1042923. doi: 10.3389/fmicb.2022.1042923 (PMC9751024; doi:10.3389/fmicb.2022.1042923)
Supplement: Supplementary file 2 [file Data_Sheet_2.ZIP › supplemental/Supplemental Table 2.docx]

**Supplemental Table 2 Comparison of body weight between Alcohol and Control Groups**

| Group | Sample  ID | Weight of before and after alcohol treatment(g) | | | P-value |
| --- | --- | --- | --- | --- | --- |
|  |  | Pre-Alcohol | Post-Alcohol | Difference |  |
| Alcohol | A1-01 | 15.1 | 26.5 | 11.4 | 0.419 |
|  | A1-02 | 17.7 | 23.7 | 6 |  |
|  | A1-03 | 16.4 | 22 | 5.6 |  |
|  | A1-04 | 15 | 22.5 | 7.5 |  |
|  | A1-05 | 13.4 | 18.6 | 5.2 |  |
|  | A1-06 | 11.9 | 21.6 | 9.7 |  |
|  | A2-01 | 14.4 | 23.6 | 9.2 |  |
|  | A2-02 | 14 | 21.4 | 7.4 |  |
|  | A2-03 | 12.8 | 23.4 | 10.6 |  |
|  | A2-04 | 16.6 | 23.5 | 6.9 |  |
|  | A2-05 | 14.6 | 22.2 | 7.6 |  |
|  | A2-06 | 11.5 | 18.8 | 7.3 |  |
| Control | C1-01 | 17.3 | 27.2 | 9.9 |  |
|  | C1-02 | 14.9 | 24 | 9.1 |  |
|  | C1-03 | 16.7 | 25.2 | 8.5 |  |
|  | C1-05 | 16.5 | 22.5 | 6 |  |
|  | C1-06 | 16.4 | 23.6 | 7.2 |  |
|  | C1-07 | 16.6 | 27.7 | 11.1 |  |
|  | C2-01 | 14.9 | 22.8 | 7.9 |  |
|  | C2-02 | 14.7 | 20.8 | 6.1 |  |
|  | C2-03 | 16.6 | 23.3 | 6.7 |  |
|  | C2-04 | 13.3 | 22.1 | 8.8 |  |
|  | C2-06 | 14 | 23.8 | 9.8 |  |
|  | C2-07 | 13.4 | 24.2 | 10.8 |  |
