# Supplementary material for: Chronic alcohol-induced dysbiosis of the gut microbiota and gut metabolites impairs sperm quality in mice
Source: Front Microbiol. 2022 Dec 1;13:1042923. doi: 10.3389/fmicb.2022.1042923 (PMC9751024; doi:10.3389/fmicb.2022.1042923)
Supplement: Supplementary file 2 [file Data_Sheet_2.ZIP › supplemental/Supplemental Table 4.docx]

**Supplemental Table 4 Comparison of body weight between Alcohol-FMT and Control-FMT Groups**

| Group | Sample  ID | Weight of before and after FMT(g) | | | P-value |
| --- | --- | --- | --- | --- | --- |
|  |  | Pre-FMT | Post-FMT | Difference |  |
| Alcohol-FMT | A18 | 22.2 | 27.7 | 5.5 | 0.479 |
|  | A24 | 18.8 | 22.2 | 3.4 |  |
|  | A27 | 18.4 | 21.4 | 3 |  |
|  | A28 | 16.7 | 18.9 | 2.2 |  |
|  | A31 | 15.3 | 17.7 | 2.4 |  |
| Control-FMT | C16 | 19.4 | 21.6 | 2.2 |  |
|  | C17 | 18.4 | 21.6 | 3.2 |  |
|  | C19 | 24.1 | 27.9 | 3.8 |  |
|  | C20 | 17.6 | 19.7 | 2.1 |  |
|  | C21 | 22.9 | 26.3 | 3.4 |  |
