# Supplementary material for: Chronic alcohol-induced dysbiosis of the gut microbiota and gut metabolites impairs sperm quality in mice
Source: Front Microbiol. 2022 Dec 1;13:1042923. doi: 10.3389/fmicb.2022.1042923 (PMC9751024; doi:10.3389/fmicb.2022.1042923)
Supplement: Supplementary file 2 [file Data_Sheet_2.ZIP › supplemental/Supplemental Table 5.docx]

**Supplemental Table 5** **Data on metabolites relative Quant of Alcohol-FMT and Control-FMT Group**

| **Compound_ID** | **Metabolites Name** | **Group** | | | | | | | | | | **P**  **value** |
| --- | --- | --- | --- | --- | --- | --- | --- | --- | --- | --- | --- | --- |
|  |  | **Alcohol-FMT** | | | | | **Control-FMT** | | | | |  |
|  |  | **A18** | **A24** | **A27** | **A28** | **A31** | **C16** | **C17** | **C19** | **C20** | **C21** |  |
| Com_10000_pos | 2-(Formylamino)Benzoic Acid | 1.66E+07 | 1.42E+07 | 7.92E+06 | 2.34E+07 | 1.49E+07 | 2.80E+07 | 2.24E+07 | 1.63E+07 | 3.22E+07 | 1.79E+07 | 0.08 |
| Com_10015_pos | 4-Ethylbenzaldehyde | 1.28E+07 | 1.11E+07 | 3.78E+07 | 5.49E+06 | 1.19E+07 | 1.08E+07 | 1.50E+07 | 1.12E+07 | 1.38E+07 | 7.14E+06 | 0.70 |
| Com_1003_pos | Thymine | 2.62E+08 | 4.67E+08 | 2.32E+08 | 1.58E+08 | 3.66E+08 | 2.46E+08 | 2.13E+08 | 1.67E+08 | 2.51E+08 | 7.00E+08 | 0.97 |
| Com_10051_pos | 3,14-dihydro-15-keto-tetranor Prostaglandin E2 | 7.05E+06 | 1.49E+07 | 1.86E+07 | 1.12E+07 | 1.19E+07 | 1.55E+06 | 2.79E+07 | 4.60E+06 | 3.19E+07 | 1.77E+07 | 0.79 |
| Com_10055_pos | ACar 10:1 | 4.94E+05 | 1.02E+06 | 3.75E+07 | 6.30E+05 | 6.23E+05 | 3.40E+06 | 7.24E+05 | 7.52E+05 | 6.25E+05 | 6.82E+05 | 0.63 |
| Com_10056_neg | 19-Nortestosterone | 6.43E+06 | 5.63E+06 | 4.30E+06 | 5.49E+06 | 7.28E+06 | 2.07E+06 | 7.86E+06 | 9.10E+06 | 3.90E+06 | 4.35E+06 | 0.55 |
| Com_10059_pos | 5-Methylcytosine | 1.07E+07 | 6.68E+06 | 2.90E+07 | 2.69E+06 | 2.01E+07 | 3.09E+07 | 8.02E+06 | 2.09E+07 | 5.87E+06 | 1.10E+07 | 0.68 |
| Com_10065_neg | 11-Dehydro thromboxane B2 | 4.93E+06 | 4.54E+06 | 2.10E+06 | 5.66E+06 | 2.03E+06 | 5.34E+06 | 3.21E+06 | 1.01E+06 | 5.62E+06 | 6.91E+06 | 0.92 |
| Com_1006_pos | LPC 18:3 | 2.29E+07 | 1.06E+08 | 1.01E+07 | 1.36E+07 | 5.74E+08 | 3.96E+08 | 1.53E+07 | 2.17E+08 | 8.92E+06 | 8.06E+06 | 0.90 |
| Com_10070_neg | Prostaglandin D3 | 1.52E+06 | 2.52E+06 | 1.12E+06 | 8.74E+05 | 2.06E+06 | 6.05E+06 | 5.47E+06 | 4.58E+06 | 4.94E+05 | 3.60E+06 | 0.21 |
| Com_10084_pos | α-Methylhistamine | 1.39E+07 | 1.48E+07 | 3.73E+07 | 3.39E+07 | 1.33E+07 | 1.69E+07 | 1.88E+07 | 1.23E+07 | 3.04E+07 | 2.01E+07 | 0.79 |
| Com_10088_pos | D-α-Tocopherol | 1.45E+07 | 1.36E+07 | 4.57E+06 | 2.55E+07 | 2.96E+07 | 2.77E+07 | 1.39E+07 | 2.19E+07 | 5.11E+06 | 2.01E+07 | 0.92 |
| Com_100_neg | 11(Z),14(Z)-Eicosadienoic acid | 1.01E+09 | 1.72E+09 | 4.91E+07 | 1.72E+09 | 1.03E+09 | 2.39E+08 | 9.55E+08 | 9.88E+07 | 2.21E+09 | 8.56E+08 | 0.78 |
| Com_10112_pos | EKK | 1.00E+07 | 1.86E+07 | 3.00E+06 | 3.37E+07 | 1.27E+07 | 1.80E+07 | 2.65E+07 | 2.79E+07 | 1.05E+07 | 2.93E+07 | 0.25 |
| Com_10120_pos | Vanillin | 1.15E+07 | 1.91E+07 | 9.46E+06 | 4.84E+06 | 1.04E+07 | 1.38E+07 | 1.42E+07 | 3.66E+07 | 1.03E+07 | 1.04E+07 | 0.25 |
| Com_10132_pos | 3-hydroxy-2-[5-nitro-2-(1-pyrrolidinyl)benzyl]propanenitrile | 2.47E+07 | 3.02E+07 | 1.12E+06 | 9.52E+06 | 1.99E+07 | 9.63E+06 | 2.51E+06 | 1.77E+07 | 6.54E+06 | 1.62E+07 | 0.73 |
| Com_10136_pos | DKK | 5.92E+06 | 9.88E+06 | 3.86E+06 | 1.18E+07 | 1.84E+07 | 2.67E+07 | 1.85E+07 | 3.65E+07 | 4.88E+06 | 1.32E+07 | 0.19 |
| Com_10166_pos | Carnosine | 9.68E+06 | 1.28E+07 | 1.20E+07 | 1.18E+07 | 1.70E+07 | 1.74E+07 | 2.41E+07 | 1.51E+07 | 2.36E+07 | 3.54E+07 | 0.01 |
| Com_10167_pos | 1-(3-ethyl-2,4-dihydroxy-6-methoxyphenyl)butan-1-one | 1.05E+07 | 7.81E+06 | 8.90E+06 | 9.32E+06 | 6.75E+06 | 1.11E+07 | 1.98E+07 | 8.21E+06 | 3.13E+07 | 1.46E+07 | 0.07 |
| Com_1018_neg | 3-Hydroxybenzoic acid | 4.95E+07 | 7.92E+07 | 2.24E+07 | 2.83E+07 | 1.62E+08 | 8.97E+07 | 2.33E+08 | 2.44E+08 | 2.02E+07 | 2.17E+07 | 0.62 |
| Com_10205_neg | 1,2,3-cyclopropanetricarboxylic acid | 2.43E+06 | 1.33E+06 | 1.03E+07 | 4.05E+06 | 3.43E+06 | 5.34E+06 | 1.09E+07 | 6.13E+06 | 2.39E+06 | 2.36E+06 | 0.53 |
| Com_10207_pos | N4-(4-chloro-2,5-dimethoxyphenyl)morpholine-4-carbothioamide | 2.61E+07 | 1.34E+07 | 2.40E+07 | 2.57E+07 | 9.36E+06 | 1.54E+07 | 4.43E+07 | 1.58E+07 | 1.94E+07 | 3.33E+07 | 0.43 |
| Com_10208_pos | LPE 20:2 | 2.69E+06 | 2.99E+07 | 4.58E+06 | 2.57E+06 | 7.57E+06 | 1.52E+06 | 8.48E+06 | 1.39E+07 | 2.13E+06 | 2.50E+06 | 0.54 |
| Com_1020_pos | Valerophenone | 5.85E+08 | 4.93E+08 | 2.20E+08 | 3.90E+08 | 4.33E+08 | 2.88E+08 | 2.99E+08 | 6.26E+07 | 6.00E+08 | 5.85E+08 | 0.47 |
| Com_10214_neg | Trenbolone acetate | 4.57E+05 | 5.16E+06 | 5.64E+06 | 5.64E+05 | 8.03E+06 | 3.05E+05 | 5.67E+05 | 5.43E+05 | 3.73E+05 | 6.20E+05 | 0.06 |
| Com_10220_pos | Anthranilic acid | 5.75E+06 | 1.14E+07 | 1.05E+07 | 3.31E+07 | 6.60E+06 | 8.27E+06 | 1.30E+07 | 5.80E+06 | 6.92E+06 | 9.85E+06 | 0.48 |
| Com_10222_pos | 13,14-Dihydro prostaglandin E1 | 2.69E+07 | 2.63E+07 | 3.32E+07 | 1.81E+07 | 2.90E+07 | 2.44E+07 | 2.10E+07 | 1.94E+07 | 2.94E+07 | 2.21E+07 | 0.33 |
| Com_10230_pos | S-Sulfo-L-cysteine | 2.76E+06 | 3.29E+06 | 5.45E+06 | 5.38E+06 | 1.45E+06 | 1.38E+07 | 5.90E+06 | 3.60E+07 | 5.91E+06 | 2.34E+06 | 0.12 |
| Com_1024_pos | L-Pyroglutamic acid | 2.07E+08 | 5.23E+08 | 3.12E+08 | 4.00E+08 | 2.06E+08 | 5.81E+08 | 2.30E+08 | 3.33E+08 | 4.38E+08 | 5.53E+08 | 0.31 |
| Com_10268_pos | ethyl 3-amino-4-(methylamino)benzoate | 9.27E+06 | 1.07E+07 | 9.25E+06 | 2.23E+06 | 2.88E+07 | 3.72E+06 | 6.17E+06 | 5.92E+06 | 3.87E+06 | 4.61E+06 | 0.20 |
| Com_10279_pos | 2-(1H-indol-3-yl)acetic acid | 1.48E+07 | 5.64E+06 | 1.43E+06 | 5.15E+06 | 2.88E+07 | 5.39E+06 | 4.10E+06 | 4.56E+06 | 2.96E+06 | 5.85E+06 | 0.42 |
| Com_1031_neg | 4-Methylphenol | 4.57E+07 | 1.41E+08 | 3.60E+07 | 9.32E+07 | 2.00E+08 | 5.27E+07 | 1.01E+07 | 1.91E+07 | 4.51E+07 | 5.56E+07 | 0.06 |
| Com_1032_pos | Isophorone | 1.70E+08 | 1.37E+08 | 3.00E+08 | 6.35E+08 | 2.08E+08 | 1.18E+08 | 2.29E+08 | 2.16E+08 | 2.21E+08 | 2.31E+08 | 0.48 |
| Com_10373_pos | 2-({2-oxo-2-[(2-oxo-3-azepanyl)amino]ethyl}sulfanyl)acetic acid | 4.42E+06 | 3.12E+06 | 3.56E+07 | 2.78E+06 | 1.04E+07 | 1.22E+07 | 5.94E+06 | 1.49E+07 | 7.32E+05 | 1.35E+06 | 0.52 |
| Com_1037_neg | Pseudouridine | 3.31E+07 | 4.19E+07 | 5.00E+07 | 6.97E+07 | 3.34E+07 | 1.47E+08 | 1.12E+08 | 2.53E+07 | 1.84E+07 | 6.14E+07 | 0.64 |
| Com_10380_pos | 3-[(2-phenyl-1H-imidazol-4-yl)methylene]-1,3-dihydro-2H-indol-2-one | 1.70E+07 | 2.06E+07 | 2.58E+07 | 8.31E+06 | 2.82E+07 | 1.49E+07 | 2.26E+07 | 2.55E+07 | 2.00E+07 | 1.63E+07 | 0.83 |
| Com_10385_pos | N-Arachidonoyl-L-serine | 1.11E+06 | 1.89E+06 | 3.56E+07 | 1.87E+06 | 2.43E+06 | 5.41E+06 | 1.60E+06 | 5.88E+06 | 1.65E+06 | 4.19E+06 | 0.99 |
| Com_10387_neg | Cetirizine N-oxide | 1.18E+06 | 1.72E+06 | 1.07E+06 | 4.24E+06 | 1.72E+06 | 3.83E+06 | 1.20E+07 | 7.53E+06 | 5.32E+06 | 6.81E+06 | 0.00 |
| Com_10390_pos | (S)-AL 8810 | 6.87E+05 | 2.67E+05 | 3.55E+07 | 5.98E+05 | 2.55E+05 | 2.39E+05 | 1.67E+06 | 1.01E+07 | 2.79E+05 | 3.29E+05 | 0.87 |
| Com_1042_neg | Thymidine | 6.78E+07 | 1.39E+08 | 7.75E+07 | 4.36E+07 | 1.24E+08 | 7.36E+07 | 7.45E+07 | 6.00E+07 | 5.57E+07 | 2.08E+08 | 0.98 |
| Com_10457_neg | 14,15-Leukotriene E4 | 6.21E+06 | 5.38E+06 | 3.65E+06 | 6.11E+06 | 4.36E+06 | 2.11E+06 | 3.26E+06 | 9.94E+05 | 4.91E+06 | 5.74E+06 | 0.15 |
| Com_10457_pos | D-(+)-Tryptophan | 1.53E+07 | 1.00E+07 | 3.51E+07 | 2.18E+07 | 5.53E+06 | 1.15E+07 | 8.04E+06 | 1.12E+07 | 1.18E+07 | 6.17E+06 | 0.26 |
| Com_1050_neg | N-Acetylsphingosine | 3.92E+07 | 1.32E+08 | 2.61E+08 | 6.52E+07 | 1.14E+08 | 5.15E+07 | 2.04E+08 | 1.19E+08 | 3.76E+07 | 7.07E+07 | 0.63 |
| Com_10512_pos | Ecgonine methyl ester | 6.01E+06 | 2.83E+07 | 6.00E+06 | 3.54E+06 | 4.98E+06 | 3.46E+06 | 2.85E+06 | 1.78E+06 | 7.03E+06 | 5.79E+06 | 0.18 |
| Com_10523_pos | Estriol | 1.50E+07 | 2.25E+07 | 1.17E+07 | 3.14E+07 | 8.76E+06 | 2.07E+07 | 2.52E+07 | 1.18E+07 | 2.89E+07 | 2.56E+07 | 0.34 |
| Com_10528_pos | 3-hydroxy-2-octylpentanedioic acid | 1.23E+07 | 7.47E+06 | 3.47E+07 | 4.68E+06 | 2.59E+07 | 9.69E+06 | 1.10E+07 | 7.89E+06 | 5.89E+06 | 6.08E+06 | 0.25 |
| Com_10546_pos | 2-(3,5-dimethyl-1H-pyrazol-4-yl)-3-nitropyridine | 1.47E+07 | 2.82E+07 | 2.98E+07 | 1.20E+07 | 1.03E+07 | 2.84E+06 | 6.59E+06 | 5.29E+06 | 1.08E+06 | 8.14E+06 | 0.01 |
| Com_1055_neg | δ-Gluconic acid δ-lactone | 1.98E+07 | 3.88E+07 | 3.96E+07 | 2.51E+07 | 5.87E+07 | 1.11E+08 | 3.18E+08 | 2.27E+08 | 3.99E+07 | 3.99E+07 | 0.06 |
| Com_10594_pos | Cytidine 5'-monophosphate (hydrate) | 3.80E+06 | 3.17E+06 | 3.43E+07 | 1.05E+07 | 4.52E+06 | 9.89E+05 | 4.54E+06 | 2.02E+06 | 6.25E+06 | 1.76E+07 | 0.39 |
| Com_105_pos | Uracil | 1.76E+09 | 2.72E+09 | 3.07E+09 | 5.30E+09 | 1.70E+09 | 1.66E+09 | 3.03E+09 | 1.27E+09 | 2.55E+09 | 6.56E+09 | 0.91 |
| Com_10609_pos | 1-Naphthol | 3.60E+07 | 1.54E+07 | 1.90E+06 | 9.13E+06 | 9.62E+06 | 1.00E+07 | 5.62E+06 | 2.83E+06 | 4.62E+06 | 5.37E+06 | 0.28 |
| Com_10613_pos | 2-(4,4-diphenyl-1-piperidinobuta-1,3-dienyl)phenyl acetate | 5.64E+06 | 1.72E+06 | 6.33E+05 | 8.13E+05 | 4.83E+05 | 5.42E+05 | 9.74E+05 | 4.12E+05 | 2.91E+07 | 7.80E+05 | 0.88 |
| Com_10620_pos | Methyltestosterone | 2.69E+07 | 2.43E+07 | 2.62E+07 | 2.27E+07 | 1.65E+07 | 2.64E+07 | 1.40E+07 | 2.01E+07 | 2.65E+07 | 3.28E+07 | 0.99 |
| Com_10627_neg | Kynurenic acid O-hexside | 2.29E+06 | 1.89E+06 | 4.58E+06 | 7.28E+06 | 2.92E+06 | 2.88E+06 | 1.05E+07 | 8.97E+06 | 3.64E+06 | 5.28E+06 | 0.19 |
| Com_10662_pos | cis-gondoic acid | 5.19E+06 | 4.00E+06 | 3.39E+07 | 7.32E+06 | 4.92E+06 | 1.97E+07 | 4.88E+06 | 6.35E+06 | 5.47E+06 | 7.84E+06 | 0.99 |
| Com_10697_pos | N-Formyl-L-methionine | 7.01E+06 | 5.45E+06 | 6.50E+06 | 8.64E+06 | 3.55E+06 | 2.24E+06 | 7.12E+06 | 3.33E+07 | 7.11E+06 | 9.19E+06 | 0.54 |
| Com_10698_pos | Ergosterol peroxide | 1.56E+07 | 7.31E+06 | 2.51E+07 | 6.75E+06 | 1.77E+07 | 1.66E+07 | 2.37E+07 | 7.88E+06 | 2.81E+07 | 3.25E+07 | 0.27 |
| Com_10710_pos | 1H-indene-3-carboxamide | 2.50E+07 | 2.57E+07 | 3.37E+07 | 1.99E+07 | 2.13E+07 | 1.08E+07 | 1.83E+07 | 1.11E+06 | 1.70E+07 | 3.08E+07 | 0.21 |
| Com_1071_neg | Prostaglandin A1 ethyl ester | 7.59E+07 | 9.29E+07 | 1.49E+08 | 1.70E+08 | 1.18E+08 | 2.29E+07 | 1.44E+08 | 1.12E+08 | 1.32E+08 | 1.30E+08 | 0.55 |
| Com_1071_pos | Y-L-Glutamyl-L-glutamic acid | 3.38E+07 | 4.71E+07 | 3.75E+08 | 6.17E+08 | 5.83E+07 | 5.08E+08 | 5.33E+07 | 2.07E+08 | 1.29E+08 | 2.17E+08 | 0.59 |
| Com_1075_neg | LPE 18:0 | 1.22E+07 | 1.00E+08 | 1.33E+07 | 1.10E+07 | 1.92E+08 | 7.81E+06 | 1.45E+08 | 1.77E+08 | 1.07E+06 | 2.04E+06 | 0.50 |
| Com_1076_pos | 2-Arachidonoyl glycerol | 1.68E+07 | 3.26E+07 | 7.81E+07 | 1.74E+07 | 1.38E+08 | 2.54E+07 | 3.65E+07 | 6.68E+08 | 3.22E+07 | 3.83E+07 | 0.60 |
| Com_1079_neg | LPG 17:0 | 6.64E+05 | 1.34E+08 | 1.45E+06 | 2.85E+06 | 8.56E+06 | 5.78E+05 | 2.41E+06 | 5.85E+06 | 6.67E+05 | 2.25E+06 | 0.32 |
| Com_10828_pos | QLK | 9.96E+06 | 1.64E+07 | 1.32E+06 | 1.96E+07 | 1.64E+07 | 2.73E+07 | 9.71E+06 | 2.10E+07 | 4.58E+06 | 1.83E+07 | 0.54 |
| Com_10863_pos | AKB48 N-(4-hydroxypentyl) metabolite | 3.63E+06 | 7.83E+06 | 2.05E+06 | 2.99E+07 | 5.77E+06 | 6.52E+06 | 1.31E+07 | 1.25E+07 | 5.41E+06 | 2.73E+07 | 0.34 |
| Com_1087_pos | Propionylcarnitine | 1.42E+07 | 2.85E+07 | 6.71E+08 | 2.79E+07 | 1.68E+07 | 4.49E+07 | 7.65E+06 | 5.68E+07 | 1.17E+07 | 2.64E+07 | 0.48 |
| Com_10907_pos | IPK | 1.05E+07 | 2.12E+07 | 4.76E+06 | 1.90E+07 | 8.81E+06 | 2.41E+07 | 1.39E+07 | 1.99E+07 | 1.16E+07 | 3.15E+07 | 0.15 |
| Com_10910_neg | 11β-Prostaglandin E2 | 6.71E+06 | 4.86E+06 | 5.04E+06 | 3.08E+06 | 3.61E+06 | 2.34E+06 | 5.29E+06 | 2.92E+06 | 4.86E+06 | 3.92E+06 | 0.37 |
| Com_10912_pos | 8-Isoprostaglandin F1β | 2.04E+07 | 2.67E+07 | 2.83E+07 | 1.25E+07 | 2.45E+07 | 1.77E+07 | 1.86E+07 | 1.36E+07 | 2.09E+07 | 2.55E+07 | 0.47 |
| Com_10919_pos | Methyl EudesMate | 3.00E+07 | 2.67E+07 | 3.07E+07 | 1.34E+07 | 1.19E+07 | 1.16E+07 | 2.17E+07 | 9.57E+06 | 2.10E+07 | 1.28E+07 | 0.21 |
| Com_10935_pos | 3-Methyladipic acid | 3.43E+07 | 2.08E+07 | 1.26E+07 | 1.51E+07 | 1.83E+07 | 1.22E+07 | 2.53E+07 | 1.16E+07 | 9.70E+06 | 1.18E+07 | 0.17 |
| Com_10941_pos | 7α-Hydroxytestosterone | 1.09E+07 | 8.57E+06 | 5.45E+06 | 7.15E+06 | 9.26E+06 | 6.82E+06 | 2.34E+07 | 5.69E+06 | 2.77E+07 | 8.66E+06 | 0.33 |
| Com_1094_pos | N8-Acetylspermidine | 4.02E+08 | 5.44E+08 | 5.46E+08 | 2.03E+08 | 2.69E+08 | 4.11E+08 | 2.17E+08 | 2.54E+08 | 1.87E+08 | 3.78E+08 | 0.29 |
| Com_10967_pos | Cholesterol | 3.14E+06 | 6.95E+06 | 7.85E+06 | 2.95E+07 | 4.36E+06 | 2.92E+06 | 5.38E+06 | 5.30E+06 | 1.71E+07 | 1.31E+07 | 0.95 |
| Com_10974_pos | N-(4-fluorophenyl)-N'-(2-piperidinophenyl)urea | 2.49E+06 | 1.95E+06 | 1.59E+06 | 8.62E+06 | 1.99E+06 | 2.67E+07 | 9.37E+06 | 1.06E+07 | 2.17E+06 | 3.71E+06 | 0.10 |
| Com_1098_neg | D-Alanyl-D-alanine | 1.74E+07 | 2.99E+07 | 2.07E+08 | 1.98E+08 | 2.17E+07 | 2.99E+07 | 4.78E+06 | 3.37E+07 | 1.07E+08 | 2.17E+08 | 0.75 |
| Com_10993_pos | HRH | 4.96E+05 | 4.58E+05 | 5.06E+05 | 5.02E+05 | 4.85E+05 | 3.93E+05 | 5.63E+05 | 5.04E+05 | 2.75E+07 | 6.09E+05 | 0.35 |
| Com_109_pos | Indole-3-acrylic acid | 1.76E+09 | 2.52E+09 | 1.57E+09 | 3.40E+09 | 3.64E+09 | 4.02E+09 | 3.30E+09 | 6.66E+09 | 1.68E+09 | 2.00E+09 | 0.44 |
| Com_11011_pos | LLK | 8.12E+06 | 1.20E+07 | 4.76E+06 | 2.34E+07 | 2.55E+07 | 1.65E+07 | 2.30E+07 | 3.18E+07 | 7.44E+06 | 2.22E+07 | 0.36 |
| Com_1102_neg | Guanosine | 1.28E+08 | 1.28E+08 | 3.55E+07 | 1.45E+08 | 1.05E+08 | 5.58E+07 | 2.77E+07 | 2.23E+08 | 1.50E+07 | 1.69E+08 | 0.45 |
| Com_1103_neg | Gluconic acid | 1.37E+07 | 1.19E+07 | 7.80E+07 | 4.39E+07 | 4.05E+07 | 1.37E+08 | 1.96E+08 | 1.28E+08 | 1.91E+07 | 5.19E+07 | 0.11 |
| Com_1103_pos | Cortisol | 1.76E+07 | 5.17E+07 | 2.14E+07 | 4.31E+07 | 1.73E+07 | 5.45E+08 | 6.81E+07 | 1.28E+08 | 3.13E+07 | 1.50E+08 | 0.03 |
| Com_11043_neg | N-Methylnicotinamide | 2.80E+06 | 3.39E+06 | 2.09E+06 | 1.93E+06 | 6.95E+06 | 2.42E+06 | 3.15E+06 | 8.01E+06 | 8.90E+05 | 2.78E+06 | 0.79 |
| Com_11063_pos | 5,6,7-trihydroxy-2-(4-methoxyphenyl)-4H-chromen-4-one | 5.50E+06 | 4.04E+06 | 8.20E+05 | 6.09E+06 | 5.53E+06 | 2.64E+07 | 1.92E+07 | 2.55E+07 | 1.98E+06 | 1.11E+07 | 0.08 |
| Com_11073_neg | N-Acetyl-1-aspartylglutamic acid | 2.76E+05 | 4.61E+05 | 3.27E+06 | 2.40E+06 | 2.73E+06 | 2.62E+06 | 3.59E+06 | 8.27E+06 | 5.81E+05 | 1.99E+06 | 0.33 |
| Com_11078_pos | 5-fluoro AB-PINACA N-(4-hydroxypentyl) metabolite | 1.61E+06 | 1.51E+06 | 1.44E+06 | 2.90E+07 | 1.36E+06 | 7.19E+06 | 2.59E+07 | 7.23E+06 | 2.80E+06 | 3.90E+06 | 0.23 |
| Com_11081_neg | 2,6-dimethoxy-N-(1-methyl-1H-pyrazol-5-yl)benzamide | 4.09E+05 | 4.60E+05 | 2.30E+05 | 7.64E+05 | 2.92E+06 | 5.07E+06 | 8.09E+05 | 5.16E+06 | 3.22E+05 | 1.43E+06 | 0.22 |
| Com_11092_neg | Hematoxylin | 6.59E+06 | 3.92E+06 | 5.34E+06 | 3.55E+06 | 4.00E+06 | 4.91E+06 | 9.43E+06 | 4.52E+06 | 4.49E+06 | 2.62E+06 | 0.85 |
| Com_11106_neg | N-[4-(aminosulfonyl)phenyl]-2-[3,4-dihydro-1(2H)-quinolinyl]acetamide | 1.92E+06 | 2.17E+06 | 3.73E+06 | 4.61E+06 | 1.25E+06 | 4.05E+06 | 4.71E+06 | 1.52E+06 | 4.68E+06 | 3.41E+06 | 0.33 |
| Com_1110_pos | 7-Methylguanine | 6.35E+07 | 1.17E+08 | 7.86E+07 | 2.20E+08 | 8.95E+07 | 5.42E+08 | 5.59E+08 | 5.62E+08 | 8.80E+07 | 3.76E+08 | 0.02 |
| Com_11110_neg | 8-iso-15-keto Prostaglandin F2α | 1.28E+06 | 1.44E+06 | 2.39E+06 | 4.45E+06 | 6.54E+06 | 3.67E+06 | 7.80E+06 | 8.20E+06 | 1.65E+06 | 4.66E+06 | 0.25 |
| Com_1112_neg | Glycerol 3-phosphate | 3.35E+06 | 2.07E+06 | 2.43E+08 | 2.81E+07 | 8.96E+06 | 4.17E+06 | 2.69E+07 | 1.41E+07 | 4.32E+06 | 7.77E+06 | 0.67 |
| Com_11140_pos | Ifenprodil | 1.08E+07 | 1.37E+07 | 1.46E+07 | 5.31E+06 | 2.51E+07 | 2.15E+06 | 7.82E+06 | 9.74E+06 | 2.37E+06 | 6.44E+06 | 0.05 |
| Com_11149_pos | 1-[4-hydroxy-3-(3-methylbut-2-en-1-yl)phenyl]ethan-1-one | 2.34E+07 | 4.00E+06 | 3.17E+07 | 2.80E+07 | 1.52E+07 | 1.44E+07 | 1.37E+07 | 2.01E+06 | 2.24E+07 | 1.44E+07 | 0.44 |
| Com_11151_pos | D-δ-Tocopherol | 5.18E+06 | 2.57E+06 | 2.73E+07 | 2.87E+07 | 2.64E+06 | 6.52E+06 | 6.66E+06 | 3.24E+06 | 2.04E+07 | 2.51E+07 | 0.78 |
| Com_11152_pos | 3-Methoxycinnamic acid | 6.13E+06 | 4.18E+06 | 3.62E+06 | 1.09E+07 | 4.04E+06 | 1.51E+07 | 1.10E+07 | 7.56E+06 | 8.17E+06 | 3.04E+07 | 0.03 |
| Com_11183_pos | 2-ethyl-2-(3-methoxyphenyl)cyclohexanone oxime | 2.15E+07 | 2.57E+07 | 2.23E+06 | 5.80E+06 | 8.40E+06 | 3.27E+06 | 4.67E+05 | 8.84E+05 | 5.13E+06 | 9.67E+06 | 0.10 |
| Com_111_pos | DL-Tryptophan | 1.74E+09 | 2.50E+09 | 1.56E+09 | 3.38E+09 | 3.62E+09 | 3.98E+09 | 3.27E+09 | 6.61E+09 | 1.67E+09 | 3.99E+09 | 0.21 |
| Com_11217_pos | Methyl indole-3-acetate | 7.10E+06 | 1.76E+07 | 6.53E+06 | 1.47E+07 | 6.33E+06 | 6.92E+06 | 3.79E+07 | 2.63E+07 | 1.55E+07 | 1.78E+07 | 0.11 |
| Com_11221_neg | (3-Methoxy-4-hydroxyphenyl)ethylene glycol sulfate | 2.94E+05 | 2.61E+05 | 4.62E+05 | 1.23E+06 | 6.96E+05 | 9.79E+05 | 4.78E+05 | 8.05E+06 | 2.12E+05 | 4.26E+05 | 0.51 |
| Com_11223_pos | methyl 3,4,5-trihydroxycyclohex-1-ene-1-carboxylate | 2.25E+07 | 2.02E+07 | 3.14E+07 | 1.24E+07 | 2.13E+07 | 2.48E+07 | 2.58E+07 | 2.05E+07 | 2.57E+07 | 1.12E+07 | 1.00 |
| Com_11250_pos | Paracetamol | 2.49E+07 | 2.55E+07 | 8.52E+06 | 8.56E+06 | 2.45E+07 | 9.39E+06 | 1.19E+07 | 1.13E+07 | 6.60E+06 | 1.66E+07 | 0.21 |
| Com_1131_pos | Valylproline | 2.25E+08 | 5.23E+08 | 3.23E+07 | 2.93E+08 | 2.77E+08 | 4.16E+08 | 7.59E+07 | 3.22E+08 | 3.04E+07 | 2.55E+08 | 0.70 |
| Com_1132_pos | N-Methylisoleucine | 4.85E+08 | 4.98E+08 | 3.10E+07 | 1.45E+08 | 5.09E+08 | 8.68E+07 | 1.93E+08 | 1.52E+08 | 2.31E+08 | 2.79E+08 | 0.70 |
| Com_11345_pos | 1-(3-phenylpropanoyl)-4-piperidinecarboxylic acid | 3.75E+06 | 5.09E+06 | 9.53E+06 | 8.93E+06 | 7.81E+06 | 1.75E+07 | 1.04E+07 | 3.05E+07 | 3.52E+06 | 1.85E+07 | 0.15 |
| Com_11375_pos | 1-hydroxy-2-phenyl-1,5,6,7-tetrahydro-4H-benzimidazol-4-one | 2.00E+07 | 2.40E+07 | 2.27E+07 | 1.11E+07 | 1.31E+07 | 1.26E+07 | 1.39E+07 | 8.68E+06 | 2.62E+07 | 1.57E+07 | 0.45 |
| Com_11381_pos | N6-Me-dA | 2.99E+06 | 7.45E+06 | 7.30E+06 | 9.88E+06 | 3.87E+06 | 3.85E+06 | 2.90E+06 | 3.93E+06 | 1.11E+07 | 2.96E+07 | 0.74 |
| Com_11383_pos | MGMG (18:2) | 3.41E+06 | 9.21E+06 | 8.90E+05 | 4.99E+06 | 1.35E+06 | 2.53E+07 | 8.90E+05 | 2.66E+06 | 2.39E+06 | 5.97E+06 | 0.68 |
| Com_11395_pos | Tetranor-12R-HETE | 8.26E+06 | 6.61E+06 | 5.08E+06 | 2.78E+07 | 5.07E+06 | 7.86E+06 | 1.69E+07 | 7.76E+06 | 1.81E+07 | 1.36E+07 | 0.34 |
| Com_1139_pos | Sedanolide | 3.29E+08 | 3.34E+08 | 3.85E+08 | 2.70E+08 | 3.73E+08 | 1.99E+08 | 2.80E+08 | 7.18E+07 | 5.44E+08 | 3.75E+08 | 0.40 |
| Com_11400_pos | (+/-)11(12)-DiHET | 2.36E+07 | 1.65E+07 | 8.06E+06 | 1.81E+07 | 7.80E+06 | 2.34E+07 | 1.63E+07 | 1.17E+07 | 1.38E+07 | 1.70E+07 | 0.52 |
| Com_11401_pos | 2-Thiocytidine | 4.74E+06 | 5.09E+06 | 1.19E+07 | 1.62E+07 | 5.72E+06 | 5.43E+06 | 1.59E+07 | 6.24E+06 | 4.22E+06 | 2.95E+07 | 0.69 |
| Com_11429_neg | 3-Phenoxybenzoic acid | 3.52E+05 | 2.91E+05 | 3.83E+05 | 5.08E+05 | 3.08E+05 | 4.77E+06 | 5.24E+05 | 1.99E+06 | 2.23E+05 | 3.30E+05 | 0.23 |
| Com_11430_neg | Salvinorin B | 2.47E+06 | 4.52E+06 | 3.22E+06 | 2.55E+06 | 2.29E+06 | 2.54E+06 | 3.51E+06 | 1.18E+06 | 3.46E+06 | 3.51E+06 | 0.70 |
| Com_11446_pos | 6-methyl-7-nitro-2,3-dihydro-1,4-benzodioxine | 2.82E+07 | 2.10E+07 | 1.92E+07 | 1.66E+07 | 2.04E+07 | 1.83E+07 | 2.47E+07 | 1.47E+07 | 2.07E+07 | 2.00E+07 | 0.59 |
| Com_1144_pos | Coumarin | 8.39E+07 | 1.64E+08 | 1.22E+08 | 1.96E+08 | 2.22E+08 | 3.23E+08 | 2.00E+08 | 6.27E+08 | 1.09E+08 | 3.09E+08 | 0.13 |
| Com_1147_neg | Arachidic acid | 5.59E+07 | 1.23E+08 | 2.04E+08 | 1.46E+08 | 1.18E+08 | 2.31E+07 | 1.76E+08 | 1.12E+08 | 1.14E+08 | 7.88E+07 | 0.41 |
| Com_1147_pos | Azetidine-2-carboxylic acid | 2.12E+08 | 2.49E+08 | 5.50E+08 | 4.54E+08 | 2.38E+08 | 3.62E+08 | 3.95E+08 | 3.26E+08 | 4.92E+08 | 6.09E+08 | 0.22 |
| Com_11493_neg | 8(R)-Hydroxy-(5Z,9E,11Z,14Z)-eicosatetraenoic acid | 3.65E+06 | 3.66E+06 | 5.30E+06 | 4.60E+06 | 3.58E+06 | 3.23E+06 | 5.67E+06 | 5.32E+06 | 4.38E+06 | 4.29E+06 | 0.50 |
| Com_1149_neg | 4-Ethylphenol | 7.03E+07 | 7.31E+07 | 8.43E+06 | 9.33E+07 | 1.65E+08 | 7.49E+07 | 1.31E+08 | 3.96E+07 | 1.20E+08 | 7.88E+07 | 0.56 |
| Com_1150_pos | 2,6-Di-tert-butyl-1,4-benzoquinone | 4.98E+08 | 4.56E+08 | 2.66E+08 | 2.89E+08 | 3.92E+08 | 5.20E+08 | 2.42E+08 | 8.72E+07 | 4.54E+08 | 4.72E+08 | 0.58 |
| Com_11526_pos | indoline-2-carboxylic acid | 1.20E+07 | 1.02E+07 | 1.69E+07 | 7.28E+06 | 7.55E+06 | 4.70E+06 | 9.65E+06 | 2.74E+06 | 2.56E+07 | 1.22E+07 | 0.63 |
| Com_11531_pos | 9,10-Dihome | 2.02E+07 | 1.35E+07 | 2.39E+07 | 9.30E+06 | 1.56E+07 | 8.13E+06 | 1.90E+07 | 1.37E+07 | 2.56E+07 | 1.67E+07 | 0.97 |
| Com_11540_neg | Prostaglandin B2 | 7.46E+05 | 7.31E+05 | 8.39E+06 | 4.79E+05 | 4.86E+05 | 2.26E+06 | 7.88E+05 | 5.37E+05 | 4.13E+05 | 4.87E+05 | 0.60 |
| Com_11581_pos | 1-(2-pyridin-2-yldiazenyl)-2-naphthol | 7.31E+06 | 4.82E+06 | 2.99E+07 | 3.79E+06 | 1.14E+07 | 9.24E+06 | 6.88E+06 | 8.84E+06 | 1.08E+07 | 6.95E+06 | 0.97 |
| Com_1159_pos | Indole-3-acetic acid | 2.30E+08 | 3.94E+08 | 5.10E+07 | 6.82E+07 | 4.95E+08 | 9.91E+07 | 3.49E+08 | 2.55E+08 | 1.03E+08 | 2.06E+08 | 0.95 |
| Com_11608_pos | 3-Acetoxyurs-12-en-23-oic acid | 1.92E+07 | 1.84E+07 | 2.21E+07 | 2.50E+07 | 1.31E+07 | 1.35E+07 | 2.20E+07 | 3.95E+06 | 2.54E+07 | 2.48E+07 | 0.53 |
| Com_1160_pos | Oxaceprol | 5.24E+07 | 1.48E+08 | 7.86E+07 | 2.30E+08 | 4.26E+08 | 3.66E+08 | 2.86E+08 | 6.15E+08 | 6.05E+07 | 3.20E+08 | 0.29 |
| Com_1161_neg | 2,6-Dihydroxybenzoic acid | 7.75E+07 | 1.20E+08 | 1.75E+07 | 1.04E+07 | 1.54E+08 | 3.51E+07 | 1.19E+08 | 9.43E+07 | 1.15E+07 | 5.64E+07 | 0.99 |
| Com_11631_pos | ACar 20:4 | 3.21E+06 | 1.08E+07 | 2.07E+06 | 1.15E+06 | 6.24E+06 | 2.45E+07 | 1.54E+06 | 2.30E+07 | 1.18E+06 | 1.56E+06 | 0.78 |
| Com_11637_neg | PG (5:0/20:1) | 5.07E+05 | 4.11E+05 | 7.43E+05 | 6.09E+05 | 6.14E+05 | 3.52E+05 | 1.01E+07 | 6.55E+05 | 4.10E+05 | 6.56E+05 | 0.49 |
| Com_11657_neg | 5-S-cysteinyldopaquinone | 3.71E+05 | 3.63E+05 | 2.14E+05 | 2.01E+05 | 6.23E+06 | 8.01E+05 | 3.09E+05 | 6.07E+05 | 1.84E+05 | 5.65E+05 | 0.82 |
| Com_11666_pos | 11β-Hydroxyandrosterone | 2.55E+07 | 1.45E+07 | 1.91E+07 | 1.69E+07 | 2.35E+07 | 1.13E+07 | 1.89E+07 | 2.01E+07 | 2.27E+07 | 1.98E+07 | 0.66 |
| Com_11677_pos | (2S)-2-(2-hydroxypropan-2-yl)-2H,3H,7H-furo[3,2-g]chromen-7-one | 2.28E+06 | 3.12E+06 | 2.96E+07 | 2.16E+06 | 2.49E+06 | 9.68E+06 | 1.19E+07 | 7.33E+06 | 2.69E+06 | 3.64E+06 | 0.51 |
| Com_116_pos | Pipecolic acid | 2.22E+08 | 6.28E+08 | 2.52E+09 | 5.91E+09 | 2.00E+08 | 5.55E+08 | 2.34E+08 | 1.24E+08 | 6.69E+08 | 2.57E+09 | 0.54 |
| Com_11706_pos | 5-chloro-6-(trifluoromethyl)-1,3-dihydro-2H-benzimidazole-2-thione | 7.27E+06 | 1.33E+07 | 5.30E+06 | 1.15E+07 | 2.33E+07 | 4.56E+06 | 1.46E+06 | 2.45E+06 | 8.17E+06 | 8.97E+06 | 0.06 |
| Com_11717_pos | Dimetghyl 4-Hydroxyisophthalate | 4.06E+06 | 7.46E+06 | 3.87E+06 | 2.96E+06 | 5.14E+06 | 1.63E+07 | 9.81E+06 | 2.90E+07 | 3.36E+06 | 6.47E+06 | 0.10 |
| Com_11738_pos | 3-Methoxy prostaglandin F1α | 7.07E+06 | 1.10E+07 | 2.11E+06 | 2.83E+06 | 1.17E+07 | 3.47E+06 | 2.41E+06 | 2.89E+07 | 2.05E+06 | 2.23E+06 | 0.62 |
| Com_11773_pos | Saxitoxin | 1.93E+06 | 5.21E+06 | 1.46E+06 | 1.23E+07 | 2.57E+06 | 1.49E+07 | 2.22E+07 | 1.11E+07 | 2.48E+07 | 1.27E+07 | 0.01 |
| Com_11788_neg | Gibberellin A4 | 3.15E+06 | 4.26E+06 | 4.21E+06 | 2.89E+06 | 4.15E+06 | 1.70E+06 | 3.83E+06 | 9.94E+05 | 3.40E+06 | 3.86E+06 | 0.20 |
| Com_1178_pos | Nicotinate ribonucleoside | 1.44E+08 | 4.67E+07 | 2.19E+08 | 1.84E+08 | 2.37E+08 | 3.24E+08 | 4.79E+08 | 2.37E+08 | 5.24E+08 | 5.25E+07 | 0.32 |
| Com_11796_pos | N-Acetyldopamine | 1.42E+06 | 3.58E+05 | 6.84E+05 | 1.46E+07 | 4.29E+06 | 4.13E+06 | 2.07E+06 | 2.87E+07 | 1.36E+06 | 4.50E+06 | 0.35 |
| Com_11859_neg | N-(2,6-difluorophenyl)-2-(4-nitrophenyl)acetamide | 1.86E+06 | 4.21E+06 | 2.26E+06 | 1.29E+06 | 1.85E+06 | 1.61E+06 | 1.63E+06 | 9.35E+05 | 1.94E+06 | 1.71E+06 | 0.20 |
| Com_11860_pos | DNK | 1.87E+06 | 2.60E+06 | 2.03E+06 | 1.25E+06 | 2.29E+07 | 7.42E+06 | 5.65E+06 | 2.12E+07 | 1.68E+06 | 1.82E+06 | 0.53 |
| Com_11867_pos | D-Erythrose 4-phosphate | 1.11E+07 | 2.05E+07 | 1.04E+07 | 1.49E+07 | 1.64E+07 | 2.37E+07 | 2.37E+07 | 1.84E+07 | 1.15E+07 | 2.43E+07 | 0.13 |
| Com_1187_neg | LPS 15:0 | 1.98E+07 | 4.93E+07 | 2.18E+06 | 3.53E+07 | 1.65E+08 | 7.36E+06 | 4.46E+06 | 9.06E+07 | 1.94E+06 | 1.35E+07 | 0.32 |
| Com_1188_pos | DL-Lysine | 1.49E+08 | 2.67E+08 | 2.25E+08 | 4.02E+08 | 2.20E+08 | 2.92E+08 | 9.44E+07 | 6.00E+08 | 1.81E+08 | 5.16E+08 | 0.74 |
| Com_11892_pos | PC (18:1e/2:0) | 1.65E+06 | 4.37E+06 | 1.91E+06 | 3.37E+06 | 2.00E+07 | 1.27E+07 | 9.44E+06 | 2.84E+07 | 5.31E+06 | 1.25E+06 | 0.38 |
| Com_11895_pos | 3-(3-methylbut-2-en-1-yl)-3H-purin-6-amine | 7.92E+06 | 1.12E+07 | 8.85E+06 | 2.60E+07 | 2.64E+06 | 1.88E+06 | 2.32E+07 | 1.81E+06 | 6.59E+06 | 6.82E+06 | 0.39 |
| Com_118_pos | Tryptamine | 1.19E+07 | 6.12E+07 | 1.47E+07 | 1.95E+07 | 5.05E+09 | 2.69E+07 | 1.22E+08 | 2.36E+08 | 6.20E+07 | 1.53E+07 | 0.96 |
| Com_11907_pos | (2E)-3-(3,4-dimethoxyphenyl)prop-2-enoic acid | 1.14E+07 | 2.04E+07 | 2.87E+07 | 8.56E+06 | 1.72E+07 | 1.61E+07 | 1.54E+07 | 2.16E+07 | 1.43E+07 | 1.52E+07 | 0.88 |
| Com_11952_pos | INK | 1.09E+07 | 2.32E+07 | 4.01E+06 | 2.17E+07 | 1.60E+07 | 2.22E+07 | 1.15E+07 | 2.70E+07 | 6.85E+06 | 2.15E+07 | 0.62 |
| Com_11967_pos | 20-Carboxy-Leukotriene B4 | 2.22E+07 | 1.94E+07 | 1.30E+07 | 2.58E+07 | 8.22E+06 | 2.09E+07 | 1.12E+07 | 6.27E+06 | 2.39E+07 | 2.24E+07 | 0.81 |
| Com_11974_pos | Cortisone | 1.45E+07 | 1.52E+07 | 6.97E+06 | 1.87E+07 | 1.34E+07 | 2.34E+07 | 2.12E+07 | 1.03E+07 | 1.14E+07 | 4.89E+06 | 0.86 |
| Com_1197_neg | Isorhapontigenin | 1.57E+08 | 3.03E+07 | 4.68E+05 | 3.83E+07 | 3.57E+07 | 1.27E+07 | 1.08E+07 | 2.19E+07 | 1.76E+07 | 1.67E+06 | 0.54 |
| Com_119_pos | 6-Methylquinoline | 1.14E+07 | 6.07E+07 | 1.42E+07 | 1.89E+07 | 5.05E+09 | 2.64E+07 | 9.57E+06 | 5.19E+06 | 8.22E+06 | 1.47E+07 | 0.20 |
| Com_12057_pos | N-Desmethylclomipramine | 1.58E+07 | 6.93E+06 | 2.43E+07 | 1.60E+07 | 2.04E+07 | 7.82E+06 | 1.23E+07 | 2.78E+07 | 8.20E+06 | 9.77E+06 | 0.40 |
| Com_12083_pos | VNH | 2.95E+07 | 1.76E+07 | 2.78E+06 | 8.88E+06 | 9.03E+06 | 1.60E+07 | 1.46E+07 | 4.12E+06 | 7.31E+06 | 9.32E+06 | 0.82 |
| Com_12110_neg | 11-keto Testosterone (CRM) | 4.91E+06 | 4.05E+06 | 6.94E+06 | 3.78E+06 | 4.56E+06 | 4.06E+06 | 5.39E+06 | 2.28E+06 | 2.75E+06 | 2.84E+06 | 0.09 |
| Com_1212_neg | Corchorifatty acid F | 7.19E+07 | 1.12E+08 | 1.20E+08 | 3.32E+07 | 5.09E+07 | 2.90E+07 | 7.92E+07 | 3.31E+07 | 5.35E+07 | 4.77E+07 | 0.20 |
| Com_12174_pos | RLH | 1.43E+07 | 2.26E+07 | 1.26E+06 | 1.85E+06 | 2.35E+06 | 1.10E+06 | 1.28E+06 | 1.27E+06 | 2.03E+06 | 5.50E+06 | 0.22 |
| Com_1220_neg | Naringenin | 1.60E+07 | 1.03E+07 | 6.73E+06 | 2.27E+07 | 1.66E+07 | 1.17E+08 | 5.50E+07 | 9.77E+07 | 3.60E+06 | 8.65E+06 | 0.34 |
| Com_1221_pos | (6E,10E)-3,7,11,15-tetramethylhexadeca-1,6,10,14-tetraene-3,5,9-triol | 4.21E+07 | 6.19E+07 | 2.02E+07 | 3.76E+07 | 4.66E+08 | 2.32E+08 | 3.53E+08 | 1.41E+08 | 2.66E+07 | 3.40E+07 | 0.53 |
| Com_1223_neg | 13,14-dihydro Prostaglandin F1α | 2.02E+07 | 1.66E+07 | 2.09E+08 | 2.94E+07 | 7.74E+06 | 7.22E+06 | 3.27E+07 | 1.28E+07 | 4.09E+07 | 5.35E+07 | 0.80 |
| Com_12257_pos | 2-(tert-butyl)-6,7-dimethoxy-4H-3,1-benzoxazin-4-one | 5.50E+06 | 1.26E+07 | 2.75E+07 | 6.01E+06 | 2.16E+07 | 1.29E+07 | 8.74E+06 | 1.39E+07 | 6.99E+06 | 5.82E+06 | 0.49 |
| Com_12266_neg | N1-(3-pyridyl)-2,3,4,5,6-pentamethylbenzene-1-sulfonamide | 3.15E+05 | 6.55E+05 | 1.40E+06 | 1.34E+06 | 8.85E+05 | 2.86E+06 | 9.49E+05 | 4.60E+06 | 3.86E+06 | 1.47E+06 | 0.03 |
| Com_12284_pos | Nicotinic acid mononucleotide | 6.92E+05 | 6.56E+05 | 2.75E+07 | 1.07E+07 | 2.31E+06 | 9.20E+05 | 1.15E+06 | 1.16E+06 | 1.17E+06 | 1.19E+06 | 0.24 |
| Com_12344_pos | Pilocarpine | 2.87E+07 | 1.39E+07 | 9.95E+05 | 7.95E+06 | 4.71E+06 | 3.80E+06 | 1.64E+06 | 1.64E+06 | 8.74E+06 | 1.74E+07 | 0.56 |
| Com_12346_neg | N1-(4-morpholinophenyl)-2-methylbut-2-enamide | 2.20E+06 | 3.00E+06 | 3.15E+06 | 3.32E+06 | 9.54E+05 | 2.11E+06 | 2.59E+06 | 5.55E+05 | 3.81E+06 | 3.80E+06 | 0.85 |
| Com_12360_pos | 4-Phenyl-3-buten-2-one | 1.31E+07 | 1.17E+07 | 2.72E+07 | 9.53E+06 | 1.11E+07 | 8.12E+06 | 1.02E+07 | 4.98E+06 | 1.31E+07 | 1.41E+07 | 0.22 |
| Com_12363_neg | Undecanedioic acid | 3.51E+06 | 2.37E+06 | 4.23E+06 | 2.13E+06 | 5.57E+06 | 2.72E+06 | 4.70E+06 | 2.53E+06 | 2.19E+06 | 4.40E+06 | 0.81 |
| Com_1238_pos | DL-Serine | 2.92E+08 | 3.19E+08 | 2.70E+08 | 4.73E+08 | 3.66E+08 | 3.47E+08 | 5.62E+08 | 4.18E+08 | 2.42E+08 | 5.53E+08 | 0.36 |
| Com_12409_neg | 5-(2-pyridinyl)-N-[2-(trifluoromethyl)phenyl]-2-thiophenesulfonamide | 8.22E+05 | 3.78E+05 | 2.94E+06 | 5.87E+06 | 1.39E+06 | 4.72E+05 | 2.43E+06 | 3.17E+06 | 2.60E+06 | 4.99E+06 | 0.57 |
| Com_12412_neg | 3-amino-2,6-diphenyl-4,7-dihydro-2H-pyrazolo[3,4-d]pyrimidin-4-one | 4.25E+05 | 4.66E+05 | 2.44E+05 | 1.12E+06 | 1.83E+05 | 4.06E+06 | 2.91E+05 | 5.48E+05 | 2.81E+05 | 4.21E+05 | 0.51 |
| Com_12458_pos | ethyl 6-chloro-2-(methylthio)-4-morpholinoquinoline-3-carboxylate | 4.68E+05 | 4.97E+05 | 5.77E+05 | 9.45E+06 | 8.62E+05 | 1.37E+07 | 2.40E+07 | 7.20E+06 | 2.28E+07 | 1.70E+07 | 0.01 |
| Com_12487_pos | N-(9-oxodecyl)acetamide | 1.60E+06 | 1.06E+06 | 2.68E+07 | 7.38E+05 | 1.04E+06 | 8.67E+05 | 1.91E+06 | 2.21E+06 | 1.09E+07 | 9.30E+05 | 0.99 |
| Com_12493_neg | N1-(6-methyl-4-oxo-3,4-dihydroquinazolin-2-yl)-4-nitrobenzamide | 5.23E+06 | 1.50E+06 | 3.46E+06 | 1.94E+06 | 2.38E+05 | 2.51E+06 | 3.65E+06 | 8.02E+05 | 2.63E+06 | 2.13E+06 | 0.70 |
| Com_1249_pos | Palmitoyl ethanolamide | 2.29E+08 | 3.09E+08 | 3.66E+08 | 3.93E+08 | 3.48E+08 | 1.30E+08 | 5.09E+08 | 5.60E+08 | 2.14E+08 | 2.27E+08 | 0.66 |
| Com_1251_neg | Xanthosine | 5.56E+06 | 5.40E+06 | 2.02E+08 | 1.18E+08 | 1.10E+07 | 1.40E+06 | 2.82E+07 | 9.38E+06 | 9.48E+06 | 7.10E+07 | 0.52 |
| Com_12521_pos | 1-(3,4-dimethoxyphenyl)ethan-1-one oxime | 1.39E+07 | 8.82E+06 | 2.18E+07 | 6.44E+06 | 5.20E+06 | 5.87E+06 | 6.34E+06 | 8.11E+06 | 2.27E+07 | 1.90E+07 | 0.85 |
| Com_12529_pos | 4-oxo-5-phenylpentanoic acid | 4.59E+06 | 3.43E+06 | 2.05E+06 | 1.14E+07 | 2.82E+06 | 1.09E+07 | 3.06E+07 | 1.54E+07 | 2.27E+07 | 2.19E+07 | 0.00 |
| Com_12531_pos | Gly-Tyr-Ala | 1.76E+07 | 2.17E+07 | 1.58E+07 | 1.46E+07 | 2.02E+07 | 3.10E+06 | 1.14E+07 | 1.26E+07 | 6.92E+06 | 2.07E+07 | 0.11 |
| Com_1254_neg | Sulfoacetic acid | 4.04E+07 | 3.25E+07 | 2.19E+07 | 2.26E+07 | 4.07E+07 | 1.13E+08 | 2.39E+07 | 6.83E+07 | 2.64E+07 | 1.79E+07 | 0.55 |
| Com_12582_pos | Tetrahydroaldosterone | 8.30E+06 | 1.53E+07 | 1.02E+07 | 2.40E+07 | 1.54E+07 | 1.14E+07 | 1.93E+07 | 1.20E+07 | 1.79E+07 | 1.28E+07 | 0.83 |
| Com_12591_pos | 17alpha-Hydroxyprogesterone | 7.35E+06 | 2.16E+07 | 6.96E+06 | 1.19E+07 | 9.51E+06 | 1.86E+07 | 1.26E+07 | 4.76E+06 | 1.67E+07 | 1.54E+07 | 0.62 |
| Com_12607_neg | Homocysteic acid | 1.43E+06 | 3.73E+06 | 7.32E+05 | 5.58E+05 | 2.12E+06 | 3.18E+06 | 2.58E+06 | 2.11E+06 | 7.36E+05 | 6.54E+05 | 0.81 |
| Com_12642_pos | 3-hydroxy-3-methylpentanedioic acid | 2.57E+07 | 1.90E+07 | 2.22E+07 | 2.09E+07 | 1.69E+07 | 1.61E+07 | 2.69E+07 | 2.50E+07 | 2.17E+07 | 2.02E+07 | 0.72 |
| Com_12656_pos | pentane-1,2,3,4,5-pentol | 2.19E+06 | 1.71E+06 | 1.05E+06 | 2.38E+07 | 4.24E+06 | 9.91E+05 | 3.63E+06 | 3.87E+06 | 3.48E+06 | 1.61E+06 | 0.61 |
| Com_1266_pos | 4'-Methoxyacetophenone | 5.84E+08 | 1.27E+08 | 4.92E+07 | 1.56E+08 | 1.32E+08 | 4.19E+08 | 7.28E+07 | 2.31E+07 | 9.28E+07 | 1.80E+08 | 0.57 |
| Com_12682_neg | 4-phenyl-2-[(3-pyridylamino)methylidene]cyclohexane-1,3-dione | 3.18E+05 | 7.02E+05 | 2.92E+05 | 2.53E+05 | 5.29E+06 | 3.63E+05 | 3.66E+05 | 4.26E+05 | 1.60E+05 | 2.70E+05 | 0.28 |
| Com_12724_pos | 2-[(3S)-1-(1H-Indol-3-ylmethyl)-3-pyrrolidinyl]-1H-benzimidazole | 1.14E+07 | 2.13E+07 | 6.68E+05 | 6.08E+06 | 1.78E+06 | 8.22E+06 | 1.91E+06 | 7.04E+06 | 4.59E+06 | 1.49E+07 | 0.70 |
| Com_12736_pos | IKK | 9.14E+06 | 1.66E+07 | 1.04E+06 | 4.26E+06 | 2.06E+07 | 1.55E+07 | 4.39E+06 | 2.49E+07 | 1.52E+06 | 1.07E+07 | 0.86 |
| Com_12855_pos | VLH | 1.94E+06 | 2.67E+06 | 1.11E+06 | 4.99E+06 | 5.32E+06 | 2.11E+07 | 3.81E+06 | 3.26E+06 | 2.26E+06 | 5.30E+06 | 0.25 |
| Com_12859_pos | Palmitoylethanolamide | 2.69E+07 | 9.39E+06 | 1.66E+06 | 9.36E+06 | 6.40E+06 | 6.97E+06 | 1.10E+07 | 5.90E+06 | 5.48E+06 | 2.02E+06 | 0.56 |
| Com_12891_pos | 1-(2,4-diphenyl-2,3-dihydro-1H-1,5-benzodiazepin-1-yl)propan-1-one | 8.13E+06 | 2.08E+07 | 2.41E+07 | 5.76E+06 | 1.36E+07 | 1.08E+07 | 2.01E+07 | 6.83E+06 | 1.12E+07 | 8.72E+06 | 0.64 |
| Com_12937_pos | 4-(4-methoxyphenyl)-2-(methylthio)pyrimidine | 4.99E+06 | 7.11E+06 | 2.56E+06 | 2.08E+06 | 2.01E+07 | 2.04E+06 | 2.63E+06 | 2.82E+06 | 2.69E+06 | 2.17E+06 | 0.14 |
| Com_12951_neg | JWH 250 N-pentanoic acid metabolite | 1.87E+05 | 2.81E+05 | 4.84E+05 | 1.47E+06 | 3.67E+05 | 3.73E+06 | 2.95E+05 | 5.02E+05 | 1.41E+05 | 1.05E+06 | 0.61 |
| Com_1297_neg | LPG 15:0 | 3.24E+07 | 8.26E+07 | 2.43E+06 | 3.51E+07 | 1.46E+08 | 7.49E+07 | 9.09E+06 | 5.91E+07 | 8.09E+05 | 2.83E+07 | 0.53 |
| Com_1299_pos | 3,4-MDEA-d5 | 1.29E+08 | 1.07E+08 | 4.92E+07 | 8.22E+07 | 1.39E+08 | 4.46E+08 | 1.81E+08 | 2.11E+08 | 2.59E+08 | 1.84E+08 | 0.01 |
| Com_129_neg | Adrenic acid | 2.26E+09 | 1.77E+09 | 2.28E+09 | 2.17E+09 | 1.20E+09 | 1.83E+08 | 1.15E+09 | 6.00E+08 | 1.10E+09 | 1.87E+09 | 0.09 |
| Com_1300_pos | Di(2-ethylhexyl) phthalate | 9.78E+07 | 1.19E+08 | 1.19E+08 | 8.07E+07 | 7.68E+07 | 7.71E+07 | 1.24E+08 | 9.03E+07 | 7.69E+07 | 5.20E+08 | 0.49 |
| Com_13018_pos | Alanyltyrosine | 2.90E+06 | 1.67E+07 | 7.12E+05 | 2.57E+06 | 1.10E+07 | 4.48E+06 | 1.81E+06 | 2.48E+07 | 2.14E+06 | 7.11E+06 | 0.77 |
| Com_1301_neg | D-Sedoheptulose 7-phosphate | 6.92E+05 | 7.24E+05 | 1.90E+08 | 1.78E+06 | 3.70E+06 | 1.70E+06 | 1.73E+06 | 4.64E+06 | 1.07E+06 | 2.05E+06 | 0.60 |
| Com_1302_neg | D-(+)-Glucose | 1.68E+07 | 2.71E+07 | 1.40E+07 | 4.20E+07 | 1.11E+08 | 4.22E+07 | 9.87E+06 | 1.72E+08 | 8.60E+06 | 4.30E+07 | 0.97 |
| Com_1304_pos | N6,N6,N6-Trimethyl-L-lysine | 8.52E+07 | 7.14E+07 | 1.33E+08 | 3.46E+08 | 1.02E+08 | 2.56E+08 | 6.50E+08 | 3.93E+08 | 2.81E+08 | 2.52E+08 | 0.02 |
| Com_13051_pos | 4-acetyl-4-(ethoxycarbonyl)heptanedioic acid | 1.78E+07 | 1.17E+07 | 2.50E+07 | 5.76E+06 | 1.31E+07 | 9.73E+06 | 1.16E+07 | 5.86E+06 | 1.47E+07 | 6.33E+06 | 0.26 |
| Com_13068_pos | MKK | 2.70E+06 | 2.03E+07 | 4.93E+05 | 6.37E+05 | 6.03E+05 | 1.44E+06 | 3.71E+05 | 3.94E+05 | 5.69E+05 | 2.66E+06 | 0.42 |
| Com_1306_neg | 3-Phosphoglyceric acid | 1.74E+07 | 1.45E+07 | 1.23E+08 | 1.52E+08 | 5.78E+07 | 4.01E+07 | 8.72E+07 | 1.56E+08 | 2.01E+07 | 7.89E+07 | 0.71 |
| Com_1306_pos | D-Ala-D-Ala | 9.13E+07 | 1.07E+08 | 4.93E+08 | 4.83E+08 | 5.12E+07 | 1.02E+08 | 1.70E+07 | 6.38E+07 | 4.57E+08 | 4.83E+08 | 0.70 |
| Com_13094_pos | 17alpha-Ethinyl estradiol | 4.39E+06 | 1.03E+06 | 2.71E+06 | 5.16E+06 | 4.49E+06 | 3.26E+06 | 5.51E+06 | 3.41E+06 | 2.11E+07 | 4.03E+06 | 0.24 |
| Com_13134_pos | VPH | 1.02E+06 | 1.85E+06 | 6.50E+06 | 1.06E+06 | 3.29E+06 | 2.04E+07 | 8.64E+05 | 5.11E+06 | 3.79E+05 | 2.23E+06 | 0.89 |
| Com_13153_pos | Diosgenin | 7.30E+06 | 1.15E+07 | 2.46E+07 | 5.21E+06 | 8.05E+06 | 4.33E+06 | 5.65E+06 | 4.90E+06 | 5.06E+06 | 1.09E+07 | 0.14 |
| Com_13167_neg | Liquiritigenin | 2.34E+06 | 3.41E+06 | 4.49E+06 | 1.63E+06 | 2.55E+06 | 2.65E+06 | 3.56E+06 | 1.49E+06 | 2.47E+06 | 1.77E+06 | 0.46 |
| Com_13202_pos | N-lactoyl-phenylalanine | 8.61E+06 | 1.03E+07 | 2.45E+07 | 4.66E+06 | 3.81E+06 | 1.12E+07 | 4.90E+06 | 3.52E+06 | 8.26E+06 | 7.65E+06 | 0.57 |
| Com_1320_pos | D-Galactosamine | 2.06E+08 | 1.06E+08 | 3.29E+08 | 1.26E+08 | 4.20E+08 | 1.55E+08 | 5.85E+08 | 2.48E+08 | 1.02E+08 | 1.03E+08 | 0.83 |
| Com_13227_pos | 1-Methyl-2-[(3S)-1-(2-methylbenzyl)-3-pyrrolidinyl]-1H-benzimidazole | 1.43E+07 | 1.99E+07 | 1.26E+07 | 9.21E+06 | 1.04E+07 | 1.68E+07 | 9.84E+06 | 6.33E+06 | 1.60E+07 | 1.47E+07 | 0.78 |
| Com_13255_pos | 5-Methoxyindole-3-Carbaldehyde | 1.94E+06 | 3.54E+06 | 2.47E+06 | 3.78E+06 | 1.93E+07 | 1.73E+06 | 2.75E+06 | 2.54E+06 | 1.77E+06 | 4.88E+06 | 0.31 |
| Com_1328_pos | D-(+)-Camphor | 1.43E+08 | 9.24E+07 | 3.32E+08 | 4.77E+08 | 1.53E+08 | 1.13E+08 | 1.96E+08 | 2.18E+08 | 2.78E+08 | 2.31E+08 | 0.98 |
| Com_13354_pos | Pyridoxamine | 7.45E+06 | 5.36E+06 | 1.45E+07 | 1.05E+07 | 7.90E+06 | 2.58E+06 | 6.86E+06 | 4.98E+06 | 1.69E+07 | 2.32E+07 | 0.89 |
| Com_13355_pos | Isovanillic acid | 3.85E+06 | 1.23E+07 | 1.37E+07 | 1.36E+07 | 9.46E+06 | 1.63E+07 | 2.91E+07 | 2.35E+07 | 1.91E+07 | 1.85E+07 | 0.03 |
| Com_13364_pos | 5-(benzyloxy)-2-(hydroxymethyl)-1,4-dihydropyridin-4-one | 1.48E+06 | 6.53E+06 | 2.47E+06 | 1.57E+07 | 2.92E+06 | 1.98E+07 | 5.06E+06 | 1.60E+07 | 2.21E+06 | 3.28E+06 | 0.45 |
| Com_13369_pos | O-Acetyl-L-carnitine | 9.76E+06 | 1.96E+07 | 1.21E+07 | 1.75E+07 | 1.30E+07 | 1.03E+07 | 2.21E+07 | 1.74E+07 | 1.69E+07 | 1.06E+07 | 0.77 |
| Com_13382_pos | 3-Methoxyflavone | 1.29E+07 | 1.84E+07 | 5.03E+06 | 7.70E+06 | 5.31E+06 | 1.17E+07 | 1.97E+07 | 1.03E+07 | 2.23E+06 | 1.64E+07 | 0.81 |
| Com_1338_neg | Ethylmalonic acid | 9.90E+07 | 9.57E+07 | 1.29E+08 | 4.20E+07 | 1.52E+07 | 1.95E+07 | 4.79E+07 | 2.61E+07 | 7.07E+07 | 4.92E+07 | 0.37 |
| Com_133_neg | Docosatrienoic acid | 3.26E+08 | 1.75E+09 | 2.73E+08 | 8.46E+08 | 2.08E+08 | 5.60E+07 | 5.28E+08 | 7.32E+07 | 8.63E+08 | 1.77E+09 | 0.61 |
| Com_13415_neg | 2-{2-oxo-2-[(2-oxo-3-azepanyl)amino]ethoxy}acetic acid | 2.10E+06 | 3.28E+06 | 2.45E+06 | 1.03E+06 | 9.90E+05 | 9.74E+05 | 2.21E+06 | 2.02E+05 | 1.02E+06 | 1.58E+06 | 0.22 |
| Com_13417_pos | 5-amino-1-phenyl-1H-pyrazole-4-carbonitrile | 1.07E+06 | 1.12E+06 | 8.76E+05 | 3.08E+06 | 9.43E+05 | 1.18E+06 | 2.33E+06 | 5.36E+05 | 2.03E+07 | 6.13E+05 | 0.63 |
| Com_1342_pos | DL-Stachydrine | 7.51E+07 | 7.09E+07 | 1.94E+08 | 1.46E+08 | 5.72E+07 | 2.92E+08 | 3.84E+08 | 2.54E+08 | 4.43E+08 | 4.14E+07 | 0.14 |
| Com_13445_pos | 2,3-Dinor-TXB2 | 1.70E+07 | 1.94E+07 | 1.75E+07 | 1.29E+07 | 1.11E+07 | 1.88E+07 | 1.70E+07 | 1.14E+07 | 1.61E+07 | 1.31E+07 | 0.91 |
| Com_13451_pos | dihydrotachysterol | 6.65E+06 | 8.23E+06 | 1.83E+07 | 2.10E+07 | 6.08E+06 | 3.94E+06 | 7.15E+06 | 3.21E+06 | 1.13E+07 | 2.29E+07 | 0.47 |
| Com_13473_neg | (2S)-4-Oxo-2-phenyl-3,4-dihydro-2H-chromen-7-yl beta-D-glucopyranoside | 1.14E+06 | 7.80E+05 | 5.96E+05 | 1.99E+06 | 6.79E+05 | 3.20E+06 | 5.83E+06 | 2.90E+06 | 3.18E+06 | 3.12E+06 | 0.00 |
| Com_13482_neg | trans-2-Methyl-2-pentenoic Acid | 1.97E+06 | 1.42E+06 | 2.19E+06 | 1.99E+06 | 1.53E+06 | 3.39E+06 | 7.52E+06 | 2.62E+06 | 1.93E+06 | 1.14E+06 | 0.26 |
| Com_13511_neg | Cymarin | 3.38E+06 | 3.16E+06 | 1.03E+06 | 4.92E+06 | 1.96E+06 | 1.63E+06 | 1.98E+06 | 7.31E+05 | 1.95E+06 | 1.78E+06 | 0.16 |
| Com_13532_neg | D-Phenylalanine | 2.58E+06 | 3.22E+06 | 3.70E+06 | 2.34E+06 | 3.82E+06 | 2.28E+06 | 3.92E+06 | 4.69E+06 | 1.98E+06 | 1.03E+06 | 0.45 |
| Com_13621_pos | Deoxycorticosterone 21-glucoside | 1.35E+07 | 1.68E+07 | 1.36E+07 | 8.70E+06 | 1.27E+07 | 8.39E+06 | 2.82E+07 | 7.60E+06 | 1.23E+07 | 8.34E+06 | 0.66 |
| Com_13637_neg | PB-22 N-pentanoic acid-3-carboxyindole metabolite | 1.39E+06 | 2.88E+06 | 3.10E+06 | 3.12E+06 | 1.65E+06 | 1.80E+06 | 3.72E+06 | 1.24E+06 | 3.09E+06 | 1.61E+06 | 0.75 |
| Com_13641_pos | N-benzyl-N-isopropyl-N'-(4-isopropylphenyl)thiourea | 3.07E+06 | 3.58E+06 | 1.45E+06 | 2.85E+06 | 2.58E+06 | 1.92E+07 | 9.00E+06 | 2.71E+06 | 3.99E+06 | 1.55E+06 | 0.23 |
| Com_13662_pos | quinoxaline-2,3-dithiol | 1.17E+07 | 7.44E+06 | 1.19E+07 | 1.18E+07 | 1.77E+07 | 7.60E+06 | 2.80E+07 | 1.21E+07 | 6.31E+06 | 1.09E+07 | 0.90 |
| Com_13680_neg | Dehydrocholic acid | 9.71E+05 | 1.02E+06 | 2.77E+05 | 2.18E+06 | 1.80E+06 | 1.55E+06 | 1.72E+06 | 5.39E+06 | 2.91E+05 | 3.42E+06 | 0.43 |
| Com_13681_neg | Mycophenolic acid | 1.58E+06 | 2.41E+06 | 1.93E+06 | 2.68E+06 | 1.55E+06 | 3.31E+06 | 1.89E+06 | 1.29E+06 | 1.34E+06 | 2.30E+06 | 0.85 |
| Com_136_neg | (±)12(13)-DiHOME | 1.50E+09 | 5.33E+08 | 1.08E+09 | 6.54E+08 | 1.65E+09 | 2.25E+08 | 2.65E+09 | 2.31E+09 | 1.58E+09 | 4.51E+08 | 0.99 |
| Com_13711_neg | 3,4-Dihydroxybenzoate | 9.34E+05 | 2.72E+06 | 2.42E+06 | 8.57E+05 | 4.48E+06 | 1.42E+06 | 1.47E+06 | 1.41E+06 | 1.67E+06 | 1.08E+06 | 0.41 |
| Com_13729_neg | 2-Amino-3-(4-hydroxy-3-methoxyphenyl)propanoic acid | 6.11E+05 | 7.88E+05 | 1.07E+06 | 4.75E+06 | 1.03E+06 | 1.27E+06 | 4.99E+06 | 1.42E+06 | 1.83E+06 | 2.37E+06 | 0.24 |
| Com_1372_pos | Delta-Tridecalactone | 1.14E+08 | 1.63E+08 | 5.08E+08 | 4.44E+08 | 1.46E+08 | 9.57E+07 | 1.55E+08 | 1.87E+08 | 2.50E+08 | 2.27E+08 | 0.47 |
| Com_13778_pos | 6-(4-phenylpiperazino)hexanoic acid hydrochloride | 1.96E+06 | 2.41E+06 | 2.29E+07 | 8.23E+05 | 2.55E+06 | 1.33E+07 | 2.15E+06 | 7.28E+06 | 8.99E+05 | 2.09E+06 | 0.89 |
| Com_137_pos | Creatinine | 6.68E+07 | 1.28E+09 | 5.74E+09 | 1.17E+08 | 1.64E+09 | 6.35E+08 | 2.55E+09 | 3.62E+09 | 7.68E+07 | 3.64E+08 | 0.92 |
| Com_13825_pos | 8,8-dimethyl-2-phenyl-4H,8H-pyrano[2,3-h]chromen-4-one | 7.78E+05 | 8.62E+05 | 6.13E+05 | 9.58E+05 | 1.43E+06 | 2.31E+06 | 3.18E+06 | 2.25E+07 | 1.04E+06 | 1.27E+06 | 0.09 |
| Com_13830_pos | 1,3-Dihydro-1,3,3-trimethyl-2H-indol-2-ylidene acetaldehyde | 2.39E+07 | 1.32E+07 | 1.35E+07 | 6.65E+06 | 1.24E+07 | 6.49E+06 | 1.09E+07 | 6.67E+06 | 7.20E+06 | 8.76E+06 | 0.07 |
| Com_13836_pos | N1-(2-pyridylmethyl)-2-[3,5-di(trifluoromethyl)benzoyl]benzamide | 1.85E+06 | 1.06E+07 | 9.89E+05 | 1.24E+06 | 9.89E+05 | 1.53E+07 | 1.67E+06 | 2.24E+07 | 8.18E+05 | 1.10E+06 | 0.48 |
| Com_13841_neg | 5-heptyl-4-hydroxy-6H-pyrido[3,2,1-jk]carbazol-6-one | 8.57E+05 | 1.37E+06 | 6.17E+05 | 2.77E+06 | 2.50E+06 | 1.32E+06 | 3.79E+06 | 5.25E+06 | 7.38E+05 | 2.95E+06 | 0.33 |
| Com_13862_neg | Esculin | 2.19E+06 | 2.49E+06 | 4.91E+06 | 1.33E+06 | 4.38E+06 | 1.64E+06 | 4.20E+06 | 3.87E+06 | 1.72E+06 | 2.80E+06 | 0.90 |
| Com_13872_neg | N1-[4-(acetylamino)phenyl]-2,2-dimethylcyclopropane-1-carboxamide | 1.81E+05 | 3.10E+05 | 2.56E+05 | 4.64E+06 | 2.41E+05 | 1.72E+05 | 3.13E+05 | 5.02E+05 | 5.54E+05 | 2.43E+05 | 0.66 |
| Com_13873_pos | 2-{2-[(1-methyl-1H-pyrazol-5-yl)amino]-2-oxoethoxy}acetic acid | 1.77E+07 | 1.25E+07 | 2.26E+07 | 8.32E+06 | 1.77E+07 | 1.26E+07 | 2.19E+07 | 1.26E+07 | 1.69E+07 | 1.75E+07 | 0.76 |
| Com_13891_pos | WLH | 3.75E+06 | 7.38E+06 | 1.50E+06 | 4.98E+06 | 6.17E+06 | 4.50E+06 | 1.05E+07 | 2.23E+07 | 2.32E+06 | 9.24E+06 | 0.27 |
| Com_1391_pos | Hydroxyproline | 1.04E+08 | 1.14E+08 | 1.65E+08 | 1.43E+08 | 2.58E+08 | 9.96E+07 | 3.28E+08 | 4.95E+08 | 1.68E+08 | 1.92E+08 | 0.26 |
| Com_13936_pos | 6-amino-1-(2-methylphenyl)-1,2,3,4-tetrahydropyrimidine-2,4-dione | 1.32E+07 | 1.83E+07 | 1.04E+07 | 5.35E+06 | 1.34E+07 | 4.97E+06 | 8.68E+06 | 7.91E+06 | 9.56E+06 | 1.03E+07 | 0.21 |
| Com_13983_pos | 4-fluoro-N-(4-piperidinophenyl)benzenesulfonamide | 1.19E+06 | 6.66E+06 | 1.91E+06 | 2.02E+07 | 7.84E+05 | 2.45E+06 | 1.57E+06 | 1.12E+06 | 2.72E+06 | 1.01E+07 | 0.85 |
| Com_13_neg | Methylmalonic acid | 2.35E+09 | 3.69E+09 | 6.13E+09 | 6.12E+09 | 8.05E+09 | 1.05E+10 | 1.13E+10 | 3.02E+10 | 3.40E+09 | 7.21E+09 | 0.14 |
| Com_14001_pos | Celestolide | 1.79E+05 | 3.59E+05 | 3.43E+05 | 1.34E+07 | 2.73E+05 | 1.10E+07 | 2.68E+07 | 1.67E+07 | 1.62E+07 | 1.27E+07 | 0.01 |
| Com_14079_pos | methyl 3-(6-methylpyridin-2-yl)-2,2-diphenylpropanoate | 7.50E+06 | 1.33E+07 | 1.62E+07 | 8.27E+06 | 1.35E+07 | 7.69E+06 | 7.51E+06 | 2.17E+07 | 6.84E+06 | 1.16E+07 | 0.66 |
| Com_14085_pos | 1-[2-(2,5-dimethyl-1H-pyrrol-1-yl)-4-nitrophenyl]-1H-imidazole | 1.48E+07 | 1.64E+07 | 1.10E+07 | 1.45E+07 | 1.03E+07 | 1.77E+07 | 2.22E+07 | 1.33E+07 | 1.87E+07 | 1.33E+07 | 0.12 |
| Com_1408_neg | Glycocholic acid | 1.21E+07 | 1.34E+07 | 5.07E+07 | 5.24E+07 | 2.87E+07 | 3.56E+07 | 5.73E+06 | 9.60E+07 | 8.90E+07 | 4.99E+07 | 0.54 |
| Com_1410_pos | 5-Methyluridine | 7.52E+07 | 1.06E+08 | 1.54E+08 | 2.39E+08 | 2.44E+08 | 4.08E+08 | 1.63E+08 | 4.28E+08 | 1.45E+08 | 1.90E+08 | 0.18 |
| Com_14118_pos | Adipamide | 6.99E+06 | 7.44E+06 | 1.47E+06 | 5.07E+06 | 7.18E+06 | 1.80E+07 | 2.86E+06 | 1.39E+07 | 1.40E+06 | 8.87E+06 | 0.70 |
| Com_14120_pos | 5'-S-Methyl-5'-thioadenosine | 4.00E+06 | 3.45E+06 | 5.45E+06 | 1.98E+07 | 6.20E+06 | 8.57E+06 | 7.59E+06 | 6.04E+06 | 1.16E+07 | 1.22E+07 | 0.33 |
| Com_14122_pos | 6-Hydroxymelatonin | 3.66E+06 | 7.23E+06 | 8.45E+05 | 1.71E+07 | 8.92E+06 | 1.80E+07 | 3.40E+06 | 1.10E+07 | 3.36E+06 | 7.20E+06 | 0.62 |
| Com_14161_neg | Lysops 22:6 | 6.77E+05 | 6.41E+05 | 1.25E+06 | 9.65E+05 | 2.38E+06 | 3.07E+06 | 2.11E+06 | 1.53E+06 | 5.30E+05 | 1.05E+06 | 0.46 |
| Com_14190_pos | Nicotinuric Acid | 1.41E+07 | 1.35E+07 | 7.49E+06 | 3.07E+06 | 1.72E+07 | 6.11E+06 | 1.03E+07 | 1.33E+07 | 4.89E+06 | 5.17E+06 | 0.52 |
| Com_14233_pos | Ecgonine | 2.26E+06 | 3.88E+06 | 2.16E+07 | 1.92E+06 | 2.64E+06 | 2.70E+06 | 3.50E+06 | 5.36E+06 | 1.74E+06 | 2.23E+06 | 0.54 |
| Com_1426_pos | Acipimox | 6.68E+06 | 7.02E+06 | 8.85E+06 | 3.24E+08 | 6.34E+06 | 4.03E+08 | 2.27E+08 | 2.35E+08 | 2.39E+08 | 2.34E+08 | 0.02 |
| Com_14295_pos | ACar 16:5 | 4.20E+05 | 3.26E+05 | 2.15E+07 | 4.53E+05 | 3.95E+05 | 3.16E+05 | 3.84E+05 | 4.18E+05 | 3.91E+05 | 2.52E+06 | 0.62 |
| Com_14346_neg | 1-[2,6-di(tert-butylamino)-4-methyl-3-pyridyl]ethan-1-one | 1.94E+06 | 1.72E+06 | 2.94E+06 | 2.07E+06 | 2.62E+06 | 6.02E+05 | 1.58E+06 | 4.84E+06 | 9.15E+05 | 2.66E+06 | 0.46 |
| Com_14381_pos | NAT13-331713_POS | 5.83E+06 | 4.60E+06 | 1.21E+06 | 8.59E+05 | 4.90E+06 | 2.13E+06 | 2.56E+07 | 3.12E+06 | 6.98E+06 | 3.95E+06 | 0.26 |
| Com_1438_pos | Cyclopentyl fentanyl-d5 | 2.10E+08 | 1.89E+07 | 3.46E+07 | 4.39E+08 | 1.84E+07 | 2.33E+08 | 1.74E+07 | 5.69E+06 | 2.74E+07 | 5.76E+07 | 0.47 |
| Com_1439_neg | 13,14-Dihydro-15-keto Prostaglandin A2 | 1.22E+08 | 7.48E+07 | 3.35E+07 | 8.49E+07 | 1.23E+08 | 6.25E+07 | 6.03E+07 | 4.16E+07 | 5.37E+07 | 6.93E+07 | 0.24 |
| Com_14422_pos | 2-(Dimethylamino)Guanosine | 3.35E+06 | 1.05E+07 | 5.07E+06 | 5.52E+06 | 9.11E+06 | 6.13E+06 | 1.24E+07 | 2.09E+07 | 2.70E+06 | 6.76E+06 | 0.58 |
| Com_14439_pos | 3,5-di(2-furylmethylidene)tetrahydro-2H-pyran-4-one | 1.38E+07 | 8.87E+06 | 6.83E+06 | 1.78E+07 | 1.67E+07 | 1.63E+07 | 1.20E+07 | 1.45E+07 | 5.22E+06 | 1.42E+07 | 0.91 |
| Com_14458_pos | 4-methoxy-6-(prop-2-en-1-yl)-2H-1,3-benzodioxole | 5.46E+06 | 2.83E+06 | 2.37E+06 | 9.31E+06 | 2.88E+06 | 1.07E+07 | 2.39E+07 | 9.64E+06 | 1.79E+07 | 1.41E+07 | 0.00 |
| Com_144_neg | trans-10-Heptadecenoic acid | 8.17E+08 | 1.43E+09 | 2.27E+09 | 2.29E+09 | 1.14E+09 | 1.68E+08 | 1.37E+09 | 7.67E+08 | 1.22E+09 | 1.42E+09 | 0.22 |
| Com_144_pos | Genistein | 3.60E+08 | 2.88E+08 | 1.21E+08 | 1.40E+09 | 1.02E+09 | 4.26E+09 | 1.52E+09 | 3.16E+09 | 1.19E+08 | 4.30E+08 | 0.34 |
| Com_1454_neg | trans-Cinnamic acid | 1.30E+07 | 2.54E+07 | 2.88E+07 | 1.34E+08 | 3.74E+07 | 4.44E+07 | 4.62E+07 | 8.37E+07 | 2.31E+07 | 1.18E+08 | 0.37 |
| Com_14577_pos | 2-oxa-4-azatetracyclo[6.3.1.1~6,10~.0~1,5~]tridecan-3-one | 1.57E+07 | 6.87E+06 | 7.55E+06 | 6.67E+06 | 6.08E+06 | 1.71E+07 | 1.01E+07 | 1.01E+07 | 7.62E+06 | 1.18E+07 | 0.19 |
| Com_14633_pos | 2-Benzyl-5-[(3S)-1-isopropyl-3-pyrrolidinyl]-1,3,4-oxadiazole | 2.25E+06 | 5.03E+06 | 5.61E+05 | 3.79E+06 | 2.96E+06 | 7.63E+06 | 3.31E+06 | 2.04E+07 | 2.38E+06 | 2.87E+06 | 0.20 |
| Com_146_neg | D-(+)-Mannose | 4.53E+08 | 7.50E+08 | 1.77E+08 | 7.96E+08 | 1.99E+09 | 8.23E+08 | 1.75E+09 | 2.54E+09 | 2.12E+08 | 1.05E+09 | 0.48 |
| Com_14705_neg | Isorhamnetin | 1.25E+05 | 2.09E+05 | 5.89E+05 | 4.07E+06 | 5.02E+05 | 2.29E+06 | 4.14E+05 | 2.47E+06 | 3.54E+05 | 9.34E+05 | 0.40 |
| Com_14721_pos | YPH | 4.14E+06 | 5.51E+06 | 2.04E+07 | 7.80E+06 | 9.44E+06 | 3.60E+06 | 9.01E+06 | 1.43E+07 | 5.70E+06 | 5.85E+06 | 0.67 |
| Com_14736_neg | N1-[4-(cyanomethyl)phenyl]-4-chlorobenzamide | 2.74E+05 | 1.63E+05 | 4.07E+05 | 2.58E+05 | 8.27E+05 | 1.04E+06 | 5.13E+05 | 4.57E+06 | 1.38E+05 | 2.45E+05 | 0.40 |
| Com_1474_neg | Undecanoic acid | 5.54E+07 | 7.30E+07 | 1.46E+08 | 8.84E+07 | 1.02E+08 | 4.48E+07 | 7.84E+07 | 7.66E+07 | 8.39E+07 | 8.21E+07 | 0.33 |
| Com_14770_pos | (5-methyl-3-isoxazolyl)[4-(5-propyl-2-pyrimidinyl)piperazino]methanone | 1.02E+06 | 3.15E+06 | 1.75E+07 | 4.09E+06 | 4.58E+06 | 1.68E+07 | 2.67E+06 | 1.01E+07 | 4.60E+05 | 4.72E+06 | 0.99 |
| Com_14795_pos | Veratramine | 8.68E+06 | 1.66E+07 | 8.41E+06 | 6.00E+06 | 7.40E+06 | 6.70E+06 | 7.68E+06 | 3.23E+06 | 8.21E+06 | 3.69E+06 | 0.10 |
| Com_147_neg | Soyasaponin I | 1.68E+08 | 6.39E+08 | 3.00E+07 | 1.35E+08 | 1.64E+09 | 1.36E+09 | 7.15E+08 | 2.50E+09 | 4.33E+07 | 8.25E+07 | 0.65 |
| Com_14806_neg | 8,8'-dicarboxy-1,1'-binaphthalene | 9.57E+05 | 2.63E+06 | 2.12E+06 | 1.72E+06 | 1.83E+06 | 1.07E+06 | 6.54E+05 | 1.66E+06 | 2.31E+06 | 2.48E+06 | 0.56 |
| Com_14808_neg | D-Glyceric acid | 1.11E+06 | 4.57E+05 | 1.24E+06 | 5.27E+05 | 5.41E+05 | 6.86E+05 | 1.66E+06 | 4.86E+05 | 1.75E+05 | 4.49E+06 | 0.78 |
| Com_14825_pos | GKK | 8.94E+06 | 1.65E+07 | 1.11E+06 | 4.94E+06 | 1.19E+07 | 9.11E+06 | 4.52E+06 | 1.58E+07 | 2.48E+06 | 1.34E+07 | 0.79 |
| Com_14870_pos | N1-(2-thienylmethyl)-2-chlorobenzamide | 1.89E+07 | 1.10E+07 | 1.68E+07 | 3.94E+06 | 1.28E+07 | 1.09E+07 | 1.70E+07 | 7.59E+06 | 1.71E+07 | 1.33E+07 | 0.72 |
| Com_1487_neg | 2'-Deoxyinosine | 1.90E+07 | 3.96E+07 | 1.33E+08 | 7.38E+07 | 3.97E+07 | 2.02E+07 | 4.03E+07 | 2.71E+07 | 8.31E+07 | 1.06E+08 | 0.86 |
| Com_1489_neg | O-Phospho-L-serine | 1.03E+07 | 1.97E+07 | 1.54E+08 | 8.07E+07 | 2.94E+07 | 3.12E+07 | 2.94E+07 | 4.65E+07 | 5.06E+07 | 1.45E+08 | 0.63 |
| Com_1491_pos | Isoquinoline | 1.58E+08 | 2.68E+08 | 3.54E+07 | 4.65E+07 | 3.66E+08 | 6.84E+07 | 2.29E+08 | 1.80E+08 | 6.98E+07 | 1.42E+08 | 0.97 |
| Com_1495_neg | Fumaric acid | 5.06E+07 | 3.60E+07 | 1.60E+08 | 1.08E+08 | 3.95E+07 | 3.70E+07 | 9.32E+07 | 7.09E+07 | 5.68E+07 | 8.49E+06 | 0.39 |
| Com_14963_neg | 3-benzyl-1-butyl-4-hydroxy-1,2-dihydroquinolin-2-one | 7.32E+05 | 8.99E+05 | 4.57E+05 | 3.90E+06 | 1.37E+06 | 2.52E+06 | 2.70E+06 | 3.36E+06 | 1.10E+06 | 2.29E+06 | 0.13 |
| Com_14972_pos | L-Cystathionine | 4.27E+06 | 1.68E+06 | 1.18E+07 | 8.52E+06 | 3.79E+06 | 8.19E+06 | 4.01E+06 | 1.97E+07 | 5.65E+06 | 5.44E+06 | 0.39 |
| Com_1497_pos | LPE 12:0 | 1.92E+07 | 9.84E+07 | 5.27E+06 | 2.59E+07 | 1.32E+08 | 7.86E+07 | 1.30E+08 | 4.55E+08 | 1.00E+07 | 5.26E+06 | 0.71 |
| Com_14_pos | Fenpropimorph | 3.73E+10 | 9.03E+09 | 5.28E+10 | 3.13E+10 | 3.67E+10 | 2.20E+10 | 5.33E+10 | 4.44E+10 | 2.82E+10 | 2.80E+10 | 0.70 |
| Com_15000_pos | gamma-Glutamylcysteine | 5.37E+05 | 5.48E+05 | 6.00E+05 | 2.68E+06 | 5.81E+05 | 1.64E+07 | 7.43E+05 | 8.68E+05 | 5.59E+05 | 9.61E+05 | 0.42 |
| Com_1500_neg | PG (4:0/18:1) | 2.43E+06 | 3.47E+07 | 5.23E+06 | 4.34E+07 | 6.40E+06 | 8.31E+05 | 4.33E+06 | 1.44E+08 | 3.96E+06 | 3.41E+06 | 0.59 |
| Com_15021_pos | Cadaverine | 4.10E+06 | 8.67E+06 | 1.35E+07 | 5.00E+06 | 1.30E+07 | 1.63E+07 | 1.95E+07 | 1.54E+07 | 7.05E+05 | 3.53E+06 | 0.79 |
| Com_15029_pos | 5α-Dihydrotestosterone glucuronide | 1.87E+06 | 1.84E+06 | 1.15E+06 | 1.12E+06 | 1.57E+07 | 1.88E+06 | 1.57E+06 | 9.53E+05 | 8.70E+05 | 1.04E+06 | 0.25 |
| Com_1507_neg | L-Serine | 5.01E+07 | 7.50E+07 | 1.02E+08 | 1.26E+08 | 1.07E+08 | 6.83E+07 | 1.78E+08 | 1.00E+08 | 4.26E+07 | 1.42E+08 | 0.82 |
| Com_15122_pos | Targinine | 3.65E+06 | 3.57E+06 | 1.97E+07 | 6.75E+06 | 6.72E+06 | 4.82E+06 | 5.54E+06 | 1.06E+07 | 2.47E+06 | 5.73E+06 | 0.60 |
| Com_15150_pos | 1-Phenyl-3-methyl-5-pyrazolone | 2.11E+06 | 2.94E+06 | 2.37E+06 | 2.36E+06 | 3.86E+06 | 7.89E+06 | 5.04E+06 | 1.94E+07 | 1.76E+06 | 3.37E+06 | 0.16 |
| Com_1517_pos | Bicine | 4.74E+08 | 1.18E+08 | 1.55E+08 | 8.21E+07 | 6.31E+07 | 7.53E+07 | 9.16E+07 | 6.50E+07 | 9.77E+07 | 9.95E+07 | 0.26 |
| Com_1518_neg | (+/-)9,10-dihydroxy-12Z-octadecenoic acid | 6.10E+07 | 4.96E+07 | 1.17E+08 | 3.66E+07 | 8.38E+07 | 4.12E+07 | 1.17E+08 | 5.51E+07 | 8.13E+07 | 7.21E+07 | 0.80 |
| Com_15201_pos | ALK | 2.98E+06 | 4.81E+06 | 1.36E+06 | 1.31E+07 | 1.41E+07 | 8.77E+06 | 9.05E+06 | 1.93E+07 | 2.25E+06 | 7.21E+06 | 0.52 |
| Com_15220_pos | QNK | 2.42E+06 | 8.23E+06 | 3.61E+06 | 1.18E+07 | 7.96E+06 | 1.61E+07 | 5.69E+06 | 1.54E+07 | 1.96E+06 | 6.80E+06 | 0.69 |
| Com_15234_pos | Tyrosylalanine | 3.65E+06 | 4.28E+06 | 3.08E+06 | 1.19E+07 | 3.32E+06 | 1.60E+07 | 9.64E+06 | 2.73E+06 | 2.46E+06 | 6.80E+06 | 0.57 |
| Com_1524_pos | Etiocholanolone | 7.76E+07 | 6.18E+07 | 8.72E+07 | 4.06E+08 | 1.26E+08 | 6.83E+07 | 1.26E+08 | 7.20E+07 | 1.08E+08 | 7.12E+07 | 0.44 |
| Com_15255_pos | 3b,7b-Dihydroxy-5-androsten-17-one | 1.69E+07 | 1.58E+07 | 1.16E+07 | 9.08E+06 | 1.10E+07 | 1.21E+07 | 9.99E+06 | 8.78E+06 | 8.58E+06 | 1.50E+07 | 0.32 |
| Com_15260_pos | PQH | 1.12E+07 | 1.58E+07 | 1.39E+07 | 7.80E+06 | 1.51E+07 | 7.90E+06 | 7.04E+06 | 4.71E+06 | 1.08E+07 | 1.24E+07 | 0.09 |
| Com_15281_pos | (4E)-1,7-bis(3,4-dihydroxyphenyl)hept-4-en-3-one | 2.03E+07 | 8.78E+06 | 1.52E+06 | 4.92E+06 | 4.21E+06 | 6.13E+06 | 3.78E+06 | 2.59E+06 | 5.27E+06 | 5.41E+06 | 0.62 |
| Com_1529_neg | Hexanoic acid | 8.65E+07 | 6.62E+07 | 6.63E+07 | 5.20E+07 | 6.69E+07 | 3.34E+07 | 8.53E+07 | 4.52E+07 | 8.05E+07 | 9.83E+07 | 0.83 |
| Com_15300_pos | 7-hydroxy-6-nitro-2,3-dihydro-1H,5H-pyrido[3,2,1-ij]quinolin-5-one | 5.43E+05 | 4.54E+05 | 5.93E+05 | 5.68E+05 | 5.08E+05 | 6.03E+05 | 6.14E+05 | 9.66E+05 | 1.64E+07 | 4.96E+06 | 0.12 |
| Com_15333_neg | N-(2-cyanoacetyl)-N'-(2-methylphenyl)urea | 1.36E+06 | 1.37E+06 | 4.53E+06 | 5.27E+05 | 3.61E+05 | 2.88E+05 | 4.77E+05 | 5.75E+05 | 5.45E+05 | 6.72E+05 | 0.15 |
| Com_1534_neg | (+/-)12(13)-DiHOME | 6.03E+07 | 4.93E+07 | 1.16E+08 | 3.61E+07 | 8.29E+07 | 4.09E+07 | 1.16E+08 | 5.46E+07 | 8.04E+07 | 7.14E+07 | 0.79 |
| Com_15351_neg | 1,3-Dimethyluric Acid | 1.68E+06 | 1.86E+06 | 3.03E+06 | 2.42E+06 | 2.56E+06 | 2.38E+06 | 5.21E+06 | 2.57E+06 | 2.10E+06 | 3.37E+06 | 0.21 |
| Com_15394_neg | LPA 22:5 | 5.42E+05 | 5.87E+05 | 4.39E+05 | 5.27E+05 | 3.49E+05 | 2.50E+06 | 4.97E+05 | 4.45E+05 | 2.27E+05 | 3.99E+05 | 0.76 |
| Com_15404_pos | 4-oxododecanedioic acid | 1.13E+07 | 1.02E+07 | 1.91E+07 | 3.40E+05 | 9.59E+06 | 6.69E+06 | 1.41E+07 | 6.38E+06 | 1.43E+07 | 1.17E+07 | 0.51 |
| Com_15407_neg | Catechin | 1.96E+06 | 1.52E+06 | 4.43E+06 | 1.20E+06 | 2.42E+06 | 1.15E+06 | 1.79E+06 | 1.05E+06 | 2.31E+06 | 1.27E+06 | 0.22 |
| Com_15421_neg | Oxaloacetic acid | 3.97E+05 | 4.77E+05 | 1.12E+06 | 1.17E+06 | 1.11E+06 | 1.20E+06 | 3.29E+06 | 3.23E+06 | 2.31E+06 | 1.62E+06 | 0.01 |
| Com_1542_neg | Lysopc 18:2 | 3.68E+06 | 8.11E+07 | 5.32E+06 | 4.76E+06 | 1.54E+07 | 4.97E+06 | 6.19E+06 | 4.86E+07 | 2.87E+06 | 3.77E+06 | 0.62 |
| Com_15491_pos | 3-(3,4-dimethoxyphenyl)-1-(2-hydroxy-4,6-dimethoxyphenyl)propan-1-one | 3.70E+06 | 3.54E+06 | 1.50E+06 | 2.85E+06 | 2.94E+06 | 3.41E+06 | 7.31E+06 | 3.43E+06 | 1.60E+07 | 3.07E+06 | 0.12 |
| Com_15513_pos | gamma-Tocopherol | 5.56E+06 | 8.53E+06 | 1.57E+07 | 1.36E+07 | 7.80E+06 | 5.33E+06 | 3.75E+06 | 2.19E+06 | 7.06E+06 | 1.81E+07 | 0.23 |
| Com_1553_pos | 9-Oxo-ODE | 2.47E+08 | 2.04E+08 | 3.41E+08 | 1.66E+08 | 1.52E+08 | 1.40E+08 | 1.80E+08 | 1.44E+08 | 3.74E+08 | 2.19E+08 | 0.75 |
| Com_15541_neg | N-Acetyl-D-tryptophan | 7.49E+05 | 1.20E+06 | 1.57E+06 | 6.62E+05 | 1.31E+06 | 2.45E+06 | 1.47E+06 | 1.58E+06 | 7.27E+05 | 9.41E+05 | 0.41 |
| Com_15565_pos | Flavin mononucleotide (FMN) | 5.88E+05 | 1.59E+06 | 1.87E+07 | 8.57E+05 | 3.13E+06 | 5.89E+06 | 1.10E+07 | 1.74E+07 | 6.87E+05 | 5.04E+05 | 0.67 |
| Com_1556_pos | Indole-3-lactic acid | 1.40E+07 | 2.46E+07 | 4.40E+08 | 6.17E+07 | 9.07E+07 | 1.80E+08 | 7.92E+07 | 2.48E+08 | 1.77E+07 | 2.22E+07 | 0.91 |
| Com_15570_pos | 4-(tert-butyl)-3-(4-chlorophenethyl)-2,3-dihydro-1,3-thiazole-2-thione | 1.08E+06 | 8.52E+05 | 1.46E+06 | 7.37E+06 | 1.03E+06 | 1.54E+07 | 1.61E+07 | 6.90E+06 | 7.79E+06 | 7.57E+06 | 0.01 |
| Com_15589_pos | GQH | 1.04E+06 | 5.97E+06 | 1.87E+07 | 2.60E+06 | 9.09E+06 | 6.94E+06 | 1.63E+07 | 1.84E+07 | 1.24E+06 | 1.46E+06 | 0.93 |
| Com_155_pos | Valine | 9.37E+08 | 1.83E+09 | 1.31E+09 | 3.97E+09 | 1.35E+09 | 3.18E+09 | 9.72E+08 | 4.87E+09 | 1.47E+09 | 2.06E+09 | 0.50 |
| Com_15659_pos | S-Methyl-L-cysteine | 3.23E+06 | 3.28E+06 | 6.26E+06 | 1.68E+07 | 4.60E+06 | 1.18E+07 | 3.74E+06 | 8.90E+06 | 4.92E+06 | 5.80E+06 | 0.68 |
| Com_15671_neg | 2-[2-(4-benzhydrylpiperazino)-2-oxoethoxy]acetic acid | 1.60E+06 | 2.02E+06 | 2.77E+05 | 4.51E+05 | 2.54E+06 | 1.42E+06 | 3.44E+06 | 2.15E+06 | 2.22E+06 | 1.13E+06 | 0.23 |
| Com_1567_neg | 4-Hydroxybenzoic acid | 2.12E+06 | 7.88E+07 | 4.73E+06 | 2.58E+06 | 3.15E+06 | 1.69E+06 | 4.94E+06 | 3.93E+06 | 1.91E+06 | 3.77E+06 | 0.39 |
| Com_15725_pos | L-5-Hydroxytryptophan | 2.09E+06 | 4.17E+06 | 1.84E+07 | 2.69E+06 | 4.22E+06 | 4.45E+05 | 2.37E+06 | 3.01E+06 | 1.57E+06 | 2.65E+06 | 0.09 |
| Com_15743_pos | Methylbenzethonium chloride | 7.28E+06 | 4.88E+06 | 5.46E+06 | 1.67E+07 | 4.49E+06 | 7.09E+06 | 7.65E+06 | 4.60E+06 | 1.39E+07 | 7.84E+06 | 0.69 |
| Com_15748_pos | RNK | 5.66E+06 | 1.09E+06 | 1.84E+07 | 1.07E+07 | 4.30E+06 | 3.42E+06 | 1.25E+07 | 4.85E+06 | 5.94E+06 | 6.40E+06 | 0.88 |
| Com_15759_pos | Morphine | 4.47E+06 | 4.45E+06 | 6.34E+05 | 1.40E+06 | 1.46E+07 | 4.92E+06 | 3.26E+05 | 4.06E+05 | 1.46E+07 | 9.18E+06 | 0.83 |
| Com_1575_neg | FAHFA (18:1/20:3) | 1.82E+07 | 5.67E+07 | 5.43E+07 | 1.19E+08 | 3.76E+07 | 1.97E+06 | 5.79E+07 | 2.20E+07 | 6.73E+07 | 3.52E+07 | 0.33 |
| Com_15764_pos | IMK | 1.29E+07 | 5.22E+06 | 8.84E+05 | 3.67E+06 | 7.03E+06 | 1.51E+07 | 7.07E+06 | 6.78E+06 | 2.04E+06 | 7.39E+06 | 0.50 |
| Com_15785_pos | Oxohongdenafil | 1.55E+06 | 1.22E+06 | 1.41E+06 | 1.15E+06 | 1.22E+06 | 1.51E+07 | 1.23E+06 | 4.86E+06 | 1.03E+06 | 1.21E+06 | 0.26 |
| Com_15825_neg | 4-[(3,4-dimethoxyphenethyl)amino]-4-oxobutanoic acid | 1.35E+05 | 6.52E+05 | 3.95E+05 | 1.96E+06 | 3.68E+05 | 1.82E+06 | 3.51E+06 | 1.51E+06 | 2.18E+06 | 1.86E+06 | 0.02 |
| Com_1582_neg | LPE 18:3 | 5.13E+06 | 8.23E+06 | 3.69E+06 | 3.27E+06 | 1.12E+08 | 5.79E+06 | 8.33E+05 | 8.96E+07 | 1.02E+06 | 1.95E+06 | 0.46 |
| Com_15835_pos | N-Acetylserotonin | 5.88E+06 | 1.02E+07 | 6.82E+06 | 6.01E+06 | 1.44E+07 | 8.24E+06 | 7.28E+06 | 5.02E+06 | 6.56E+06 | 5.05E+06 | 0.25 |
| Com_15836_pos | RMH | 1.37E+06 | 4.11E+06 | 1.02E+06 | 7.10E+06 | 7.56E+05 | 2.63E+06 | 1.04E+07 | 6.07E+06 | 2.48E+06 | 1.75E+07 | 0.09 |
| Com_1583_neg | 1a,1b-Dihomo prostaglandin E1 | 4.44E+07 | 4.79E+07 | 5.46E+07 | 4.68E+07 | 3.00E+07 | 1.34E+07 | 3.30E+07 | 7.66E+06 | 7.64E+07 | 6.39E+07 | 0.37 |
| Com_1583_pos | 16-Hydroxyhexadecanoic acid | 9.22E+07 | 5.27E+07 | 4.33E+08 | 1.63E+08 | 9.18E+07 | 1.29E+08 | 1.20E+08 | 2.53E+08 | 1.41E+08 | 2.88E+08 | 0.45 |
| Com_15856_pos | Artemisinin | 1.06E+06 | 4.21E+05 | 1.13E+06 | 7.78E+05 | 3.79E+05 | 3.47E+05 | 2.19E+07 | 2.22E+06 | 1.02E+06 | 1.23E+06 | 0.23 |
| Com_15869_pos | 2-(2-acetyl-3,5-dihydroxyphenyl)acetic acid | 1.54E+06 | 2.13E+06 | 1.89E+06 | 1.01E+07 | 2.75E+06 | 8.86E+06 | 2.19E+07 | 1.09E+07 | 1.27E+07 | 1.51E+07 | 0.01 |
| Com_1588_pos | ethyl 5-methoxy-2-methyl-1-phenyl-1H-indole-3-carboxylate | 2.95E+08 | 3.53E+08 | 3.72E+08 | 2.20E+08 | 1.84E+08 | 1.69E+08 | 1.24E+08 | 6.60E+07 | 3.35E+08 | 2.60E+08 | 0.16 |
| Com_1589_pos | delta-Tocopherol | 2.57E+08 | 3.52E+08 | 1.74E+08 | 7.71E+07 | 9.74E+07 | 7.69E+07 | 1.32E+08 | 3.06E+07 | 1.70E+08 | 1.61E+08 | 0.26 |
| Com_15909_neg | Glutathione (reduced) | 1.57E+05 | 1.24E+05 | 2.06E+05 | 1.72E+05 | 2.26E+06 | 2.31E+06 | 2.57E+05 | 8.66E+05 | 1.41E+05 | 1.89E+05 | 0.58 |
| Com_1590_pos | 4-(methylthio)-6-phenyl-2-(3-pyridyl)pyrimidine-5-carbonitrile | 4.48E+07 | 1.22E+08 | 3.68E+06 | 5.29E+06 | 1.20E+07 | 7.66E+07 | 1.95E+07 | 4.25E+08 | 9.26E+06 | 1.35E+07 | 0.42 |
| Com_15923_neg | 5-Methyl-dl-tryptophan | 1.63E+06 | 2.19E+06 | 1.50E+06 | 9.00E+05 | 2.32E+06 | 1.03E+06 | 1.82E+06 | 7.10E+05 | 7.94E+05 | 7.01E+05 | 0.06 |
| Com_15973_pos | Troxerutin | 9.56E+06 | 8.18E+06 | 1.44E+07 | 1.20E+07 | 9.27E+06 | 9.05E+06 | 2.17E+07 | 8.34E+06 | 8.82E+06 | 8.78E+06 | 0.99 |
| Com_15978_pos | 9-chloro-1-fluoro-12H-benzo[4,5][1,3]thiazolo[2,3-b]quinazolin-12-one | 4.77E+06 | 3.73E+06 | 7.91E+06 | 4.76E+06 | 6.16E+06 | 1.48E+07 | 6.05E+06 | 3.09E+06 | 7.08E+06 | 3.90E+06 | 0.70 |
| Com_1597_neg | 4-Nitrocatechol | 4.42E+07 | 4.15E+07 | 6.14E+07 | 1.17E+08 | 5.71E+07 | 1.83E+07 | 1.01E+08 | 3.52E+07 | 7.57E+07 | 6.63E+07 | 0.66 |
| Com_16032_neg | 1,3-diazaspiro[4.5]decane-2,4-dione | 7.66E+05 | 9.86E+05 | 4.05E+06 | 1.27E+06 | 1.56E+06 | 7.10E+05 | 2.19E+06 | 1.38E+06 | 9.05E+05 | 1.64E+06 | 0.72 |
| Com_16071_neg | Prostaglandin A2 | 1.16E+06 | 2.13E+06 | 2.18E+05 | 7.35E+05 | 2.13E+06 | 6.62E+05 | 7.06E+05 | 2.05E+05 | 6.50E+05 | 1.21E+06 | 0.38 |
| Com_16078_pos | PC (7:0/7:0) | 1.37E+06 | 1.73E+06 | 1.80E+06 | 3.45E+06 | 2.80E+06 | 1.38E+07 | 1.82E+06 | 1.75E+07 | 1.45E+06 | 1.91E+06 | 0.29 |
| Com_16094_neg | 15(R)-Lipoxin A4 | 2.91E+06 | 7.85E+05 | 4.50E+05 | 6.10E+05 | 2.14E+05 | 4.05E+05 | 7.42E+05 | 1.94E+05 | 1.06E+06 | 9.74E+05 | 0.77 |
| Com_160_neg | D-α-Hydroxyglutaric acid | 1.39E+08 | 1.14E+08 | 5.66E+08 | 1.54E+09 | 2.99E+08 | 1.35E+09 | 1.06E+09 | 7.51E+08 | 3.81E+08 | 7.00E+08 | 0.16 |
| Com_16105_pos | 7Z, 10Z, 13Z, 16Z, 19Z-docosapentaenoic acid | 9.05E+06 | 9.85E+06 | 1.61E+07 | 1.50E+07 | 7.09E+06 | 6.58E+06 | 1.68E+07 | 6.17E+06 | 1.18E+07 | 1.70E+07 | 0.94 |
| Com_16125_pos | NNK | 1.20E+07 | 8.13E+06 | 4.60E+06 | 4.03E+06 | 3.69E+06 | 1.28E+07 | 2.91E+06 | 9.40E+06 | 1.20E+07 | 3.98E+06 | 0.64 |
| Com_16145_neg | N-[4-(diethylamino)phenyl]-N'-phenylurea | 4.79E+05 | 3.08E+05 | 3.25E+05 | 7.70E+05 | 4.48E+05 | 2.22E+06 | 1.36E+06 | 7.55E+05 | 3.85E+05 | 2.92E+05 | 0.24 |
| Com_16154_pos | (1R,2R)-trans-N-Boc-1,2-cyclohexanediamine | 7.77E+05 | 1.49E+06 | 1.73E+06 | 3.63E+06 | 2.67E+06 | 1.17E+06 | 2.13E+07 | 1.45E+06 | 1.10E+06 | 1.24E+06 | 0.78 |
| Com_1618_neg | (+/-)9-HpODE | 4.83E+07 | 3.42E+07 | 8.11E+07 | 3.79E+07 | 4.37E+07 | 3.13E+07 | 1.78E+08 | 6.04E+07 | 4.66E+07 | 4.53E+07 | 0.51 |
| Com_16193_pos | SNK | 1.14E+07 | 1.43E+07 | 1.26E+07 | 7.88E+06 | 1.23E+07 | 6.48E+06 | 8.51E+06 | 7.75E+06 | 9.48E+06 | 9.53E+06 | 0.03 |
| Com_16201_pos | Spermine | 3.77E+06 | 1.32E+07 | 3.11E+06 | 3.08E+06 | 1.10E+07 | 5.58E+06 | 3.76E+06 | 1.73E+07 | 6.07E+06 | 4.53E+06 | 0.77 |
| Com_16239_pos | 3-(methylsulfonyl)-2H-chromen-2-one | 1.38E+06 | 1.64E+06 | 1.21E+06 | 1.75E+06 | 3.15E+06 | 5.82E+06 | 6.36E+06 | 1.73E+07 | 3.13E+06 | 5.09E+06 | 0.01 |
| Com_162_neg | L-Glutamic acid monosodium salt | 6.03E+08 | 5.61E+08 | 1.55E+09 | 1.39E+09 | 5.62E+08 | 4.50E+08 | 1.07E+09 | 5.54E+08 | 1.25E+09 | 1.61E+09 | 0.88 |
| Com_16302_pos | N-Oleoyl Glycine | 3.79E+06 | 4.56E+06 | 1.74E+07 | 7.58E+06 | 9.27E+06 | 5.64E+06 | 1.07E+07 | 1.39E+07 | 3.55E+06 | 5.72E+06 | 0.91 |
| Com_16344_pos | LPC 16:3 | 4.31E+06 | 4.09E+06 | 6.16E+06 | 3.67E+06 | 8.94E+06 | 1.42E+07 | 4.64E+06 | 1.64E+06 | 5.22E+06 | 4.71E+06 | 0.88 |
| Com_16348_pos | Guanidineacetic acid | 3.05E+06 | 5.13E+06 | 8.94E+06 | 4.16E+06 | 6.93E+06 | 7.31E+06 | 3.93E+06 | 1.71E+07 | 4.15E+06 | 3.55E+06 | 0.75 |
| Com_1634_pos | 6-Ketoprostaglandin F1α | 2.96E+08 | 3.24E+08 | 3.75E+08 | 9.59E+07 | 3.31E+08 | 2.21E+08 | 2.65E+08 | 2.05E+08 | 7.30E+07 | 1.20E+08 | 0.20 |
| Com_16352_pos | 1-(3-methoxy-2-nitrostyryl)pyrrolidine | 3.80E+06 | 3.62E+06 | 9.18E+05 | 1.36E+07 | 1.37E+07 | 1.83E+06 | 1.61E+06 | 1.08E+06 | 4.25E+06 | 7.77E+05 | 0.11 |
| Com_1636_neg | Tretinoin | 1.02E+08 | 7.51E+07 | 1.40E+08 | 1.06E+08 | 9.28E+07 | 7.38E+07 | 1.55E+08 | 1.15E+08 | 6.68E+07 | 1.19E+08 | 0.99 |
| Com_1638_pos | trans,trans-2,4-Heptadienal | 2.54E+08 | 3.31E+08 | 4.15E+08 | 2.13E+08 | 2.57E+08 | 1.50E+08 | 3.40E+08 | 2.87E+08 | 1.83E+08 | 1.94E+08 | 0.21 |
| Com_16410_pos | 7-(2-aminophenyl)heptanoic acid | 9.94E+06 | 8.45E+06 | 1.17E+07 | 5.38E+06 | 1.36E+07 | 3.07E+06 | 1.05E+07 | 1.41E+07 | 3.49E+06 | 1.18E+07 | 0.48 |
| Com_16441_neg | ethyl 3-(1,3-benzodioxol-5-ylamino)-2-cyanoacrylate | 1.24E+06 | 1.56E+06 | 2.29E+06 | 1.04E+06 | 2.85E+06 | 1.28E+06 | 2.06E+06 | 2.21E+06 | 1.41E+06 | 2.32E+06 | 0.75 |
| Com_16463_pos | Daidzin | 7.38E+06 | 5.20E+06 | 8.15E+05 | 9.38E+05 | 7.59E+06 | 5.84E+06 | 2.14E+06 | 1.68E+07 | 1.49E+06 | 1.30E+06 | 0.87 |
| Com_1646_pos | ACar 15:2 | 4.60E+06 | 2.78E+06 | 4.14E+08 | 1.41E+06 | 2.09E+06 | 1.06E+08 | 1.31E+06 | 1.76E+07 | 1.24E+06 | 1.66E+06 | 0.87 |
| Com_16473_pos | Deoxyadenosine | 1.25E+06 | 1.57E+06 | 3.45E+06 | 9.62E+05 | 6.39E+06 | 6.41E+06 | 1.49E+06 | 2.93E+06 | 1.46E+06 | 1.64E+07 | 0.37 |
| Com_16484_pos | DL-Panthenol | 4.13E+06 | 7.10E+06 | 9.22E+06 | 2.77E+06 | 1.35E+07 | 2.87E+06 | 8.37E+06 | 4.77E+06 | 6.70E+06 | 2.24E+06 | 0.37 |
| Com_16485_pos | N-(2,3-dihydro-1,4-benzodioxin-6-yl)-2,5-dimethyl-3-furamide | 1.21E+07 | 1.21E+07 | 1.16E+07 | 1.71E+06 | 1.04E+07 | 7.31E+06 | 1.01E+07 | 5.56E+06 | 1.45E+07 | 1.23E+07 | 0.69 |
| Com_1648_neg | Oxoadipic Acid | 6.37E+06 | 8.60E+06 | 2.52E+07 | 8.30E+06 | 7.79E+06 | 3.51E+07 | 2.07E+07 | 1.97E+07 | 2.01E+07 | 1.27E+08 | 0.03 |
| Com_164_neg | Picolinic acid | 8.45E+08 | 1.21E+09 | 2.40E+09 | 1.12E+09 | 1.54E+09 | 5.66E+08 | 1.73E+09 | 1.00E+09 | 1.08E+09 | 1.59E+09 | 0.51 |
| Com_16503_pos | 3-Hydroxyphenylacetic acid | 1.30E+07 | 1.32E+07 | 1.16E+07 | 1.16E+07 | 1.35E+07 | 1.30E+07 | 9.92E+06 | 6.13E+06 | 1.29E+07 | 8.97E+06 | 0.16 |
| Com_16519_pos | 3,5,7-trihydroxy-2-phenyl-4H-chromen-4-one | 6.82E+06 | 5.10E+06 | 1.70E+07 | 6.49E+06 | 7.62E+06 | 7.66E+06 | 5.16E+06 | 5.38E+06 | 8.38E+06 | 6.00E+06 | 0.41 |
| Com_16554_pos | LysoPE 18:2 | 7.63E+06 | 5.79E+06 | 1.69E+07 | 1.17E+07 | 8.23E+06 | 1.05E+07 | 1.55E+06 | 3.68E+06 | 1.13E+06 | 5.42E+06 | 0.06 |
| Com_16581_pos | 6β-Naltrexol | 7.50E+06 | 1.04E+07 | 1.23E+07 | 1.20E+07 | 8.98E+06 | 1.23E+07 | 1.05E+07 | 8.34E+06 | 1.43E+07 | 1.27E+07 | 0.36 |
| Com_16587_pos | S-Adenosyl-L-methionine | 1.77E+07 | 7.85E+06 | 1.21E+07 | 5.14E+06 | 9.43E+06 | 5.85E+06 | 1.03E+07 | 8.66E+06 | 8.99E+06 | 4.37E+05 | 0.30 |
| Com_16602_neg | Lysope 18:1 | 1.25E+06 | 1.67E+06 | 3.65E+06 | 1.70E+06 | 1.09E+06 | 1.55E+06 | 8.94E+05 | 1.40E+06 | 6.30E+05 | 1.13E+06 | 0.12 |
| Com_16616_pos | KNK | 6.05E+06 | 1.18E+07 | 1.07E+06 | 5.26E+06 | 1.03E+07 | 9.80E+06 | 3.82E+06 | 1.66E+07 | 1.48E+06 | 7.59E+06 | 0.86 |
| Com_1664_neg | 2-Hydroxy-2-methylbutanoic acid | 2.82E+07 | 7.35E+07 | 2.31E+07 | 9.64E+07 | 2.82E+07 | 3.06E+07 | 8.50E+07 | 3.79E+07 | 2.95E+07 | 8.51E+07 | 0.74 |
| Com_1666_pos | Methyl beta-D-galactopyranoside | 1.01E+07 | 3.21E+07 | 1.16E+07 | 6.80E+07 | 1.75E+07 | 1.54E+08 | 1.01E+08 | 4.01E+08 | 1.62E+07 | 1.70E+08 | 0.04 |
| Com_16686_pos | 3-{[(3S)-3-(1,3-Benzothiazol-2-yl)-1-pyrrolidinyl]methyl}benzonitrile | 5.66E+05 | 5.33E+05 | 1.67E+07 | 3.87E+06 | 9.76E+05 | 1.32E+07 | 6.18E+06 | 1.36E+07 | 3.85E+06 | 2.24E+06 | 0.15 |
| Com_166_pos | Choline | 4.26E+08 | 8.72E+08 | 7.27E+08 | 6.35E+08 | 5.90E+08 | 3.89E+09 | 7.84E+08 | 1.87E+09 | 4.74E+08 | 4.45E+08 | 0.31 |
| Com_16757_pos | 3,4-Dihydroxybenzaldehyde | 1.74E+07 | 4.24E+06 | 1.97E+06 | 5.78E+06 | 3.62E+06 | 2.11E+06 | 3.20E+06 | 3.82E+06 | 3.71E+06 | 2.19E+06 | 0.22 |
| Com_1676_pos | (±)-Abscisic acid | 3.41E+08 | 2.68E+08 | 5.04E+06 | 3.67E+08 | 1.14E+08 | 9.58E+07 | 1.29E+08 | 4.82E+07 | 2.40E+08 | 1.23E+08 | 0.98 |
| Com_16777_pos | Phenylpropiolic acid | 8.78E+06 | 2.69E+06 | 5.40E+06 | 6.14E+06 | 4.90E+06 | 3.79E+06 | 1.38E+07 | 4.29E+06 | 6.13E+06 | 5.84E+06 | 0.63 |
| Com_16838_pos | Formononetin | 2.41E+06 | 1.73E+06 | 1.15E+06 | 3.21E+06 | 3.93E+06 | 8.67E+06 | 7.22E+06 | 1.62E+07 | 2.52E+06 | 4.25E+06 | 0.03 |
| Com_1685_pos | Pyrogallol | 9.61E+07 | 1.23E+08 | 1.35E+08 | 8.97E+07 | 2.49E+08 | 1.11E+08 | 1.44E+08 | 3.98E+08 | 8.66E+07 | 1.42E+08 | 0.63 |
| Com_16893_neg | Glycochenodeoxycholic acid sodium salt | 8.35E+05 | 5.37E+05 | 2.85E+06 | 1.96E+05 | 2.68E+05 | 5.76E+05 | 1.52E+06 | 2.64E+05 | 1.79E+06 | 2.28E+06 | 0.42 |
| Com_168_neg | FAHFA (3:0/16:0) | 1.79E+08 | 3.05E+08 | 2.31E+09 | 4.06E+08 | 6.80E+08 | 7.26E+07 | 3.69E+08 | 2.59E+08 | 1.77E+08 | 3.50E+08 | 0.14 |
| Com_168_pos | Linoleoyl ethanolamide | 1.83E+09 | 1.70E+09 | 2.68E+09 | 1.06E+09 | 2.71E+09 | 1.01E+09 | 4.81E+09 | 4.62E+09 | 1.12E+09 | 1.05E+09 | 0.97 |
| Com_16914_neg | Feruloylcholine | 1.22E+06 | 1.23E+06 | 7.11E+05 | 1.73E+05 | 2.62E+06 | 8.51E+04 | 1.23E+06 | 1.40E+06 | 1.15E+05 | 1.47E+05 | 0.20 |
| Com_16968_pos | Galangin | 8.83E+06 | 7.67E+06 | 1.62E+07 | 7.00E+06 | 9.50E+06 | 9.69E+06 | 6.28E+06 | 5.46E+06 | 9.00E+06 | 6.57E+06 | 0.19 |
| Com_16988_pos | 3-amino-4-(propylamino)cyclobut-3-ene-1,2-dione | 7.86E+06 | 8.67E+06 | 1.62E+07 | 1.24E+07 | 8.12E+06 | 9.18E+06 | 9.39E+06 | 1.18E+07 | 1.11E+07 | 1.23E+07 | 0.78 |
| Com_169_neg | α-Lactose | 7.84E+07 | 2.62E+08 | 3.43E+08 | 4.81E+07 | 1.74E+09 | 5.69E+07 | 2.15E+08 | 4.74E+08 | 2.78E+07 | 7.59E+07 | 0.36 |
| Com_16_neg | Arachidonic acid | 9.46E+09 | 8.51E+09 | 1.03E+10 | 1.96E+10 | 1.64E+10 | 5.30E+09 | 1.88E+10 | 2.71E+10 | 1.06E+10 | 1.32E+10 | 0.84 |
| Com_1705_neg | Glycerol-3-phosphate | 5.42E+07 | 7.08E+07 | 3.31E+07 | 1.36E+07 | 7.09E+07 | 1.32E+07 | 2.57E+07 | 1.11E+07 | 1.78E+07 | 1.80E+07 | 0.04 |
| Com_170_neg | Uridine | 6.73E+08 | 1.21E+09 | 3.63E+08 | 8.36E+08 | 4.99E+08 | 4.07E+08 | 3.42E+08 | 5.29E+08 | 7.69E+07 | 1.09E+09 | 0.26 |
| Com_17104_pos | ethyl 2-{[(1-methyl-3-propyl-1H-pyrazol-4-yl)carbonyl]amino}acetate | 2.64E+06 | 2.39E+06 | 2.28E+06 | 2.29E+06 | 9.90E+06 | 1.65E+06 | 1.93E+07 | 6.22E+06 | 4.42E+06 | 4.26E+06 | 0.35 |
| Com_17112_pos | Jervine | 1.68E+07 | 1.26E+07 | 6.95E+06 | 4.65E+06 | 6.87E+06 | 3.98E+06 | 6.80E+06 | 3.58E+06 | 7.46E+06 | 3.40E+06 | 0.08 |
| Com_17127_pos | 3,3-dimethyl-5-oxo-5-[(3-phenyl-1H-pyrazol-5-yl)amino]pentanoic acid | 2.79E+06 | 3.39E+06 | 2.22E+06 | 1.30E+06 | 1.26E+07 | 2.08E+06 | 1.56E+06 | 1.37E+06 | 2.02E+06 | 1.51E+06 | 0.16 |
| Com_17157_pos | Deoxyguanosine | 5.53E+06 | 7.21E+06 | 1.59E+07 | 7.51E+06 | 1.06E+07 | 8.13E+06 | 1.24E+07 | 1.35E+07 | 5.99E+06 | 1.00E+07 | 0.69 |
| Com_17189_pos | 3-benzyl-4-hydroxy-5-(4-hydroxyphenyl)-2,5-dihydrofuran-2-one | 1.23E+07 | 9.11E+06 | 1.04E+06 | 5.94E+06 | 1.26E+07 | 3.62E+06 | 7.05E+06 | 1.96E+06 | 1.19E+07 | 5.05E+06 | 0.71 |
| Com_1721_pos | Isotretinoin | 2.99E+08 | 3.03E+08 | 2.54E+08 | 1.86E+08 | 2.64E+08 | 3.06E+08 | 1.45E+08 | 1.14E+08 | 3.35E+08 | 2.83E+08 | 0.50 |
| Com_1725_pos | LysoPE 18:0 | 2.74E+07 | 1.30E+08 | 2.58E+07 | 5.51E+07 | 2.16E+08 | 1.16E+07 | 1.43E+08 | 3.89E+08 | 4.14E+06 | 7.98E+07 | 0.74 |
| Com_17281_pos | L-Palmitoylcarnitine | 3.21E+06 | 3.10E+06 | 1.57E+07 | 1.35E+07 | 5.43E+06 | 6.74E+06 | 4.64E+06 | 1.15E+07 | 6.72E+06 | 6.77E+06 | 0.85 |
| Com_172_neg | 3-Hydroxy-3-methylglutaric acid | 1.72E+08 | 4.31E+08 | 3.54E+08 | 2.75E+08 | 1.73E+09 | 5.13E+08 | 5.88E+08 | 6.36E+08 | 3.32E+08 | 4.38E+08 | 0.71 |
| Com_1732_pos | Sakuranetin | 1.95E+07 | 1.99E+07 | 4.27E+06 | 3.55E+08 | 9.95E+06 | 8.34E+07 | 2.11E+08 | 1.62E+08 | 2.02E+08 | 1.14E+08 | 0.06 |
| Com_1735_pos | tert-Butyl N-[1-(aminocarbonyl)-3-methylbutyl]carbamate | 9.91E+07 | 2.08E+08 | 1.59E+07 | 3.26E+08 | 1.20E+08 | 2.76E+08 | 2.12E+08 | 2.01E+08 | 4.56E+07 | 3.76E+08 | 0.41 |
| Com_17395_pos | L-(-)-alpha-Amino-epsilon-Caprolactam | 5.55E+06 | 7.51E+06 | 1.83E+06 | 1.08E+07 | 8.44E+06 | 8.32E+06 | 6.93E+06 | 1.53E+07 | 2.01E+06 | 1.00E+07 | 0.69 |
| Com_17408_pos | Ureidosuccinic acid | 1.12E+07 | 1.26E+07 | 2.94E+06 | 2.06E+06 | 2.55E+06 | 7.23E+06 | 5.19E+06 | 4.66E+06 | 2.59E+06 | 5.25E+06 | 0.97 |
| Com_17457_neg | 2'-Deoxyadenosine 5'-monophosphate (dAMP) | 2.75E+05 | 3.31E+05 | 1.51E+05 | 5.87E+05 | 6.01E+05 | 1.74E+06 | 5.47E+05 | 8.62E+05 | 1.78E+05 | 1.45E+05 | 0.60 |
| Com_1745_neg | LPG 18:0 | 5.63E+05 | 9.51E+06 | 9.35E+05 | 1.98E+07 | 9.91E+07 | 4.18E+05 | 9.55E+05 | 2.28E+07 | 5.37E+05 | 1.35E+07 | 0.46 |
| Com_1745_pos | Cytosine | 2.75E+07 | 3.16E+07 | 1.29E+08 | 4.36E+07 | 2.12E+07 | 3.20E+08 | 3.89E+07 | 8.33E+06 | 1.88E+07 | 5.02E+07 | 0.99 |
| Com_17498_neg | Cynaropicrin | 2.10E+05 | 1.78E+05 | 2.84E+05 | 2.50E+06 | 2.27E+05 | 1.74E+05 | 3.28E+05 | 2.72E+05 | 1.65E+05 | 2.68E+05 | 0.44 |
| Com_17526_pos | LNH | 6.95E+06 | 7.63E+06 | 4.84E+06 | 2.84E+06 | 5.24E+06 | 5.06E+06 | 5.99E+06 | 2.23E+06 | 1.30E+07 | 5.27E+06 | 0.91 |
| Com_1752_neg | DGMG (18:2) | 1.21E+06 | 1.65E+06 | 2.19E+06 | 1.75E+06 | 5.68E+06 | 1.15E+06 | 1.78E+06 | 1.18E+08 | 9.02E+05 | 1.50E+06 | 0.69 |
| Com_17530_pos | 6-methyl-4-(morpholinomethyl)-2H-chromen-2-one | 1.92E+06 | 2.41E+06 | 5.76E+06 | 8.49E+06 | 2.25E+06 | 2.23E+06 | 3.12E+06 | 1.73E+06 | 1.30E+07 | 1.21E+07 | 0.63 |
| Com_17582_pos | LPC 4:0 | 2.94E+06 | 2.81E+06 | 2.13E+06 | 2.99E+06 | 1.21E+07 | 2.71E+06 | 4.65E+06 | 4.99E+06 | 1.84E+06 | 3.15E+06 | 0.76 |
| Com_17593_neg | Trehalose | 3.82E+05 | 6.78E+05 | 1.54E+06 | 2.98E+05 | 2.31E+06 | 1.14E+06 | 1.53E+06 | 1.80E+06 | 1.11E+06 | 5.35E+05 | 0.42 |
| Com_17595_pos | 2-(4-chlorophenyl)-3-phenylquinoxaline | 3.25E+06 | 1.24E+07 | 1.79E+06 | 3.31E+05 | 5.58E+06 | 1.69E+06 | 1.85E+06 | 9.76E+06 | 3.53E+06 | 2.61E+06 | 0.84 |
| Com_17596_pos | 4-(3,4-dimethoxyphenyl)-3-methyl-1H-pyrazol-5-amine | 9.46E+06 | 1.24E+07 | 3.55E+06 | 7.24E+06 | 8.67E+06 | 6.93E+06 | 7.12E+06 | 6.47E+06 | 8.85E+06 | 1.20E+07 | 0.84 |
| Com_17637_pos | Resiniferatoxin | 2.33E+06 | 6.71E+05 | 6.40E+05 | 3.63E+06 | 6.31E+05 | 4.21E+06 | 1.83E+07 | 8.08E+06 | 7.97E+06 | 4.86E+06 | 0.00 |
| Com_1765_pos | Anandamide (AEA) | 1.69E+08 | 3.13E+08 | 2.40E+08 | 1.39E+08 | 1.21E+08 | 1.60E+08 | 1.36E+08 | 8.55E+07 | 1.81E+08 | 1.49E+08 | 0.22 |
| Com_17684_pos | Papaverine | 7.93E+06 | 8.46E+06 | 8.21E+06 | 8.96E+05 | 1.20E+07 | 7.82E+06 | 6.61E+06 | 1.04E+07 | 1.13E+07 | 5.68E+06 | 0.50 |
| Com_17704_neg | 13,14-dihydro-15-keto-tetranor Prostaglandin E2 | 1.11E+06 | 1.04E+06 | 1.92E+06 | 1.29E+06 | 1.69E+06 | 1.67E+06 | 1.83E+06 | 1.23E+06 | 1.43E+06 | 1.26E+06 | 0.66 |
| Com_17730_pos | morphine-d3 | 1.91E+06 | 6.16E+06 | 1.35E+06 | 2.06E+06 | 9.08E+06 | 7.85E+06 | 3.55E+06 | 1.48E+07 | 1.45E+06 | 2.82E+06 | 0.54 |
| Com_1773_pos | 2'-Deoxyadenosine | 4.80E+07 | 3.10E+08 | 2.88E+08 | 1.34E+08 | 5.23E+06 | 1.96E+06 | 1.19E+06 | 2.19E+06 | 1.09E+08 | 1.63E+08 | 0.16 |
| Com_1776_neg | Phe-Pro | 1.56E+07 | 2.84E+07 | 6.50E+06 | 2.45E+07 | 3.13E+07 | 7.13E+07 | 1.20E+07 | 5.05E+07 | 2.02E+06 | 3.68E+07 | 0.92 |
| Com_17777_pos | 3-Methoxytyramine | 1.22E+07 | 1.19E+07 | 1.21E+07 | 5.98E+06 | 1.19E+07 | 7.97E+06 | 9.89E+06 | 1.12E+07 | 1.21E+07 | 1.13E+07 | 0.97 |
| Com_17812_neg | 2-Methylbutyl beta-D-glucopyranoside | 2.13E+06 | 8.30E+05 | 5.93E+05 | 2.03E+05 | 2.27E+05 | 2.24E+05 | 3.84E+05 | 2.73E+05 | 1.32E+06 | 2.66E+05 | 0.53 |
| Com_17814_pos | SNH | 2.89E+06 | 3.56E+06 | 1.17E+07 | 6.62E+06 | 5.65E+06 | 1.23E+07 | 9.52E+06 | 8.96E+06 | 1.04E+07 | 8.39E+06 | 0.07 |
| Com_1790_pos | Carvone | 2.96E+08 | 2.58E+08 | 2.99E+08 | 3.40E+08 | 1.90E+08 | 1.30E+08 | 2.12E+08 | 2.44E+08 | 1.24E+08 | 1.65E+08 | 0.02 |
| Com_17925_pos | IPH | 2.07E+06 | 4.25E+06 | 5.51E+05 | 1.34E+07 | 1.18E+06 | 3.15E+06 | 8.26E+05 | 1.80E+06 | 1.64E+06 | 1.09E+07 | 0.98 |
| Com_17937_pos | UR-144 N-(2-hydroxypentyl) metabolite | 6.53E+06 | 8.15E+06 | 2.24E+06 | 1.34E+06 | 1.17E+07 | 8.75E+06 | 2.80E+06 | 1.06E+07 | 1.87E+06 | 2.47E+06 | 0.87 |
| Com_17960_pos | D-(-)-Salicin | 6.57E+05 | 2.19E+06 | 7.61E+05 | 1.50E+06 | 5.45E+06 | 1.18E+07 | 1.78E+07 | 1.45E+07 | 1.34E+06 | 9.80E+06 | 0.02 |
| Com_17963_pos | 20-Hydroxy prostaglandin F2α | 1.08E+07 | 9.86E+06 | 5.19E+06 | 4.88E+06 | 7.02E+06 | 4.47E+06 | 8.59E+06 | 3.90E+06 | 1.25E+07 | 5.58E+06 | 0.67 |
| Com_179_neg | D-(-)-Lyxose | 2.60E+08 | 4.00E+08 | 1.24E+09 | 5.46E+08 | 6.75E+08 | 5.58E+08 | 2.70E+09 | 1.34E+09 | 3.55E+08 | 9.90E+08 | 0.25 |
| Com_18016_pos | Phenytoin | 1.03E+07 | 1.20E+07 | 2.84E+06 | 9.24E+06 | 6.81E+06 | 6.60E+06 | 7.68E+06 | 4.30E+06 | 4.40E+06 | 3.33E+06 | 0.24 |
| Com_1801_neg | PG (3:0/18:1) | 1.26E+06 | 6.65E+07 | 5.41E+06 | 1.44E+07 | 5.43E+06 | 1.16E+06 | 4.77E+06 | 1.93E+07 | 2.20E+06 | 2.60E+06 | 0.35 |
| Com_18051_pos | 2-[(butylamino)(imino)methyl]-1-oxohydrazinium-1-olate | 8.07E+05 | 6.74E+05 | 6.80E+05 | 3.52E+06 | 5.49E+05 | 1.20E+07 | 5.78E+05 | 5.25E+06 | 6.36E+05 | 9.03E+05 | 0.37 |
| Com_1805_pos | RPK | 1.23E+07 | 2.56E+07 | 3.73E+08 | 1.89E+07 | 2.82E+07 | 9.80E+07 | 3.62E+07 | 3.60E+07 | 1.67E+07 | 1.61E+07 | 0.87 |
| Com_1809_pos | all-cis-4,7,10,13,16-Docosapentaenoic acid | 5.57E+07 | 4.44E+07 | 6.37E+07 | 3.37E+08 | 9.14E+07 | 5.14E+07 | 1.01E+08 | 5.11E+07 | 9.62E+07 | 6.27E+07 | 0.59 |
| Com_1810_pos | 2-Methylpentanedioic acid | 3.90E+08 | 1.02E+08 | 1.37E+08 | 7.76E+07 | 5.66E+07 | 3.07E+07 | 6.76E+07 | 8.32E+07 | 1.05E+08 | 6.04E+07 | 0.16 |
| Com_18151_pos | Tetranor-12(S)-HETE | 1.52E+07 | 5.24E+06 | 7.44E+05 | 1.34E+06 | 8.80E+05 | 1.13E+06 | 9.00E+05 | 8.27E+05 | 1.56E+06 | 4.91E+06 | 0.50 |
| Com_18177_pos | Neosaxitoxin | 1.51E+07 | 3.47E+06 | 1.62E+06 | 1.08E+06 | 8.21E+05 | 9.00E+05 | 7.94E+05 | 7.30E+05 | 1.10E+06 | 9.12E+05 | 0.13 |
| Com_18180_pos | 6-Keto-prostaglandin f1alpha | 1.40E+07 | 7.29E+06 | 7.00E+06 | 7.44E+06 | 5.30E+06 | 4.12E+06 | 8.87E+06 | 1.69E+06 | 1.22E+07 | 7.36E+06 | 0.43 |
| Com_18214_pos | 1,7-bis(4-hydroxyphenyl)heptan-3-one | 1.76E+06 | 1.00E+06 | 1.55E+06 | 1.84E+06 | 2.09E+06 | 1.18E+07 | 2.15E+06 | 4.94E+06 | 1.54E+06 | 1.43E+06 | 0.18 |
| Com_18220_pos | 3-hydroxy-N-(1-hydroxy-4-methylpentan-2-yl)-5-oxo-6-phenylhexanamide | 1.13E+07 | 1.17E+07 | 2.25E+06 | 3.44E+06 | 3.81E+06 | 2.50E+06 | 1.93E+06 | 2.39E+06 | 2.59E+06 | 5.25E+06 | 0.14 |
| Com_18280_pos | VLK | 4.04E+06 | 6.15E+06 | 1.20E+06 | 1.29E+07 | 5.75E+06 | 6.09E+06 | 6.56E+06 | 9.57E+06 | 2.84E+06 | 9.85E+06 | 0.51 |
| Com_18317_pos | Traumatic acid | 1.29E+06 | 1.08E+07 | 1.02E+06 | 1.69E+06 | 8.74E+06 | 8.69E+05 | 9.75E+05 | 1.40E+07 | 9.72E+05 | 9.66E+05 | 0.45 |
| Com_18406_pos | Levodopa | 6.90E+06 | 8.39E+06 | 9.15E+06 | 6.17E+06 | 1.12E+07 | 9.32E+06 | 1.01E+07 | 1.14E+07 | 8.72E+06 | 8.87E+06 | 0.21 |
| Com_18430_pos | 7-Aminoflunitrazepam-d7 | 3.61E+06 | 1.08E+07 | 5.13E+05 | 1.86E+06 | 1.11E+07 | 4.23E+06 | 1.11E+07 | 6.65E+06 | 7.23E+05 | 1.70E+06 | 0.98 |
| Com_18441_pos | ANK | 1.31E+06 | 2.78E+06 | 6.06E+06 | 9.45E+06 | 3.25E+06 | 1.01E+07 | 4.33E+06 | 1.39E+07 | 1.88E+06 | 2.26E+06 | 0.62 |
| Com_18460_neg | Kinetin 9-riboside | 2.50E+05 | 1.71E+05 | 1.86E+05 | 1.57E+05 | 2.27E+05 | 1.43E+06 | 2.44E+05 | 1.81E+05 | 1.11E+05 | 1.75E+05 | 0.55 |
| Com_18466_pos | 2-Naphthol | 4.73E+06 | 1.14E+07 | 8.67E+05 | 1.28E+06 | 8.40E+05 | 5.51E+05 | 9.93E+05 | 1.04E+06 | 6.42E+05 | 2.02E+06 | 0.19 |
| Com_18472_pos | QLH | 9.20E+06 | 1.09E+07 | 8.40E+06 | 6.66E+06 | 1.11E+07 | 5.73E+06 | 8.47E+06 | 9.40E+06 | 7.11E+06 | 8.06E+06 | 0.21 |
| Com_184_neg | 2-Isopropylmalate | 5.68E+07 | 2.39E+08 | 1.67E+08 | 5.78E+07 | 1.57E+09 | 5.32E+07 | 4.27E+08 | 5.30E+08 | 6.68E+07 | 4.85E+07 | 0.69 |
| Com_184_pos | 3-methyl-5-oxo-5-(4-toluidino)pentanoic acid | 1.41E+09 | 5.79E+08 | 1.99E+09 | 8.98E+08 | 3.52E+09 | 1.69E+09 | 1.87E+09 | 3.21E+09 | 7.68E+08 | 1.18E+09 | 0.78 |
| Com_1850_pos | N-Acetylvaline | 3.72E+07 | 9.81E+07 | 1.84E+08 | 1.25E+08 | 2.04E+08 | 8.88E+07 | 1.16E+08 | 1.44E+08 | 2.11E+08 | 3.45E+08 | 0.37 |
| Com_1851_pos | gamma-Glutamylmethionine | 4.04E+07 | 1.12E+08 | 6.42E+07 | 1.56E+08 | 7.17E+07 | 2.95E+08 | 4.04E+07 | 3.54E+08 | 2.48E+07 | 1.41E+08 | 0.62 |
| Com_18532_pos | LPC 3:0 | 1.64E+06 | 1.12E+06 | 2.10E+06 | 2.70E+06 | 1.10E+07 | 1.42E+06 | 1.90E+06 | 2.52E+06 | 1.18E+06 | 2.23E+06 | 0.41 |
| Com_1853_pos | L-Pipecolate | 1.61E+08 | 1.04E+08 | 3.58E+08 | 2.22E+08 | 2.38E+08 | 1.26E+08 | 1.72E+08 | 5.43E+07 | 1.05E+08 | 2.33E+08 | 0.18 |
| Com_18569_pos | methyl 1-isopropyl-1H-1,2,3-benzotriazole-5-carboxylate | 4.10E+06 | 1.03E+07 | 2.18E+06 | 3.92E+06 | 1.99E+06 | 3.45E+06 | 2.93E+06 | 1.12E+06 | 4.24E+06 | 1.33E+07 | 0.96 |
| Com_18571_pos | Stanozolol | 1.12E+06 | 1.15E+06 | 1.38E+07 | 1.35E+06 | 5.64E+05 | 5.54E+05 | 6.14E+05 | 6.73E+05 | 2.42E+06 | 7.60E+05 | 0.30 |
| Com_18589_neg | Dithranol | 1.37E+06 | 1.27E+06 | 1.04E+06 | 5.58E+05 | 1.90E+06 | 1.30E+06 | 1.80E+06 | 1.23E+06 | 1.20E+06 | 8.47E+05 | 0.73 |
| Com_185_pos | N-Benzylformamide | 5.18E+08 | 1.09E+09 | 7.85E+08 | 1.34E+09 | 1.49E+09 | 1.92E+09 | 1.26E+09 | 4.32E+09 | 6.98E+08 | 2.02E+09 | 0.16 |
| Com_1866_pos | N,N-Diethylethanolamine | 2.61E+08 | 1.71E+08 | 1.82E+08 | 6.72E+07 | 1.55E+08 | 5.87E+07 | 1.27E+08 | 1.01E+08 | 3.02E+08 | 1.05E+08 | 0.49 |
| Com_1870_neg | 4-Chlorophenol | 8.19E+06 | 1.64E+07 | 2.94E+07 | 9.69E+07 | 9.91E+06 | 7.47E+06 | 4.86E+07 | 1.06E+07 | 9.04E+06 | 7.29E+07 | 0.90 |
| Com_18712_neg | N-Acetyl-Asp-Glu | 9.20E+05 | 9.02E+05 | 2.21E+06 | 8.56E+05 | 1.26E+06 | 1.36E+06 | 2.72E+06 | 1.66E+06 | 9.98E+05 | 9.32E+05 | 0.44 |
| Com_1877_pos | 2-Methoxybenzaldehyde | 1.90E+07 | 1.46E+07 | 7.75E+06 | 1.46E+08 | 1.61E+07 | 1.00E+08 | 2.75E+08 | 1.47E+08 | 3.00E+08 | 1.98E+08 | 0.01 |
| Com_187_neg | 2,2-Bis(hydroxymethyl)propionic acid | 1.77E+08 | 3.09E+08 | 2.45E+08 | 7.21E+08 | 4.97E+08 | 3.61E+08 | 2.47E+09 | 1.04E+09 | 9.60E+08 | 3.57E+08 | 0.10 |
| Com_1880_pos | Estrone | 7.10E+07 | 4.52E+07 | 1.53E+07 | 4.36E+06 | 6.40E+07 | 2.91E+08 | 1.80E+07 | 7.69E+07 | 9.76E+07 | 5.18E+07 | 0.19 |
| Com_188_neg | 1,3-Dimethyluracil | 3.90E+07 | 9.76E+07 | 6.20E+07 | 2.06E+08 | 1.85E+08 | 4.71E+08 | 7.28E+08 | 9.68E+08 | 1.02E+09 | 1.46E+08 | 0.01 |
| Com_18915_pos | MNK | 6.50E+06 | 5.80E+06 | 1.33E+07 | 3.28E+06 | 1.44E+06 | 9.74E+05 | 1.23E+06 | 1.31E+06 | 3.03E+06 | 9.81E+06 | 0.20 |
| Com_18950_pos | Atropine | 7.59E+06 | 1.09E+07 | 8.54E+06 | 5.09E+06 | 5.79E+06 | 2.33E+06 | 2.36E+06 | 2.10E+06 | 2.85E+06 | 6.23E+06 | 0.01 |
| Com_18961_neg | N-Acetyl-DL-phenylalanine | 4.48E+05 | 3.85E+05 | 3.42E+05 | 6.70E+05 | 3.28E+05 | 1.29E+06 | 7.59E+05 | 2.04E+06 | 2.10E+05 | 5.45E+05 | 0.22 |
| Com_1896_neg | Gentisic acid | 7.77E+07 | 6.22E+06 | 4.56E+07 | 2.70E+07 | 8.99E+07 | 4.75E+07 | 5.19E+07 | 6.53E+06 | 4.98E+07 | 3.85E+07 | 0.87 |
| Com_18996_pos | 4-(allyloxy)-1,2-dihydroquinolin-2-one | 1.98E+06 | 2.72E+06 | 2.44E+06 | 3.23E+06 | 1.61E+06 | 3.71E+06 | 3.39E+06 | 1.31E+07 | 1.71E+06 | 2.97E+06 | 0.22 |
| Com_18_pos | Hexadecanamide | 1.87E+10 | 2.76E+10 | 4.19E+10 | 1.59E+10 | 2.32E+10 | 7.64E+09 | 3.03E+10 | 2.15E+10 | 1.17E+10 | 1.72E+10 | 0.20 |
| Com_19018_pos | D-Fructose 6-phosphate | 2.67E+06 | 2.59E+06 | 1.32E+07 | 4.91E+06 | 9.97E+06 | 2.47E+06 | 3.88E+06 | 3.75E+06 | 4.03E+06 | 2.09E+06 | 0.19 |
| Com_1901_pos | D-Pantethine | 5.99E+07 | 5.36E+07 | 1.95E+06 | 7.82E+07 | 1.27E+07 | 1.81E+07 | 3.71E+07 | 2.91E+06 | 2.96E+08 | 2.77E+08 | 0.58 |
| Com_1909_pos | All-Trans-13,14-Dihydroretinol | 4.26E+07 | 3.86E+07 | 4.02E+07 | 3.15E+08 | 7.70E+07 | 4.16E+07 | 7.46E+07 | 4.15E+07 | 7.52E+07 | 4.36E+07 | 0.55 |
| Com_1911_neg | Hypotaurine | 1.48E+06 | 1.01E+07 | 4.51E+06 | 9.60E+06 | 8.91E+07 | 5.26E+06 | 4.12E+07 | 4.52E+07 | 1.01E+06 | 3.13E+07 | 0.74 |
| Com_1912_pos | 3,6-bis[(1H-indol-3-yl)methyl]piperazine-2,5-dione | 2.50E+07 | 1.20E+07 | 1.46E+07 | 2.96E+07 | 2.75E+08 | 1.95E+07 | 2.19E+07 | 1.12E+08 | 6.16E+06 | 2.09E+07 | 0.64 |
| Com_19147_neg | Adenosine 3'5'-cyclic monophosphate | 2.95E+05 | 3.15E+05 | 2.22E+06 | 1.61E+06 | 2.33E+05 | 3.73E+05 | 4.26E+05 | 2.86E+05 | 3.90E+05 | 1.54E+06 | 0.72 |
| Com_19163_pos | APH | 6.43E+05 | 4.53E+05 | 5.77E+05 | 1.18E+07 | 2.97E+05 | 1.35E+06 | 1.59E+06 | 1.88E+06 | 4.14E+05 | 2.86E+06 | 0.59 |
| Com_19170_pos | N-(2-morpholinophenyl)-2-furamide | 1.29E+07 | 8.82E+06 | 1.28E+07 | 6.91E+06 | 1.03E+07 | 3.40E+06 | 8.43E+06 | 3.76E+06 | 3.50E+06 | 8.07E+06 | 0.02 |
| Com_19191_pos | geranyl pp | 1.35E+06 | 1.45E+06 | 1.54E+06 | 4.53E+06 | 1.52E+06 | 6.20E+06 | 1.19E+07 | 4.35E+06 | 1.11E+07 | 9.98E+06 | 0.00 |
| Com_19222_pos | N-(2,3-dihydro-1,4-benzodioxin-6-yl)-4-fluorobenzamide | 8.35E+06 | 1.06E+07 | 7.65E+06 | 4.16E+06 | 6.58E+06 | 4.66E+06 | 5.59E+06 | 6.68E+06 | 7.14E+06 | 4.09E+06 | 0.21 |
| Com_19257_pos | SPK | 2.86E+06 | 4.69E+06 | 1.18E+06 | 1.11E+07 | 7.69E+06 | 1.07E+07 | 7.32E+06 | 7.32E+06 | 2.15E+06 | 7.61E+06 | 0.45 |
| Com_19274_pos | 2-[(3S)-1-(3-Chlorobenzyl)-3-pyrrolidinyl]-5-methyl-1,3-benzoxazole | 1.65E+06 | 2.38E+06 | 7.47E+05 | 8.97E+05 | 1.02E+07 | 9.04E+05 | 7.85E+05 | 6.87E+05 | 6.72E+05 | 8.89E+05 | 0.12 |
| Com_19311_pos | Obscurolide A1 | 6.03E+05 | 9.35E+05 | 2.94E+05 | 2.35E+06 | 1.02E+07 | 3.94E+05 | 1.02E+06 | 2.85E+06 | 3.64E+05 | 3.43E+05 | 0.40 |
| Com_19330_pos | Cytidine-5'-monophosphate | 7.49E+06 | 1.01E+07 | 3.30E+06 | 4.85E+06 | 5.43E+06 | 1.06E+07 | 3.81E+06 | 3.53E+06 | 1.02E+07 | 6.10E+06 | 0.85 |
| Com_19379_neg | Thymidine 3,5-cyclic monophosphate | 2.44E+05 | 1.10E+06 | 1.07E+06 | 4.23E+05 | 4.86E+05 | 5.94E+05 | 5.86E+05 | 1.60E+06 | 3.26E+05 | 1.31E+06 | 0.52 |
| Com_1940_neg | LPE 13:0 | 1.79E+07 | 6.10E+07 | 1.50E+06 | 1.90E+07 | 5.75E+07 | 5.32E+07 | 2.41E+06 | 4.69E+07 | 8.01E+05 | 3.67E+06 | 0.42 |
| Com_19441_pos | 4-methyl-3-(methylthio)-5-undecyl-4H-1,2,4-triazole | 2.20E+06 | 5.28E+06 | 7.06E+06 | 9.57E+06 | 1.01E+07 | 8.48E+06 | 5.25E+06 | 1.07E+07 | 2.35E+06 | 6.78E+06 | 0.98 |
| Com_19445_pos | O-7460 | 1.01E+07 | 1.04E+07 | 2.73E+06 | 5.66E+06 | 7.77E+06 | 6.53E+06 | 6.27E+06 | 3.70E+06 | 8.12E+06 | 1.06E+07 | 0.99 |
| Com_19469_pos | IRH | 1.55E+06 | 3.48E+06 | 7.05E+05 | 1.15E+07 | 2.78E+06 | 3.56E+06 | 7.97E+06 | 4.43E+06 | 1.96E+06 | 7.89E+06 | 0.34 |
| Com_19486_pos | Flavanone | 1.11E+07 | 1.03E+07 | 7.84E+05 | 3.87E+06 | 8.52E+06 | 2.05E+06 | 6.72E+06 | 2.39E+06 | 9.95E+06 | 6.05E+06 | 0.90 |
| Com_194_pos | Styrene | 1.43E+08 | 1.38E+08 | 5.25E+07 | 4.87E+08 | 4.54E+08 | 3.44E+09 | 3.93E+08 | 6.47E+08 | 4.54E+07 | 1.16E+08 | 0.51 |
| Com_19503_pos | (2R,3S,4S,5R,6R)-2-(hydroxymethyl)-6-(propan-2-yloxy)oxane-3,4,5-triol | 8.03E+06 | 1.03E+07 | 1.00E+06 | 2.61E+06 | 5.64E+06 | 4.84E+06 | 3.65E+06 | 1.81E+06 | 7.83E+06 | 2.66E+06 | 0.81 |
| Com_19533_pos | (+/-)-UR-144 N-(4-Hydroxypentyl) metabolite | 7.23E+06 | 1.03E+07 | 5.27E+05 | 9.10E+05 | 7.38E+05 | 5.68E+05 | 8.70E+05 | 7.44E+05 | 9.38E+05 | 1.20E+06 | 0.25 |
| Com_1955_pos | (R)-Equol | 3.56E+08 | 2.65E+08 | 1.62E+06 | 2.76E+08 | 1.55E+08 | 8.43E+07 | 9.60E+07 | 5.09E+07 | 7.28E+07 | 6.50E+07 | 0.82 |
| Com_195_pos | Glycitein | 1.14E+09 | 1.57E+09 | 5.16E+07 | 2.42E+09 | 2.44E+09 | 3.36E+09 | 3.68E+09 | 4.10E+09 | 5.69E+08 | 2.47E+09 | 0.28 |
| Com_19683_pos | 4-ethoxy-N-[(4-methoxyphenyl)methylene]aniline | 8.33E+06 | 1.02E+07 | 4.27E+05 | 2.08E+06 | 3.00E+06 | 8.14E+05 | 4.10E+05 | 4.56E+05 | 1.58E+06 | 3.17E+06 | 0.14 |
| Com_19685_pos | N'',N'''-di[1-(4-nitrophenyl)ethylidene]carbonic dihydrazide | 1.33E+06 | 4.78E+05 | 4.48E+05 | 2.97E+06 | 4.34E+05 | 1.28E+06 | 3.36E+06 | 6.44E+05 | 1.06E+07 | 4.06E+06 | 0.10 |
| Com_1968_pos | D-Sphingosine | 4.85E+07 | 2.74E+08 | 1.18E+08 | 3.01E+07 | 1.77E+08 | 2.38E+08 | 1.14E+08 | 9.17E+07 | 2.55E+07 | 7.40E+07 | 0.84 |
| Com_19785_pos | Senecionine | 1.01E+07 | 1.01E+07 | 1.84E+06 | 3.20E+06 | 3.37E+06 | 2.98E+06 | 4.64E+06 | 9.43E+05 | 6.81E+06 | 6.59E+06 | 0.63 |
| Com_1980_pos | N-Methylalanine | 2.37E+07 | 2.72E+08 | 2.55E+07 | 8.55E+07 | 1.77E+07 | 3.63E+07 | 1.81E+07 | 1.35E+07 | 3.74E+07 | 6.60E+07 | 0.44 |
| Com_19846_pos | Vitamin B2 | 3.79E+06 | 1.00E+07 | 3.69E+05 | 1.04E+06 | 8.40E+05 | 8.59E+05 | 6.28E+05 | 1.61E+06 | 6.13E+05 | 9.16E+05 | 0.34 |
| Com_19941_pos | L-Tryptophan | 2.80E+06 | 2.60E+06 | 1.10E+06 | 2.09E+06 | 9.64E+06 | 1.73E+06 | 2.01E+06 | 2.53E+06 | 1.08E+06 | 1.52E+06 | 0.26 |
| Com_19953_pos | 7-(2-thienyl)[1,2,4]triazolo[4,3-a]pyrimidine | 6.71E+06 | 3.83E+06 | 9.27E+05 | 5.36E+06 | 6.65E+06 | 4.17E+06 | 3.75E+06 | 3.02E+06 | 1.03E+07 | 5.32E+06 | 0.62 |
| Com_1995_pos | 2-hydroxy-6-[(8Z,11Z)-pentadeca-8,11,14-trien-1-yl]benzoic acid | 2.29E+08 | 1.02E+08 | 6.91E+07 | 2.12E+08 | 2.07E+08 | 4.65E+07 | 2.83E+08 | 3.27E+08 | 1.77E+08 | 1.87E+08 | 0.75 |
| Com_19995_pos | Prostaglandin J2 | 9.17E+06 | 9.71E+06 | 4.68E+06 | 1.10E+07 | 1.91E+06 | 4.59E+06 | 5.52E+06 | 2.94E+06 | 4.71E+06 | 6.13E+06 | 0.46 |
| Com_19_pos | Oleoyl ethylamide | 2.18E+10 | 2.38E+10 | 4.14E+10 | 1.83E+10 | 2.58E+10 | 1.31E+10 | 2.58E+10 | 2.71E+10 | 1.91E+10 | 1.95E+10 | 0.28 |
| Com_1_pos | Oleamide | 1.47E+11 | 1.86E+11 | 2.49E+11 | 1.25E+11 | 1.66E+11 | 9.13E+10 | 2.17E+11 | 1.87E+11 | 1.12E+11 | 1.24E+11 | 0.35 |
| Com_20006_pos | Puerarin | 1.21E+06 | 1.59E+06 | 4.40E+05 | 1.60E+06 | 8.64E+05 | 9.95E+06 | 1.26E+07 | 9.75E+06 | 2.61E+06 | 3.36E+06 | 0.00 |
| Com_2001_pos | Lovastatin | 2.52E+08 | 2.55E+08 | 2.44E+08 | 2.38E+08 | 1.61E+08 | 2.05E+08 | 1.03E+08 | 1.10E+08 | 2.81E+08 | 2.29E+08 | 0.26 |
| Com_20029_pos | HLK | 3.22E+06 | 5.27E+06 | 7.85E+05 | 7.88E+06 | 3.77E+06 | 9.92E+06 | 3.45E+06 | 9.48E+06 | 2.61E+06 | 6.43E+06 | 0.30 |
| Com_2005_pos | β-Cortolone | 6.96E+07 | 1.58E+08 | 4.43E+07 | 1.24E+08 | 1.48E+08 | 2.72E+08 | 3.22E+08 | 1.85E+08 | 2.02E+08 | 1.19E+08 | 0.04 |
| Com_20072_pos | 2-(tert-butyl)-1,3-thiazolane-4-carboxylic acid | 6.42E+05 | 6.16E+05 | 4.90E+05 | 1.69E+06 | 3.00E+06 | 1.63E+06 | 9.21E+05 | 2.97E+06 | 6.42E+05 | 1.15E+07 | 0.29 |
| Com_20174_pos | Milbemycin A3 oxime | 2.50E+06 | 4.45E+06 | 3.68E+06 | 2.58E+06 | 4.31E+06 | 9.78E+06 | 4.50E+06 | 7.12E+06 | 3.12E+06 | 1.95E+06 | 0.40 |
| Com_2026_pos | DL-Carnitine | 1.53E+08 | 7.07E+07 | 2.67E+08 | 2.63E+08 | 5.51E+07 | 9.33E+07 | 6.59E+07 | 1.36E+08 | 2.49E+08 | 3.14E+08 | 0.84 |
| Com_20270_neg | Biopterin | 1.00E+06 | 8.64E+05 | 1.63E+06 | 8.03E+05 | 7.51E+05 | 6.82E+05 | 9.80E+05 | 5.89E+05 | 5.53E+05 | 1.44E+06 | 0.41 |
| Com_20285_pos | ACar 12:3 | 1.55E+06 | 1.35E+06 | 1.18E+07 | 1.43E+06 | 1.29E+06 | 2.34E+06 | 1.26E+06 | 1.23E+06 | 1.24E+06 | 1.22E+06 | 0.39 |
| Com_20306_pos | Dutasteride | 4.09E+06 | 6.98E+06 | 2.32E+06 | 4.47E+06 | 5.89E+06 | 2.19E+06 | 4.55E+06 | 1.29E+06 | 9.97E+06 | 2.39E+06 | 0.42 |
| Com_20312_pos | Choline Glycerophosphate | 1.42E+06 | 3.31E+06 | 1.30E+06 | 2.75E+06 | 9.30E+06 | 5.56E+06 | 1.27E+06 | 9.57E+06 | 1.08E+06 | 1.11E+06 | 0.83 |
| Com_20317_pos | Arecoline | 6.43E+06 | 9.56E+06 | 6.86E+06 | 1.61E+06 | 7.22E+06 | 3.50E+06 | 6.31E+06 | 5.30E+06 | 4.61E+06 | 5.62E+06 | 0.78 |
| Com_2031_pos | Tauro-alpha-Muricholic acid sodium salt | 3.42E+08 | 9.74E+07 | 2.58E+08 | 4.98E+07 | 1.03E+07 | 2.89E+07 | 1.59E+08 | 1.23E+07 | 1.86E+07 | 3.12E+07 | 0.24 |
| Com_2034_pos | Ala-Ile | 6.65E+07 | 2.31E+08 | 7.81E+06 | 1.72E+08 | 7.33E+07 | 2.68E+08 | 3.91E+07 | 1.18E+08 | 2.08E+07 | 2.46E+08 | 0.72 |
| Com_20404_pos | 3,4-dihydro-2H,6H-[1,3]thiazino[2,3-b]quinazolin-6-one | 1.22E+07 | 9.11E+06 | 3.59E+06 | 1.75E+06 | 6.63E+06 | 1.59E+06 | 2.97E+06 | 2.03E+06 | 2.37E+06 | 2.65E+06 | 0.07 |
| Com_20418_pos | Tramadol N-Oxide | 1.01E+07 | 4.72E+06 | 1.67E+06 | 4.00E+06 | 2.38E+06 | 9.58E+06 | 2.26E+06 | 2.45E+06 | 3.04E+06 | 4.96E+06 | 0.98 |
| Com_20433_pos | 2-(benzylamino)-1,3-thiazole-5-carboxylic acid | 1.76E+06 | 8.12E+05 | 1.51E+06 | 5.43E+06 | 2.65E+06 | 5.40E+06 | 1.40E+07 | 1.12E+07 | 2.98E+06 | 4.46E+06 | 0.03 |
| Com_20434_pos | Kaempferol | 8.72E+05 | 5.31E+05 | 6.51E+05 | 6.00E+05 | 9.43E+05 | 9.57E+06 | 6.98E+05 | 1.09E+06 | 8.04E+05 | 9.05E+05 | 0.23 |
| Com_2043_pos | 7-Ketocholesterol | 2.36E+08 | 1.43E+08 | 4.21E+07 | 2.62E+08 | 1.59E+08 | 2.55E+08 | 2.28E+08 | 1.41E+08 | 1.18E+08 | 1.73E+08 | 0.58 |
| Com_20451_pos | β-Estradiol | 1.83E+06 | 8.05E+05 | 3.57E+06 | 4.41E+06 | 2.20E+06 | 1.81E+06 | 3.85E+06 | 1.30E+06 | 6.88E+06 | 1.12E+07 | 0.33 |
| Com_2046_neg | N-Glycolylneuraminic acid | 4.30E+06 | 5.16E+06 | 8.67E+06 | 8.41E+06 | 7.62E+06 | 6.00E+07 | 1.63E+07 | 2.14E+07 | 5.85E+06 | 1.31E+07 | 0.06 |
| Com_2047_neg | Prostaglandin K1 | 7.82E+07 | 1.77E+07 | 6.05E+06 | 6.29E+06 | 4.58E+06 | 5.71E+06 | 2.84E+06 | 2.57E+06 | 1.77E+06 | 6.09E+06 | 0.07 |
| Com_20487_pos | 1-adamantyl(piperidino)methanone | 4.42E+06 | 6.29E+06 | 6.32E+06 | 3.43E+06 | 9.17E+06 | 1.21E+06 | 3.25E+06 | 1.84E+06 | 2.09E+06 | 4.61E+06 | 0.02 |
| Com_20495_pos | 2-methyl-1H-benzimidazole-5-carboxylic acid | 2.00E+06 | 2.26E+06 | 2.09E+06 | 1.26E+06 | 6.84E+06 | 2.47E+06 | 1.40E+07 | 1.02E+06 | 2.32E+06 | 1.67E+06 | 0.85 |
| Com_204_neg | Xylitol | 8.49E+07 | 1.18E+08 | 2.46E+08 | 7.47E+08 | 1.10E+08 | 2.25E+08 | 8.66E+08 | 3.41E+08 | 9.67E+08 | 2.35E+08 | 0.13 |
| Com_204_pos | gamma-Glutamylleucine | 1.59E+08 | 3.81E+08 | 3.21E+08 | 2.80E+09 | 3.66E+08 | 3.23E+09 | 2.33E+08 | 2.52E+09 | 2.24E+08 | 6.35E+08 | 0.50 |
| Com_2050_pos | Ala-Val | 9.06E+07 | 2.62E+08 | 1.14E+07 | 1.26E+08 | 1.18E+08 | 1.96E+08 | 3.52E+07 | 1.67E+08 | 1.74E+07 | 2.08E+08 | 0.99 |
| Com_20549_pos | INH | 9.14E+06 | 9.23E+06 | 8.01E+06 | 5.70E+06 | 9.11E+06 | 6.79E+06 | 8.61E+06 | 5.59E+06 | 7.82E+06 | 7.52E+06 | 0.34 |
| Com_20573_neg | ADBICA N-pentanoic acid metabolite | 1.10E+05 | 9.66E+04 | 1.55E+05 | 1.19E+06 | 7.32E+05 | 1.25E+05 | 1.85E+05 | 1.52E+05 | 1.84E+05 | 1.63E+05 | 0.37 |
| Com_2058_pos | 13-HPODE | 2.40E+08 | 1.35E+08 | 2.13E+08 | 1.66E+08 | 1.55E+08 | 1.07E+08 | 2.26E+08 | 2.15E+08 | 2.72E+08 | 8.03E+07 | 0.74 |
| Com_20612_neg | 5,6-Dihydroxyindole-2-Carboxylic Acid | 4.43E+05 | 4.73E+05 | 6.44E+05 | 2.25E+05 | 2.82E+05 | 4.78E+05 | 7.48E+05 | 3.03E+05 | 7.54E+05 | 2.10E+05 | 0.67 |
| Com_20616_pos | (3R)-8-hydroxy-3-(4-methoxyphenyl)-3,4-dihydro-1H-2-benzopyran-1-one | 1.10E+07 | 2.58E+06 | 1.08E+06 | 3.28E+06 | 7.29E+06 | 2.08E+06 | 5.37E+06 | 1.75E+06 | 9.71E+06 | 5.54E+06 | 0.89 |
| Com_2061_pos | ACar 18:2 | 1.52E+07 | 4.31E+07 | 1.76E+07 | 4.83E+07 | 4.49E+07 | 1.32E+08 | 2.10E+07 | 3.15E+08 | 2.24E+07 | 1.26E+07 | 0.52 |
| Com_2062_pos | Sinapyl aldehyde | 1.78E+08 | 1.10E+08 | 3.18E+08 | 8.85E+07 | 1.51E+08 | 1.80E+08 | 2.08E+08 | 9.62E+07 | 1.46E+08 | 1.18E+08 | 0.82 |
| Com_2071_pos | Corticosterone | 5.90E+07 | 1.05E+08 | 6.12E+07 | 8.40E+07 | 9.89E+07 | 2.61E+08 | 2.11E+08 | 1.02E+08 | 9.09E+07 | 3.10E+07 | 0.45 |
| Com_20722_neg | Thymopentin | 2.35E+05 | 1.57E+05 | 2.55E+05 | 2.28E+05 | 2.01E+05 | 7.88E+05 | 2.98E+05 | 2.48E+05 | 1.37E+05 | 2.48E+05 | 0.36 |
| Com_207_pos | PC (18:5e/2:0) | 4.79E+07 | 1.61E+08 | 1.58E+07 | 3.48E+07 | 3.06E+09 | 1.04E+09 | 3.45E+07 | 7.21E+08 | 4.86E+06 | 6.43E+06 | 0.72 |
| Com_20807_pos | 3-(phenethylethanimidoyl)tetrahydrofuran-2-one | 9.83E+06 | 7.81E+06 | 5.09E+06 | 2.58E+06 | 8.91E+06 | 5.20E+06 | 5.81E+06 | 6.29E+06 | 4.63E+06 | 4.99E+06 | 0.60 |
| Com_2084_pos | (+)-ar-Turmerone | 2.44E+08 | 2.56E+08 | 1.97E+08 | 1.14E+08 | 1.41E+08 | 2.11E+08 | 2.02E+08 | 1.27E+08 | 2.43E+08 | 2.83E+08 | 0.55 |
| Com_208_neg | 2-Ketohexanoic acid | 1.76E+08 | 2.21E+08 | 1.57E+08 | 4.19E+08 | 1.40E+08 | 4.30E+08 | 1.14E+09 | 9.18E+08 | 2.66E+08 | 1.61E+09 | 0.01 |
| Com_2091_neg | Prostaglandin H2 | 7.59E+07 | 6.87E+06 | 5.00E+06 | 3.39E+07 | 1.59E+07 | 2.92E+07 | 3.13E+07 | 1.81E+07 | 2.47E+07 | 3.31E+07 | 0.42 |
| Com_2093_pos | Methylimidazoleacetic acid | 1.99E+08 | 2.46E+08 | 2.61E+08 | 1.72E+08 | 1.49E+08 | 2.17E+08 | 1.91E+08 | 5.69E+07 | 2.65E+08 | 1.64E+08 | 0.45 |
| Com_20963_pos | ethyl 2-[(5-ethoxy-4-phenyl-4H-1,2,4-triazol-3-yl)thio]acetate | 1.39E+06 | 2.48E+06 | 4.14E+06 | 2.33E+06 | 2.09E+06 | 9.11E+06 | 4.21E+06 | 4.46E+06 | 1.61E+06 | 1.88E+06 | 0.31 |
| Com_20978_pos | P-Coumaroyl Agmatine | 5.28E+06 | 4.45E+06 | 2.55E+06 | 3.01E+06 | 8.48E+06 | 4.23E+06 | 3.46E+06 | 1.09E+07 | 1.91E+06 | 2.81E+06 | 0.75 |
| Com_20987_pos | 3-(2-methylpropyl)-octahydropyrrolo[1,2-a]pyrazine-1,4-dione | 1.50E+06 | 1.31E+06 | 1.16E+06 | 9.12E+06 | 1.43E+06 | 4.77E+06 | 1.33E+07 | 8.13E+06 | 4.86E+06 | 3.53E+06 | 0.04 |
| Com_20_neg | Stearic acid | 2.90E+09 | 2.83E+09 | 2.17E+10 | 8.34E+09 | 3.91E+09 | 3.73E+09 | 2.21E+10 | 4.71E+09 | 4.56E+09 | 5.64E+09 | 0.84 |
| Com_2102_pos | Desoxycortone | 5.54E+07 | 2.03E+08 | 6.34E+07 | 1.96E+08 | 7.34E+07 | 2.02E+08 | 2.27E+08 | 7.92E+07 | 2.63E+08 | 1.33E+08 | 0.20 |
| Com_2105_neg | N7-Methylguanosine | 1.08E+07 | 2.21E+07 | 1.78E+07 | 6.83E+07 | 7.00E+07 | 3.64E+07 | 7.95E+07 | 8.48E+07 | 6.16E+06 | 9.32E+07 | 0.56 |
| Com_21129_neg | 5-Hydroxymethyluracil | 1.10E+05 | 9.05E+04 | 1.77E+05 | 8.81E+05 | 9.69E+04 | 1.19E+05 | 2.09E+05 | 1.57E+05 | 2.03E+05 | 1.09E+06 | 0.56 |
| Com_21133_pos | bicyclo[2.2.2]oct-2-en-1-yl 4-methylbenzene-1-sulfonate | 5.90E+06 | 4.29E+06 | 4.65E+06 | 8.46E+06 | 3.70E+06 | 8.98E+06 | 4.49E+06 | 6.04E+06 | 2.96E+06 | 4.65E+06 | 0.94 |
| Com_21137_pos | 4-methylphenyl 1-ethyl-3-methyl-1H-pyrazole-5-carbothioate | 4.48E+06 | 8.88E+06 | 4.40E+06 | 1.81E+06 | 6.30E+06 | 1.39E+06 | 4.81E+06 | 3.75E+06 | 2.55E+06 | 3.72E+06 | 0.25 |
| Com_21175_pos | 4-(cyclohexylmethyl)-6-(2-thienyl)-2,3-dihydropyridazin-3-one hydrate | 2.29E+06 | 2.85E+06 | 2.51E+06 | 3.49E+06 | 3.91E+06 | 7.23E+06 | 3.79E+06 | 1.07E+07 | 1.88E+06 | 1.60E+06 | 0.51 |
| Com_21222_pos | 2-[(3S)-1-(3-Methoxybenzyl)-3-pyrrolidinyl]-1,3-benzothiazole | 5.99E+06 | 6.81E+06 | 5.71E+06 | 4.60E+06 | 5.39E+06 | 5.68E+06 | 5.43E+06 | 3.69E+06 | 9.20E+06 | 5.26E+06 | 0.95 |
| Com_21234_pos | 2-Methylbutyroylcarnitine | 7.21E+06 | 8.81E+06 | 5.15E+06 | 1.96E+06 | 4.23E+06 | 2.21E+06 | 2.29E+06 | 1.92E+06 | 2.85E+06 | 3.77E+06 | 0.07 |
| Com_21263_pos | KPH | 6.62E+06 | 4.26E+06 | 5.12E+06 | 9.29E+06 | 1.24E+06 | 8.88E+06 | 6.18E+06 | 1.12E+06 | 1.83E+06 | 7.11E+06 | 0.79 |
| Com_2127_neg | LPG 14:0 | 2.41E+07 | 4.10E+07 | 1.46E+07 | 2.34E+07 | 6.04E+07 | 5.71E+07 | 4.73E+07 | 3.20E+07 | 3.90E+06 | 6.33E+07 | 0.99 |
| Com_21283_pos | YLK | 2.30E+06 | 4.02E+06 | 1.06E+06 | 6.61E+06 | 6.58E+06 | 5.00E+06 | 5.16E+06 | 1.06E+07 | 2.22E+06 | 6.95E+06 | 0.32 |
| Com_21362_pos | Indole-3-pyruvic acid | 3.13E+06 | 5.45E+06 | 2.22E+06 | 2.46E+06 | 4.29E+06 | 2.74E+06 | 4.11E+06 | 1.06E+07 | 3.50E+06 | 7.02E+06 | 0.22 |
| Com_213_neg | L-Histidine | 7.11E+07 | 1.39E+08 | 4.74E+07 | 1.12E+08 | 6.60E+08 | 1.61E+08 | 5.76E+07 | 1.58E+09 | 2.88E+07 | 1.39E+08 | 0.90 |
| Com_21421_pos | Ecdysterone | 1.07E+06 | 2.70E+06 | 3.75E+05 | 3.93E+06 | 9.34E+05 | 8.77E+06 | 1.07E+06 | 9.49E+05 | 8.55E+05 | 1.65E+06 | 0.71 |
| Com_21433_pos | Glycitin | 2.99E+05 | 3.25E+05 | 4.41E+05 | 5.03E+06 | 4.23E+05 | 3.62E+06 | 5.12E+06 | 4.45E+05 | 9.04E+06 | 5.19E+06 | 0.05 |
| Com_21477_pos | VMH | 4.58E+06 | 5.17E+06 | 4.77E+05 | 3.98E+06 | 3.41E+06 | 2.12E+06 | 1.54E+06 | 1.05E+07 | 1.56E+06 | 6.44E+06 | 0.79 |
| Com_21504_pos | N-(p-Coumaroyl) serotonin | 6.89E+06 | 4.41E+06 | 6.40E+05 | 9.05E+05 | 3.69E+06 | 6.94E+06 | 7.55E+06 | 1.04E+07 | 3.64E+06 | 1.73E+06 | 0.20 |
| Com_2151_pos | Caffeic acid | 3.26E+07 | 2.17E+07 | 3.02E+08 | 2.62E+07 | 2.75E+07 | 2.00E+07 | 3.36E+07 | 3.81E+07 | 2.48E+07 | 3.29E+07 | 0.47 |
| Com_2155_pos | 5α-Pregnan-3,20-dione | 2.34E+08 | 1.06E+08 | 4.61E+07 | 2.73E+08 | 1.96E+08 | 2.00E+08 | 2.01E+08 | 1.67E+08 | 1.87E+08 | 2.06E+08 | 0.43 |
| Com_21564_pos | Gatifloxacin | 5.71E+05 | 1.56E+06 | 1.97E+05 | 1.25E+06 | 4.02E+06 | 6.15E+06 | 1.64E+06 | 1.04E+07 | 1.01E+06 | 1.58E+06 | 0.16 |
| Com_2157_neg | 3,4,5-trihydroxycyclohex-1-ene-1-carboxylic acid | 4.18E+07 | 5.21E+07 | 5.30E+07 | 2.14E+07 | 7.64E+07 | 2.68E+07 | 5.35E+07 | 4.91E+07 | 3.28E+07 | 2.86E+07 | 0.44 |
| Com_215_neg | Monobenzyl phthalate | 8.18E+07 | 1.43E+08 | 9.43E+08 | 6.30E+07 | 1.71E+08 | 9.49E+08 | 1.95E+08 | 3.20E+08 | 8.86E+06 | 2.53E+08 | 0.98 |
| Com_21609_pos | 3-hydroxy-3,4-bis[(4-hydroxy-3-methoxyphenyl)methyl]oxolan-2-one | 8.93E+05 | 1.56E+06 | 7.86E+05 | 3.69E+06 | 9.32E+05 | 4.53E+06 | 1.26E+07 | 6.43E+06 | 2.05E+06 | 5.82E+06 | 0.01 |
| Com_2161_pos | Palmitoleic Acid | 2.54E+08 | 1.93E+08 | 2.04E+08 | 1.19E+08 | 2.21E+08 | 1.11E+08 | 2.84E+08 | 2.78E+08 | 1.16E+08 | 1.87E+08 | 0.79 |
| Com_2163_neg | 19(R)-Hydroxy-prostaglandin E2 | 7.32E+07 | 5.48E+06 | 5.50E+06 | 6.59E+06 | 8.14E+06 | 6.77E+06 | 1.53E+07 | 5.01E+06 | 6.18E+06 | 5.16E+06 | 0.49 |
| Com_21661_pos | APK | 1.00E+06 | 9.12E+05 | 7.53E+05 | 5.23E+06 | 4.46E+06 | 8.58E+06 | 5.81E+06 | 1.82E+06 | 2.57E+06 | 4.89E+06 | 0.14 |
| Com_2169_pos | Glycyl-L-leucine | 7.43E+07 | 1.70E+08 | 2.21E+07 | 1.72E+08 | 1.63E+08 | 1.20E+08 | 1.15E+08 | 2.95E+08 | 3.55E+07 | 2.18E+08 | 0.62 |
| Com_2171_pos | Quinoline | 1.07E+07 | 1.77E+07 | 3.51E+06 | 2.71E+08 | 1.99E+07 | 3.23E+07 | 1.92E+07 | 6.31E+06 | 8.74E+07 | 4.86E+06 | 0.87 |
| Com_2172_neg | Glutaconic acid | 6.42E+06 | 4.82E+06 | 4.69E+06 | 3.83E+06 | 4.55E+06 | 5.58E+07 | 1.04E+07 | 8.88E+06 | 4.46E+06 | 4.39E+06 | 0.19 |
| Com_21752_pos | Vindoline | 3.77E+05 | 3.88E+05 | 4.17E+05 | 4.69E+05 | 3.81E+05 | 3.50E+05 | 4.61E+05 | 4.34E+05 | 8.78E+06 | 5.24E+05 | 0.32 |
| Com_217_pos | 8-Hydroxyquinoline | 1.79E+09 | 1.94E+09 | 3.74E+09 | 2.05E+09 | 2.75E+09 | 2.61E+09 | 3.20E+09 | 1.88E+09 | 3.00E+09 | 1.77E+09 | 0.89 |
| Com_21807_neg | N-acetyl-D-glucosamine | 3.18E+05 | 1.47E+05 | 3.11E+05 | 1.20E+05 | 5.54E+05 | 3.63E+05 | 3.49E+05 | 2.91E+05 | 3.60E+05 | 1.94E+05 | 0.54 |
| Com_2183_neg | LPI 20:4 | 1.65E+06 | 5.26E+07 | 8.94E+05 | 7.73E+05 | 1.07E+07 | 4.31E+05 | 7.90E+05 | 2.47E+07 | 4.36E+05 | 6.53E+05 | 0.35 |
| Com_2183_pos | Jasmonic acid | 1.40E+08 | 8.16E+07 | 7.00E+07 | 2.03E+08 | 1.90E+08 | 7.90E+07 | 2.46E+08 | 2.28E+08 | 2.51E+08 | 2.10E+08 | 0.22 |
| Com_218_neg | Dodecanedioic acid | 8.94E+08 | 8.86E+08 | 1.35E+09 | 7.11E+08 | 1.05E+09 | 3.53E+08 | 1.15E+09 | 2.20E+08 | 1.26E+08 | 9.23E+08 | 0.11 |
| Com_21936_pos | 3-Indoleacrylic acid | 5.68E+06 | 4.81E+06 | 3.03E+06 | 6.55E+06 | 6.11E+06 | 6.15E+06 | 6.71E+06 | 1.00E+07 | 5.28E+06 | 6.79E+06 | 0.13 |
| Com_219_neg | Methyl-beta-galactopyranoside | 2.49E+08 | 3.58E+08 | 4.36E+08 | 3.45E+08 | 8.06E+08 | 6.26E+08 | 9.45E+08 | 1.52E+09 | 1.78E+08 | 4.68E+08 | 0.38 |
| Com_22011_pos | ethyl 2-{[5-(5-nitro-2-furyl)-1,3,4-oxadiazol-2-yl]thio}acetate | 2.08E+06 | 5.99E+06 | 1.33E+06 | 5.93E+05 | 5.97E+06 | 4.09E+06 | 4.77E+05 | 9.97E+06 | 3.58E+05 | 1.57E+06 | 0.68 |
| Com_22041_pos | 5,8-dihydroxy-10-methyl-5,8,9,10-tetrahydro-2H-oxecin-2-one | 5.32E+06 | 8.21E+06 | 8.89E+06 | 2.98E+06 | 3.94E+06 | 4.16E+06 | 8.17E+06 | 1.95E+06 | 6.82E+06 | 1.07E+06 | 0.34 |
| Com_22070_pos | 3-Methylhippuric acid | 3.53E+06 | 8.19E+06 | 4.19E+06 | 3.29E+06 | 2.38E+06 | 6.44E+06 | 4.76E+06 | 2.74E+06 | 5.40E+06 | 4.37E+06 | 0.57 |
| Com_22071_pos | Catharanthine | 1.43E+06 | 1.38E+06 | 4.74E+05 | 4.20E+06 | 4.72E+06 | 8.28E+06 | 4.43E+06 | 2.07E+06 | 2.59E+06 | 4.57E+06 | 0.16 |
| Com_22083_pos | Diflorasone | 1.69E+06 | 8.06E+05 | 1.12E+06 | 9.40E+05 | 1.38E+06 | 8.27E+06 | 1.28E+06 | 1.02E+06 | 1.17E+06 | 1.16E+06 | 0.38 |
| Com_220_pos | FMH | 1.17E+09 | 1.21E+09 | 2.23E+09 | 7.34E+06 | 8.10E+08 | 8.05E+08 | 1.67E+09 | 1.03E+09 | 3.15E+09 | 2.76E+09 | 0.29 |
| Com_2211_neg | PC (14:0e/2:0) | 3.46E+07 | 5.19E+07 | 3.92E+06 | 2.28E+07 | 1.09E+07 | 3.41E+07 | 8.93E+06 | 2.43E+07 | 2.46E+06 | 3.29E+07 | 0.76 |
| Com_22146_pos | N-[(4-hydroxy-3-methoxyphenyl)methyl]-8-methylnonanamide | 6.35E+06 | 8.14E+06 | 6.63E+06 | 4.60E+06 | 7.00E+06 | 2.89E+06 | 4.98E+06 | 4.23E+06 | 4.73E+06 | 6.17E+06 | 0.05 |
| Com_2214_neg | 2-Isopropylmalic acid | 1.34E+07 | 2.08E+07 | 2.51E+07 | 2.21E+07 | 3.72E+07 | 1.49E+07 | 1.20E+08 | 3.20E+07 | 3.41E+07 | 1.92E+07 | 0.38 |
| Com_2217_pos | (2E,4E)-N-(2-methylpropyl)deca-2,4-dienamide | 1.75E+08 | 1.83E+08 | 2.89E+08 | 1.49E+08 | 2.04E+08 | 1.14E+08 | 2.61E+08 | 2.03E+08 | 1.23E+08 | 1.57E+08 | 0.38 |
| Com_22181_pos | Phenylacetylglycine | 4.41E+06 | 4.98E+06 | 9.96E+06 | 4.00E+06 | 7.48E+06 | 5.49E+06 | 6.16E+06 | 7.42E+06 | 5.80E+06 | 4.30E+06 | 0.97 |
| Com_221_neg | Equol | 1.18E+09 | 8.50E+08 | 2.32E+06 | 9.28E+08 | 6.46E+08 | 3.07E+08 | 4.59E+08 | 1.82E+08 | 2.15E+08 | 2.80E+08 | 0.99 |
| Com_2222_pos | Caprolactam | 1.60E+08 | 2.35E+08 | 2.24E+08 | 1.29E+08 | 1.55E+08 | 1.02E+08 | 2.49E+08 | 2.17E+08 | 1.25E+08 | 1.29E+08 | 0.55 |
| Com_2223_pos | Boc-beta-cyano-L-alanine | 1.57E+08 | 2.35E+08 | 1.57E+08 | 1.10E+08 | 1.74E+08 | 1.64E+08 | 1.45E+08 | 9.56E+07 | 2.20E+08 | 1.75E+08 | 0.80 |
| Com_22277_pos | Boldione | 7.53E+06 | 8.06E+06 | 5.72E+06 | 4.91E+06 | 6.72E+06 | 5.38E+06 | 4.47E+06 | 2.08E+06 | 8.04E+06 | 5.26E+06 | 0.22 |
| Com_2228_neg | N-Acetyl-DL-norvaline | 1.62E+07 | 2.72E+07 | 1.47E+07 | 7.86E+07 | 2.36E+07 | 3.24E+07 | 1.77E+07 | 1.60E+07 | 8.55E+06 | 1.67E+07 | 0.26 |
| Com_2229_neg | Ursolic acid | 1.64E+07 | 5.16E+07 | 3.37E+07 | 1.73E+07 | 1.32E+07 | 2.04E+07 | 3.61E+07 | 7.71E+06 | 3.69E+07 | 5.43E+07 | 0.80 |
| Com_222_neg | D-Proline | 2.78E+08 | 3.75E+08 | 8.06E+08 | 7.77E+08 | 1.02E+09 | 8.07E+08 | 1.02E+09 | 1.46E+09 | 2.86E+08 | 8.53E+08 | 0.45 |
| Com_22303_pos | 2,6-Xylidine | 5.10E+06 | 8.05E+06 | 5.51E+06 | 5.04E+06 | 4.12E+06 | 3.37E+06 | 5.36E+06 | 6.49E+06 | 6.36E+06 | 5.67E+06 | 0.90 |
| Com_2233_neg | Dihydroroseoside | 6.59E+05 | 3.94E+05 | 5.90E+05 | 1.29E+06 | 5.34E+05 | 5.42E+07 | 7.41E+05 | 1.15E+06 | 3.31E+05 | 1.94E+06 | 0.27 |
| Com_22353_neg | 5-Phenylvaleric Acid | 2.43E+04 | 4.23E+04 | 6.37E+04 | 3.26E+04 | 5.14E+04 | 2.74E+04 | 6.84E+04 | 1.42E+05 | 2.54E+04 | 5.73E+04 | 0.51 |
| Com_22354_pos | Anserine | 3.49E+06 | 5.15E+06 | 9.21E+06 | 3.75E+06 | 7.04E+06 | 4.49E+06 | 9.72E+06 | 9.71E+06 | 6.74E+06 | 6.32E+06 | 0.26 |
| Com_22355_pos | ENK | 9.53E+05 | 2.20E+06 | 8.64E+05 | 8.91E+06 | 2.66E+06 | 2.53E+06 | 5.72E+06 | 2.25E+06 | 8.03E+05 | 5.24E+06 | 0.68 |
| Com_22396_pos | 3-ethyl-4-hydroxy-1-methyl-1,2-dihydroquinolin-2-one | 7.50E+06 | 1.08E+06 | 7.67E+05 | 8.53E+05 | 1.66E+06 | 8.07E+06 | 1.20E+06 | 1.85E+06 | 9.30E+05 | 9.94E+05 | 0.83 |
| Com_2242_pos | Indole | 2.48E+08 | 1.88E+08 | 3.77E+07 | 2.02E+08 | 2.26E+08 | 1.11E+08 | 7.42E+07 | 2.01E+08 | 1.05E+08 | 1.17E+08 | 0.51 |
| Com_22536_pos | 6-Methoxy-2-naphthoic acid | 7.62E+06 | 7.89E+06 | 1.40E+06 | 3.58E+06 | 5.56E+06 | 4.22E+06 | 3.72E+06 | 4.15E+06 | 7.28E+06 | 3.48E+06 | 0.99 |
| Com_22556_pos | Nervonic acid | 1.02E+07 | 6.31E+06 | 6.85E+06 | 4.79E+06 | 1.37E+06 | 2.19E+06 | 3.59E+06 | 2.71E+06 | 2.97E+06 | 3.24E+06 | 0.20 |
| Com_22557_pos | 5-Fluoro-2-[(3S)-1-(2-methylbenzyl)-3-pyrrolidinyl]-1H-benzimidazole | 1.72E+06 | 3.00E+06 | 2.99E+06 | 8.76E+06 | 4.35E+06 | 5.16E+06 | 3.24E+06 | 2.99E+06 | 1.17E+06 | 4.94E+06 | 0.72 |
| Com_2259_neg | Prostaglandin K2 | 4.53E+07 | 2.40E+07 | 2.03E+07 | 2.32E+07 | 2.01E+07 | 5.33E+07 | 1.43E+07 | 8.18E+06 | 1.64E+07 | 2.79E+07 | 0.49 |
| Com_22663_pos | N1-(2,3-dihydro-1,4-benzodioxin-2-ylmethyl)-2,2-dimethylpropanamide | 5.69E+06 | 7.81E+06 | 6.49E+06 | 4.75E+06 | 4.33E+06 | 2.57E+06 | 3.03E+06 | 2.98E+06 | 1.99E+06 | 5.66E+06 | 0.02 |
| Com_22724_pos | IMH | 6.57E+06 | 7.77E+06 | 7.84E+06 | 5.67E+06 | 4.82E+06 | 6.36E+06 | 3.93E+06 | 3.86E+06 | 2.93E+06 | 4.07E+06 | 0.02 |
| Com_2279_pos | Kynurenic acid | 1.59E+08 | 1.76E+08 | 2.81E+08 | 6.00E+07 | 1.79E+08 | 9.09E+07 | 1.35E+08 | 7.35E+07 | 7.44E+07 | 6.78E+07 | 0.09 |
| Com_2293_pos | Guanine | 9.58E+07 | 1.98E+08 | 6.39E+07 | 4.78E+07 | 1.93E+08 | 9.83E+07 | 3.42E+07 | 1.96E+08 | 1.39E+08 | 2.69E+08 | 0.74 |
| Com_22966_pos | (4-methoxyphenyl)(4-nitrophenyl)methanone | 1.18E+06 | 2.43E+06 | 8.20E+05 | 8.41E+05 | 2.56E+06 | 1.50E+06 | 8.79E+06 | 9.21E+06 | 1.54E+06 | 2.02E+06 | 0.12 |
| Com_229_neg | acetoacetate | 7.21E+07 | 9.61E+07 | 4.32E+08 | 1.26E+09 | 1.83E+08 | 8.11E+08 | 7.62E+08 | 6.47E+08 | 3.14E+08 | 1.07E+09 | 0.12 |
| Com_23011_pos | 2-{[2-(4-methylpiperazino)phenyl]methylene}hydrazine-1-carbothioamide | 4.22E+06 | 7.57E+06 | 2.58E+06 | 3.86E+06 | 4.99E+06 | 5.68E+06 | 4.67E+06 | 3.61E+06 | 3.74E+06 | 1.22E+06 | 0.45 |
| Com_23013_pos | N-gamma-Acetyl-N-2-Formyl-5-Methoxykynurenamine | 2.28E+06 | 3.10E+06 | 1.42E+06 | 8.42E+06 | 1.72E+06 | 1.15E+06 | 1.89E+06 | 1.61E+06 | 2.66E+06 | 2.84E+06 | 0.37 |
| Com_23065_pos | glutathione disulfide | 1.97E+06 | 5.87E+06 | 1.89E+06 | 1.62E+06 | 4.14E+06 | 2.21E+06 | 4.72E+06 | 9.13E+06 | 1.86E+06 | 2.37E+06 | 0.60 |
| Com_2313_pos | 4-Methyl-5-thiazoleethanol | 5.35E+07 | 1.64E+08 | 1.50E+08 | 1.80E+08 | 4.68E+07 | 3.17E+07 | 3.84E+07 | 4.05E+07 | 2.35E+08 | 5.56E+07 | 0.26 |
| Com_2318_pos | 8Z,11Z,14Z-Eicosatrienoic acid | 4.20E+07 | 2.45E+07 | 1.42E+07 | 2.50E+08 | 1.53E+07 | 6.88E+07 | 1.38E+08 | 6.62E+07 | 2.25E+08 | 1.42E+08 | 0.09 |
| Com_23269_pos | O-Desmethylnaproxen | 3.05E+06 | 1.45E+06 | 1.25E+06 | 1.35E+06 | 3.88E+05 | 7.48E+06 | 3.98E+06 | 5.37E+06 | 1.29E+06 | 6.58E+06 | 0.03 |
| Com_232_neg | LPG 16:0 | 3.62E+06 | 2.39E+08 | 2.56E+06 | 4.24E+08 | 2.55E+08 | 1.12E+06 | 7.37E+06 | 1.41E+09 | 4.37E+06 | 1.68E+08 | 0.71 |
| Com_2332_pos | D-Glucuronic acid | 2.54E+06 | 2.14E+06 | 3.97E+06 | 3.03E+06 | 3.46E+06 | 2.26E+08 | 6.97E+06 | 4.36E+06 | 2.98E+06 | 2.04E+07 | 0.13 |
| Com_2333_neg | 9-HpOTrE | 2.31E+07 | 4.79E+07 | 4.35E+07 | 3.25E+07 | 3.52E+07 | 3.81E+07 | 7.33E+07 | 5.83E+07 | 4.75E+07 | 4.27E+07 | 0.07 |
| Com_23482_pos | N4-Acetylcytidine | 1.92E+06 | 2.90E+06 | 2.47E+06 | 8.09E+06 | 1.58E+06 | 9.02E+05 | 4.75E+06 | 3.62E+06 | 1.53E+06 | 7.80E+06 | 0.98 |
| Com_2348_pos | UR-144 N-Heptyl analog | 3.37E+07 | 2.06E+07 | 2.96E+07 | 2.85E+07 | 2.16E+08 | 2.45E+07 | 6.73E+07 | 1.18E+08 | 1.18E+08 | 3.00E+07 | 0.54 |
| Com_2352_neg | N-Acetylhistidine | 2.22E+07 | 3.61E+07 | 9.07E+07 | 4.58E+07 | 5.95E+07 | 3.42E+07 | 3.06E+07 | 7.85E+07 | 2.31E+07 | 2.37E+07 | 0.39 |
| Com_2356_neg | N-{4-methyl-3-[(2-toluidinocarbothioyl)amino]phenyl}methanesulfonamide | 2.38E+05 | 1.83E+05 | 5.58E+05 | 5.06E+05 | 3.52E+07 | 5.06E+07 | 4.06E+05 | 2.79E+07 | 1.69E+05 | 3.00E+05 | 0.60 |
| Com_235_pos | Apocynin | 2.10E+07 | 8.09E+07 | 1.62E+07 | 1.52E+09 | 1.57E+08 | 1.62E+09 | 3.22E+09 | 1.27E+09 | 2.94E+09 | 2.25E+09 | 0.02 |
| Com_23647_pos | Ip7G | 7.75E+05 | 8.10E+05 | 7.79E+05 | 1.00E+06 | 5.94E+05 | 7.25E+06 | 7.28E+05 | 1.54E+06 | 8.55E+05 | 9.51E+05 | 0.21 |
| Com_23663_pos | 2-methyl-6-{[(5-phenyl-2-thienyl)carbonyl]amino}benzoic acid | 1.45E+06 | 4.73E+06 | 8.79E+06 | 3.68E+06 | 4.82E+06 | 4.08E+06 | 5.12E+06 | 5.22E+06 | 7.22E+06 | 2.27E+06 | 0.78 |
| Com_23696_pos | 3-[(4-chlorophenyl)thio]-1-phenylprop-2-en-1-one | 5.40E+05 | 6.13E+05 | 8.93E+05 | 1.47E+06 | 7.52E+05 | 4.91E+05 | 5.29E+06 | 1.01E+06 | 3.13E+06 | 8.44E+06 | 0.11 |
| Com_2373_neg | 3-Hydroxyanthranilic Acid | 1.96E+07 | 1.52E+07 | 1.87E+07 | 3.87E+07 | 4.25E+07 | 3.01E+07 | 7.66E+07 | 3.03E+07 | 4.65E+07 | 7.04E+07 | 0.06 |
| Com_23782_pos | 3-methyl-1-benzothiophene-2-carbaldehyde oxime | 8.52E+06 | 3.75E+06 | 1.16E+06 | 2.27E+06 | 6.90E+06 | 1.51E+06 | 1.40E+06 | 1.45E+06 | 4.29E+06 | 4.47E+06 | 0.35 |
| Com_23850_pos | MLK | 1.41E+06 | 2.22E+06 | 4.38E+05 | 7.83E+06 | 3.18E+06 | 3.45E+06 | 3.49E+06 | 4.18E+06 | 1.89E+06 | 5.47E+06 | 0.33 |
| Com_2389_pos | Glycerol 1-hexadecanoate | 1.22E+08 | 4.50E+07 | 8.95E+07 | 8.19E+07 | 2.12E+08 | 1.18E+08 | 2.71E+07 | 8.78E+07 | 5.18E+07 | 8.87E+07 | 0.33 |
| Com_238_neg | Propylparaben | 2.92E+06 | 7.97E+06 | 4.13E+06 | 7.92E+07 | 2.68E+06 | 5.73E+07 | 6.49E+08 | 1.01E+08 | 7.87E+08 | 6.87E+08 | 0.00 |
| Com_23903_pos | N1-(2-oxoazepan-3-yl)-N2-(3-pyridylmethyl)ethanediamide | 6.16E+06 | 7.02E+06 | 1.27E+06 | 3.47E+06 | 5.00E+06 | 1.96E+06 | 2.08E+06 | 3.10E+06 | 3.31E+06 | 3.64E+06 | 0.32 |
| Com_2392_pos | Octanedioic acid | 2.80E+08 | 1.76E+08 | 1.48E+08 | 1.07E+08 | 1.18E+08 | 8.32E+07 | 1.74E+08 | 5.98E+07 | 1.69E+08 | 1.38E+08 | 0.30 |
| Com_23_neg | Docosahexaenoic acid | 5.27E+09 | 6.36E+09 | 4.60E+09 | 1.14E+10 | 1.13E+10 | 3.68E+09 | 9.99E+09 | 1.60E+10 | 3.59E+09 | 5.25E+09 | 0.75 |
| Com_23_pos | L-Glutamic acid | 1.07E+10 | 1.03E+10 | 3.36E+10 | 2.48E+10 | 9.51E+09 | 1.50E+10 | 1.84E+10 | 1.23E+10 | 3.09E+10 | 3.21E+10 | 0.43 |
| Com_24016_pos | L-(+)-Arabinose | 3.31E+06 | 6.95E+06 | 2.88E+06 | 5.71E+06 | 1.17E+06 | 2.09E+06 | 4.30E+06 | 2.75E+06 | 3.42E+06 | 5.16E+06 | 0.99 |
| Com_24101_pos | Glycocholic acid hydrate | 8.96E+05 | 6.88E+05 | 1.61E+06 | 8.35E+05 | 1.04E+06 | 6.98E+06 | 1.35E+06 | 9.25E+05 | 8.30E+05 | 9.78E+05 | 0.36 |
| Com_2410_pos | Heptadecanoic Acid | 1.76E+08 | 9.31E+07 | 1.18E+08 | 1.39E+08 | 2.10E+08 | 5.21E+07 | 7.92E+07 | 1.31E+08 | 1.33E+08 | 8.73E+07 | 0.09 |
| Com_24157_pos | 2-((Dimethylamino)methyl)phenol | 1.12E+06 | 2.01E+06 | 1.46E+06 | 7.64E+06 | 1.93E+06 | 6.00E+06 | 2.55E+06 | 6.80E+06 | 1.31E+06 | 2.35E+06 | 0.43 |
| Com_24182_pos | (±)8-HEPE | 2.25E+06 | 2.06E+06 | 3.56E+06 | 2.95E+06 | 2.84E+06 | 2.30E+06 | 1.02E+07 | 3.37E+06 | 2.80E+06 | 3.25E+06 | 0.29 |
| Com_2426_pos | 5α-Dihydrotestosterone | 6.47E+07 | 4.97E+07 | 4.00E+07 | 2.38E+08 | 1.07E+08 | 7.05E+07 | 8.49E+07 | 7.02E+07 | 5.14E+07 | 6.33E+07 | 0.62 |
| Com_2427_neg | 10-Nitrolinoleate | 3.23E+06 | 1.49E+07 | 1.31E+06 | 5.05E+06 | 2.19E+07 | 2.92E+07 | 4.90E+07 | 7.92E+07 | 9.80E+05 | 5.21E+06 | 0.39 |
| Com_2433_pos | 17α-Ethynylestradiol | 5.67E+07 | 4.40E+07 | 1.81E+07 | 1.03E+07 | 5.48E+07 | 2.16E+08 | 1.93E+07 | 6.26E+07 | 6.88E+07 | 4.43E+07 | 0.22 |
| Com_24356_pos | Glu-Val-Phe | 3.04E+06 | 4.28E+06 | 2.45E+06 | 5.23E+06 | 4.12E+06 | 6.45E+06 | 9.29E+06 | 6.20E+06 | 3.80E+06 | 7.96E+06 | 0.03 |
| Com_24387_pos | TKK | 3.44E+06 | 4.35E+06 | 2.47E+06 | 7.48E+06 | 5.23E+06 | 4.70E+06 | 4.55E+06 | 4.28E+06 | 2.50E+06 | 6.72E+06 | 0.96 |
| Com_24438_pos | Tyr-Tyr | 5.43E+05 | 1.96E+06 | 3.04E+05 | 1.55E+06 | 1.34E+06 | 6.77E+06 | 5.32E+05 | 2.24E+06 | 3.08E+05 | 2.35E+06 | 0.53 |
| Com_2443_pos | Asparagine | 4.19E+07 | 1.91E+07 | 1.58E+07 | 1.88E+08 | 1.79E+08 | 1.73E+08 | 3.15E+08 | 1.88E+08 | 5.60E+07 | 1.57E+08 | 0.12 |
| Com_24441_pos | PLH | 2.12E+06 | 6.70E+06 | 2.03E+05 | 2.09E+05 | 1.93E+05 | 3.70E+06 | 2.07E+05 | 2.26E+05 | 2.67E+05 | 2.31E+05 | 0.62 |
| Com_24476_pos | N-acetyl-L-ornithine | 1.35E+06 | 1.82E+06 | 1.28E+06 | 4.95E+06 | 3.57E+06 | 2.22E+06 | 7.67E+06 | 6.58E+06 | 6.96E+06 | 1.45E+06 | 0.21 |
| Com_24486_pos | TNK | 1.79E+06 | 3.52E+06 | 1.15E+06 | 4.13E+06 | 4.39E+06 | 4.82E+06 | 4.76E+06 | 8.07E+06 | 4.16E+06 | 4.27E+06 | 0.07 |
| Com_2462_pos | LPE 22:6 | 2.88E+07 | 3.82E+07 | 1.17E+07 | 1.48E+07 | 1.15E+08 | 2.13E+08 | 9.05E+07 | 9.36E+07 | 3.48E+06 | 1.58E+07 | 0.73 |
| Com_24641_pos | Quinoline-4-carboxylic acid | 4.24E+06 | 6.56E+06 | 5.51E+06 | 2.45E+06 | 2.32E+06 | 2.05E+06 | 4.60E+06 | 1.25E+06 | 6.66E+06 | 3.97E+06 | 0.59 |
| Com_2464_pos | Gly-Phe | 3.76E+07 | 8.58E+07 | 5.02E+06 | 6.08E+07 | 1.26E+08 | 6.34E+07 | 3.24E+07 | 2.55E+08 | 9.28E+06 | 8.67E+07 | 0.76 |
| Com_24671_pos | Bisphenol M | 4.29E+06 | 6.03E+06 | 7.72E+06 | 4.56E+06 | 4.84E+06 | 4.01E+06 | 5.13E+06 | 3.11E+06 | 6.82E+06 | 7.36E+06 | 0.75 |
| Com_24702_pos | Taurodeoxycholic Acid | 1.05E+06 | 4.70E+05 | 5.21E+06 | 3.17E+06 | 8.06E+05 | 2.84E+06 | 1.52E+06 | 1.45E+06 | 9.20E+05 | 7.70E+06 | 0.53 |
| Com_24704_pos | 6-phenyl-1,2,3,4-tetrahydro-2,5-benzodiazocin-1-one | 2.84E+06 | 2.66E+06 | 8.00E+06 | 2.69E+06 | 2.61E+06 | 1.26E+06 | 3.34E+06 | 9.49E+05 | 5.20E+06 | 3.97E+06 | 0.44 |
| Com_24709_pos | PPH | 3.15E+06 | 1.48E+06 | 4.20E+06 | 2.06E+06 | 3.62E+06 | 5.26E+06 | 2.75E+06 | 7.90E+06 | 2.03E+06 | 3.28E+06 | 0.32 |
| Com_24869_pos | 4-Hexyloxyaniline | 4.29E+06 | 6.43E+06 | 5.82E+06 | 3.07E+06 | 3.49E+06 | 2.08E+06 | 2.15E+06 | 2.70E+06 | 3.01E+06 | 4.26E+06 | 0.04 |
| Com_2487_neg | Prostaglandin H1 | 6.10E+07 | 1.16E+07 | 6.72E+06 | 4.68E+06 | 1.24E+07 | 5.24E+06 | 4.05E+06 | 4.46E+06 | 5.33E+06 | 1.76E+07 | 0.23 |
| Com_24929_pos | 2-(2,6-dimethoxyphenyl)-5,6-dimethoxy-4H-chromen-4-one | 4.58E+06 | 1.29E+06 | 9.80E+05 | 1.67E+06 | 1.20E+06 | 4.03E+06 | 9.47E+06 | 3.18E+06 | 2.08E+06 | 1.86E+06 | 0.10 |
| Com_2497_pos | LPC 14:0 | 2.05E+07 | 6.79E+07 | 1.88E+06 | 1.03E+07 | 1.46E+08 | 2.09E+08 | 5.80E+06 | 7.57E+07 | 1.81E+06 | 3.20E+07 | 0.96 |
| Com_2498_pos | Palmitoylcarnitine | 1.01E+07 | 6.63E+07 | 1.97E+06 | 8.80E+05 | 6.76E+07 | 1.33E+08 | 1.40E+06 | 2.50E+08 | 9.69E+05 | 2.37E+06 | 0.97 |
| Com_24_pos | Ursodeoxycholic acid | 3.54E+10 | 2.81E+10 | 2.04E+10 | 1.94E+10 | 2.50E+10 | 2.32E+10 | 8.35E+09 | 2.61E+09 | 3.04E+10 | 3.34E+10 | 0.30 |
| Com_25038_pos | Indoxylsulfuric acid | 2.55E+06 | 2.97E+06 | 2.76E+06 | 3.23E+06 | 3.11E+06 | 5.37E+06 | 3.44E+06 | 1.77E+06 | 6.60E+06 | 4.14E+06 | 0.27 |
| Com_2505_neg | LPI 18:1 | 6.58E+06 | 4.40E+07 | 4.63E+06 | 7.93E+06 | 4.50E+06 | 1.47E+06 | 1.02E+07 | 6.62E+06 | 6.66E+06 | 5.19E+06 | 0.36 |
| Com_2518_pos | 7-(2-hydroxypropan-2-yl)-1,4a-dimethyl-decahydronaphthalen-1-ol | 6.96E+07 | 4.98E+07 | 1.50E+08 | 2.27E+08 | 6.89E+07 | 6.15E+07 | 8.23E+07 | 9.98E+07 | 1.13E+08 | 1.03E+08 | 0.84 |
| Com_25191_pos | 2-(4-aminophenoxy)isophthalonitrile | 1.08E+06 | 8.22E+05 | 1.35E+06 | 1.00E+06 | 1.44E+06 | 6.31E+06 | 2.59E+06 | 3.38E+06 | 9.42E+05 | 1.12E+06 | 0.12 |
| Com_251_pos | 5-[(10Z)-14-(3,5-dihydroxyphenyl)tetradec-10-en-1-yl]benzene-1,3-diol | 2.55E+09 | 2.70E+09 | 1.52E+09 | 9.53E+08 | 1.46E+09 | 1.10E+09 | 1.59E+09 | 1.32E+08 | 2.63E+09 | 2.04E+09 | 0.43 |
| Com_2520_neg | 18-β-Glycyrrhetinic acid | 4.83E+07 | 4.37E+07 | 4.28E+06 | 7.41E+06 | 3.72E+07 | 2.07E+07 | 6.65E+06 | 2.15E+07 | 5.56E+06 | 7.79E+06 | 0.35 |
| Com_2521_neg | Ascorbic acid | 3.13E+07 | 1.79E+07 | 7.22E+07 | 6.65E+07 | 4.30E+07 | 2.86E+07 | 4.13E+07 | 2.75E+07 | 3.03E+07 | 6.79E+07 | 0.73 |
| Com_2521_pos | Allolithocholic acid | 1.29E+06 | 9.35E+07 | 4.25E+07 | 1.34E+07 | 2.83E+07 | 4.14E+07 | 1.62E+07 | 3.68E+06 | 2.13E+08 | 1.33E+08 | 0.51 |
| Com_25256_pos | Tetracycline | 1.67E+06 | 3.46E+06 | 9.93E+05 | 3.39E+06 | 1.29E+06 | 6.27E+06 | 2.34E+06 | 2.30E+06 | 3.89E+06 | 2.04E+06 | 0.19 |
| Com_25308_pos | 5,6-dimethyl-4-oxo-4H-pyran-2-carboxylic acid | 5.68E+06 | 3.53E+06 | 3.02E+06 | 4.54E+06 | 6.01E+06 | 5.37E+06 | 5.54E+06 | 4.98E+06 | 2.72E+06 | 3.78E+06 | 0.93 |
| Com_25408_pos | Retrorsine | 3.97E+05 | 1.39E+06 | 3.32E+05 | 3.28E+06 | 3.27E+05 | 2.83E+05 | 3.98E+05 | 4.03E+05 | 6.39E+06 | 5.01E+05 | 0.94 |
| Com_2543_pos | 1-(3,4-dihydroxyphenyl)-7-(4-hydroxyphenyl)heptan-3-one | 1.48E+08 | 2.01E+08 | 1.47E+08 | 2.23E+08 | 5.93E+07 | 1.95E+08 | 1.23E+08 | 6.20E+07 | 1.93E+08 | 2.14E+08 | 0.97 |
| Com_2553_neg | 2-Deoxyuridine | 8.22E+06 | 1.87E+07 | 1.54E+07 | 5.57E+06 | 3.48E+07 | 2.85E+07 | 2.44E+07 | 7.39E+07 | 8.59E+06 | 8.20E+06 | 0.45 |
| Com_2556_neg | N-Acetyl-L-phenylalanine | 3.19E+06 | 5.01E+06 | 9.82E+06 | 5.23E+07 | 1.12E+07 | 9.27E+06 | 6.54E+06 | 7.37E+07 | 4.74E+06 | 1.17E+07 | 0.78 |
| Com_2562_neg | 5-Aminoimidazole-4-carboxamide-1-beta-d-ribofuranosyl 5-monophosphate | 2.11E+07 | 3.83E+07 | 1.62E+07 | 1.82E+07 | 6.15E+07 | 1.26E+07 | 5.42E+07 | 4.57E+07 | 5.28E+06 | 2.52E+07 | 0.63 |
| Com_25660_pos | S-(5-Adenosy)-L-Homocysteine | 7.73E+06 | 1.67E+06 | 1.29E+06 | 2.36E+06 | 2.02E+06 | 2.18E+06 | 5.11E+06 | 6.19E+06 | 2.64E+06 | 2.31E+06 | 0.41 |
| Com_2571_pos | 3-(5-phenyl-1,3-oxazol-2-yl)-4-(trifluoromethyl)pyridine | 5.66E+06 | 2.48E+07 | 1.00E+08 | 2.20E+08 | 7.41E+07 | 1.38E+07 | 6.61E+07 | 1.06E+08 | 1.08E+08 | 1.69E+08 | 0.61 |
| Com_25752_pos | Testosterone | 1.37E+06 | 1.13E+06 | 7.30E+06 | 1.53E+06 | 9.68E+05 | 9.25E+05 | 2.67E+06 | 1.20E+06 | 8.46E+05 | 1.35E+06 | 0.47 |
| Com_2576_pos | L-Tyrosinemethylester | 2.25E+08 | 1.97E+08 | 1.89E+07 | 5.86E+07 | 2.19E+07 | 2.16E+07 | 1.26E+07 | 9.86E+06 | 2.66E+07 | 2.44E+07 | 0.07 |
| Com_2584_pos | D-Glucosamine 6-phosphate | 1.01E+08 | 1.96E+08 | 8.11E+07 | 1.69E+08 | 4.22E+07 | 1.09E+08 | 9.59E+07 | 3.80E+07 | 9.37E+07 | 2.18E+08 | 0.86 |
| Com_2592_pos | Lysopa 18:0 | 3.54E+06 | 3.01E+06 | 4.94E+06 | 2.71E+06 | 2.91E+06 | 1.86E+06 | 3.73E+06 | 3.34E+06 | 2.99E+06 | 2.30E+08 | 0.46 |
| Com_25935_pos | 3,3-dimethyl-5-oxo-5-(2-piperidinoanilino)pentanoic acid | 4.18E+05 | 5.47E+05 | 7.18E+06 | 2.82E+06 | 4.29E+06 | 8.20E+05 | 1.46E+06 | 1.98E+06 | 7.42E+05 | 2.21E+06 | 0.61 |
| Com_2596_neg | 7-(4-methylpiperazino)pyrimido[4,5-d]pyrimidine-2,4(1H,3H)-dithione | 7.39E+06 | 1.83E+07 | 4.93E+05 | 6.99E+06 | 2.25E+06 | 4.47E+07 | 2.46E+07 | 2.18E+07 | 4.87E+06 | 2.55E+06 | 0.21 |
| Com_25993_pos | 4-methoxy-9-(3-methylbut-2-en-1-yl)-7H-furo[3,2-g]chromen-7-one | 2.60E+06 | 2.32E+06 | 1.89E+06 | 2.04E+06 | 3.02E+06 | 2.36E+06 | 5.72E+06 | 7.07E+06 | 2.39E+06 | 2.50E+06 | 0.16 |
| Com_2600_neg | 1-methyl-N-(3-methyl-5-cinnolinyl)-1H-imidazole-4-sulfonamide | 2.29E+05 | 9.33E+05 | 4.95E+05 | 1.51E+07 | 8.11E+05 | 4.46E+07 | 7.16E+06 | 2.76E+07 | 1.44E+07 | 9.64E+06 | 0.01 |
| Com_2600_pos | DL-2-(acetylamino)-3-phenylpropanoic acid | 2.34E+07 | 2.60E+07 | 2.78E+07 | 1.76E+08 | 3.63E+07 | 2.94E+07 | 1.91E+07 | 2.35E+08 | 2.81E+07 | 3.83E+07 | 0.93 |
| Com_26073_pos | Nobiletin | 1.48E+06 | 1.13E+06 | 1.48E+06 | 1.80E+06 | 5.64E+06 | 1.05E+06 | 4.36E+06 | 4.68E+06 | 4.47E+06 | 5.29E+06 | 0.18 |
| Com_26075_pos | Dopamine HCl | 9.00E+05 | 7.37E+05 | 6.38E+05 | 6.20E+05 | 7.79E+05 | 5.86E+06 | 3.25E+06 | 4.59E+06 | 7.20E+05 | 5.10E+06 | 0.02 |
| Com_2609_pos | 5,7-dihydroxy-3-(4-hydroxyphenyl)-4H-chromen-4-one | 1.30E+07 | 1.10E+07 | 4.78E+06 | 5.44E+07 | 4.10E+07 | 1.96E+08 | 6.65E+07 | 1.55E+08 | 4.34E+06 | 1.39E+07 | 0.34 |
| Com_2614_pos | TNH | 9.66E+07 | 1.10E+08 | 1.34E+08 | 5.13E+07 | 1.88E+08 | 5.09E+07 | 9.98E+07 | 1.48E+08 | 6.81E+07 | 1.45E+08 | 0.69 |
| Com_2615_pos | SQH | 3.16E+07 | 2.52E+07 | 2.08E+08 | 1.39E+08 | 4.37E+07 | 1.95E+08 | 1.38E+08 | 1.39E+08 | 1.45E+08 | 1.13E+08 | 0.12 |
| Com_26235_pos | L-Kynurenine | 4.57E+06 | 4.21E+06 | 3.73E+06 | 2.77E+06 | 5.55E+06 | 3.61E+06 | 4.20E+06 | 5.54E+06 | 2.85E+06 | 5.62E+06 | 0.83 |
| Com_26261_pos | 6-anilino-1,3-dimethyl-1,2,3,4-tetrahydropyrimidine-2,4-dione | 4.79E+06 | 5.20E+06 | 5.07E+06 | 3.47E+06 | 5.54E+06 | 3.18E+06 | 5.04E+06 | 3.53E+06 | 5.36E+06 | 4.68E+06 | 0.44 |
| Com_26273_pos | Cytisine | 7.34E+06 | 5.38E+06 | 3.49E+06 | 3.20E+06 | 3.62E+06 | 1.14E+06 | 1.68E+06 | 9.28E+05 | 3.37E+06 | 2.04E+06 | 0.01 |
| Com_26296_pos | N-Acetylglucosamine 1-phosphate | 8.18E+05 | 8.18E+05 | 6.97E+06 | 3.12E+06 | 1.75E+06 | 2.07E+06 | 1.35E+06 | 4.49E+06 | 1.15E+06 | 1.50E+06 | 0.95 |
| Com_2629_pos | L-Dopa | 1.32E+08 | 1.92E+08 | 1.36E+08 | 9.57E+07 | 1.50E+08 | 1.33E+08 | 1.21E+08 | 8.25E+07 | 1.83E+08 | 1.57E+08 | 0.78 |
| Com_2630_pos | ACar 18:1 | 1.91E+07 | 9.58E+07 | 4.70E+06 | 8.85E+07 | 1.07E+08 | 1.38E+08 | 2.71E+07 | 2.32E+08 | 2.40E+07 | 1.94E+07 | 0.70 |
| Com_2633_neg | (±)10(11)-EpDPA | 5.71E+07 | 3.65E+07 | 3.69E+07 | 3.76E+07 | 5.06E+07 | 2.14E+07 | 5.21E+07 | 5.12E+07 | 2.16E+07 | 3.50E+07 | 0.31 |
| Com_26360_pos | NPK | 1.25E+06 | 1.84E+06 | 4.15E+05 | 7.33E+05 | 1.32E+06 | 5.05E+06 | 8.78E+05 | 6.85E+06 | 7.02E+05 | 9.31E+05 | 0.31 |
| Com_2636_pos | 2,4-Dimethylbenzaldehyde | 1.74E+08 | 1.54E+08 | 2.30E+08 | 1.75E+08 | 1.07E+08 | 1.93E+08 | 1.77E+08 | 1.47E+08 | 1.16E+08 | 1.74E+08 | 0.88 |
| Com_26375_pos | 2-Furoic acid | 4.77E+06 | 3.29E+06 | 4.65E+06 | 4.89E+06 | 3.88E+06 | 3.18E+06 | 8.37E+06 | 5.14E+06 | 4.60E+06 | 4.80E+06 | 0.40 |
| Com_26404_pos | KKK | 1.18E+06 | 5.64E+06 | 3.59E+05 | 3.57E+05 | 5.26E+06 | 2.62E+06 | 3.56E+05 | 6.31E+06 | 2.82E+05 | 6.33E+05 | 0.74 |
| Com_2647_pos | 5-[(8Z,11Z)-pentadeca-8,11-dien-1-yl]benzene-1,3-diol | 2.14E+08 | 1.63E+08 | 3.90E+07 | 1.66E+08 | 1.85E+08 | 1.83E+08 | 1.89E+08 | 1.52E+08 | 1.58E+08 | 1.87E+08 | 0.45 |
| Com_264_pos | D-(+)-Maltose | 8.35E+08 | 1.66E+09 | 6.43E+08 | 3.16E+08 | 2.52E+09 | 2.18E+08 | 1.49E+09 | 2.28E+09 | 2.73E+08 | 3.43E+08 | 0.46 |
| Com_2651_neg | 1-Methylxanthine | 7.38E+05 | 1.36E+06 | 1.36E+06 | 2.12E+07 | 7.18E+06 | 4.32E+07 | 5.36E+07 | 2.58E+07 | 3.29E+07 | 1.84E+07 | 0.02 |
| Com_2653_pos | 6-[2-(2H-1,3-benzodioxol-5-yl)ethyl]-4-methoxy-2H-pyran-2-one | 5.38E+06 | 5.29E+06 | 1.01E+07 | 2.30E+06 | 1.23E+07 | 1.92E+08 | 6.62E+06 | 4.46E+07 | 1.13E+06 | 5.08E+06 | 0.47 |
| Com_2654_pos | Vitamin A | 4.24E+07 | 5.03E+07 | 5.70E+07 | 1.26E+08 | 7.89E+07 | 5.76E+07 | 9.09E+07 | 2.30E+08 | 6.25E+07 | 7.12E+07 | 0.38 |
| Com_26607_pos | N,N'-di[4-(2,6-dimethylmorpholino)phenyl]thiourea | 2.08E+06 | 3.20E+06 | 3.90E+05 | 1.51E+06 | 8.22E+05 | 5.59E+06 | 1.70E+06 | 8.61E+05 | 1.42E+06 | 1.77E+06 | 0.46 |
| Com_2661_pos | Allantoin | 1.61E+06 | 1.89E+08 | 1.51E+06 | 1.05E+06 | 2.59E+06 | 1.65E+06 | 1.36E+06 | 2.54E+06 | 1.16E+06 | 1.52E+06 | 0.38 |
| Com_2665_neg | tetranor-12(R)-HETE | 3.25E+07 | 4.08E+07 | 1.82E+07 | 2.95E+07 | 2.84E+07 | 1.15E+07 | 2.56E+07 | 1.88E+07 | 1.66E+07 | 2.73E+07 | 0.08 |
| Com_266_pos | Triethanolamine | 3.33E+09 | 4.13E+08 | 1.26E+08 | 4.82E+07 | 1.92E+07 | 2.45E+07 | 2.57E+07 | 2.22E+07 | 1.61E+08 | 1.95E+07 | 0.15 |
| Com_2672_pos | U-44069 | 6.07E+07 | 1.89E+08 | 1.01E+08 | 3.43E+07 | 5.30E+07 | 5.32E+07 | 8.55E+07 | 4.62E+07 | 1.70E+07 | 3.75E+07 | 0.20 |
| Com_267_neg | LPE 20:4 | 1.46E+08 | 7.03E+08 | 6.32E+06 | 2.49E+07 | 5.39E+08 | 2.81E+08 | 1.50E+08 | 7.49E+08 | 1.69E+06 | 4.12E+07 | 0.85 |
| Com_2681_pos | Clinafloxacin | 1.25E+06 | 1.38E+06 | 1.30E+06 | 1.22E+08 | 1.20E+06 | 1.90E+08 | 9.41E+07 | 1.24E+08 | 8.95E+07 | 1.07E+08 | 0.02 |
| Com_2687_neg | cis-4-Hydroxy-D-proline | 1.00E+07 | 1.73E+07 | 3.77E+07 | 2.04E+07 | 4.27E+07 | 1.46E+07 | 5.12E+07 | 6.92E+07 | 1.38E+07 | 1.71E+07 | 0.73 |
| Com_26_neg | Lithocholic Acid | 7.04E+09 | 7.94E+09 | 3.61E+09 | 3.90E+09 | 4.64E+09 | 2.81E+09 | 3.49E+09 | 2.44E+08 | 8.24E+09 | 8.29E+09 | 0.40 |
| Com_26_pos | Testosterone undecanoate | 2.24E+09 | 2.27E+09 | 9.61E+08 | 2.74E+10 | 9.58E+09 | 3.72E+09 | 8.45E+09 | 5.23E+09 | 5.69E+09 | 2.72E+09 | 0.84 |
| Com_2703_neg | L-Asparagine | 8.93E+06 | 7.29E+06 | 1.10E+07 | 5.28E+07 | 5.75E+07 | 3.91E+07 | 7.82E+07 | 6.46E+07 | 9.53E+06 | 4.58E+07 | 0.24 |
| Com_2708_neg | Prostaglandin D2 | 2.30E+07 | 1.83E+07 | 7.55E+07 | 1.91E+07 | 3.90E+07 | 9.28E+06 | 1.06E+07 | 7.43E+06 | 2.93E+07 | 2.00E+07 | 0.06 |
| Com_2709_neg | N-α-Acetyl-L-asparagine | 5.52E+06 | 8.20E+06 | 1.36E+07 | 4.71E+07 | 1.27E+07 | 4.21E+07 | 4.20E+07 | 3.49E+07 | 1.37E+07 | 4.66E+07 | 0.07 |
| Com_2712_pos | (+/-)11(12)-EET | 1.08E+08 | 8.42E+07 | 2.98E+07 | 2.06E+08 | 8.39E+07 | 9.91E+07 | 1.06E+08 | 9.21E+07 | 9.45E+07 | 1.29E+08 | 0.59 |
| Com_2713_pos | Asp-glu | 1.52E+07 | 3.05E+07 | 6.91E+07 | 1.20E+08 | 4.31E+07 | 1.84E+08 | 1.15E+08 | 2.24E+08 | 3.91E+07 | 3.11E+07 | 0.22 |
| Com_2718_pos | Biotin | 5.14E+07 | 1.08E+08 | 1.37E+08 | 5.95E+07 | 1.80E+08 | 5.43E+07 | 5.62E+07 | 7.49E+07 | 4.59E+07 | 6.59E+07 | 0.11 |
| Com_2727_pos | N6-Acetyl-L-lysine | 7.39E+07 | 9.91E+07 | 1.93E+08 | 1.64E+08 | 1.42E+08 | 1.40E+08 | 1.27E+08 | 1.49E+08 | 1.38E+08 | 2.17E+08 | 0.40 |
| Com_27314_pos | 13,14-dihydro-15-keto-PGD2 | 3.91E+06 | 5.21E+06 | 1.03E+06 | 4.99E+06 | 1.13E+06 | 8.48E+05 | 1.04E+06 | 6.43E+05 | 1.73E+06 | 2.82E+06 | 0.14 |
| Com_2734_neg | Glycoursodeoxycholic acid | 1.50E+07 | 2.01E+07 | 1.55E+07 | 1.02E+07 | 4.54E+07 | 4.17E+07 | 4.95E+06 | 2.40E+06 | 1.46E+07 | 2.62E+07 | 0.44 |
| Com_2743_pos | N-Acetyl-L-glutamic acid | 1.05E+07 | 2.22E+07 | 3.03E+07 | 8.97E+06 | 1.69E+07 | 1.85E+08 | 2.67E+07 | 1.46E+07 | 1.76E+07 | 1.14E+07 | 0.38 |
| Com_2746_pos | 5-Hydroxyindole | 1.77E+08 | 1.67E+08 | 1.38E+08 | 8.34E+07 | 1.62E+08 | 1.41E+08 | 1.61E+08 | 8.74E+07 | 1.91E+08 | 1.35E+08 | 0.94 |
| Com_27506_pos | Cyclohexylsulfamate | 2.96E+06 | 5.12E+06 | 1.69E+06 | 1.61E+06 | 3.10E+06 | 9.96E+05 | 2.36E+06 | 4.66E+06 | 1.79E+06 | 2.33E+06 | 0.55 |
| Com_2752_pos | Hydrocortisone | 2.05E+06 | 9.57E+07 | 4.91E+07 | 1.19E+08 | 1.17E+08 | 1.84E+08 | 2.06E+08 | 1.24E+08 | 1.12E+08 | 1.10E+06 | 0.82 |
| Com_27550_pos | Adenosine 5'-Diphosphate | 1.19E+06 | 5.10E+06 | 1.25E+06 | 8.35E+05 | 8.93E+05 | 9.07E+05 | 9.63E+05 | 1.45E+06 | 7.04E+05 | 2.72E+06 | 0.69 |
| Com_2755_neg | Glu-Gln | 6.30E+06 | 8.36E+06 | 1.36E+07 | 2.71E+07 | 1.37E+07 | 4.13E+07 | 1.61E+07 | 5.43E+07 | 7.38E+06 | 1.28E+07 | 0.29 |
| Com_27589_pos | 1,7-bis(4-hydroxyphenyl)-5-methoxyheptan-3-one | 6.56E+06 | 4.17E+06 | 4.13E+06 | 3.79E+06 | 2.87E+06 | 5.11E+06 | 5.51E+06 | 3.52E+06 | 4.33E+06 | 4.59E+06 | 0.56 |
| Com_2768_pos | N-Stearoyl taurine | 2.14E+08 | 1.55E+08 | 6.29E+07 | 2.01E+08 | 1.48E+08 | 3.31E+07 | 1.52E+08 | 2.65E+07 | 9.56E+07 | 1.40E+08 | 0.14 |
| Com_27696_pos | (6E)-7-(2H-1,3-benzodioxol-5-yl)-1-(piperidin-1-yl)hept-6-en-1-one | 2.55E+06 | 3.22E+06 | 1.22E+06 | 3.43E+06 | 3.56E+06 | 5.09E+06 | 3.00E+06 | 2.45E+06 | 1.36E+06 | 3.73E+06 | 0.77 |
| Com_27718_pos | 17beta-Trenbolone | 3.63E+06 | 5.03E+06 | 4.71E+06 | 1.94E+06 | 4.40E+06 | 8.86E+05 | 3.52E+06 | 1.47E+06 | 3.31E+06 | 3.34E+06 | 0.15 |
| Com_2773_pos | PC (18:2e/2:0) | 3.93E+06 | 1.81E+08 | 7.83E+06 | 4.00E+06 | 6.36E+07 | 4.16E+07 | 1.40E+07 | 6.68E+07 | 4.14E+06 | 4.17E+06 | 0.88 |
| Com_2779_neg | Choline bitartrate | 4.06E+06 | 7.63E+06 | 1.13E+07 | 5.90E+07 | 5.37E+06 | 1.39E+07 | 1.57E+07 | 1.87E+07 | 9.36E+06 | 2.06E+07 | 0.46 |
| Com_2781_pos | Tropine | 1.80E+07 | 3.47E+07 | 2.21E+08 | 9.92E+07 | 1.62E+07 | 1.25E+07 | 2.68E+07 | 1.06E+07 | 8.52E+07 | 4.19E+07 | 0.40 |
| Com_2782_pos | Trigonelline | 4.49E+07 | 6.62E+07 | 1.03E+08 | 1.40E+08 | 1.36E+08 | 1.24E+08 | 2.41E+08 | 1.53E+08 | 1.59E+08 | 2.13E+08 | 0.04 |
| Com_2791_pos | Progesterone | 2.19E+08 | 1.39E+08 | 9.75E+07 | 9.74E+07 | 1.24E+08 | 1.82E+08 | 8.24E+07 | 2.96E+07 | 1.61E+08 | 1.29E+08 | 0.48 |
| Com_279_pos | Threonine | 8.39E+08 | 1.00E+09 | 1.23E+09 | 1.78E+09 | 1.14E+09 | 1.68E+09 | 1.77E+09 | 1.64E+09 | 1.37E+09 | 2.86E+09 | 0.04 |
| Com_28025_pos | 2-deoxyglucose-6-phosphate | 3.75E+06 | 4.88E+06 | 2.35E+06 | 7.90E+05 | 3.79E+06 | 1.37E+06 | 2.93E+06 | 1.22E+06 | 3.28E+06 | 6.92E+05 | 0.29 |
| Com_2808_neg | 2-Ketoadipic acid | 1.38E+07 | 1.63E+07 | 2.45E+07 | 1.53E+07 | 5.48E+07 | 1.74E+07 | 3.65E+07 | 3.16E+07 | 2.54E+07 | 5.97E+07 | 0.28 |
| Com_2809_pos | (-)-Caryophyllene oxide | 5.01E+07 | 3.95E+07 | 6.37E+07 | 1.26E+08 | 6.56E+07 | 1.80E+08 | 6.71E+07 | 8.02E+07 | 4.59E+07 | 1.06E+08 | 0.34 |
| Com_2812_pos | N-(1-benzothiophen-2-yl)-N'-(2-methylphenyl)urea | 2.19E+06 | 7.12E+06 | 3.27E+06 | 2.32E+06 | 1.13E+07 | 7.45E+07 | 5.41E+07 | 2.15E+08 | 3.35E+06 | 8.22E+06 | 0.06 |
| Com_2814_pos | Pyridoxine | 2.29E+08 | 7.55E+07 | 3.13E+07 | 3.09E+07 | 1.29E+08 | 4.72E+07 | 1.04E+08 | 1.08E+08 | 7.12E+07 | 9.93E+07 | 0.80 |
| Com_28160_pos | N-{5-[(dimethylamino)sulfonyl]-2-methylphenyl}cyclohexanecarboxamide | 1.74E+06 | 7.17E+05 | 4.15E+05 | 4.72E+05 | 6.35E+05 | 9.05E+05 | 4.27E+05 | 4.10E+05 | 5.04E+06 | 5.10E+05 | 0.73 |
| Com_28198_pos | Epoxomicin | 3.27E+06 | 2.75E+06 | 2.58E+06 | 2.19E+06 | 2.48E+06 | 2.84E+06 | 1.89E+06 | 7.08E+05 | 5.02E+06 | 3.30E+06 | 0.70 |
| Com_2819_neg | LPG 20:2 | 4.36E+06 | 3.80E+07 | 5.63E+06 | 4.06E+06 | 4.50E+06 | 1.21E+06 | 4.96E+06 | 3.29E+06 | 2.29E+06 | 2.60E+06 | 0.08 |
| Com_28205_pos | 1-Oleoyl-Sn-Glycero-3-Phosphocholine | 2.62E+06 | 1.29E+06 | 3.04E+06 | 5.35E+06 | 3.48E+06 | 4.43E+06 | 1.83E+06 | 1.60E+06 | 1.59E+06 | 3.86E+06 | 0.61 |
| Com_28292_pos | 5,7-dihydroxy-3-(4-methoxyphenyl)-4H-chromen-4-one | 4.03E+06 | 3.61E+06 | 4.46E+06 | 3.70E+06 | 2.41E+06 | 2.42E+06 | 4.26E+06 | 5.56E+06 | 4.47E+06 | 1.87E+06 | 0.87 |
| Com_282_pos | L-Aspartic acid | 8.53E+08 | 8.36E+08 | 1.89E+09 | 2.68E+09 | 9.74E+08 | 7.78E+08 | 2.09E+09 | 8.42E+08 | 2.14E+09 | 1.62E+09 | 0.86 |
| Com_28445_pos | 5-[(dimethylamino)methylidene]-3-phenyl-1,3-thiazolane-2,4-dione | 3.21E+06 | 4.61E+06 | 2.53E+06 | 1.58E+06 | 1.91E+06 | 2.08E+06 | 1.75E+06 | 1.29E+06 | 4.91E+06 | 2.16E+06 | 0.59 |
| Com_28481_pos | (+/-)5(6)-DiHET | 5.77E+06 | 1.82E+06 | 3.87E+06 | 2.15E+06 | 2.05E+06 | 1.62E+06 | 2.55E+06 | 3.15E+06 | 1.20E+06 | 5.54E+06 | 0.68 |
| Com_284_pos | N-Tetradecanamide | 1.36E+09 | 2.40E+09 | 2.73E+09 | 9.69E+08 | 1.24E+09 | 7.42E+08 | 2.26E+09 | 1.86E+09 | 1.01E+09 | 1.65E+09 | 0.63 |
| Com_28524_pos | L-arginine | 2.01E+06 | 3.60E+06 | 1.25E+06 | 3.03E+06 | 1.93E+06 | 3.67E+06 | 2.13E+06 | 1.79E+06 | 4.88E+06 | 5.46E+06 | 0.21 |
| Com_2856_pos | 12-Oxo phytodienoic acid | 7.63E+07 | 1.31E+08 | 1.56E+08 | 7.03E+07 | 5.08E+07 | 8.90E+07 | 9.72E+07 | 1.91E+07 | 1.82E+08 | 1.42E+08 | 0.91 |
| Com_28587_pos | Asp-Phe | 2.63E+06 | 3.73E+06 | 3.26E+06 | 2.81E+06 | 3.57E+06 | 2.81E+06 | 3.14E+06 | 2.55E+06 | 4.85E+06 | 1.83E+06 | 0.60 |
| Com_2860_pos | 6-Hydroxynicotinic acid | 9.04E+07 | 6.33E+06 | 2.14E+08 | 4.12E+07 | 3.26E+07 | 1.57E+07 | 4.60E+07 | 1.35E+07 | 4.99E+07 | 1.76E+08 | 0.87 |
| Com_2861_neg | Ascorbyl palmitate | 3.64E+07 | 9.34E+06 | 2.56E+07 | 2.96E+07 | 3.61E+07 | 9.80E+06 | 4.61E+07 | 5.95E+07 | 3.66E+07 | 2.99E+07 | 0.58 |
| Com_2862_pos | LSD-d3 | 1.20E+08 | 8.78E+07 | 1.36E+08 | 1.94E+08 | 1.15E+08 | 2.40E+07 | 1.20E+08 | 6.96E+06 | 1.08E+08 | 2.03E+08 | 0.24 |
| Com_2863_pos | Isopropyl myristate | 3.56E+07 | 1.32E+08 | 1.09E+08 | 2.51E+07 | 1.70E+08 | 1.67E+08 | 2.40E+07 | 5.57E+07 | 2.41E+07 | 3.42E+07 | 0.37 |
| Com_28676_pos | 6-methoxy-4-(trifluoromethyl)nicotinamide | 4.23E+05 | 4.82E+05 | 5.65E+06 | 4.32E+05 | 1.66E+06 | 3.72E+05 | 5.29E+05 | 5.79E+05 | 3.15E+05 | 4.93E+05 | 0.21 |
| Com_28746_pos | Dl-3-Hydroxy-kynurenine | 3.32E+06 | 3.53E+06 | 4.20E+06 | 2.94E+06 | 4.46E+06 | 2.95E+06 | 2.76E+06 | 2.30E+06 | 2.90E+06 | 3.55E+06 | 0.05 |
| Com_28755_pos | 5-Hydroxytryptophan | 2.01E+06 | 3.70E+06 | 1.81E+06 | 2.70E+06 | 4.18E+05 | 3.40E+05 | 1.83E+06 | 5.85E+05 | 4.77E+06 | 1.41E+06 | 0.56 |
| Com_2878_pos | PC (14:0e/4:0) | 1.58E+07 | 8.58E+07 | 5.37E+07 | 1.36E+07 | 9.43E+07 | 2.70E+07 | 2.51E+07 | 2.09E+08 | 1.16E+07 | 2.20E+07 | 0.77 |
| Com_28868_pos | 5-[(1-benzothiophen-3-ylmethyl)sulfanyl]-1-methyl-4-nitro-1H-imidazole | 5.52E+05 | 4.94E+05 | 7.75E+05 | 5.29E+05 | 5.13E+05 | 4.52E+05 | 8.40E+05 | 5.54E+05 | 4.72E+06 | 6.43E+05 | 0.32 |
| Com_288_neg | D-(+)-Malic acid | 3.79E+08 | 2.89E+08 | 1.21E+09 | 8.14E+08 | 4.82E+08 | 3.86E+08 | 1.04E+09 | 6.74E+08 | 9.42E+07 | 5.07E+08 | 0.58 |
| Com_2891_pos | DL-o-Tyrosine | 1.29E+08 | 9.46E+07 | 1.18E+08 | 5.97E+07 | 1.67E+08 | 1.17E+08 | 1.50E+08 | 1.17E+08 | 1.68E+08 | 1.30E+08 | 0.28 |
| Com_2894_pos | Tetrahydrocortisone | 2.63E+07 | 9.01E+07 | 6.54E+07 | 1.91E+08 | 8.13E+07 | 1.27E+08 | 1.58E+08 | 9.73E+07 | 1.49E+08 | 9.54E+07 | 0.21 |
| Com_2899_pos | (2R)-2-[(2R,5S)-5-[(2S)-2-hydroxybutyl]oxolan-2-yl]propanoic acid | 1.39E+08 | 9.94E+07 | 8.62E+07 | 8.42E+07 | 5.65E+07 | 3.67E+07 | 1.27E+08 | 3.18E+07 | 1.79E+08 | 1.01E+08 | 0.70 |
| Com_29212_pos | Lysopa 16:0 | 5.66E+06 | 3.85E+06 | 2.01E+06 | 2.22E+06 | 1.98E+06 | 2.18E+06 | 6.28E+06 | 6.04E+05 | 3.51E+06 | 3.73E+06 | 0.81 |
| Com_2921_pos | All trans retinal | 2.89E+07 | 2.33E+07 | 2.74E+07 | 1.89E+08 | 4.61E+07 | 3.36E+07 | 6.27E+07 | 4.89E+07 | 2.49E+07 | 1.67E+07 | 0.58 |
| Com_29258_pos | beta-Estradiol 17-Acetate | 4.84E+06 | 4.37E+06 | 3.04E+06 | 2.76E+06 | 2.16E+06 | 1.44E+06 | 2.04E+06 | 6.48E+05 | 2.23E+06 | 2.48E+06 | 0.04 |
| Com_29297_pos | Hesperetin | 3.80E+06 | 3.71E+06 | 5.11E+06 | 3.10E+06 | 3.28E+06 | 3.16E+06 | 3.90E+06 | 2.23E+06 | 3.33E+06 | 2.51E+06 | 0.12 |
| Com_292_neg | 4-Hydroxybenzaldehyde | 1.26E+08 | 2.07E+08 | 2.02E+08 | 1.49E+08 | 1.87E+08 | 3.11E+08 | 4.83E+08 | 1.09E+09 | 1.34E+08 | 5.61E+08 | 0.06 |
| Com_2931_pos | Glycine anhydride | 2.58E+07 | 6.02E+07 | 1.83E+08 | 3.87E+07 | 5.18E+07 | 1.72E+08 | 2.40E+07 | 2.19E+07 | 2.50E+07 | 6.74E+07 | 0.62 |
| Com_2938_neg | 2-Hydroxy-4-methylpentanoic acid | 3.84E+07 | 2.97E+07 | 4.25E+07 | 3.08E+07 | 2.36E+07 | 2.06E+07 | 4.40E+07 | 2.00E+07 | 3.19E+07 | 6.17E+07 | 0.99 |
| Com_29400_pos | Pyridoxine O-Glucoside | 4.37E+06 | 4.32E+06 | 6.98E+05 | 2.04E+06 | 2.39E+06 | 1.71E+06 | 2.61E+06 | 6.54E+05 | 2.42E+06 | 2.46E+06 | 0.55 |
| Com_2944_pos | N6-Me-Adenosine | 2.89E+07 | 2.44E+07 | 4.51E+07 | 1.88E+08 | 1.19E+07 | 9.50E+06 | 2.61E+07 | 1.16E+07 | 1.86E+07 | 1.51E+08 | 0.54 |
| Com_29492_pos | KMH | 1.01E+06 | 1.39E+06 | 3.70E+05 | 1.99E+06 | 1.31E+06 | 1.83E+06 | 3.91E+06 | 3.44E+06 | 2.92E+06 | 1.60E+06 | 0.03 |
| Com_2949_pos | (S)-2-Hydroxybutanoicacid | 6.17E+07 | 2.81E+07 | 2.07E+08 | 1.73E+08 | 6.48E+07 | 7.04E+07 | 2.08E+08 | 7.37E+07 | 9.06E+07 | 1.01E+08 | 0.68 |
| Com_29514_pos | Quinolinic acid | 7.63E+05 | 8.51E+05 | 5.00E+06 | 1.40E+06 | 1.05E+06 | 1.14E+06 | 5.06E+06 | 5.18E+06 | 2.44E+06 | 1.21E+06 | 0.25 |
| Com_2952_pos | N-Propionylglycine | 2.19E+07 | 2.36E+07 | 5.06E+07 | 4.39E+07 | 6.82E+07 | 6.02E+07 | 6.34E+07 | 2.03E+08 | 3.21E+07 | 5.86E+07 | 0.16 |
| Com_29564_pos | FMK | 4.52E+05 | 4.32E+05 | 3.14E+05 | 3.76E+05 | 4.64E+05 | 2.66E+05 | 3.76E+05 | 3.61E+05 | 4.44E+06 | 4.11E+05 | 0.52 |
| Com_2956_neg | N-Acetyl-D-galactosamine 4-sulfate | 6.75E+06 | 9.72E+06 | 2.55E+06 | 1.22E+07 | 3.03E+07 | 3.78E+07 | 8.60E+06 | 3.79E+07 | 9.96E+05 | 1.06E+07 | 0.85 |
| Com_2958_pos | Ergosta-5,7,9(11),22-Tetraen-3-beta-Ol | 6.58E+07 | 3.13E+07 | 2.06E+08 | 1.67E+08 | 1.20E+08 | 1.41E+07 | 8.87E+07 | 2.69E+07 | 1.05E+08 | 7.20E+07 | 0.21 |
| Com_2963_pos | Epinephrine bitartrate | 5.19E+06 | 3.76E+06 | 7.49E+06 | 9.24E+06 | 5.30E+06 | 1.69E+08 | 8.17E+06 | 5.93E+06 | 9.15E+06 | 2.92E+07 | 0.14 |
| Com_29673_pos | 4-Pregnen-17alpha,20alpha-Diol-3-One | 3.21E+06 | 3.55E+06 | 3.78E+06 | 4.69E+06 | 1.07E+06 | 2.58E+06 | 2.27E+06 | 9.85E+05 | 4.12E+06 | 2.42E+06 | 0.47 |
| Com_2977_neg | 2-Hydroxy-1,4-naphthoquinone | 3.37E+06 | 7.52E+06 | 1.42E+07 | 5.27E+07 | 8.02E+06 | 3.73E+07 | 2.90E+07 | 2.63E+07 | 1.06E+07 | 4.00E+07 | 0.14 |
| Com_297_pos | Pantothenic acid | 6.27E+08 | 1.62E+09 | 1.39E+09 | 1.37E+09 | 1.51E+09 | 1.14E+09 | 3.29E+09 | 1.94E+09 | 1.88E+09 | 2.26E+09 | 0.09 |
| Com_29840_pos | N'2-(2-hydroxybenzylidene)-5-nitrofuran-2-carbohydrazide | 3.26E+06 | 1.94E+06 | 5.10E+06 | 1.72E+06 | 3.15E+06 | 2.46E+06 | 3.90E+06 | 2.30E+06 | 4.00E+06 | 1.93E+06 | 0.98 |
| Com_298_pos | D-(-)-Glutamine | 6.81E+08 | 7.96E+08 | 7.89E+08 | 1.62E+09 | 1.57E+09 | 1.74E+09 | 1.56E+09 | 2.68E+09 | 9.76E+08 | 8.82E+08 | 0.24 |
| Com_2_neg | Elaidic acid | 2.88E+10 | 4.64E+10 | 7.02E+10 | 1.20E+11 | 5.29E+10 | 7.44E+09 | 6.15E+10 | 4.07E+10 | 6.48E+10 | 5.31E+10 | 0.38 |
| Com_30152_pos | NG,NG-Dimethyl-L-arginine | 4.16E+06 | 3.61E+06 | 4.96E+06 | 3.12E+06 | 3.66E+06 | 2.72E+06 | 4.41E+06 | 4.37E+06 | 3.37E+06 | 4.77E+06 | 0.99 |
| Com_3019_neg | Aldosterone | 8.47E+06 | 2.12E+07 | 1.07E+07 | 4.23E+07 | 1.39E+07 | 3.43E+07 | 8.04E+07 | 9.96E+06 | 3.12E+07 | 2.46E+07 | 0.22 |
| Com_30421_pos | 4-Hydroxyretinoic Acid | 3.29E+06 | 3.57E+06 | 3.77E+06 | 4.13E+06 | 3.24E+06 | 3.23E+06 | 3.29E+06 | 2.06E+06 | 4.12E+06 | 2.57E+06 | 0.20 |
| Com_30481_pos | Methylmalonate | 1.43E+06 | 1.61E+06 | 1.27E+06 | 1.78E+06 | 1.61E+06 | 1.33E+06 | 3.45E+06 | 1.25E+06 | 1.46E+06 | 4.64E+06 | 0.33 |
| Com_30494_pos | 2-[5-(2-hydroxypropyl)oxolan-2-yl]propanoic acid | 2.53E+06 | 3.93E+06 | 2.39E+06 | 1.37E+06 | 1.90E+06 | 1.80E+06 | 2.66E+06 | 1.54E+06 | 2.49E+06 | 1.77E+06 | 0.55 |
| Com_3049_neg | L-Alanyl-L-proline | 2.94E+07 | 3.41E+07 | 2.50E+07 | 1.93E+07 | 2.99E+07 | 2.69E+07 | 1.99E+07 | 2.41E+07 | 8.75E+06 | 1.61E+07 | 0.11 |
| Com_3055_pos | Physostigmine | 1.66E+06 | 3.10E+06 | 9.01E+05 | 1.78E+08 | 2.87E+07 | 2.49E+06 | 1.16E+07 | 6.85E+06 | 3.97E+05 | 1.85E+06 | 0.40 |
| Com_3060_pos | Methionine sulfoxide | 5.51E+07 | 1.13E+08 | 6.77E+07 | 8.94E+07 | 9.54E+07 | 1.18E+08 | 1.39E+08 | 1.51E+08 | 5.93E+07 | 1.67E+08 | 0.13 |
| Com_3062_pos | SLK | 3.79E+07 | 7.21E+07 | 1.06E+08 | 1.38E+08 | 1.55E+08 | 1.12E+08 | 9.90E+07 | 1.52E+08 | 3.59E+07 | 1.52E+08 | 0.84 |
| Com_30669_pos | Estradiol Benzoate | 1.06E+06 | 9.89E+05 | 9.79E+05 | 2.28E+06 | 1.22E+06 | 3.90E+06 | 8.44E+05 | 3.67E+06 | 8.31E+05 | 3.03E+06 | 0.27 |
| Com_3067_pos | o-Toluic Acid | 1.91E+07 | 2.64E+07 | 1.95E+08 | 1.36E+07 | 4.46E+07 | 2.12E+07 | 1.94E+07 | 1.81E+07 | 1.76E+07 | 2.14E+07 | 0.26 |
| Com_306_pos | Acetylcholine | 4.88E+08 | 1.96E+08 | 1.17E+09 | 1.90E+09 | 2.58E+08 | 6.26E+08 | 1.68E+09 | 9.64E+08 | 1.81E+09 | 2.57E+09 | 0.12 |
| Com_3070_neg | Malonic acid | 5.76E+06 | 2.13E+07 | 1.25E+07 | 5.50E+06 | 1.35E+07 | 3.56E+07 | 3.69E+07 | 5.73E+07 | 3.92E+06 | 7.82E+06 | 0.34 |
| Com_30782_pos | Uridine 5'-Diphospho-N-Acetylgalactosamine | 5.43E+05 | 4.19E+05 | 6.36E+05 | 4.25E+06 | 4.96E+05 | 4.14E+05 | 6.04E+05 | 5.89E+05 | 5.40E+05 | 5.87E+05 | 0.43 |
| Com_30823_pos | Estradiol | 2.59E+06 | 3.02E+06 | 3.01E+06 | 1.11E+06 | 3.70E+06 | 1.72E+06 | 2.02E+06 | 1.13E+06 | 1.97E+06 | 2.79E+06 | 0.28 |
| Com_30871_pos | VMK | 1.47E+06 | 2.72E+06 | 3.96E+05 | 4.21E+06 | 2.39E+06 | 2.76E+06 | 2.42E+06 | 2.34E+06 | 2.10E+06 | 3.26E+06 | 0.41 |
| Com_308_neg | 5-[(Benzoyloxy)methyl]-4,5,6-trihydroxy-2-cyclohexen-1-yl benzoate | 3.26E+07 | 8.00E+07 | 8.06E+07 | 1.17E+07 | 8.77E+08 | 1.99E+07 | 1.13E+08 | 2.15E+08 | 8.99E+06 | 1.68E+07 | 0.49 |
| Com_3093_neg | L-Threonic acid | 8.54E+06 | 1.86E+07 | 3.76E+07 | 3.62E+07 | 1.88E+07 | 1.15E+07 | 6.28E+07 | 5.73E+07 | 2.70E+07 | 4.05E+07 | 0.27 |
| Com_30994_pos | Folic acid | 6.20E+05 | 6.41E+05 | 7.56E+05 | 9.77E+05 | 1.81E+06 | 1.21E+06 | 4.22E+06 | 4.53E+06 | 5.43E+05 | 8.02E+05 | 0.27 |
| Com_309_neg | FAHFA (18:2/18:1) | 3.25E+08 | 3.65E+08 | 1.13E+08 | 4.38E+08 | 4.83E+08 | 1.32E+07 | 4.22E+08 | 3.27E+08 | 5.98E+08 | 1.48E+08 | 0.47 |
| Com_30_pos | L-Phenylalanine | 4.59E+09 | 1.01E+10 | 6.15E+09 | 2.53E+10 | 9.30E+09 | 1.27E+10 | 1.06E+10 | 2.05E+10 | 6.94E+09 | 2.04E+10 | 0.35 |
| Com_3107_neg | Protectin D1 | 4.50E+07 | 3.33E+07 | 5.85E+06 | 1.18E+07 | 3.90E+07 | 1.45E+07 | 1.01E+07 | 6.74E+06 | 7.73E+06 | 2.02E+07 | 0.19 |
| Com_3118_neg | 19(R)-Hydroxy prostaglandin A2 | 2.65E+07 | 2.56E+07 | 3.99E+07 | 3.88E+07 | 1.94E+07 | 2.01E+07 | 3.55E+07 | 1.19E+07 | 3.24E+07 | 3.45E+07 | 0.56 |
| Com_31291_pos | Taurodeoxycholic acid sodium salt | 3.00E+06 | 3.64E+06 | 9.57E+05 | 2.38E+06 | 1.55E+06 | 1.60E+06 | 1.54E+06 | 4.58E+05 | 1.69E+06 | 1.68E+06 | 0.19 |
| Com_3130_pos | Thr-Leu | 3.60E+07 | 7.01E+07 | 1.99E+06 | 6.97E+07 | 4.82E+07 | 1.57E+08 | 3.89E+07 | 6.93E+07 | 1.25E+07 | 8.05E+07 | 0.44 |
| Com_3131_neg | Cannabigerolic acid | 4.45E+07 | 3.30E+07 | 3.95E+06 | 1.02E+07 | 3.86E+07 | 1.44E+07 | 9.26E+06 | 5.38E+06 | 7.17E+06 | 1.80E+07 | 0.26 |
| Com_3133_pos | 4-(3,4-dihydro-2H-1,5-benzodioxepin-7-ylamino)-4-oxobutanoic acid | 3.90E+06 | 1.43E+07 | 7.29E+06 | 1.45E+07 | 7.07E+06 | 2.51E+07 | 8.23E+06 | 1.88E+08 | 2.58E+06 | 2.67E+07 | 0.32 |
| Com_3141_pos | 4-(benzoylamino)-3-hydroxybutanoic acid | 6.26E+07 | 6.51E+07 | 7.30E+07 | 1.70E+08 | 4.72E+07 | 2.76E+07 | 1.65E+08 | 7.33E+07 | 1.05E+08 | 7.51E+07 | 0.96 |
| Com_3150_pos | Lysopc 20:4 | 5.05E+06 | 1.54E+08 | 2.38E+06 | 3.23E+06 | 4.02E+07 | 8.57E+06 | 6.01E+06 | 1.51E+06 | 8.59E+05 | 6.63E+05 | 0.12 |
| Com_31564_pos | 1-(7-methoxy-2-oxo-2H-chromen-8-yl)-3-methyl-2-oxobutyl acetate | 8.05E+05 | 4.96E+05 | 5.90E+05 | 3.95E+06 | 1.13E+06 | 1.46E+06 | 2.33E+06 | 1.42E+06 | 1.88E+06 | 2.11E+06 | 0.19 |
| Com_3161_neg | LPS 18:2 | 2.95E+06 | 3.23E+07 | 1.41E+06 | 1.74E+06 | 1.72E+07 | 3.13E+06 | 1.73E+06 | 5.60E+07 | 1.15E+06 | 1.54E+06 | 0.68 |
| Com_3168_pos | Citraconic acid | 7.30E+06 | 1.10E+07 | 4.00E+07 | 1.35E+08 | 1.90E+07 | 1.55E+08 | 6.57E+07 | 9.68E+07 | 3.97E+07 | 1.47E+08 | 0.06 |
| Com_31898_pos | 6-(3-hydroxybutan-2-yl)-5-(hydroxymethyl)-4-methoxy-2H-pyran-2-one | 1.26E+06 | 1.36E+06 | 7.52E+05 | 8.41E+05 | 7.49E+05 | 2.26E+06 | 1.14E+06 | 2.09E+06 | 3.59E+06 | 2.66E+06 | 0.01 |
| Com_31944_pos | N,5-Bis(3-nitrophenyl)oxazol-2-amine | 7.68E+05 | 7.29E+05 | 4.21E+06 | 7.71E+05 | 5.68E+05 | 4.78E+05 | 6.43E+05 | 5.87E+05 | 7.02E+05 | 8.02E+05 | 0.27 |
| Com_319_neg | Valeric acid | 5.78E+08 | 5.90E+08 | 2.90E+08 | 2.86E+08 | 5.21E+08 | 9.65E+07 | 4.43E+08 | 2.70E+08 | 2.89E+08 | 3.63E+08 | 0.15 |
| Com_3201_pos | Carbocysteine | 2.75E+06 | 2.46E+06 | 4.15E+06 | 6.08E+07 | 4.25E+06 | 1.53E+08 | 3.07E+07 | 1.25E+08 | 7.09E+06 | 1.59E+07 | 0.06 |
| Com_32032_pos | DPK | 1.01E+06 | 1.83E+06 | 2.92E+05 | 4.21E+05 | 4.47E+05 | 3.44E+06 | 3.02E+05 | 3.89E+05 | 2.52E+05 | 3.32E+05 | 0.72 |
| Com_32140_pos | Glutathione | 6.04E+05 | 6.32E+05 | 4.82E+05 | 5.72E+05 | 2.15E+06 | 2.67E+06 | 5.01E+05 | 4.08E+06 | 6.20E+05 | 6.45E+05 | 0.40 |
| Com_32376_pos | Chlortetracycline | 1.56E+05 | 1.67E+05 | 1.70E+05 | 1.51E+06 | 1.81E+05 | 2.96E+06 | 4.60E+06 | 1.97E+06 | 3.44E+06 | 1.37E+06 | 0.00 |
| Com_32381_pos | 17α-Hydroxyprogesterone | 2.72E+06 | 1.94E+06 | 3.06E+06 | 3.67E+06 | 1.87E+06 | 3.22E+06 | 2.64E+06 | 1.88E+06 | 2.81E+06 | 3.06E+06 | 0.79 |
| Com_3240_neg | (±)19(20)-DiHDPA | 3.87E+07 | 2.87E+07 | 9.00E+06 | 1.68E+07 | 3.25E+07 | 3.31E+07 | 2.51E+07 | 1.13E+07 | 1.25E+07 | 3.18E+07 | 0.83 |
| Com_324_neg | 4-Pyridoxic acid | 6.03E+08 | 5.15E+08 | 3.81E+08 | 2.75E+08 | 7.18E+08 | 4.91E+08 | 7.00E+08 | 4.89E+08 | 5.68E+08 | 5.46E+08 | 0.42 |
| Com_32513_pos | Pyrithioxin | 2.63E+06 | 3.26E+06 | 3.73E+06 | 1.36E+06 | 2.26E+06 | 2.42E+06 | 2.29E+06 | 1.77E+06 | 2.63E+06 | 1.75E+06 | 0.45 |
| Com_3252_pos | 2-Arachidonyl glycerol ether | 9.35E+06 | 7.64E+06 | 1.81E+08 | 1.49E+07 | 7.97E+06 | 7.60E+06 | 2.69E+07 | 9.16E+06 | 9.16E+06 | 2.34E+07 | 0.70 |
| Com_3256_pos | JWH 412 N-(5-hydroxypentyl) metabolite | 1.70E+06 | 4.62E+06 | 5.80E+06 | 1.32E+08 | 9.16E+06 | 1.49E+08 | 1.21E+07 | 1.33E+08 | 1.98E+06 | 5.32E+06 | 0.52 |
| Com_3257_neg | 7-Methylxanthine | 1.56E+07 | 1.40E+07 | 5.90E+07 | 1.98E+07 | 1.47E+07 | 1.05E+07 | 2.59E+07 | 2.08E+07 | 1.26E+07 | 3.00E+07 | 0.75 |
| Com_325_neg | 2-Hydroxyvaleric acid | 2.87E+07 | 5.16E+07 | 2.02E+08 | 2.91E+08 | 2.92E+08 | 2.03E+08 | 8.02E+08 | 9.96E+08 | 3.85E+08 | 2.55E+08 | 0.06 |
| Com_3264_pos | Glycylproline | 7.31E+07 | 7.77E+07 | 5.21E+07 | 7.84E+07 | 1.39E+08 | 1.41E+08 | 4.93E+07 | 1.74E+08 | 4.60E+07 | 7.35E+07 | 0.88 |
| Com_3267_pos | (12Z)-9,10,11-trihydroxyoctadec-12-enoic acid | 7.34E+07 | 9.54E+07 | 1.37E+08 | 8.48E+07 | 1.43E+08 | 8.41E+07 | 8.77E+07 | 1.10E+08 | 5.58E+07 | 5.49E+07 | 0.14 |
| Com_3275_neg | 4-Hydroxy-3-methylbenzoic acid | 1.38E+07 | 2.86E+07 | 1.79E+07 | 1.20E+07 | 1.71E+07 | 1.54E+07 | 2.64E+07 | 5.31E+07 | 1.03E+07 | 1.82E+07 | 0.54 |
| Com_3284_pos | 4-Hydroxyisoleucine | 2.16E+07 | 2.40E+07 | 6.55E+07 | 6.14E+07 | 5.35E+07 | 4.73E+07 | 5.71E+07 | 3.20E+07 | 6.03E+07 | 1.72E+08 | 0.29 |
| Com_3287_pos | Stearoyl ethanolamide | 4.36E+07 | 7.93E+07 | 1.78E+08 | 9.26E+07 | 5.75E+07 | 3.02E+07 | 8.09E+07 | 1.09E+08 | 5.90E+07 | 1.14E+08 | 0.73 |
| Com_3289_neg | Orotic acid | 1.06E+06 | 1.89E+06 | 1.19E+07 | 4.68E+07 | 7.06E+06 | 8.41E+06 | 1.56E+07 | 9.37E+06 | 7.90E+06 | 9.15E+06 | 0.51 |
| Com_32989_pos | N-{6-[(5-chloro-3-pyridyl)oxy]-3-pyridyl}-N'-methylurea | 2.10E+06 | 1.61E+06 | 1.23E+06 | 2.80E+06 | 1.81E+06 | 8.03E+05 | 2.73E+06 | 2.35E+06 | 1.30E+06 | 1.97E+06 | 0.73 |
| Com_32_neg | cholesteryl sulfate | 6.26E+09 | 5.71E+09 | 6.01E+09 | 3.90E+09 | 1.26E+09 | 4.11E+09 | 5.26E+09 | 3.89E+09 | 6.77E+09 | 1.11E+10 | 0.35 |
| Com_3310_neg | 11,12-Epoxy-(5Z,8Z,11Z)-icosatrienoic acid | 3.77E+07 | 2.20E+07 | 5.77E+07 | 2.39E+07 | 2.96E+07 | 2.69E+07 | 5.41E+07 | 4.10E+07 | 2.18E+07 | 2.54E+07 | 0.99 |
| Com_3312_pos | 1,4-dihydroxy-1,4-dimethyl-7-(propan-2-ylidene)-decahydroazulen-6-one | 1.61E+08 | 3.82E+07 | 8.50E+06 | 3.76E+07 | 2.28E+07 | 1.45E+08 | 1.78E+07 | 1.88E+07 | 3.12E+07 | 7.20E+07 | 0.78 |
| Com_33209_pos | N2-tetrahydrofuran-2-ylmethyl-4-(4-fluorophenyl)-1,3-thiazol-2-amine | 6.32E+05 | 6.23E+05 | 5.94E+05 | 6.97E+05 | 1.03E+06 | 3.07E+06 | 6.68E+05 | 3.49E+06 | 5.47E+05 | 7.43E+05 | 0.23 |
| Com_33218_pos | Glycohyocholic acid Sodium salt | 1.79E+06 | 2.70E+06 | 7.43E+05 | 1.39E+06 | 1.16E+06 | 2.14E+06 | 1.16E+06 | 2.60E+06 | 1.32E+06 | 3.58E+06 | 0.30 |
| Com_3324_neg | Pyridoxal | 2.41E+07 | 6.15E+06 | 3.34E+07 | 2.44E+07 | 2.26E+07 | 2.47E+07 | 7.05E+07 | 1.66E+07 | 1.13E+07 | 2.44E+07 | 0.63 |
| Com_3340_pos | Cytidine | 1.13E+08 | 1.22E+08 | 1.74E+08 | 6.53E+07 | 4.24E+07 | 1.24E+08 | 1.28E+08 | 6.29E+07 | 6.46E+07 | 1.43E+08 | 0.84 |
| Com_3342_pos | 4-Aminobenzoic acid | 1.13E+08 | 1.42E+08 | 6.25E+07 | 3.87E+07 | 1.17E+08 | 3.12E+07 | 6.89E+07 | 4.14E+07 | 3.74E+07 | 6.70E+07 | 0.08 |
| Com_33530_pos | S-(Methyl)Glutathione | 1.06E+06 | 1.97E+06 | 1.73E+06 | 3.26E+06 | 1.41E+06 | 1.76E+06 | 1.49E+06 | 1.50E+06 | 2.15E+06 | 1.14E+06 | 0.62 |
| Com_335_pos | 4',7-Dihydroxyflavanone | 2.09E+08 | 2.86E+08 | 1.85E+09 | 1.51E+08 | 3.54E+08 | 1.99E+09 | 3.65E+08 | 7.54E+08 | 2.31E+07 | 5.83E+08 | 0.96 |
| Com_3361_neg | FAHFA (16:0/18:2) | 3.53E+06 | 6.06E+06 | 5.65E+07 | 3.04E+07 | 9.24E+05 | 4.18E+06 | 3.80E+07 | 1.02E+07 | 1.72E+07 | 9.59E+06 | 0.64 |
| Com_3369_neg | N-Acetyl-α-D-glucosamine 1-phosphate | 2.12E+06 | 1.60E+06 | 5.64E+07 | 3.55E+07 | 1.21E+07 | 2.31E+06 | 5.43E+06 | 4.51E+06 | 1.92E+06 | 5.18E+06 | 0.25 |
| Com_3369_pos | Lagochilin | 7.66E+07 | 1.13E+08 | 1.19E+08 | 5.97E+07 | 4.80E+07 | 6.08E+07 | 7.03E+07 | 9.82E+06 | 1.46E+08 | 9.13E+07 | 0.53 |
| Com_336_pos | 9-Oxo-10(E),12(E)-octadecadienoic acid | 1.93E+09 | 1.03E+09 | 1.61E+09 | 1.16E+09 | 1.47E+09 | 6.62E+08 | 2.17E+09 | 2.02E+09 | 2.05E+09 | 1.04E+09 | 0.93 |
| Com_3371_neg | Ribitol | 7.40E+06 | 1.15E+07 | 2.44E+07 | 1.77E+07 | 1.65E+07 | 3.15E+07 | 5.44E+07 | 4.50E+07 | 2.33E+07 | 1.73E+07 | 0.03 |
| Com_3380_pos | 2,4-dihydroxyheptadec-16-en-1-yl acetate | 4.65E+07 | 3.64E+07 | 2.74E+07 | 7.04E+07 | 7.38E+07 | 3.74E+07 | 9.08E+07 | 1.69E+08 | 4.96E+07 | 5.55E+07 | 0.29 |
| Com_3387_neg | Salicylic acid | 1.77E+07 | 2.96E+07 | 1.15E+07 | 8.35E+06 | 3.57E+07 | 1.65E+07 | 3.01E+07 | 2.62E+07 | 7.55E+06 | 1.56E+07 | 0.93 |
| Com_3392_neg | Citric acid | 4.17E+06 | 5.10E+06 | 5.58E+07 | 1.43E+07 | 9.08E+06 | 1.10E+07 | 3.67E+07 | 3.07E+07 | 1.11E+07 | 1.16E+07 | 0.41 |
| Com_33979_pos | 5-S-cysteinyldopa | 5.88E+05 | 8.16E+05 | 1.16E+06 | 8.39E+05 | 8.33E+05 | 2.82E+06 | 1.27E+06 | 1.72E+06 | 6.50E+05 | 8.29E+05 | 0.18 |
| Com_3398_pos | Xanthurenic acid | 1.66E+08 | 1.32E+08 | 1.58E+08 | 7.68E+07 | 1.35E+08 | 1.28E+08 | 1.34E+08 | 1.01E+08 | 1.13E+08 | 9.04E+07 | 0.39 |
| Com_3404_neg | N-Acetyl-L-carnosine | 9.79E+06 | 1.36E+07 | 2.24E+07 | 1.16E+07 | 2.36E+07 | 1.85E+07 | 5.00E+07 | 3.29E+07 | 2.87E+07 | 1.19E+07 | 0.14 |
| Com_3406_neg | Δ17-6-keto prostaglandin F1α | 2.02E+07 | 2.41E+07 | 3.14E+07 | 2.40E+07 | 3.46E+07 | 3.09E+07 | 4.43E+07 | 2.59E+07 | 2.83E+07 | 3.60E+07 | 0.16 |
| Com_3411_pos | N1-[4-hydroxy-6-(methoxymethyl)pyrimidin-2-yl]acetamide | 4.40E+07 | 4.41E+07 | 1.69E+08 | 5.37E+07 | 7.71E+07 | 9.64E+07 | 3.33E+07 | 1.38E+08 | 8.35E+07 | 5.24E+07 | 0.85 |
| Com_3415_neg | LPC 20:5 | 1.76E+06 | 1.47E+06 | 2.23E+06 | 1.74E+06 | 4.19E+07 | 6.28E+06 | 2.48E+06 | 2.81E+07 | 1.18E+06 | 1.89E+06 | 0.85 |
| Com_3416_pos | Gly-Val | 3.71E+07 | 9.94E+07 | 9.24E+06 | 1.00E+08 | 8.34E+07 | 8.17E+07 | 6.07E+07 | 1.67E+08 | 8.12E+06 | 1.12E+08 | 0.79 |
| Com_3417_pos | (9cis)-Retinal | 3.82E+07 | 3.46E+07 | 2.03E+07 | 1.53E+08 | 5.50E+07 | 4.45E+07 | 7.87E+07 | 5.38E+07 | 5.80E+07 | 4.65E+07 | 0.67 |
| Com_3428_pos | 16,16-Dimethyl prostaglandin A1 | 1.58E+08 | 1.37E+08 | 1.18E+08 | 1.13E+08 | 9.99E+07 | 1.19E+08 | 6.30E+07 | 7.83E+07 | 9.92E+07 | 1.11E+08 | 0.07 |
| Com_3439_neg | 5,7-Dihydroxy-2-(3-hydroxy-4-methoxyphenyl)chroman-4-one | 1.17E+06 | 1.38E+06 | 1.46E+06 | 4.15E+06 | 2.57E+06 | 3.05E+07 | 2.26E+07 | 1.18E+07 | 2.26E+06 | 6.96E+06 | 0.02 |
| Com_3441_pos | 15-Acetyldeoxynivalenol | 5.04E+06 | 1.78E+07 | 1.67E+08 | 6.43E+06 | 7.29E+07 | 4.39E+07 | 1.16E+08 | 1.06E+08 | 1.07E+07 | 7.93E+06 | 0.68 |
| Com_34427_pos | Enrofloxacin | 6.33E+05 | 1.41E+06 | 1.01E+06 | 1.31E+06 | 1.07E+06 | 1.21E+06 | 1.56E+06 | 3.23E+06 | 6.13E+05 | 1.42E+06 | 0.38 |
| Com_344_pos | Urocanic acid | 3.89E+08 | 4.01E+08 | 1.77E+09 | 1.34E+09 | 2.42E+08 | 3.67E+08 | 2.29E+09 | 3.25E+08 | 1.10E+09 | 2.27E+09 | 0.50 |
| Com_345_neg | Melibiose | 4.70E+07 | 1.71E+08 | 2.94E+08 | 2.71E+07 | 7.80E+08 | 2.11E+07 | 1.40E+08 | 2.42E+08 | 2.30E+07 | 4.31E+07 | 0.31 |
| Com_3462_pos | N-Lauroylsarcosine | 6.12E+07 | 7.96E+07 | 5.53E+07 | 1.05E+08 | 6.22E+07 | 1.37E+08 | 1.48E+08 | 5.83E+07 | 3.56E+07 | 3.90E+07 | 0.96 |
| Com_346_pos | Nootkatone | 3.97E+08 | 3.28E+08 | 3.63E+08 | 2.10E+09 | 7.57E+08 | 4.12E+08 | 8.33E+08 | 4.71E+08 | 5.39E+08 | 4.85E+08 | 0.77 |
| Com_3475_neg | Tetradecanedioic acid | 2.58E+07 | 2.60E+07 | 1.85E+07 | 2.49E+07 | 1.27E+07 | 1.55E+07 | 2.38E+07 | 1.24E+07 | 2.79E+07 | 3.91E+07 | 0.85 |
| Com_3476_neg | 4-Methylcatechol | 4.60E+06 | 3.82E+06 | 5.39E+07 | 3.46E+06 | 5.68E+06 | 5.84E+06 | 5.27E+06 | 2.45E+06 | 3.00E+06 | 4.24E+06 | 0.32 |
| Com_3479_neg | Uridine monophosphate (UMP) | 4.68E+05 | 3.22E+05 | 5.39E+07 | 4.84E+05 | 5.75E+05 | 1.42E+06 | 6.47E+05 | 6.59E+05 | 2.92E+05 | 4.48E+05 | 0.53 |
| Com_347_neg | 4-Hydroxybutyric acid (GHB) | 1.20E+08 | 1.94E+08 | 3.61E+08 | 3.08E+08 | 1.90E+08 | 5.71E+08 | 1.18E+09 | 9.21E+08 | 1.99E+08 | 3.64E+08 | 0.05 |
| Com_3480_neg | Riboflavin | 7.67E+06 | 6.80E+06 | 1.46E+07 | 1.14E+07 | 4.09E+07 | 7.69E+06 | 2.89E+07 | 2.66E+07 | 1.85E+07 | 2.36E+07 | 0.35 |
| Com_34898_pos | Lysope 14:0 | 3.28E+06 | 1.38E+06 | 4.14E+05 | 3.32E+05 | 1.33E+06 | 4.29E+05 | 3.76E+05 | 3.36E+05 | 6.52E+05 | 3.35E+05 | 0.12 |
| Com_34_pos | Hypoxanthine | 7.41E+09 | 1.66E+10 | 8.34E+09 | 1.41E+10 | 1.28E+10 | 1.22E+10 | 2.02E+10 | 8.63E+09 | 1.69E+10 | 2.18E+10 | 0.25 |
| Com_3513_pos | N-Acetyl-aspartic acid | 6.51E+07 | 9.36E+07 | 1.16E+08 | 4.33E+07 | 4.52E+07 | 1.34E+08 | 5.62E+07 | 1.18E+08 | 7.04E+07 | 1.03E+08 | 0.26 |
| Com_3518_neg | Suberic acid | 2.87E+07 | 1.95E+07 | 3.33E+07 | 9.41E+06 | 2.72E+07 | 2.96E+07 | 2.28E+07 | 7.36E+06 | 6.20E+06 | 2.06E+07 | 0.33 |
| Com_3518_pos | LPE 20:5 | 3.92E+06 | 2.00E+07 | 1.51E+06 | 2.66E+06 | 1.29E+08 | 5.58E+07 | 1.31E+07 | 4.19E+07 | 1.30E+06 | 1.36E+06 | 0.96 |
| Com_3521_pos | DL-3,4-Dihydroxyphenyl glycol | 7.31E+07 | 8.18E+07 | 7.77E+07 | 1.07E+08 | 7.37E+07 | 6.38E+07 | 7.97E+07 | 1.60E+08 | 9.38E+07 | 1.27E+08 | 0.32 |
| Com_3537_pos | N-[2-chloro-6-(trifluoromethoxy)phenyl]-2,2-dimethylpropanamide | 8.78E+07 | 6.73E+07 | 5.33E+07 | 3.38E+07 | 1.28E+08 | 3.18E+07 | 1.18E+08 | 1.05E+08 | 2.17E+07 | 4.60E+07 | 0.55 |
| Com_35470_pos | Oxymatrine | 2.52E+06 | 2.37E+06 | 2.30E+05 | 4.20E+05 | 5.26E+05 | 6.68E+05 | 2.61E+05 | 2.64E+05 | 4.07E+05 | 1.28E+06 | 0.40 |
| Com_35679_pos | 3-(2-Naphthyl)-D-Alanine | 1.85E+06 | 1.43E+06 | 1.77E+06 | 1.50E+06 | 2.26E+06 | 2.33E+06 | 2.13E+06 | 1.97E+06 | 2.29E+06 | 1.42E+06 | 0.29 |
| Com_3567_neg | 3-(2-thienyl)-1,2,4-oxadiazole-5-carbohydrazide | 1.38E+07 | 2.70E+07 | 1.35E+07 | 1.23E+07 | 2.84E+07 | 2.74E+07 | 3.63E+07 | 1.67E+07 | 2.69E+07 | 1.88E+07 | 0.21 |
| Com_3577_pos | ACar 16:1 | 7.39E+06 | 5.73E+07 | 4.03E+07 | 4.26E+06 | 4.47E+07 | 1.31E+08 | 3.21E+06 | 1.20E+08 | 4.97E+06 | 5.99E+06 | 0.88 |
| Com_3578_neg | MAG (18:3) | 3.01E+07 | 1.63E+07 | 1.54E+06 | 4.18E+07 | 1.27E+07 | 1.48E+07 | 3.73E+07 | 2.05E+07 | 1.29E+07 | 4.19E+07 | 0.42 |
| Com_357_pos | Taurine | 1.45E+09 | 7.85E+08 | 8.88E+08 | 3.94E+08 | 1.80E+09 | 1.13E+09 | 2.86E+08 | 1.88E+09 | 1.29E+09 | 8.69E+08 | 0.98 |
| Com_358_pos | 2-[(3S)-1-Benzyl-3-pyrrolidinyl]-1,3-benzothiazole | 7.88E+07 | 1.83E+08 | 1.89E+08 | 1.53E+09 | 2.13E+08 | 1.86E+09 | 1.46E+08 | 1.52E+09 | 1.45E+08 | 3.77E+08 | 0.41 |
| Com_3601_neg | Sinapinic acid | 5.08E+06 | 1.09E+06 | 1.07E+07 | 1.34E+07 | 4.00E+06 | 2.88E+07 | 1.71E+07 | 3.12E+06 | 1.36E+07 | 1.32E+07 | 0.16 |
| Com_3613_pos | Cuminaldehyde | 1.49E+08 | 1.19E+08 | 1.42E+08 | 1.20E+08 | 6.04E+07 | 1.30E+08 | 1.47E+08 | 9.23E+07 | 1.08E+08 | 1.22E+08 | 0.80 |
| Com_36158_pos | 4,7,8-trimethoxyfuro[2,3-b]quinoline | 2.53E+05 | 4.64E+05 | 9.96E+05 | 3.56E+05 | 8.66E+05 | 1.31E+06 | 7.08E+05 | 7.28E+05 | 2.28E+06 | 4.51E+05 | 0.16 |
| Com_3618_pos | 3-Nitro-L-Tyrosine | 9.49E+07 | 1.02E+08 | 1.57E+08 | 5.37E+07 | 3.69E+07 | 1.11E+08 | 1.23E+08 | 4.68E+07 | 5.77E+07 | 1.31E+08 | 0.78 |
| Com_3628_neg | Sodium cholate | 2.42E+07 | 1.72E+07 | 5.67E+06 | 6.88E+06 | 1.40E+07 | 1.87E+07 | 2.56E+07 | 3.83E+07 | 2.49E+07 | 4.31E+07 | 0.03 |
| Com_3629_neg | LPA 20:4 | 6.57E+05 | 1.22E+06 | 9.28E+05 | 8.12E+05 | 3.87E+07 | 4.83E+05 | 1.03E+06 | 9.11E+06 | 4.78E+05 | 7.81E+05 | 0.59 |
| Com_3633_pos | L-Leucyl-L-alanine Hydrate | 1.83E+07 | 9.35E+06 | 1.26E+06 | 4.11E+07 | 3.64E+07 | 1.29E+08 | 4.02E+07 | 4.23E+07 | 2.67E+06 | 3.21E+07 | 0.39 |
| Com_3635_pos | (+)-alpha-Lipoic acid | 2.87E+07 | 5.49E+07 | 6.60E+07 | 1.06E+08 | 6.28E+07 | 6.72E+07 | 6.45E+07 | 1.06E+08 | 5.65E+07 | 1.50E+08 | 0.25 |
| Com_3639_pos | N-Acetylglycine | 2.13E+07 | 3.07E+07 | 4.54E+07 | 9.07E+07 | 4.49E+07 | 1.29E+08 | 3.70E+07 | 1.22E+08 | 2.26E+07 | 6.93E+07 | 0.36 |
| Com_3641_pos | (2E)-1-(2-hydroxy-3,4,5,6-tetramethoxyphenyl)-3-phenylprop-2-en-1-one | 1.12E+07 | 6.92E+06 | 3.41E+06 | 5.77E+07 | 7.99E+06 | 4.39E+07 | 7.08E+07 | 9.51E+06 | 1.33E+08 | 8.06E+07 | 0.04 |
| Com_3647_neg | (3beta,9xi)-3-(beta-D-Glucopyranosyloxy)-14-hydroxycard-20(22)-enolide | 2.65E+05 | 3.76E+05 | 3.39E+05 | 3.05E+05 | 2.87E+05 | 2.83E+07 | 3.76E+05 | 2.85E+06 | 2.04E+05 | 5.08E+06 | 0.11 |
| Com_36490_pos | Methyl 3-indolyacetate | 7.37E+05 | 8.94E+05 | 7.56E+05 | 9.27E+05 | 1.01E+06 | 1.13E+06 | 1.10E+06 | 2.54E+06 | 6.34E+05 | 1.55E+06 | 0.17 |
| Com_3654_pos | 16,16-Dimethyl prostaglandin A2 | 1.37E+08 | 8.09E+07 | 1.49E+07 | 3.73E+07 | 6.44E+07 | 1.28E+08 | 4.93E+07 | 2.59E+07 | 5.46E+07 | 6.64E+07 | 0.86 |
| Com_3660_neg | Hexadecanedioic acid | 3.57E+06 | 3.92E+06 | 2.11E+07 | 2.95E+06 | 3.71E+06 | 2.82E+07 | 3.06E+06 | 6.12E+06 | 2.39E+06 | 6.01E+06 | 0.77 |
| Com_3666_neg | LPG 16:1 | 5.76E+06 | 6.09E+06 | 6.48E+06 | 7.09E+06 | 3.82E+07 | 5.68E+06 | 9.39E+06 | 3.74E+07 | 5.91E+05 | 2.86E+06 | 0.48 |
| Com_3672_neg | FAHFA (20:4/18:1) | 1.65E+07 | 1.54E+07 | 1.21E+07 | 3.44E+07 | 2.38E+07 | 3.35E+06 | 1.93E+07 | 4.56E+07 | 1.99E+07 | 1.08E+07 | 0.58 |
| Com_367_pos | 5-Hydroxyindole-3-acetic acid | 1.81E+09 | 1.46E+09 | 2.41E+08 | 4.12E+08 | 1.05E+09 | 3.28E+08 | 3.17E+08 | 6.24E+07 | 1.88E+09 | 1.51E+09 | 0.48 |
| Com_3682_pos | GLK | 4.62E+07 | 7.50E+07 | 3.50E+07 | 9.80E+07 | 3.99E+07 | 8.49E+07 | 4.75E+07 | 6.86E+07 | 3.78E+07 | 1.48E+08 | 0.46 |
| Com_3690_neg | LPA 16:0 | 1.29E+07 | 1.15E+07 | 8.79E+06 | 2.39E+07 | 2.65E+07 | 9.86E+06 | 3.40E+07 | 4.54E+07 | 7.94E+06 | 1.41E+07 | 0.73 |
| Com_3690_pos | PB-22 N-(4-Hydroxypentyl)-3-carboxyindole metabolite | 2.48E+07 | 6.17E+07 | 4.47E+07 | 1.63E+07 | 1.91E+07 | 1.27E+08 | 2.62E+07 | 1.49E+08 | 2.36E+07 | 1.77E+07 | 0.41 |
| Com_369_neg | Methionine | 2.19E+08 | 4.40E+08 | 3.41E+08 | 5.79E+08 | 4.29E+08 | 3.02E+08 | 5.62E+08 | 6.99E+08 | 1.87E+08 | 8.51E+08 | 0.62 |
| Com_36_neg | Pentadecanoic acid | 1.60E+09 | 3.02E+09 | 1.24E+10 | 2.99E+09 | 2.92E+09 | 1.03E+09 | 5.32E+09 | 1.16E+09 | 2.60E+09 | 2.32E+09 | 0.28 |
| Com_36_pos | 7-Ketodeoxycholic acid | 4.07E+09 | 2.96E+09 | 1.02E+09 | 4.44E+08 | 4.28E+09 | 1.78E+10 | 9.90E+08 | 4.62E+09 | 6.05E+09 | 3.51E+09 | 0.22 |
| Com_3710_pos | O-Aceyl-L-Serine | 3.00E+07 | 3.81E+07 | 1.93E+07 | 2.78E+07 | 1.86E+07 | 1.69E+07 | 3.39E+07 | 1.71E+07 | 8.14E+07 | 1.47E+08 | 0.35 |
| Com_3711_neg | D-3-Phenyllactic acid | 1.61E+07 | 1.72E+07 | 4.96E+07 | 4.49E+06 | 1.61E+07 | 5.70E+06 | 7.61E+06 | 6.41E+06 | 9.13E+06 | 6.08E+06 | 0.09 |
| Com_3712_pos | Hydroxyprogesterone caproate | 2.26E+07 | 1.17E+07 | 1.14E+08 | 4.87E+07 | 3.49E+07 | 4.41E+07 | 3.34E+07 | 5.20E+06 | 1.20E+08 | 1.46E+08 | 0.79 |
| Com_3716_pos | (2E)-6-hydroxy-2-methyl-6-(4-methylphenyl)hept-2-enoic acid | 3.44E+07 | 2.86E+07 | 2.65E+07 | 1.79E+07 | 1.20E+08 | 2.09E+07 | 5.84E+07 | 7.39E+07 | 1.41E+07 | 5.79E+07 | 0.91 |
| Com_3718_pos | L-Alanyl-L-Lysine | 2.54E+07 | 8.09E+07 | 3.93E+06 | 1.45E+07 | 6.45E+07 | 1.25E+08 | 9.58E+06 | 1.01E+08 | 2.69E+06 | 3.24E+07 | 0.94 |
| Com_372_pos | LPE 18:2 | 8.62E+06 | 4.41E+08 | 2.71E+07 | 2.79E+07 | 1.74E+09 | 5.77E+08 | 5.90E+07 | 1.54E+09 | 6.64E+06 | 9.41E+06 | 0.96 |
| Com_3733_pos | Avocadyne 1-acetate | 9.64E+07 | 1.23E+08 | 1.29E+08 | 8.51E+07 | 1.13E+08 | 8.69E+07 | 8.95E+07 | 8.96E+07 | 6.64E+07 | 8.99E+07 | 0.03 |
| Com_3741_pos | 3-Methoxybenzaldehyde | 2.05E+07 | 1.74E+07 | 1.26E+07 | 5.87E+07 | 1.44E+07 | 5.66E+07 | 1.47E+08 | 8.33E+07 | 1.28E+08 | 9.46E+07 | 0.00 |
| Com_3745_pos | 4-Guanidinobutyric acid | 1.76E+07 | 2.11E+07 | 1.50E+08 | 8.88E+06 | 3.12E+07 | 2.95E+07 | 6.63E+07 | 5.23E+07 | 1.20E+07 | 2.00E+07 | 0.87 |
| Com_37521_pos | RPH | 1.23E+06 | 1.30E+06 | 1.22E+06 | 1.29E+06 | 1.52E+06 | 1.11E+06 | 1.49E+06 | 1.84E+06 | 1.32E+06 | 2.15E+06 | 0.24 |
| Com_3759_pos | 3,6-diphenyl-1,2-dihydro-1,2,4,5-tetraazine | 1.07E+08 | 1.22E+08 | 5.50E+07 | 2.64E+07 | 3.45E+07 | 1.17E+08 | 1.21E+08 | 4.35E+07 | 5.47E+07 | 1.77E+07 | 0.97 |
| Com_375_neg | Ethyl-β-D-glucuronide | 6.36E+07 | 1.13E+08 | 7.99E+07 | 5.68E+07 | 7.05E+08 | 5.70E+07 | 1.70E+08 | 2.90E+08 | 7.01E+07 | 5.22E+07 | 0.78 |
| Com_3761_pos | Hydrocortisone acetate | 3.75E+07 | 5.68E+07 | 2.61E+07 | 9.73E+07 | 1.25E+07 | 1.23E+08 | 8.42E+07 | 6.75E+07 | 5.58E+07 | 4.03E+07 | 0.16 |
| Com_37794_pos | PNK | 8.15E+05 | 1.25E+06 | 1.51E+06 | 1.84E+06 | 8.64E+05 | 1.04E+06 | 1.39E+06 | 2.10E+06 | 9.93E+05 | 1.52E+06 | 0.57 |
| Com_3792_pos | 2-(acetyloxy)-3-amino-1-[1,2-di(acetyloxy)ethyl]-3-oxopropyl acetate | 3.20E+06 | 8.43E+06 | 3.13E+06 | 6.18E+06 | 3.79E+06 | 1.22E+08 | 5.40E+06 | 4.63E+06 | 6.82E+06 | 1.47E+07 | 0.17 |
| Com_3793_pos | 4-(1H-pyrazol-1-yl)-N,N-bis(2-pyridinylmethyl)benzenesulfonamide | 8.84E+07 | 1.07E+08 | 7.53E+06 | 1.32E+08 | 1.95E+07 | 1.22E+08 | 1.38E+08 | 3.22E+07 | 6.41E+07 | 8.51E+07 | 0.40 |
| Com_3794_pos | N-Acetylornithine | 6.35E+07 | 6.02E+07 | 1.48E+08 | 8.45E+07 | 5.28E+07 | 7.39E+07 | 5.66E+07 | 3.67E+07 | 5.12E+07 | 1.06E+08 | 0.41 |
| Com_379_pos | Inosine | 2.34E+08 | 1.83E+08 | 7.53E+08 | 1.92E+09 | 5.45E+08 | 1.17E+08 | 5.04E+08 | 8.27E+08 | 9.00E+08 | 1.36E+09 | 0.86 |
| Com_3803_pos | 2-(acetylamino)-3-[4-(acetylamino)phenyl]acrylic acid | 1.13E+07 | 2.76E+07 | 1.48E+08 | 2.02E+07 | 3.07E+07 | 2.55E+07 | 1.57E+07 | 2.79E+07 | 7.57E+06 | 9.68E+06 | 0.20 |
| Com_3806_neg | indole-5,6-quinone | 2.11E+07 | 2.54E+07 | 1.95E+07 | 2.76E+07 | 3.05E+07 | 2.45E+07 | 2.75E+07 | 3.81E+07 | 1.43E+07 | 2.14E+07 | 0.91 |
| Com_3808_pos | Diaminopimelic acid | 1.79E+07 | 1.94E+07 | 7.67E+07 | 1.34E+08 | 2.08E+07 | 1.51E+07 | 8.54E+07 | 2.47E+07 | 3.04E+07 | 3.64E+07 | 0.78 |
| Com_3816_pos | 3-[4-methyl-1-(2-methylpropanoyl)-3-oxocyclohexyl]butanoic acid | 1.18E+08 | 1.16E+08 | 4.89E+06 | 7.00E+07 | 3.85E+07 | 1.86E+07 | 2.70E+07 | 3.34E+06 | 4.73E+07 | 1.42E+08 | 0.53 |
| Com_381_neg | Decanoic acid | 2.26E+08 | 2.03E+08 | 4.88E+08 | 2.91E+08 | 6.95E+08 | 1.33E+08 | 3.33E+08 | 3.88E+08 | 2.84E+08 | 3.07E+08 | 0.48 |
| Com_38357_pos | FPH | 2.97E+05 | 3.11E+05 | 2.71E+05 | 1.77E+06 | 2.80E+05 | 4.60E+05 | 2.69E+05 | 3.39E+05 | 2.19E+05 | 5.97E+05 | 0.70 |
| Com_38511_pos | 2-(1H-indol-3-yl)-3-[4-(trifluoromethyl)phenyl]acrylonitrile | 1.24E+06 | 6.67E+05 | 1.78E+06 | 1.01E+06 | 6.25E+05 | 6.41E+05 | 2.30E+06 | 1.35E+06 | 4.68E+05 | 1.15E+06 | 0.94 |
| Com_3860_neg | L-Cysteine-S-sulfate | 3.79E+06 | 4.02E+06 | 1.09E+07 | 1.05E+07 | 2.63E+06 | 1.37E+07 | 1.04E+07 | 4.25E+07 | 6.43E+06 | 6.12E+06 | 0.12 |
| Com_3874_pos | ACar 14:1 | 6.22E+06 | 1.45E+07 | 2.18E+07 | 1.33E+06 | 1.23E+07 | 1.19E+08 | 9.09E+05 | 3.51E+07 | 8.69E+05 | 1.35E+06 | 0.74 |
| Com_3879_pos | Purine | 4.50E+06 | 2.86E+07 | 3.87E+07 | 2.09E+07 | 4.18E+06 | 2.66E+06 | 2.45E+07 | 1.23E+06 | 5.04E+07 | 1.39E+08 | 0.96 |
| Com_387_neg | 3,4-Dihydroxyphenylpropionic acid | 8.00E+06 | 1.68E+07 | 9.06E+08 | 4.52E+06 | 7.73E+07 | 6.34E+06 | 9.98E+06 | 9.58E+06 | 4.32E+06 | 6.66E+06 | 0.18 |
| Com_38881_pos | GNH | 1.42E+06 | 1.45E+06 | 1.32E+06 | 8.78E+05 | 1.24E+06 | 1.40E+06 | 8.61E+05 | 7.77E+05 | 1.10E+06 | 7.87E+05 | 0.12 |
| Com_38894_pos | 3-[(4-hydroxyphenyl)methyl]-octahydropyrrolo[1,2-a]pyrazine-1,4-dione | 7.32E+05 | 7.36E+05 | 1.09E+06 | 4.35E+05 | 1.21E+06 | 8.41E+05 | 5.47E+05 | 9.59E+05 | 4.52E+05 | 3.09E+05 | 0.27 |
| Com_390_neg | D-Raffinose | 4.28E+07 | 9.75E+07 | 4.93E+07 | 2.24E+07 | 6.84E+08 | 9.57E+07 | 2.03E+08 | 2.50E+08 | 2.25E+07 | 4.15E+07 | 0.93 |
| Com_3928_neg | LPG 13:0 | 1.13E+07 | 2.42E+07 | 7.79E+05 | 1.90E+07 | 1.08E+07 | 1.11E+06 | 2.35E+07 | 1.25E+07 | 1.98E+06 | 6.39E+06 | 0.59 |
| Com_39344_pos | VNK | 3.58E+05 | 1.31E+06 | 3.79E+05 | 3.23E+05 | 3.55E+05 | 3.71E+05 | 4.17E+05 | 3.74E+05 | 5.97E+05 | 5.24E+05 | 0.93 |
| Com_3938_pos | N-Desmethyltramadol | 1.07E+07 | 2.96E+06 | 1.42E+08 | 3.46E+06 | 9.76E+06 | 6.06E+06 | 6.59E+06 | 1.50E+07 | 9.26E+07 | 5.51E+06 | 0.88 |
| Com_393_neg | D-2-Aminoadipic acid | 9.51E+07 | 2.34E+08 | 6.22E+08 | 2.35E+08 | 3.36E+08 | 5.00E+08 | 2.13E+08 | 3.70E+08 | 1.75E+08 | 2.32E+08 | 0.84 |
| Com_394_neg | 3,8,9-trihydroxy-10-propyl-3,4,5,8,9,10-hexahydro-2H-oxecin-2-one | 3.94E+08 | 3.45E+08 | 2.05E+08 | 3.06E+08 | 3.67E+08 | 2.09E+08 | 3.00E+08 | 4.93E+07 | 4.62E+08 | 4.96E+08 | 0.53 |
| Com_3950_pos | o-Cresol | 4.40E+07 | 3.66E+07 | 3.99E+07 | 1.28E+08 | 5.53E+07 | 3.54E+07 | 5.52E+07 | 4.44E+07 | 5.64E+07 | 5.27E+07 | 0.66 |
| Com_39513_pos | Kinetin | 1.55E+05 | 1.50E+05 | 1.39E+06 | 9.72E+05 | 1.66E+05 | 1.28E+06 | 1.24E+06 | 6.18E+05 | 1.09E+06 | 7.31E+05 | 0.11 |
| Com_3959_pos | 2-[(3S)-1-Cyclobutyl-3-pyrrolidinyl]-5-fluoro-1H-benzimidazole | 5.63E+07 | 6.87E+07 | 2.86E+07 | 1.27E+08 | 3.04E+07 | 6.41E+07 | 5.52E+07 | 3.64E+07 | 7.73E+07 | 1.28E+08 | 0.55 |
| Com_39601_pos | 1-benzyl-3-butyl-4-hydroxy-6-phenylpyridin-2(1H)-one | 8.57E+05 | 1.25E+06 | 4.84E+05 | 3.12E+05 | 1.15E+06 | 4.11E+05 | 9.70E+05 | 1.13E+06 | 8.82E+05 | 5.72E+05 | 0.90 |
| Com_39639_pos | 5-Hydroxytryptophol | 5.48E+05 | 6.11E+05 | 8.13E+05 | 9.65E+05 | 1.20E+06 | 6.39E+05 | 9.98E+05 | 8.37E+05 | 9.07E+05 | 9.44E+05 | 0.67 |
| Com_3972_pos | 6 β-Hydroxycortisol | 2.13E+07 | 2.21E+07 | 2.92E+07 | 1.51E+07 | 1.88E+07 | 1.15E+08 | 2.81E+07 | 3.16E+07 | 2.13E+07 | 1.58E+07 | 0.28 |
| Com_397_pos | PC (14:1e/4:0) | 1.17E+07 | 5.12E+08 | 2.72E+07 | 1.56E+07 | 1.11E+09 | 2.80E+08 | 9.04E+07 | 2.03E+09 | 5.74E+06 | 6.63E+06 | 0.96 |
| Com_3988_neg | L-Homocystine | 2.69E+06 | 8.93E+06 | 8.02E+06 | 6.31E+05 | 3.40E+07 | 4.15E+05 | 8.88E+06 | 7.34E+06 | 6.68E+05 | 1.80E+06 | 0.32 |
| Com_398_pos | Monoolein | 1.20E+07 | 8.68E+08 | 1.68E+07 | 9.70E+06 | 4.93E+08 | 8.19E+06 | 1.12E+07 | 2.02E+09 | 7.45E+06 | 8.66E+06 | 0.58 |
| Com_3992_neg | Saccharin | 7.59E+06 | 8.06E+06 | 1.47E+07 | 3.61E+07 | 1.18E+07 | 9.94E+06 | 9.79E+05 | 3.10E+06 | 2.91E+06 | 6.45E+06 | 0.03 |
| Com_3993_neg | Corey Lactone Diol | 8.12E+06 | 1.52E+07 | 7.20E+06 | 1.85E+07 | 2.87E+07 | 2.50E+07 | 1.50E+07 | 2.16E+07 | 1.55E+07 | 1.51E+07 | 0.36 |
| Com_39962_pos | 8-(1,2-dihydroxy-3-methylbut-3-en-1-yl)-7-methoxy-2H-chromen-2-one | 2.29E+05 | 2.99E+05 | 1.81E+05 | 1.26E+06 | 1.04E+05 | 2.10E+05 | 3.10E+05 | 2.43E+05 | 3.33E+05 | 1.55E+05 | 0.77 |
| Com_39980_pos | N-METHYL (-)EPHEDRINE | 6.86E+05 | 3.86E+05 | 1.03E+06 | 4.18E+05 | 3.62E+05 | 1.14E+06 | 7.17E+05 | 8.64E+05 | 2.70E+05 | 3.34E+05 | 0.81 |
| Com_399_neg | D-(-)-Ribose | 2.01E+08 | 3.35E+08 | 4.52E+08 | 4.07E+08 | 4.67E+08 | 4.83E+08 | 1.00E+09 | 5.76E+08 | 1.64E+08 | 6.26E+08 | 0.38 |
| Com_4004_neg | Uracil 1-beta-D-arabinofuranoside | 2.83E+06 | 4.21E+06 | 1.24E+07 | 8.03E+06 | 7.91E+06 | 2.21E+07 | 2.19E+07 | 4.06E+07 | 4.50E+06 | 1.15E+07 | 0.08 |
| Com_4017_pos | Lysopc 16:1 | 8.36E+06 | 1.12E+08 | 8.85E+06 | 1.00E+07 | 7.50E+06 | 3.67E+06 | 9.22E+06 | 8.68E+06 | 1.95E+06 | 2.29E+06 | 0.08 |
| Com_4019_pos | Butein | 8.51E+06 | 3.68E+06 | 1.37E+08 | 4.68E+06 | 6.07E+06 | 1.87E+07 | 4.50E+06 | 2.40E+06 | 2.08E+06 | 6.22E+06 | 0.35 |
| Com_402_neg | Tyrosol | 7.98E+06 | 2.52E+07 | 8.53E+08 | 3.38E+06 | 1.24E+08 | 9.80E+06 | 6.90E+06 | 1.06E+07 | 6.32E+06 | 8.96E+06 | 0.21 |
| Com_402_pos | 2-Amino-1,3,4-octadecanetriol | 2.81E+08 | 5.42E+08 | 3.96E+07 | 2.09E+08 | 1.27E+09 | 1.67E+09 | 1.07E+09 | 5.63E+08 | 1.44E+08 | 2.70E+08 | 0.41 |
| Com_4030_pos | Maltotetraose | 2.59E+07 | 9.29E+07 | 1.63E+07 | 9.16E+06 | 1.08E+08 | 5.55E+07 | 1.17E+08 | 1.24E+08 | 1.40E+07 | 1.38E+07 | 0.69 |
| Com_403_neg | L-lysine | 3.05E+08 | 3.21E+08 | 4.07E+08 | 5.56E+08 | 3.96E+08 | 3.82E+08 | 5.88E+08 | 7.70E+08 | 2.24E+08 | 5.71E+08 | 0.47 |
| Com_404_neg | Tridecylic acid | 2.64E+08 | 3.77E+08 | 7.64E+08 | 5.44E+08 | 3.63E+08 | 1.78E+08 | 6.88E+08 | 3.44E+08 | 4.39E+08 | 3.62E+08 | 0.59 |
| Com_405_pos | Glutaric Acid | 1.93E+08 | 2.56E+08 | 2.06E+08 | 2.23E+08 | 4.21E+08 | 8.40E+08 | 5.01E+08 | 1.98E+09 | 2.03E+08 | 3.28E+08 | 0.11 |
| Com_4071_neg | N-Oleoyl dopamine | 4.47E+06 | 1.06E+07 | 5.96E+06 | 2.02E+07 | 1.68E+07 | 2.45E+07 | 5.50E+06 | 2.06E+06 | 1.26E+07 | 2.68E+07 | 0.99 |
| Com_407_pos | Leucylproline | 6.82E+08 | 1.47E+09 | 1.84E+08 | 1.01E+09 | 7.40E+08 | 1.65E+09 | 5.03E+08 | 1.08E+09 | 1.04E+08 | 1.19E+09 | 0.95 |
| Com_40859_pos | AMK | 3.59E+05 | 8.74E+05 | 8.13E+04 | 6.83E+05 | 3.45E+05 | 3.98E+05 | 3.43E+05 | 1.42E+05 | 1.34E+05 | 3.55E+05 | 0.46 |
| Com_4089_pos | Homo-Gamma-Linolenic Acid (C20:3) | 8.65E+07 | 1.10E+08 | 7.92E+07 | 5.07E+07 | 7.43E+07 | 9.26E+07 | 6.58E+07 | 5.80E+07 | 1.00E+08 | 1.20E+08 | 0.67 |
| Com_40960_pos | Cortodoxone | 2.21E+05 | 2.57E+05 | 2.69E+05 | 3.40E+05 | 8.25E+05 | 6.24E+04 | 3.09E+05 | 1.64E+05 | 1.56E+05 | 1.11E+05 | 0.04 |
| Com_41084_pos | Triacanthine | 1.03E+06 | 6.80E+05 | 5.00E+05 | 3.33E+05 | 4.55E+05 | 4.80E+05 | 4.52E+05 | 2.60E+05 | 3.21E+05 | 4.15E+05 | 0.13 |
| Com_41100_pos | Melanin | 1.54E+05 | 1.58E+05 | 3.73E+05 | 8.80E+05 | 2.41E+05 | 1.29E+05 | 8.12E+05 | 1.83E+05 | 1.99E+05 | 8.43E+05 | 0.85 |
| Com_4112_pos | 4-(Diethylamino)salicylaldehyde | 7.85E+07 | 1.09E+08 | 7.15E+07 | 3.42E+07 | 5.55E+07 | 4.74E+07 | 4.43E+07 | 3.40E+07 | 6.13E+07 | 5.51E+07 | 0.20 |
| Com_4137_neg | L-Ascorbate | 1.27E+07 | 5.71E+06 | 1.98E+07 | 1.53E+07 | 2.99E+07 | 1.72E+07 | 5.26E+07 | 8.41E+06 | 7.99E+06 | 2.34E+07 | 0.74 |
| Com_4140_pos | 4-[4-(methoxymethyl)-6-piperidinopyrimidin-2-yl]-2-methyl-1,3-thiazole | 2.27E+07 | 2.86E+07 | 6.13E+07 | 9.45E+07 | 4.50E+07 | 1.09E+08 | 4.64E+07 | 9.48E+07 | 3.06E+07 | 6.25E+07 | 0.36 |
| Com_4143_neg | Thromboxane B3 | 4.81E+06 | 4.19E+06 | 1.97E+06 | 2.31E+06 | 1.18E+07 | 2.39E+07 | 1.89E+07 | 4.79E+06 | 1.52E+06 | 1.22E+07 | 0.27 |
| Com_414_neg | Dl-Threitol | 3.99E+07 | 6.82E+07 | 9.59E+07 | 2.07E+08 | 1.28E+08 | 4.61E+08 | 9.27E+08 | 7.21E+08 | 9.91E+07 | 2.16E+08 | 0.03 |
| Com_4167_pos | 6-Aminonicotinamide | 3.68E+07 | 3.08E+07 | 1.11E+08 | 7.32E+07 | 2.68E+07 | 3.31E+07 | 1.38E+08 | 3.80E+07 | 6.35E+07 | 1.26E+08 | 0.42 |
| Com_4169_pos | 15-Deoxy-Δ12,14-prostaglandin A1 | 9.62E+07 | 7.68E+07 | 5.70E+07 | 5.04E+07 | 5.31E+07 | 9.91E+07 | 7.60E+07 | 6.14E+07 | 5.23E+07 | 1.26E+08 | 0.35 |
| Com_416_pos | DL-Arginine | 5.36E+08 | 9.96E+08 | 1.53E+08 | 7.57E+08 | 5.13E+08 | 5.52E+08 | 2.79E+08 | 1.95E+09 | 6.75E+07 | 1.25E+09 | 0.95 |
| Com_4170_neg | Prostaglandin F2alpha | 3.09E+07 | 1.52E+07 | 1.42E+07 | 1.76E+07 | 1.40E+07 | 1.25E+07 | 1.67E+07 | 5.92E+06 | 1.23E+07 | 1.88E+07 | 0.20 |
| Com_4191_neg | 2-Aminoadipic acid | 1.36E+07 | 2.24E+07 | 2.88E+07 | 2.01E+07 | 7.35E+06 | 7.14E+06 | 2.18E+07 | 8.60E+06 | 1.43E+07 | 3.30E+07 | 0.71 |
| Com_419_neg | β-D-Glucopyranuronic acid | 6.21E+07 | 9.07E+06 | 9.21E+07 | 1.34E+08 | 9.69E+07 | 4.55E+08 | 2.48E+08 | 6.15E+07 | 4.26E+07 | 3.42E+07 | 0.46 |
| Com_41_neg | β-Muricholic acid | 2.33E+09 | 2.06E+09 | 1.79E+09 | 2.38E+09 | 2.37E+09 | 6.12E+09 | 1.72E+09 | 1.81E+09 | 2.95E+09 | 3.92E+09 | 0.28 |
| Com_420_neg | Ornithine | 1.48E+08 | 1.62E+08 | 3.73E+08 | 6.49E+08 | 2.90E+08 | 2.30E+08 | 5.36E+08 | 3.09E+08 | 2.19E+08 | 6.89E+08 | 0.51 |
| Com_4210_pos | 7-methyl-3-nitroimidazo[1,2-a]pyridine | 1.13E+07 | 1.39E+07 | 4.06E+06 | 1.12E+07 | 3.27E+07 | 3.06E+07 | 8.05E+06 | 1.28E+08 | 2.26E+06 | 1.06E+07 | 0.77 |
| Com_4216_pos | Gamma-Glu-Leu | 2.02E+07 | 5.17E+07 | 6.14E+06 | 7.64E+07 | 3.24E+07 | 7.35E+07 | 3.89E+07 | 6.99E+07 | 1.30E+07 | 1.24E+08 | 0.33 |
| Com_4218_neg | LPS 18:1 | 1.40E+06 | 2.22E+07 | 3.57E+06 | 8.00E+06 | 1.47E+07 | 1.28E+06 | 5.73E+06 | 3.66E+07 | 1.56E+06 | 1.87E+06 | 0.50 |
| Com_4218_pos | All trans-Retinal | 1.48E+07 | 3.57E+07 | 4.98E+06 | 1.17E+08 | 6.31E+07 | 8.51E+06 | 1.09E+07 | 2.17E+07 | 2.23E+07 | 2.38E+07 | 0.37 |
| Com_4219_neg | Citrinin | 5.28E+06 | 9.06E+06 | 2.63E+07 | 3.38E+07 | 1.76E+07 | 1.15E+07 | 1.80E+07 | 2.40E+07 | 7.25E+06 | 1.22E+07 | 0.80 |
| Com_4223_pos | 5-(2,5-dihydroxyhexyl)oxolan-2-one | 9.51E+06 | 6.43E+06 | 7.95E+06 | 6.77E+06 | 6.03E+06 | 6.19E+06 | 1.56E+08 | 5.82E+06 | 6.46E+06 | 9.32E+06 | 0.42 |
| Com_4232_neg | FAHFA (2:0/24:1) | 1.81E+06 | 2.52E+06 | 6.90E+06 | 5.58E+06 | 1.10E+07 | 1.67E+06 | 6.82E+06 | 2.76E+06 | 3.15E+06 | 3.76E+07 | 0.84 |
| Com_4247_neg | lipoamide | 2.48E+07 | 1.85E+07 | 2.55E+07 | 1.80E+07 | 2.34E+07 | 1.55E+07 | 3.48E+07 | 2.12E+07 | 2.15E+07 | 2.11E+07 | 0.94 |
| Com_424_pos | Adenosine | 1.34E+09 | 1.58E+09 | 1.63E+09 | 1.65E+09 | 8.07E+07 | 1.28E+07 | 1.36E+07 | 3.66E+07 | 2.59E+08 | 8.47E+08 | 0.04 |
| Com_4254_pos | Sodium Dehydrocholate | 8.77E+06 | 1.51E+07 | 6.22E+06 | 2.22E+07 | 1.77E+07 | 4.61E+06 | 2.05E+07 | 1.26E+08 | 6.96E+06 | 2.27E+07 | 0.59 |
| Com_4257_pos | Tetrahydrocorticosterone | 1.61E+06 | 2.00E+06 | 1.27E+08 | 1.13E+06 | 1.45E+06 | 7.24E+07 | 1.15E+06 | 6.37E+06 | 1.46E+06 | 1.79E+06 | 0.90 |
| Com_4260_neg | HexCer-AP (t18:0/16:0) | 1.30E+07 | 5.09E+06 | 6.00E+06 | 2.18E+06 | 8.28E+06 | 4.24E+05 | 1.61E+07 | 3.75E+07 | 1.15E+07 | 2.22E+07 | 0.62 |
| Com_4261_pos | 3-pentadecyl-4,5,6,7-tetrahydrobenzo[d]isoxazol-4-one oxime | 1.31E+07 | 2.41E+07 | 1.27E+08 | 7.57E+06 | 3.98E+06 | 4.61E+06 | 4.21E+06 | 3.04E+06 | 3.54E+06 | 1.08E+07 | 0.10 |
| Com_4319_neg | Adenosine 5'-monophosphate | 3.15E+05 | 3.45E+05 | 4.06E+07 | 1.31E+06 | 4.07E+05 | 4.88E+05 | 7.11E+05 | 6.88E+05 | 2.68E+05 | 1.16E+06 | 0.50 |
| Com_433_neg | 2-Hydroxycaproic acid | 2.23E+07 | 3.89E+07 | 1.11E+08 | 1.34E+08 | 2.39E+08 | 4.09E+08 | 5.26E+08 | 7.13E+08 | 1.68E+08 | 1.74E+08 | 0.03 |
| Com_4344_pos | Ala-Gln | 3.04E+07 | 4.20E+07 | 8.70E+07 | 3.90E+07 | 1.86E+07 | 1.02E+08 | 1.88E+07 | 2.45E+07 | 2.07E+07 | 3.77E+07 | 0.70 |
| Com_4352_pos | TQH | 1.48E+07 | 2.28E+07 | 1.23E+08 | 2.25E+07 | 4.51E+07 | 5.11E+06 | 2.19E+07 | 2.29E+07 | 1.41E+07 | 1.45E+07 | 0.10 |
| Com_4385_pos | PC (18:4e/4:0) | 5.16E+06 | 9.47E+06 | 4.82E+06 | 2.59E+06 | 9.72E+07 | 6.38E+07 | 2.93E+07 | 4.22E+07 | 1.99E+06 | 3.01E+06 | 0.67 |
| Com_4387_pos | 3-(propan-2-yl)-octahydropyrrolo[1,2-a]pyrazine-1,4-dione | 6.03E+07 | 1.00E+08 | 7.91E+07 | 8.60E+07 | 6.38E+07 | 6.26E+07 | 9.41E+07 | 7.17E+07 | 7.99E+07 | 4.67E+07 | 0.52 |
| Com_4390_pos | Glycerol 2-phosphate | 8.41E+06 | 4.19E+06 | 1.23E+08 | 1.47E+07 | 6.14E+06 | 1.08E+07 | 1.67E+07 | 9.33E+06 | 1.10E+07 | 1.15E+07 | 0.85 |
| Com_4408_pos | Epitestosterone | 1.09E+08 | 8.18E+07 | 6.40E+07 | 5.42E+07 | 7.95E+07 | 1.01E+08 | 3.22E+07 | 1.49E+07 | 9.20E+07 | 8.09E+07 | 0.37 |
| Com_4425_pos | 11-Deoxy prostaglandin F1α | 4.08E+07 | 2.69E+07 | 1.21E+08 | 1.61E+07 | 8.95E+06 | 1.05E+07 | 2.89E+07 | 6.03E+06 | 4.68E+07 | 6.30E+07 | 0.70 |
| Com_4433_neg | FAHFA (20:4/20:3) | 8.18E+05 | 4.50E+06 | 2.07E+06 | 1.79E+07 | 1.15E+07 | 1.89E+06 | 8.96E+06 | 3.55E+07 | 7.49E+06 | 3.53E+06 | 0.55 |
| Com_4442_neg | Milbemectin A3 | 2.83E+07 | 4.77E+06 | 4.70E+06 | 3.84E+06 | 6.95E+06 | 1.67E+06 | 1.64E+07 | 2.42E+06 | 3.17E+06 | 1.78E+06 | 0.21 |
| Com_4445_pos | N-Acetyl-L-glutamine | 4.68E+07 | 5.51E+07 | 9.11E+07 | 5.29E+07 | 4.10E+07 | 7.84E+07 | 7.00E+07 | 8.25E+07 | 8.78E+07 | 1.16E+08 | 0.03 |
| Com_4469_neg | 20-Hydroxy-(5Z,8Z,11Z,14Z)-eicosatetraenoic acid | 2.81E+07 | 9.11E+06 | 1.05E+07 | 1.34E+07 | 1.06E+07 | 1.32E+07 | 1.09E+07 | 6.90E+06 | 1.24E+07 | 2.36E+07 | 0.85 |
| Com_4473_pos | LPE 22:5 | 4.69E+06 | 1.44E+07 | 1.82E+06 | 2.68E+06 | 9.49E+07 | 2.04E+07 | 3.26E+07 | 9.69E+06 | 1.19E+06 | 1.45E+06 | 0.84 |
| Com_4475_pos | L-Fucose | 6.96E+07 | 8.95E+07 | 6.42E+07 | 4.92E+07 | 3.76E+07 | 6.35E+07 | 9.20E+07 | 5.27E+07 | 7.41E+07 | 1.15E+08 | 0.25 |
| Com_4489_pos | Bilirubin | 2.97E+06 | 5.64E+05 | 7.40E+06 | 3.53E+06 | 1.11E+06 | 9.83E+07 | 1.46E+07 | 4.37E+06 | 2.87E+06 | 5.54E+06 | 0.09 |
| Com_448_pos | Histamine | 1.34E+07 | 9.71E+07 | 6.52E+08 | 1.65E+09 | 2.02E+08 | 1.23E+08 | 3.03E+08 | 3.43E+08 | 2.32E+08 | 2.38E+08 | 0.84 |
| Com_4497_pos | 4-(5H-thieno[3',2':5,6]thiino[4,3-d]pyrimidin-2-yl)benzamide | 5.60E+07 | 8.58E+07 | 1.60E+07 | 6.10E+07 | 4.03E+07 | 9.80E+07 | 4.68E+07 | 3.45E+07 | 5.89E+07 | 3.30E+07 | 0.79 |
| Com_44_neg | Octadeca-11E,13E,15Z-trienoic acid | 4.50E+06 | 7.36E+06 | 4.59E+06 | 5.19E+06 | 6.59E+06 | 4.01E+06 | 4.77E+06 | 9.59E+09 | 5.25E+06 | 5.71E+09 | 0.20 |
| Com_44_pos | Creatine | 2.44E+09 | 2.48E+09 | 1.73E+10 | 1.85E+09 | 2.24E+09 | 2.91E+09 | 3.15E+09 | 6.49E+09 | 3.25E+09 | 2.29E+09 | 0.99 |
| Com_4502_neg | dCMP | 9.83E+06 | 1.37E+07 | 1.41E+07 | 3.08E+07 | 1.18E+07 | 1.96E+07 | 2.62E+07 | 2.53E+07 | 6.58E+06 | 2.25E+07 | 0.54 |
| Com_4503_neg | LPS 14:0 | 7.31E+06 | 1.28E+07 | 9.44E+05 | 3.08E+07 | 1.95E+07 | 3.03E+06 | 2.29E+06 | 2.31E+07 | 1.28E+06 | 1.02E+07 | 0.44 |
| Com_4504_pos | Debromohymenialdisine | 4.15E+06 | 1.06E+07 | 3.81E+06 | 1.49E+07 | 4.51E+07 | 3.76E+07 | 6.27E+07 | 1.17E+08 | 2.82E+07 | 9.24E+07 | 0.01 |
| Com_4509_pos | 2-Thio-acetyl MAGE | 4.89E+07 | 3.46E+07 | 6.51E+07 | 2.59E+07 | 8.73E+06 | 1.00E+07 | 6.22E+07 | 2.21E+06 | 9.54E+07 | 1.14E+08 | 0.91 |
| Com_450_neg | 13(S)-HOTrE | 4.31E+08 | 2.61E+08 | 4.98E+08 | 2.68E+08 | 3.43E+08 | 2.03E+08 | 6.62E+08 | 4.13E+08 | 3.89E+08 | 2.15E+08 | 0.94 |
| Com_4510_neg | Lactobionic acid | 5.74E+06 | 6.51E+06 | 1.35E+07 | 8.51E+06 | 1.14E+07 | 2.13E+07 | 2.18E+07 | 2.06E+07 | 5.90E+06 | 1.19E+07 | 0.13 |
| Com_4522_pos | Acetylcysteine | 2.45E+06 | 2.65E+06 | 5.51E+06 | 4.87E+07 | 2.73E+06 | 9.74E+07 | 3.61E+06 | 2.58E+07 | 4.83E+06 | 1.18E+07 | 0.29 |
| Com_452_neg | Syringic acid | 2.84E+06 | 1.64E+07 | 2.16E+07 | 7.52E+07 | 2.24E+07 | 1.11E+08 | 9.20E+08 | 2.46E+08 | 4.05E+07 | 1.39E+08 | 0.02 |
| Com_4550_pos | Val-Ser | 3.47E+07 | 9.58E+07 | 3.53E+06 | 6.34E+07 | 4.85E+07 | 7.71E+07 | 1.19E+07 | 7.81E+07 | 6.77E+06 | 5.42E+07 | 0.94 |
| Com_4556_pos | methyl isoquinoline-3-carboxylate | 1.34E+07 | 3.50E+07 | 1.88E+07 | 1.06E+08 | 2.81E+07 | 3.69E+07 | 3.11E+07 | 7.67E+07 | 2.03E+07 | 7.97E+07 | 0.47 |
| Com_4582_neg | 8-iso-15-keto Prostaglandin E2 | 1.51E+07 | 1.98E+07 | 1.06E+06 | 1.44E+07 | 1.11E+07 | 6.61E+06 | 1.03E+07 | 9.87E+05 | 1.15E+07 | 2.74E+07 | 0.83 |
| Com_4595_neg | Imidazoleacetic acid | 9.90E+06 | 1.75E+07 | 1.46E+07 | 9.21E+06 | 1.67E+07 | 1.21E+07 | 1.61E+07 | 1.14E+07 | 8.60E+06 | 3.36E+07 | 0.72 |
| Com_45_neg | Eicosapentaenoic acid | 3.35E+09 | 3.10E+09 | 2.12E+09 | 4.18E+09 | 6.39E+09 | 1.65E+09 | 5.93E+09 | 9.58E+09 | 2.60E+09 | 3.01E+09 | 0.91 |
| Com_4619_neg | Feruloyl Putrescine | 4.09E+06 | 9.05E+06 | 1.17E+06 | 9.12E+06 | 1.35E+07 | 2.06E+07 | 4.47E+06 | 3.11E+07 | 1.71E+06 | 1.41E+07 | 0.48 |
| Com_4626_neg | PC (2:0/13:0) | 2.02E+07 | 1.16E+07 | 2.19E+06 | 1.94E+07 | 2.79E+07 | 9.51E+06 | 1.49E+06 | 1.29E+07 | 9.15E+06 | 7.65E+05 | 0.18 |
| Com_4657_pos | Ritalinic acid | 2.15E+07 | 2.84E+07 | 3.49E+07 | 3.78E+06 | 3.35E+07 | 9.39E+07 | 6.66E+06 | 8.54E+06 | 8.89E+06 | 6.11E+06 | 0.52 |
| Com_465_pos | Glycerophospho-N-palmitoyl ethanolamine | 1.51E+07 | 5.68E+08 | 2.74E+07 | 5.80E+07 | 4.30E+08 | 1.95E+07 | 8.20E+07 | 1.67E+09 | 8.29E+06 | 4.69E+07 | 0.77 |
| Com_4695_neg | LPS 18:0 | 3.32E+06 | 1.23E+07 | 2.59E+06 | 2.01E+07 | 2.73E+07 | 8.06E+06 | 9.08E+06 | 2.74E+07 | 8.55E+05 | 1.17E+07 | 0.78 |
| Com_4698_pos | Noroxymorphone | 4.90E+06 | 6.03E+06 | 1.80E+06 | 2.82E+07 | 4.74E+06 | 9.30E+07 | 3.58E+06 | 4.65E+07 | 1.67E+06 | 9.45E+06 | 0.45 |
| Com_4715_neg | 3-O-Feruloylquinic acid | 4.23E+06 | 4.66E+06 | 5.25E+06 | 9.35E+06 | 2.34E+07 | 9.50E+06 | 2.84E+07 | 3.25E+07 | 2.81E+06 | 6.32E+06 | 0.51 |
| Com_471_neg | trans-2-Butene-1,4-dicarboxylic Acid | 1.09E+08 | 1.35E+08 | 2.13E+08 | 9.19E+07 | 5.38E+08 | 9.35E+07 | 2.15E+08 | 1.63E+08 | 1.67E+08 | 1.20E+08 | 0.64 |
| Com_472_neg | LPC 18:1 | 7.38E+06 | 1.71E+08 | 5.23E+06 | 9.12E+06 | 1.66E+08 | 6.44E+07 | 1.09E+07 | 6.44E+08 | 7.14E+06 | 5.51E+06 | 0.93 |
| Com_4733_pos | 4-(beta-D-Glucopyranosyloxy)-2-methylenebutanoic acid | 2.77E+07 | 9.12E+07 | 1.01E+07 | 1.32E+07 | 2.26E+07 | 7.87E+06 | 2.63E+07 | 3.54E+07 | 2.18E+07 | 2.92E+07 | 0.84 |
| Com_4737_neg | Nonadecanoic acid | 1.20E+07 | 1.88E+07 | 2.56E+07 | 1.67E+07 | 2.01E+07 | 3.57E+06 | 1.58E+07 | 2.47E+07 | 1.38E+07 | 1.08E+07 | 0.25 |
| Com_4745_pos | Lysope 16:0 | 1.73E+06 | 5.05E+07 | 2.82E+06 | 3.46E+06 | 5.26E+07 | 5.93E+06 | 1.74E+07 | 1.10E+08 | 1.88E+06 | 4.26E+06 | 0.89 |
| Com_4758_pos | 2-(2-hydroxy-3-methylbutanamido)-4-methylpentanoic acid | 6.85E+07 | 9.06E+07 | 7.45E+07 | 6.14E+07 | 7.32E+07 | 6.20E+07 | 5.96E+07 | 2.73E+07 | 6.98E+07 | 5.74E+07 | 0.13 |
| Com_475_neg | Phloretin | 8.21E+06 | 6.98E+06 | 8.84E+06 | 2.33E+06 | 1.76E+07 | 3.93E+08 | 1.08E+07 | 7.17E+07 | 3.17E+05 | 7.86E+06 | 0.59 |
| Com_4774_neg | Protocatechuic acid | 5.27E+06 | 3.43E+06 | 6.92E+06 | 6.22E+06 | 2.67E+07 | 5.51E+06 | 6.61E+06 | 3.16E+06 | 3.99E+06 | 4.68E+06 | 0.27 |
| Com_4807_pos | N'-[6-(tert-butyl)thieno[3,2-d]pyrimidin-4-yl]-4-methylbenzohydrazide | 8.88E+07 | 3.79E+07 | 2.00E+06 | 5.99E+07 | 3.71E+07 | 6.16E+07 | 1.35E+07 | 1.14E+07 | 3.97E+07 | 1.05E+08 | 0.81 |
| Com_4808_neg | Prostaglandin F1β | 1.59E+07 | 1.60E+07 | 2.48E+07 | 1.22E+07 | 1.16E+07 | 1.62E+07 | 1.85E+07 | 6.98E+06 | 1.80E+07 | 1.58E+07 | 0.73 |
| Com_4823_pos | 4-methyl-6-phenyl-5,6-dihydro-2H-pyran-2-one | 4.77E+07 | 4.65E+07 | 1.09E+08 | 1.50E+07 | 1.31E+07 | 2.26E+07 | 1.95E+07 | 2.89E+07 | 3.41E+07 | 2.29E+07 | 0.48 |
| Com_4832_pos | P-Aminohippuric Acid | 2.43E+07 | 3.27E+07 | 1.06E+08 | 1.73E+07 | 8.63E+07 | 3.70E+07 | 2.44E+07 | 9.46E+07 | 1.71E+07 | 3.54E+07 | 0.71 |
| Com_4836_pos | Eicosapentaenoic acid ethyl ester | 3.95E+07 | 5.79E+07 | 1.09E+08 | 2.89E+07 | 3.96E+07 | 1.64E+07 | 2.66E+07 | 1.98E+07 | 3.84E+07 | 4.93E+07 | 0.10 |
| Com_483_neg | 1,5-Anhydro-D-glucitol | 2.36E+08 | 3.56E+08 | 6.94E+08 | 4.03E+08 | 4.38E+08 | 3.80E+08 | 7.85E+08 | 4.13E+08 | 2.42E+08 | 5.73E+08 | 0.71 |
| Com_4846_neg | LPA 22:6 | 7.93E+06 | 1.43E+07 | 2.24E+06 | 3.70E+06 | 2.41E+07 | 1.53E+07 | 2.87E+07 | 3.11E+07 | 7.96E+05 | 8.34E+05 | 0.85 |
| Com_4849_pos | Ofloxacin impurity E | 3.02E+06 | 5.57E+06 | 2.84E+06 | 3.49E+07 | 7.99E+06 | 6.28E+07 | 8.55E+07 | 2.07E+07 | 9.22E+07 | 8.47E+07 | 0.00 |
| Com_4852_neg | 1-acetyl-N-(6-chloro-1,3-benzothiazol-2-yl)-4-piperidinecarboxamide | 4.19E+06 | 3.33E+06 | 2.61E+07 | 9.49E+06 | 2.61E+06 | 1.91E+07 | 1.19E+07 | 1.57E+07 | 3.70E+06 | 8.66E+06 | 0.35 |
| Com_4854_neg | LPG 4:0 | 1.76E+06 | 3.74E+05 | 8.19E+05 | 1.12E+07 | 8.43E+05 | 8.00E+05 | 4.20E+07 | 5.48E+06 | 5.72E+06 | 3.88E+06 | 0.16 |
| Com_4865_pos | Phenylglyoxylic acid | 4.41E+06 | 9.53E+06 | 3.12E+06 | 4.08E+07 | 9.00E+06 | 5.31E+07 | 1.31E+08 | 7.07E+07 | 7.45E+07 | 6.69E+07 | 0.01 |
| Com_4873_pos | 23-Norcholic acid | 4.75E+07 | 6.36E+07 | 6.06E+07 | 4.25E+07 | 3.77E+07 | 2.86E+07 | 3.55E+07 | 3.68E+07 | 9.15E+07 | 5.85E+07 | 0.75 |
| Com_4874_pos | JNJ-1661010 | 1.81E+07 | 3.19E+07 | 1.50E+07 | 5.36E+07 | 1.48E+07 | 3.75E+07 | 5.19E+07 | 2.98E+07 | 9.15E+07 | 2.44E+07 | 0.12 |
| Com_48_neg | Ethyl chrysanthemumate | 6.82E+09 | 4.52E+09 | 3.37E+09 | 5.42E+09 | 4.85E+09 | 5.78E+09 | 1.53E+09 | 4.96E+08 | 4.31E+09 | 4.47E+09 | 0.21 |
| Com_4901_neg | LPG 5:0 | 2.23E+06 | 1.06E+06 | 1.03E+06 | 1.28E+07 | 2.38E+06 | 1.23E+05 | 4.15E+07 | 1.89E+06 | 9.61E+06 | 4.77E+06 | 0.75 |
| Com_4905_pos | Tropolone | 6.98E+06 | 1.04E+07 | 1.06E+08 | 6.85E+06 | 1.92E+07 | 6.18E+06 | 7.20E+06 | 7.26E+06 | 5.75E+06 | 6.63E+06 | 0.16 |
| Com_4908_pos | Methyl palmitate | 9.43E+07 | 7.40E+07 | 9.30E+07 | 9.28E+07 | 8.12E+07 | 7.49E+07 | 1.07E+08 | 9.67E+07 | 7.35E+07 | 1.02E+08 | 0.72 |
| Com_4916_neg | Flavin adenine dinucleotide (FAD) | 1.64E+06 | 5.45E+06 | 9.05E+06 | 2.71E+07 | 7.64E+06 | 2.33E+05 | 4.63E+05 | 3.42E+05 | 1.92E+06 | 7.24E+06 | 0.03 |
| Com_4923_neg | 3-Indoxyl sulphate | 1.35E+05 | 1.51E+06 | 7.35E+05 | 2.57E+06 | 5.03E+06 | 1.07E+06 | 4.70E+05 | 3.05E+07 | 5.58E+05 | 2.92E+05 | 0.96 |
| Com_4924_pos | N-(5-acetamidopentyl)acetamide | 6.08E+06 | 1.29E+07 | 6.07E+07 | 5.42E+07 | 6.65E+07 | 4.96E+07 | 1.28E+08 | 2.02E+07 | 6.48E+06 | 1.52E+07 | 0.93 |
| Com_4926_pos | Thiamine monophosphate | 1.10E+07 | 5.09E+06 | 1.06E+08 | 5.33E+07 | 8.34E+07 | 7.86E+06 | 4.81E+07 | 2.85E+06 | 3.56E+07 | 3.40E+07 | 0.48 |
| Com_492_neg | Maltotriose | 2.39E+07 | 7.12E+07 | 2.59E+07 | 9.55E+06 | 5.17E+08 | 7.34E+07 | 1.26E+08 | 2.41E+08 | 1.07E+07 | 1.61E+07 | 0.90 |
| Com_4945_neg | Adrenosterone | 4.03E+05 | 2.69E+06 | 1.81E+06 | 2.54E+07 | 4.52E+06 | 5.99E+06 | 7.61E+06 | 2.37E+07 | 1.73E+07 | 1.00E+07 | 0.12 |
| Com_4947_neg | (R)-3-Hydroxy myristic acid | 7.16E+06 | 8.39E+06 | 2.18E+07 | 1.29E+07 | 6.65E+06 | 7.20E+06 | 9.64E+06 | 3.03E+07 | 8.69E+06 | 1.42E+07 | 0.63 |
| Com_4952_pos | 1-(4-hydroxyphenyl)propane-1,2-diol | 4.25E+07 | 1.90E+07 | 1.05E+08 | 4.16E+07 | 3.04E+07 | 6.57E+07 | 5.19E+07 | 6.02E+06 | 4.09E+07 | 4.88E+07 | 0.72 |
| Com_4965_pos | 3-Hydroxysebacic acid | 8.03E+07 | 8.50E+07 | 7.46E+07 | 3.75E+07 | 4.75E+07 | 5.61E+07 | 5.68E+07 | 3.62E+07 | 5.71E+07 | 4.97E+07 | 0.31 |
| Com_5035_pos | Skatole | 2.84E+07 | 3.66E+07 | 2.29E+07 | 5.31E+07 | 5.82E+07 | 6.79E+07 | 5.09E+07 | 1.01E+08 | 2.69E+07 | 5.99E+07 | 0.19 |
| Com_5037_neg | 8(S),15(S)-DiHETE | 1.92E+07 | 1.50E+07 | 1.90E+07 | 1.33E+07 | 9.95E+06 | 8.88E+06 | 1.46E+07 | 6.60E+06 | 1.68E+07 | 1.31E+07 | 0.24 |
| Com_5045_neg | Xanthohumol | 1.07E+07 | 1.71E+07 | 6.13E+05 | 1.41E+07 | 1.44E+07 | 5.85E+06 | 1.61E+07 | 8.63E+06 | 5.93E+06 | 1.63E+07 | 0.73 |
| Com_5050_pos | Mag (18:1) | 3.75E+07 | 5.66E+07 | 1.92E+07 | 9.22E+07 | 1.85E+07 | 1.46E+07 | 2.73E+07 | 1.28E+07 | 3.76E+07 | 4.71E+07 | 0.34 |
| Com_506_pos | L-Threonic acid-1,4-lactone | 1.12E+08 | 2.14E+08 | 1.75E+08 | 1.86E+08 | 3.31E+08 | 6.79E+08 | 4.10E+08 | 1.51E+09 | 1.67E+08 | 2.69E+08 | 0.09 |
| Com_509_neg | LPE 20:3 | 3.36E+07 | 3.49E+08 | 8.50E+06 | 1.24E+07 | 3.15E+08 | 2.53E+08 | 8.52E+07 | 7.69E+07 | 1.89E+07 | 2.63E+06 | 0.79 |
| Com_50_neg | Deoxycholic acid | 7.53E+09 | 3.13E+09 | 3.68E+09 | 1.83E+09 | 2.27E+09 | 2.16E+09 | 1.60E+09 | 1.67E+09 | 3.32E+09 | 2.63E+09 | 0.21 |
| Com_5104_neg | 1-Methylguanine | 1.22E+06 | 2.33E+06 | 1.55E+06 | 2.81E+06 | 2.61E+06 | 1.77E+07 | 9.52E+06 | 7.81E+06 | 1.42E+06 | 5.65E+06 | 0.05 |
| Com_511_pos | N-[1-(4-methoxy-2-oxo-2H-pyran-6-yl)-2-methylbutyl]acetamide | 9.82E+08 | 1.24E+09 | 8.10E+06 | 1.29E+08 | 8.16E+07 | 4.86E+07 | 2.01E+06 | 2.58E+06 | 4.98E+07 | 3.28E+08 | 0.17 |
| Com_5129_pos | L-beta-Imidazolelactic acid | 1.28E+07 | 1.82E+07 | 4.60E+07 | 5.87E+07 | 1.78E+07 | 8.22E+07 | 3.83E+07 | 4.34E+07 | 3.63E+07 | 5.98E+07 | 0.10 |
| Com_5133_pos | Lysopc 17:0 | 2.29E+06 | 8.05E+06 | 2.49E+06 | 3.29E+06 | 1.72E+07 | 4.07E+06 | 5.01E+06 | 9.83E+07 | 2.09E+06 | 2.93E+06 | 0.71 |
| Com_513_neg | 7-Hydroxy-3,4-dihydrocarbostyril | 2.94E+08 | 3.42E+08 | 3.41E+08 | 1.58E+08 | 4.55E+08 | 2.85E+08 | 4.06E+08 | 1.82E+08 | 3.07E+08 | 1.50E+07 | 0.35 |
| Com_5145_neg | 4-Hydroxy-L-Glutamic Acid | 2.18E+06 | 1.98E+06 | 4.28E+06 | 2.53E+07 | 1.81E+06 | 2.42E+06 | 7.57E+06 | 5.73E+06 | 1.02E+07 | 8.02E+06 | 0.44 |
| Com_5153_neg | Leukotriene C4 | 3.57E+06 | 2.97E+06 | 3.49E+05 | 4.80E+06 | 9.83E+06 | 3.68E+06 | 4.01E+05 | 2.85E+07 | 1.98E+05 | 3.14E+05 | 0.47 |
| Com_5160_neg | Shikimic acid | 1.10E+07 | 1.34E+07 | 2.07E+07 | 1.02E+07 | 2.00E+07 | 9.44E+06 | 1.58E+07 | 1.09E+07 | 1.45E+07 | 7.80E+06 | 0.25 |
| Com_5160_pos | 11-Oxoetiocholanolone | 3.55E+07 | 2.45E+07 | 1.22E+07 | 1.22E+07 | 3.19E+07 | 8.15E+07 | 2.63E+07 | 3.09E+07 | 4.83E+07 | 4.46E+07 | 0.05 |
| Com_5161_pos | 7-Methylguanosine | 1.02E+07 | 1.66E+07 | 1.80E+07 | 8.96E+07 | 1.60E+07 | 1.20E+07 | 2.20E+07 | 4.33E+07 | 1.93E+07 | 5.53E+07 | 0.67 |
| Com_5162_pos | 15-Deoxy-Δ12,14-prostaglandin J2-2-glycerol ester | 1.35E+07 | 8.64E+06 | 1.13E+07 | 8.54E+06 | 1.28E+06 | 4.40E+06 | 4.94E+06 | 5.96E+05 | 8.40E+07 | 2.00E+07 | 0.93 |
| Com_516_pos | 1-[(3,5-dimethylisoxazol-4-yl)sulfonyl]piperidine | 9.60E+06 | 2.23E+06 | 1.09E+06 | 1.54E+08 | 3.67E+07 | 1.24E+09 | 5.98E+07 | 3.57E+08 | 1.92E+06 | 4.44E+07 | 0.21 |
| Com_5182_pos | Dehydroepiandrosterone (DHEA) | 3.48E+07 | 3.39E+07 | 1.71E+07 | 8.92E+07 | 5.95E+07 | 3.43E+07 | 6.05E+07 | 4.24E+07 | 4.77E+07 | 3.49E+07 | 0.84 |
| Com_5184_pos | 1-(3-acetyl-2,4,6-trihydroxyphenyl)ethan-1-one | 6.80E+06 | 1.20E+07 | 5.42E+06 | 4.11E+07 | 8.81E+06 | 4.89E+07 | 1.13E+08 | 6.20E+07 | 8.36E+07 | 7.42E+07 | 0.00 |
| Com_5187_pos | 4-(3-methoxyphenyl)-4-methyl-2,6-dioxopiperidine-3,5-dicarbonitrile | 7.53E+06 | 6.83E+06 | 1.81E+06 | 4.32E+06 | 7.79E+07 | 3.70E+06 | 1.28E+07 | 2.64E+07 | 1.46E+06 | 1.60E+06 | 0.59 |
| Com_5194_pos | 2-[(3S)-1-(2-Methylbenzyl)-3-pyrrolidinyl]-1H-imidazo[4,5-b]pyridine | 1.24E+07 | 8.00E+07 | 2.33E+06 | 2.05E+06 | 3.56E+07 | 7.79E+06 | 2.58E+06 | 1.33E+07 | 1.29E+06 | 4.37E+06 | 0.30 |
| Com_5195_pos | PC (18:5e/3:0) | 3.47E+06 | 3.03E+06 | 3.60E+06 | 6.50E+06 | 7.78E+07 | 1.34E+07 | 3.72E+06 | 1.45E+07 | 3.09E+06 | 2.96E+06 | 0.77 |
| Com_519_neg | N-Acetylmannosamine | 2.17E+08 | 2.72E+08 | 2.86E+08 | 3.75E+08 | 2.93E+08 | 2.14E+08 | 2.34E+08 | 2.35E+08 | 1.19E+08 | 5.69E+08 | 0.55 |
| Com_5205_pos | Androstenedione | 7.61E+07 | 7.16E+07 | 6.66E+07 | 5.11E+07 | 4.09E+07 | 7.08E+07 | 4.40E+07 | 4.10E+07 | 8.31E+07 | 8.36E+07 | 0.88 |
| Com_5211_neg | Gibberellic acid | 2.25E+07 | 4.55E+05 | 5.10E+05 | 1.25E+06 | 3.16E+05 | 7.66E+05 | 2.84E+06 | 8.62E+05 | 1.21E+06 | 1.08E+06 | 0.97 |
| Com_5212_pos | 2-Amino-1,3-octadecanediol | 2.26E+07 | 7.96E+07 | 4.25E+07 | 2.87E+07 | 6.91E+07 | 7.71E+07 | 3.04E+07 | 3.88E+07 | 1.22E+07 | 3.73E+07 | 0.52 |
| Com_5218_pos | RNH | 9.29E+06 | 1.11E+07 | 3.57E+07 | 3.14E+07 | 1.05E+07 | 1.02E+07 | 1.61E+07 | 7.16E+06 | 8.28E+07 | 2.11E+07 | 0.84 |
| Com_5226_neg | Monobutyl phthalate | 4.06E+06 | 4.67E+06 | 2.10E+06 | 9.53E+05 | 1.70E+06 | 1.46E+07 | 1.77E+06 | 2.78E+07 | 6.43E+05 | 9.91E+05 | 0.65 |
| Com_522_neg | Citrulline | 1.13E+08 | 1.45E+08 | 3.13E+08 | 4.64E+08 | 2.25E+08 | 1.65E+08 | 4.46E+08 | 2.12E+08 | 1.70E+08 | 5.63E+08 | 0.59 |
| Com_523_pos | Thiamine | 8.90E+08 | 5.29E+08 | 1.16E+09 | 3.87E+08 | 8.99E+08 | 4.18E+08 | 6.94E+08 | 8.77E+08 | 1.24E+09 | 6.88E+08 | 0.93 |
| Com_5245_neg | FAHFA (4:0/24:1) | 4.51E+06 | 5.86E+06 | 9.71E+06 | 1.21E+07 | 6.42E+06 | 1.70E+07 | 8.04E+06 | 5.54E+06 | 7.35E+06 | 6.34E+06 | 0.68 |
| Com_5247_pos | 2-(3,4-dihydroxyphenyl)acetamide | 6.23E+07 | 7.88E+07 | 4.29E+07 | 2.73E+07 | 6.89E+07 | 3.50E+07 | 4.96E+07 | 3.86E+07 | 5.48E+07 | 3.73E+07 | 0.36 |
| Com_527_pos | N-Acetylputrescine | 4.85E+07 | 5.95E+07 | 1.44E+09 | 9.20E+07 | 4.14E+08 | 2.21E+08 | 4.97E+08 | 4.12E+08 | 2.33E+07 | 4.71E+07 | 0.80 |
| Com_528_neg | 5-oxoproline | 1.33E+08 | 1.43E+08 | 6.18E+08 | 4.02E+08 | 1.51E+08 | 1.56E+08 | 2.79E+08 | 1.74E+08 | 3.12E+08 | 4.69E+08 | 0.82 |
| Com_5296_pos | 1,4-dihydroxyheptadec-16-en-2-yl acetate | 1.05E+07 | 7.79E+07 | 1.03E+07 | 6.90E+06 | 1.28E+07 | 7.38E+06 | 9.11E+06 | 6.16E+07 | 7.37E+06 | 8.76E+06 | 0.74 |
| Com_5297_neg | 1-Methyluric acid | 8.08E+05 | 1.31E+06 | 2.22E+06 | 1.63E+06 | 5.64E+06 | 1.29E+07 | 4.85E+06 | 2.72E+07 | 2.57E+06 | 3.84E+06 | 0.04 |
| Com_5303_pos | Estazolam | 8.31E+06 | 2.50E+07 | 2.06E+06 | 7.62E+06 | 9.92E+06 | 7.86E+07 | 2.55E+07 | 2.23E+07 | 7.04E+06 | 2.09E+06 | 0.44 |
| Com_5305_pos | 4-ethoxy-6,7-dimethoxyquinazoline | 3.01E+07 | 5.55E+07 | 3.96E+07 | 3.88E+07 | 7.56E+07 | 3.21E+07 | 6.30E+07 | 5.64E+07 | 3.56E+07 | 3.03E+07 | 0.69 |
| Com_5314_pos | PC (14:0e/3:0) | 7.06E+06 | 7.76E+07 | 9.81E+06 | 6.06E+06 | 2.77E+07 | 5.86E+06 | 1.33E+06 | 8.80E+07 | 5.24E+06 | 6.10E+06 | 0.40 |
| Com_5321_pos | (11E,15Z)-9,10,13-trihydroxyoctadeca-11,15-dienoic acid | 3.00E+07 | 5.83E+07 | 4.66E+07 | 3.88E+07 | 2.35E+07 | 7.83E+07 | 4.87E+07 | 2.07E+07 | 7.46E+07 | 6.77E+07 | 0.29 |
| Com_5326_neg | Hydroxyglutaric acid | 1.01E+06 | 7.47E+05 | 1.40E+06 | 1.11E+06 | 1.80E+06 | 1.66E+07 | 1.98E+06 | 1.42E+06 | 6.43E+05 | 2.02E+06 | 0.29 |
| Com_5334_pos | (+/-)-Cannabichromeorcin | 2.43E+07 | 4.30E+07 | 3.87E+07 | 5.74E+07 | 6.78E+06 | 3.43E+07 | 2.77E+07 | 1.23E+07 | 5.03E+07 | 9.11E+07 | 0.64 |
| Com_5349_pos | Piperine | 5.04E+06 | 1.50E+07 | 4.41E+07 | 8.37E+06 | 3.96E+07 | 4.61E+07 | 9.92E+07 | 9.31E+07 | 2.66E+07 | 1.07E+07 | 0.15 |
| Com_5367_pos | dopaquinone | 1.54E+07 | 2.11E+07 | 1.62E+07 | 1.49E+07 | 2.41E+07 | 7.75E+07 | 1.96E+07 | 2.93E+07 | 1.47E+07 | 1.99E+07 | 0.26 |
| Com_536_neg | 4-((5-(4-Nitrophenyl)oxazol-2-yl)amino)benzonitrile | 4.78E+06 | 1.21E+08 | 5.58E+06 | 7.89E+05 | 2.72E+07 | 2.55E+08 | 1.09E+08 | 5.58E+08 | 3.01E+06 | 8.01E+05 | 0.44 |
| Com_5371_pos | 3-Succinoylpyridine | 6.17E+06 | 1.21E+07 | 9.40E+07 | 7.64E+06 | 2.40E+07 | 1.83E+07 | 2.70E+07 | 2.02E+07 | 6.86E+06 | 5.62E+06 | 0.69 |
| Com_5384_pos | 1,3-bis(4-methoxybenzyl)-2-phenylhexahydropyrimidine | 3.68E+07 | 7.64E+07 | 3.31E+07 | 1.86E+07 | 2.15E+07 | 4.18E+06 | 1.86E+07 | 1.90E+07 | 3.31E+07 | 2.12E+07 | 0.14 |
| Com_5389_pos | 13-OxoODE | 7.33E+07 | 5.05E+07 | 3.04E+07 | 4.15E+07 | 1.91E+07 | 6.08E+07 | 9.60E+07 | 8.58E+06 | 6.02E+07 | 3.87E+07 | 0.91 |
| Com_5398_neg | L-Cysteinesulfinic acid | 1.67E+06 | 5.20E+06 | 2.14E+06 | 4.16E+06 | 1.43E+07 | 1.54E+07 | 2.94E+06 | 2.65E+07 | 2.69E+06 | 3.96E+06 | 0.44 |
| Com_539_neg | gamma-Nonanolactone | 3.28E+08 | 2.82E+08 | 3.19E+08 | 1.27E+08 | 1.34E+08 | 8.05E+07 | 2.49E+08 | 1.02E+08 | 3.17E+08 | 2.09E+08 | 0.46 |
| Com_53_neg | Lauric acid ethyl ester | 2.99E+09 | 3.66E+09 | 6.72E+09 | 6.80E+09 | 3.06E+09 | 1.45E+09 | 9.75E+09 | 2.97E+09 | 5.17E+09 | 5.85E+09 | 0.92 |
| Com_53_pos | 3-(3,4-dihydroxyphenyl)propanoic acid | 1.81E+09 | 3.65E+09 | 2.83E+09 | 4.77E+09 | 5.45E+09 | 6.44E+09 | 4.30E+09 | 1.51E+10 | 2.55E+09 | 7.46E+09 | 0.16 |
| Com_5400_neg | LPG 18:3 | 2.11E+06 | 1.55E+07 | 7.47E+05 | 5.84E+05 | 6.63E+06 | 1.35E+06 | 7.88E+05 | 1.49E+07 | 4.47E+05 | 2.39E+06 | 0.70 |
| Com_5403_pos | Malvidin | 1.01E+07 | 1.49E+07 | 5.91E+06 | 2.81E+07 | 1.84E+07 | 7.68E+07 | 1.11E+08 | 7.15E+07 | 2.61E+07 | 2.62E+07 | 0.01 |
| Com_5435_pos | Andrographolide | 6.81E+07 | 7.55E+07 | 4.72E+07 | 3.10E+07 | 3.27E+07 | 1.92E+07 | 5.67E+07 | 2.93E+07 | 7.18E+07 | 3.64E+07 | 0.49 |
| Com_5439_pos | 2-Linoleoyl glycerol | 1.18E+07 | 2.51E+07 | 2.31E+07 | 6.91E+07 | 3.23E+07 | 1.62E+07 | 3.21E+07 | 9.14E+07 | 5.42E+07 | 2.21E+07 | 0.55 |
| Com_5440_pos | C-8 Ceramide-1-phosphate | 1.08E+07 | 1.88E+07 | 5.12E+07 | 1.16E+07 | 3.42E+07 | 1.96E+07 | 7.94E+06 | 9.13E+07 | 1.08E+07 | 1.80E+07 | 0.88 |
| Com_5457_pos | D-Panthenol | 9.68E+07 | 8.14E+06 | 3.88E+06 | 2.27E+06 | 5.66E+06 | 2.13E+06 | 2.83E+06 | 1.64E+06 | 7.58E+06 | 3.19E+06 | 0.20 |
| Com_545_pos | 3-amino-2-phenyl-2H-pyrazolo[4,3-c]pyridine-4,6-diol | 7.88E+08 | 2.74E+08 | 1.39E+09 | 1.33E+08 | 5.28E+08 | 5.63E+07 | 2.06E+08 | 1.59E+08 | 2.32E+08 | 2.35E+08 | 0.07 |
| Com_5464_pos | N-Acetyl-L-leucine | 3.68E+07 | 7.49E+07 | 3.74E+07 | 2.98E+07 | 4.08E+07 | 1.95E+07 | 3.73E+07 | 2.32E+07 | 2.96E+07 | 4.47E+07 | 0.15 |
| Com_5468_pos | Acetophenone | 4.89E+07 | 3.91E+07 | 8.59E+06 | 4.71E+07 | 6.38E+07 | 4.31E+07 | 6.52E+07 | 2.58E+07 | 7.80E+07 | 4.49E+07 | 0.45 |
| Com_5478_neg | Lactitol | 1.12E+07 | 8.59E+06 | 2.88E+07 | 1.46E+07 | 1.11E+07 | 1.41E+07 | 2.72E+07 | 2.41E+07 | 8.59E+06 | 2.37E+07 | 0.37 |
| Com_5492_neg | 3-Hydroxybutyric acid | 4.56E+06 | 3.38E+06 | 5.66E+06 | 4.02E+06 | 7.17E+06 | 3.31E+06 | 8.01E+06 | 2.61E+07 | 2.70E+06 | 4.27E+06 | 0.61 |
| Com_54_neg | Docosapentaenoic acid | 8.88E+08 | 2.35E+09 | 9.86E+09 | 2.19E+09 | 4.80E+09 | 3.13E+08 | 1.94E+09 | 1.45E+09 | 1.00E+09 | 1.03E+09 | 0.07 |
| Com_5506_pos | LPC 16:2 | 1.01E+07 | 1.67E+07 | 7.40E+06 | 7.99E+06 | 6.98E+07 | 7.48E+07 | 6.74E+06 | 1.94E+07 | 8.53E+06 | 8.30E+06 | 1.00 |
| Com_5514_pos | Phenyl[(3S)-3-(5-propyl-1,3,4-oxadiazol-2-yl)-1-pyrrolidinyl]methanone | 6.26E+06 | 8.09E+06 | 2.15E+06 | 7.95E+06 | 7.18E+07 | 1.09E+07 | 9.21E+06 | 2.83E+07 | 1.97E+06 | 3.38E+06 | 0.76 |
| Com_551_pos | Melatonin | 2.50E+07 | 6.02E+07 | 2.24E+06 | 1.23E+09 | 1.65E+07 | 1.34E+08 | 5.64E+07 | 8.71E+06 | 4.03E+08 | 6.76E+06 | 0.89 |
| Com_5525_pos | β-Asarone | 4.44E+07 | 5.75E+07 | 8.95E+07 | 1.37E+07 | 6.64E+07 | 5.15E+07 | 7.46E+07 | 3.94E+07 | 7.68E+07 | 6.55E+07 | 0.49 |
| Com_5544_pos | 6-Pentyl-2H-pyran-2-one | 7.66E+07 | 7.33E+07 | 4.48E+07 | 3.78E+07 | 5.47E+07 | 6.87E+07 | 4.57E+07 | 1.57E+07 | 6.21E+07 | 6.74E+07 | 0.57 |
| Com_5574_pos | LNK | 6.12E+07 | 2.04E+07 | 2.42E+07 | 3.58E+07 | 7.05E+07 | 7.35E+07 | 3.65E+07 | 1.24E+07 | 5.84E+07 | 5.40E+07 | 0.88 |
| Com_5580_neg | MAG (18:2) | 1.73E+07 | 1.10E+07 | 4.33E+06 | 2.26E+07 | 1.00E+07 | 1.91E+06 | 1.97E+07 | 1.61E+06 | 9.73E+06 | 1.73E+07 | 0.37 |
| Com_5617_pos | L-Adrenaline | 3.50E+07 | 1.06E+07 | 5.30E+07 | 4.74E+07 | 6.21E+07 | 4.78E+07 | 9.40E+07 | 5.64E+07 | 7.50E+07 | 7.23E+07 | 0.12 |
| Com_5632_neg | LPA 20:5 | 5.99E+05 | 4.67E+05 | 7.57E+05 | 5.89E+05 | 2.10E+07 | 3.65E+06 | 8.40E+05 | 1.50E+07 | 4.01E+05 | 6.59E+05 | 0.76 |
| Com_5636_pos | N6-Isopentenyladenosine | 1.60E+07 | 1.43E+07 | 1.49E+07 | 7.97E+07 | 4.72E+06 | 1.94E+06 | 1.20E+07 | 4.06E+06 | 7.49E+06 | 6.33E+07 | 0.39 |
| Com_5640_pos | 12,13-EODE | 5.16E+07 | 4.53E+07 | 4.10E+07 | 5.91E+07 | 6.96E+07 | 1.83E+07 | 6.41E+07 | 7.99E+07 | 6.10E+07 | 3.43E+07 | 0.64 |
| Com_564_pos | Oleoyl ethanolamide | 3.71E+08 | 5.97E+08 | 1.33E+09 | 6.92E+08 | 7.05E+08 | 1.81E+08 | 9.94E+08 | 1.27E+09 | 5.40E+08 | 2.79E+08 | 0.52 |
| Com_5658_pos | Cholecalciferol | 4.69E+07 | 3.36E+07 | 5.18E+07 | 6.04E+07 | 2.59E+07 | 3.12E+07 | 2.79E+07 | 3.25E+07 | 7.43E+07 | 4.92E+07 | 0.86 |
| Com_5668_neg | 3,3-Dimethylglutaric acid | 6.92E+06 | 1.14E+07 | 8.20E+06 | 1.07E+07 | 1.02E+07 | 1.53E+07 | 8.94E+06 | 1.14E+07 | 8.87E+06 | 8.94E+06 | 0.45 |
| Com_5685_pos | LysoPC 10:0 | 1.71E+07 | 6.38E+07 | 1.73E+06 | 6.24E+06 | 6.89E+07 | 1.69E+07 | 9.45E+06 | 6.89E+07 | 2.07E+06 | 1.85E+06 | 0.56 |
| Com_5687_neg | LPC 17:1 | 1.16E+06 | 1.44E+07 | 1.50E+06 | 1.71E+06 | 1.11E+07 | 5.37E+06 | 3.04E+06 | 2.07E+06 | 1.73E+06 | 1.15E+06 | 0.54 |
| Com_5687_pos | 4-phenoxyphenyl 4-hydroxypiperidine-1-carboxylate | 4.46E+06 | 8.93E+06 | 2.31E+06 | 2.10E+06 | 6.88E+07 | 7.63E+06 | 5.17E+06 | 1.26E+07 | 1.69E+06 | 2.81E+06 | 0.65 |
| Com_5699_pos | 5-Methyl-2'-deoxycytidine | 4.63E+07 | 5.49E+07 | 6.88E+07 | 3.46E+07 | 6.88E+07 | 2.31E+07 | 4.45E+07 | 4.31E+07 | 3.41E+07 | 5.44E+07 | 0.14 |
| Com_56_pos | Nicotinic acid | 8.22E+09 | 9.15E+09 | 1.51E+10 | 8.65E+09 | 9.87E+09 | 4.67E+09 | 1.09E+10 | 7.34E+09 | 1.08E+10 | 1.07E+10 | 0.44 |
| Com_5708_pos | N-Carbamyl-L-glutamicacid | 1.00E+07 | 6.08E+06 | 3.29E+07 | 1.73E+07 | 1.83E+07 | 5.90E+07 | 5.91E+07 | 8.55E+07 | 7.46E+06 | 1.63E+07 | 0.18 |
| Com_5717_pos | Coenzyme Q2 | 6.54E+07 | 5.27E+07 | 2.39E+07 | 5.98E+07 | 4.97E+07 | 1.90E+07 | 7.17E+07 | 6.10E+07 | 7.34E+07 | 5.91E+07 | 0.81 |
| Com_5747_pos | ELK | 2.15E+07 | 4.46E+07 | 5.91E+06 | 7.76E+07 | 2.34E+07 | 5.70E+07 | 2.67E+07 | 3.58E+07 | 2.71E+07 | 7.25E+07 | 0.36 |
| Com_5757_pos | N-(1-benzyl-4-piperidinyl)-4-(1H-pyrazol-1-yl)benzamide | 2.26E+07 | 4.49E+07 | 2.95E+07 | 7.75E+07 | 2.42E+07 | 6.29E+07 | 7.70E+07 | 2.68E+07 | 4.75E+07 | 3.72E+07 | 0.37 |
| Com_576_neg | alpha-Ketoglutaric acid | 7.87E+07 | 5.36E+07 | 1.78E+08 | 6.99E+07 | 2.91E+07 | 1.37E+08 | 3.60E+08 | 3.51E+08 | 2.88E+08 | 3.32E+08 | 0.01 |
| Com_5773_pos | Dl-3-Hydroxynorvaline | 2.63E+07 | 4.13E+07 | 3.30E+07 | 7.68E+07 | 6.76E+07 | 4.85E+07 | 4.05E+07 | 2.93E+07 | 2.89E+07 | 5.32E+07 | 0.56 |
| Com_5776_pos | Taurocholic acid | 8.95E+07 | 2.79E+07 | 6.67E+07 | 8.36E+06 | 6.45E+06 | 1.19E+07 | 2.84E+07 | 6.07E+06 | 8.18E+06 | 6.23E+06 | 0.19 |
| Com_579_neg | alpha-Farnesene | 1.94E+08 | 1.90E+08 | 9.12E+07 | 1.35E+08 | 2.27E+08 | 6.20E+07 | 1.36E+08 | 2.58E+08 | 2.87E+08 | 1.38E+08 | 0.91 |
| Com_57_pos | L-Tyrosine | 1.74E+09 | 3.49E+09 | 2.72E+09 | 4.59E+09 | 5.25E+09 | 6.14E+09 | 4.13E+09 | 1.45E+10 | 2.47E+09 | 7.19E+09 | 0.16 |
| Com_5808_pos | 5-Methoxytryptamine | 1.35E+06 | 1.06E+07 | 3.74E+05 | 7.66E+07 | 7.31E+05 | 1.25E+06 | 6.49E+06 | 4.69E+05 | 2.13E+07 | 5.71E+05 | 0.77 |
| Com_5823_neg | 2,3-dinor Prostaglandin E1 | 1.29E+07 | 1.40E+07 | 1.64E+07 | 8.19E+06 | 7.11E+06 | 1.19E+07 | 1.00E+07 | 3.33E+06 | 1.36E+07 | 1.00E+07 | 0.46 |
| Com_5825_pos | 4-(pentyloxy)benzene-1-carbohydrazide | 2.49E+07 | 6.86E+07 | 2.69E+06 | 6.64E+06 | 1.31E+07 | 1.72E+07 | 2.73E+06 | 9.84E+06 | 5.62E+06 | 6.98E+06 | 0.37 |
| Com_5832_pos | N-(2-Methoxyphenyl)acetamide | 8.82E+07 | 3.15E+07 | 6.42E+06 | 2.38E+07 | 3.35E+07 | 2.93E+07 | 1.66E+07 | 5.38E+07 | 1.09E+07 | 2.56E+07 | 0.80 |
| Com_5836_pos | Bisphenol TMC | 1.31E+07 | 3.05E+07 | 7.02E+06 | 4.15E+07 | 1.56E+07 | 1.83E+07 | 2.81E+07 | 2.54E+07 | 1.49E+07 | 8.06E+07 | 0.35 |
| Com_583_neg | Y-Aminobutyric acid (GABA) | 2.69E+07 | 4.22E+07 | 6.48E+07 | 4.44E+08 | 4.61E+07 | 8.73E+07 | 1.40E+08 | 1.46E+08 | 1.15E+08 | 1.81E+08 | 0.26 |
| Com_5858_neg | L-threo-3-Phenylserine | 3.33E+06 | 1.39E+07 | 1.84E+06 | 1.46E+06 | 1.40E+07 | 9.41E+06 | 7.81E+06 | 9.55E+06 | 1.19E+06 | 5.08E+06 | 0.78 |
| Com_586_pos | PC (18:3e/2:0) | 4.05E+07 | 1.20E+08 | 9.70E+06 | 2.93E+07 | 9.68E+08 | 1.06E+09 | 1.91E+08 | 4.47E+08 | 3.67E+06 | 6.15E+06 | 0.95 |
| Com_5889_pos | 3-(3-pyridinyl)propanoic acid | 3.38E+07 | 2.77E+07 | 2.69E+07 | 1.77E+07 | 6.53E+07 | 3.89E+07 | 5.52E+07 | 4.33E+07 | 3.40E+07 | 3.36E+07 | 0.31 |
| Com_588_neg | D-(-)-Mannitol | 3.96E+07 | 5.61E+07 | 3.12E+08 | 3.74E+08 | 1.31E+08 | 2.65E+08 | 5.02E+08 | 1.04E+08 | 2.19E+08 | 4.94E+08 | 0.20 |
| Com_58_pos | 5-Aminovaleric acid | 3.85E+09 | 2.59E+09 | 3.07E+09 | 9.48E+09 | 5.10E+09 | 1.21E+10 | 2.92E+09 | 6.08E+09 | 2.09E+09 | 2.88E+09 | 0.95 |
| Com_5902_pos | DQH | 8.91E+05 | 7.14E+05 | 8.91E+05 | 9.84E+05 | 7.44E+05 | 6.77E+07 | 8.24E+05 | 2.50E+06 | 7.56E+05 | 1.47E+06 | 0.23 |
| Com_5903_pos | Dihomo-γ-linolenic acid methyl ester | 4.56E+06 | 6.28E+06 | 3.99E+06 | 3.36E+06 | 6.52E+07 | 5.89E+06 | 6.23E+06 | 8.07E+06 | 2.67E+06 | 3.73E+06 | 0.50 |
| Com_5905_neg | Phosphoethanolamine | 2.01E+06 | 1.97E+06 | 2.59E+07 | 4.54E+06 | 6.07E+06 | 2.93E+06 | 7.21E+06 | 4.60E+06 | 1.77E+06 | 6.09E+06 | 0.72 |
| Com_5918_pos | 2-(2-amino-3-methylbutanamido)-3-phenylpropanoic acid | 1.44E+07 | 2.66E+07 | 1.03E+07 | 5.21E+07 | 2.56E+07 | 6.76E+07 | 1.50E+07 | 4.95E+07 | 1.36E+07 | 4.20E+07 | 0.45 |
| Com_592_pos | Phosphocholine | 1.36E+07 | 1.64E+08 | 5.59E+06 | 2.04E+07 | 1.01E+09 | 4.59E+08 | 3.09E+07 | 4.85E+08 | 1.82E+06 | 7.16E+05 | 0.70 |
| Com_5939_neg | 2-{1-[2-(4-benzhydrylpiperazino)-2-oxoethyl]cyclopentyl}acetic acid | 2.44E+05 | 3.29E+05 | 3.25E+05 | 2.76E+05 | 2.89E+05 | 1.43E+07 | 4.62E+05 | 4.34E+05 | 1.64E+05 | 5.20E+05 | 0.28 |
| Com_5949_pos | LPK | 2.77E+06 | 2.34E+06 | 2.84E+06 | 2.59E+06 | 2.32E+06 | 4.06E+06 | 2.46E+06 | 8.04E+07 | 1.86E+06 | 3.00E+06 | 0.34 |
| Com_594_pos | 2-Oxindole | 1.15E+09 | 1.04E+09 | 7.77E+08 | 9.78E+07 | 6.04E+08 | 7.11E+07 | 1.78E+08 | 1.52E+08 | 2.81E+08 | 3.59E+08 | 0.07 |
| Com_5965_pos | Gly-Tyr | 9.81E+06 | 2.86E+07 | 1.86E+06 | 1.02E+07 | 3.92E+07 | 2.55E+07 | 8.66E+06 | 7.99E+07 | 3.94E+06 | 2.37E+07 | 0.59 |
| Com_5969_neg | (±)8(9)-DiHET | 1.30E+07 | 1.35E+07 | 1.01E+07 | 7.21E+06 | 1.02E+07 | 6.74E+06 | 1.35E+07 | 1.65E+07 | 1.22E+07 | 9.46E+06 | 0.77 |
| Com_5978_pos | L-Ornithine | 2.38E+07 | 2.77E+07 | 4.98E+07 | 7.32E+07 | 1.95E+07 | 5.88E+07 | 5.88E+07 | 4.88E+07 | 4.38E+07 | 7.51E+07 | 0.12 |
| Com_5991_neg | ethyl 3-cyano-6-methyl-2-(phenylthio)isonicotinate | 6.03E+06 | 4.66E+06 | 1.02E+07 | 2.93E+06 | 3.02E+06 | 1.14E+06 | 5.96E+06 | 1.76E+06 | 7.22E+06 | 2.29E+07 | 0.93 |
| Com_6013_pos | FLK | 2.01E+07 | 4.51E+07 | 1.10E+06 | 7.25E+07 | 1.55E+07 | 4.45E+07 | 1.78E+07 | 2.95E+07 | 2.17E+07 | 5.91E+07 | 0.43 |
| Com_6027_pos | DLK | 2.62E+07 | 3.92E+07 | 4.02E+06 | 7.18E+07 | 3.24E+07 | 6.57E+07 | 5.59E+07 | 6.63E+07 | 1.78E+07 | 7.60E+07 | 0.25 |
| Com_602_pos | Isohomovanillic acid | 2.72E+07 | 9.52E+07 | 1.27E+09 | 9.65E+06 | 2.09E+08 | 3.50E+07 | 1.53E+07 | 1.75E+07 | 1.90E+07 | 2.23E+07 | 0.15 |
| Com_6030_neg | Chenodeoxycholic acid-3-beta-D-glucuronide | 9.25E+06 | 1.33E+07 | 9.01E+06 | 7.21E+06 | 5.70E+06 | 3.84E+06 | 6.54E+06 | 9.66E+05 | 1.02E+07 | 1.39E+07 | 0.34 |
| Com_6031_pos | TLK | 1.49E+07 | 3.10E+07 | 3.97E+06 | 6.23E+07 | 3.53E+07 | 6.56E+07 | 2.62E+07 | 4.74E+07 | 1.66E+07 | 5.08E+07 | 0.33 |
| Com_6048_pos | DL-Homoserine | 1.27E+07 | 1.94E+07 | 7.91E+07 | 2.66E+07 | 8.89E+06 | 6.54E+07 | 1.41E+07 | 3.37E+07 | 2.76E+07 | 2.10E+07 | 0.57 |
| Com_6067_pos | 15(S)-HpETE | 8.29E+07 | 3.38E+07 | 5.18E+07 | 2.92E+07 | 3.71E+07 | 2.59E+07 | 7.87E+07 | 3.75E+07 | 5.77E+07 | 3.08E+07 | 0.92 |
| Com_606_pos | 5-hydroxy-4-methoxy-5,6-dihydro-2H-pyran-2-one | 2.74E+08 | 3.18E+08 | 5.13E+08 | 4.75E+08 | 5.13E+08 | 1.00E+09 | 3.23E+08 | 1.24E+09 | 7.47E+08 | 4.81E+08 | 0.11 |
| Com_6074_pos | 4-morpholinobenzoic acid | 3.56E+07 | 1.66E+07 | 7.88E+07 | 5.05E+07 | 4.49E+06 | 4.13E+06 | 2.44E+07 | 4.25E+06 | 1.22E+07 | 6.02E+07 | 0.36 |
| Com_6089_pos | 2-(acetylamino)-3-(1H-indol-3-yl)propanoic acid | 1.16E+07 | 1.21E+07 | 8.70E+06 | 2.62E+07 | 2.80E+07 | 2.90E+07 | 2.23E+07 | 7.75E+07 | 1.81E+07 | 1.41E+07 | 0.20 |
| Com_6110_pos | (2E,4E)-N-[2-(4-hydroxyphenyl)ethyl]dodeca-2,4-dienamide | 4.88E+07 | 5.38E+07 | 7.81E+07 | 3.06E+07 | 2.36E+07 | 1.61E+07 | 3.91E+07 | 3.20E+07 | 3.10E+07 | 4.62E+07 | 0.27 |
| Com_613_neg | alpha-Benzylsuccinic acid | 2.07E+08 | 1.22E+08 | 4.97E+08 | 9.59E+07 | 1.81E+08 | 2.24E+08 | 3.44E+08 | 7.82E+07 | 1.20E+08 | 1.72E+08 | 0.77 |
| Com_614_pos | (2E,4E)-N-(2-methylpropyl)dodeca-2,4-dienamide | 7.00E+08 | 8.67E+08 | 1.24E+09 | 8.80E+08 | 7.25E+08 | 9.10E+08 | 1.09E+09 | 9.14E+08 | 5.71E+08 | 1.01E+09 | 0.92 |
| Com_6158_pos | Tyramine | 1.95E+07 | 6.15E+06 | 2.61E+07 | 4.86E+07 | 4.13E+06 | 6.13E+06 | 7.24E+06 | 3.68E+06 | 6.56E+07 | 1.51E+07 | 0.70 |
| Com_6168_neg | N6-Methyladenine | 3.67E+06 | 8.31E+06 | 3.97E+06 | 1.26E+07 | 2.19E+06 | 4.59E+06 | 1.72E+07 | 1.05E+06 | 2.25E+06 | 2.18E+07 | 0.96 |
| Com_6177_pos | 5α-Tetrahydrocortisol | 9.45E+06 | 3.15E+07 | 1.38E+07 | 3.17E+07 | 4.15E+07 | 6.33E+07 | 6.13E+07 | 5.53E+07 | 4.34E+07 | 2.71E+07 | 0.05 |
| Com_6190_pos | 4-Acetamidobutyric Acid | 4.19E+07 | 3.42E+07 | 7.52E+07 | 3.70E+07 | 5.94E+07 | 4.75E+07 | 6.01E+07 | 4.11E+07 | 2.89E+07 | 5.45E+07 | 0.81 |
| Com_6194_neg | Capryloylglycine | 1.06E+06 | 2.78E+06 | 1.38E+06 | 1.94E+07 | 5.18E+06 | 3.24E+06 | 4.69E+06 | 7.42E+06 | 3.89E+06 | 1.76E+07 | 0.36 |
| Com_619_pos | Phenethylamine | 4.68E+07 | 4.34E+07 | 1.48E+07 | 1.51E+08 | 1.39E+08 | 1.01E+09 | 1.16E+08 | 1.94E+08 | 1.65E+07 | 3.77E+07 | 0.48 |
| Com_61_pos | PC (16:2e/2:0) | 7.48E+07 | 2.34E+09 | 7.42E+07 | 1.68E+08 | 1.07E+10 | 3.87E+09 | 1.22E+08 | 7.73E+09 | 1.81E+07 | 2.96E+07 | 0.76 |
| Com_6204_neg | Terephthalic Acid | 4.55E+06 | 2.14E+06 | 2.50E+06 | 2.10E+06 | 4.30E+06 | 1.34E+07 | 4.38E+06 | 1.57E+07 | 9.70E+05 | 1.91E+06 | 0.50 |
| Com_621_neg | 4-(hydroxymethyl)benzoic acid | 8.12E+07 | 2.70E+08 | 7.08E+07 | 2.01E+08 | 3.86E+08 | 9.75E+07 | 3.10E+07 | 4.49E+07 | 9.09E+07 | 1.12E+08 | 0.07 |
| Com_6243_pos | 1,2-dihydroxyheptadec-16-yn-4-yl acetate | 6.52E+07 | 2.62E+07 | 3.52E+07 | 4.64E+07 | 4.17E+07 | 5.89E+07 | 3.80E+07 | 4.67E+07 | 3.17E+07 | 3.31E+07 | 0.95 |
| Com_6248_pos | Spermidine | 6.33E+07 | 6.16E+07 | 1.66E+07 | 2.47E+07 | 5.97E+07 | 4.06E+07 | 8.07E+06 | 7.24E+07 | 4.55E+07 | 5.08E+07 | 0.82 |
| Com_6250_neg | hydroxyphenylpyruvate | 4.80E+06 | 9.24E+06 | 5.54E+06 | 2.79E+06 | 6.09E+06 | 1.17E+07 | 1.35E+07 | 2.16E+07 | 1.47E+06 | 7.39E+06 | 0.42 |
| Com_6250_pos | Arachidonoyl ethanolamide phosphate | 5.03E+06 | 2.03E+06 | 8.89E+05 | 1.04E+06 | 1.35E+06 | 8.07E+05 | 1.17E+06 | 9.07E+05 | 6.42E+07 | 1.30E+06 | 0.71 |
| Com_6263_pos | Nor-9-carboxy-δ9-THC | 2.04E+07 | 2.60E+07 | 1.29E+07 | 3.60E+07 | 2.37E+07 | 6.21E+07 | 5.76E+07 | 3.72E+07 | 2.99E+07 | 1.14E+07 | 0.28 |
| Com_6266_pos | O-Arachidonoyl ethanolamine | 3.95E+07 | 3.40E+07 | 6.37E+07 | 5.09E+07 | 2.93E+07 | 6.21E+07 | 5.05E+07 | 4.02E+07 | 5.58E+07 | 3.78E+07 | 0.41 |
| Com_6268_neg | 15(R),19(R)-Hydroxy prostaglandin F1α | 8.44E+06 | 1.02E+07 | 8.43E+06 | 1.45E+07 | 7.42E+06 | 1.08E+07 | 2.07E+07 | 5.67E+06 | 1.22E+07 | 1.16E+07 | 0.50 |
| Com_626_pos | Stercobilin | 1.58E+08 | 8.42E+07 | 9.82E+07 | 2.76E+07 | 9.65E+08 | 1.05E+08 | 1.41E+08 | 1.91E+06 | 3.20E+07 | 1.01E+08 | 0.26 |
| Com_6272_pos | Prostaglandin G2 | 1.79E+06 | 1.71E+06 | 2.24E+06 | 2.19E+06 | 1.46E+06 | 1.53E+06 | 9.09E+07 | 1.63E+06 | 2.02E+06 | 1.69E+06 | 0.42 |
| Com_6285_pos | Temazepam | 1.89E+07 | 3.09E+07 | 3.30E+07 | 2.97E+07 | 4.40E+07 | 5.18E+07 | 5.52E+07 | 7.42E+07 | 2.35E+07 | 3.63E+07 | 0.14 |
| Com_628_neg | Mevalonic acid | 6.49E+07 | 8.32E+07 | 1.80E+08 | 1.32E+08 | 1.74E+08 | 1.60E+08 | 5.99E+08 | 2.64E+08 | 1.27E+08 | 1.52E+08 | 0.12 |
| Com_6300_pos | Lysopc 16:2 (2N Isomer) | 3.67E+07 | 5.37E+07 | 7.53E+06 | 6.52E+06 | 1.60E+07 | 8.65E+06 | 2.01E+07 | 7.38E+07 | 3.82E+07 | 2.06E+07 | 0.52 |
| Com_6321_neg | 6-Sialyllactose | 1.84E+06 | 6.47E+06 | 2.80E+06 | 1.70E+06 | 1.77E+07 | 7.11E+05 | 7.44E+06 | 2.07E+07 | 1.80E+06 | 3.02E+06 | 0.89 |
| Com_633_neg | 4-Oxoproline | 7.25E+07 | 5.33E+07 | 1.95E+08 | 8.67E+07 | 8.37E+07 | 2.72E+08 | 1.11E+08 | 2.84E+08 | 1.46E+08 | 1.51E+08 | 0.04 |
| Com_6340_pos | Isobutyryl carnitine | 1.93E+07 | 3.05E+07 | 6.81E+07 | 2.46E+07 | 5.87E+07 | 4.19E+07 | 3.93E+07 | 3.20E+07 | 2.92E+07 | 8.56E+06 | 0.46 |
| Com_6348_pos | Deoxycytidine | 1.48E+07 | 2.18E+07 | 1.61E+07 | 1.78E+07 | 3.15E+07 | 6.08E+07 | 2.77E+07 | 5.56E+07 | 3.90E+07 | 2.73E+07 | 0.01 |
| Com_635_pos | N-(5-Aminopentyl)acetamide | 5.31E+07 | 4.13E+07 | 1.20E+09 | 5.14E+08 | 9.80E+07 | 3.11E+08 | 2.49E+08 | 7.35E+07 | 2.69E+07 | 7.41E+07 | 0.56 |
| Com_6361_pos | 3-(4-hydroxy-3-methoxyphenyl)propanoic acid | 6.14E+06 | 4.05E+06 | 5.90E+06 | 2.61E+07 | 4.61E+06 | 1.06E+07 | 2.01E+07 | 7.81E+06 | 2.10E+07 | 7.08E+07 | 0.09 |
| Com_6378_pos | 8-Aminooctanoic acid | 3.64E+07 | 4.53E+07 | 6.84E+06 | 1.91E+07 | 5.82E+07 | 1.23E+07 | 2.05E+07 | 1.43E+07 | 2.60E+07 | 2.64E+07 | 0.47 |
| Com_6381_pos | LPE 16:2 | 1.53E+07 | 5.98E+07 | 1.03E+07 | 7.94E+06 | 2.81E+07 | 9.20E+06 | 1.04E+07 | 2.44E+07 | 1.27E+07 | 1.63E+07 | 0.49 |
| Com_63_pos | PC (18:4e/2:0) | 1.67E+08 | 1.01E+09 | 4.51E+07 | 2.16E+08 | 1.05E+10 | 5.78E+09 | 3.61E+08 | 3.32E+09 | 1.58E+07 | 2.07E+07 | 0.80 |
| Com_6410_pos | 3-Amino-4-methylpentanoic acid | 9.11E+06 | 1.26E+07 | 1.35E+07 | 4.99E+07 | 1.27E+07 | 6.00E+07 | 1.42E+07 | 4.78E+07 | 1.07E+07 | 2.14E+07 | 0.34 |
| Com_6412_neg | 15(R),19(R)-Hydroxy prostaglandin F2α | 1.48E+07 | 4.52E+06 | 1.69E+07 | 1.25E+07 | 1.54E+07 | 7.15E+06 | 1.58E+07 | 7.08E+06 | 1.11E+07 | 2.02E+07 | 0.91 |
| Com_6412_pos | 2-Aminobenzenesulfonic acid | 6.54E+06 | 9.74E+06 | 1.21E+07 | 1.22E+07 | 1.04E+07 | 1.10E+07 | 9.17E+06 | 7.18E+07 | 1.62E+07 | 1.27E+07 | 0.22 |
| Com_6419_pos | 1-(2-furyl)pentane-1,4-dione | 3.00E+06 | 4.01E+06 | 4.17E+07 | 3.83E+07 | 8.85E+06 | 5.72E+07 | 2.55E+07 | 7.17E+07 | 6.49E+06 | 1.29E+07 | 0.30 |
| Com_6421_pos | (+/-)-CP 47,497-C7-Hydroxy metabolite | 7.69E+06 | 2.27E+07 | 7.45E+06 | 6.18E+06 | 5.75E+07 | 4.43E+07 | 8.35E+06 | 2.86E+07 | 4.87E+06 | 6.84E+06 | 0.93 |
| Com_6461_neg | LPG 20:4 | 5.79E+06 | 4.79E+06 | 7.03E+05 | 1.18E+06 | 1.64E+07 | 1.26E+07 | 6.23E+05 | 4.10E+06 | 3.39E+05 | 8.34E+05 | 0.42 |
| Com_6462_pos | 2-[2-(1-isobutylcyclohexyl)-1-methylethylidene]hydrazine-1-carboxamide | 1.82E+07 | 3.17E+07 | 2.94E+07 | 4.44E+07 | 5.74E+06 | 2.55E+07 | 2.14E+07 | 8.90E+06 | 4.00E+07 | 6.93E+07 | 0.66 |
| Com_6463_pos | 2-phenyl-4H-furo[2,3-h]chromen-4-one | 2.28E+07 | 4.04E+07 | 6.77E+06 | 2.13E+07 | 3.13E+07 | 2.20E+07 | 6.69E+07 | 7.11E+07 | 1.67E+07 | 2.11E+07 | 0.35 |
| Com_6468_neg | Sulfamethoxazole hydroxylamine | 3.06E+06 | 4.82E+06 | 4.14E+05 | 8.81E+06 | 9.27E+06 | 1.26E+07 | 1.69E+07 | 1.73E+07 | 1.35E+06 | 8.27E+06 | 0.27 |
| Com_6471_neg | 9-KODE | 1.09E+07 | 1.10E+07 | 1.57E+07 | 1.29E+07 | 1.13E+07 | 7.63E+06 | 1.31E+07 | 1.62E+07 | 1.17E+07 | 1.53E+07 | 0.94 |
| Com_6496_pos | L-Histidinol | 3.16E+07 | 2.75E+07 | 4.64E+07 | 1.25E+07 | 5.68E+07 | 1.02E+07 | 3.61E+07 | 3.84E+07 | 2.57E+07 | 3.46E+07 | 0.66 |
| Com_64_pos | Stearamide | 6.22E+09 | 5.86E+09 | 1.30E+10 | 3.63E+09 | 7.01E+09 | 2.41E+09 | 7.18E+09 | 5.53E+09 | 2.99E+09 | 5.10E+09 | 0.18 |
| Com_650_neg | Quinic acid | 1.35E+07 | 2.88E+07 | 1.50E+07 | 9.21E+06 | 3.59E+08 | 3.15E+06 | 1.86E+07 | 8.11E+07 | 1.99E+06 | 5.79E+06 | 0.25 |
| Com_6533_pos | 3-(3-Methoxyphenyl)propionic acid | 7.46E+07 | 1.62E+07 | 9.22E+06 | 2.10E+07 | 1.82E+07 | 5.83E+07 | 1.42E+07 | 5.30E+06 | 1.34E+07 | 2.07E+07 | 0.64 |
| Com_6542_pos | WKK | 1.51E+07 | 4.37E+07 | 4.71E+07 | 6.43E+07 | 2.75E+07 | 1.13E+07 | 1.63E+07 | 1.68E+07 | 2.73E+07 | 5.26E+07 | 0.21 |
| Com_6543_pos | Pregnenolone | 7.46E+07 | 1.03E+07 | 7.00E+07 | 2.16E+07 | 2.09E+07 | 5.08E+07 | 3.22E+07 | 5.11E+07 | 2.05E+07 | 1.77E+07 | 0.93 |
| Com_6547_pos | RKK | 3.51E+07 | 5.55E+07 | 3.44E+07 | 2.13E+07 | 3.92E+07 | 5.84E+07 | 2.49E+07 | 2.15E+07 | 3.10E+07 | 2.45E+07 | 0.49 |
| Com_6548_neg | Chenodeoxycholic Acid | 1.07E+07 | 4.71E+06 | 1.01E+07 | 4.79E+06 | 5.50E+06 | 1.24E+07 | 1.99E+06 | 8.38E+06 | 5.92E+06 | 6.00E+06 | 0.74 |
| Com_654_pos | 4-Phenylbutyric acid | 8.96E+08 | 1.31E+08 | 1.03E+09 | 9.84E+08 | 5.48E+08 | 5.47E+08 | 3.85E+08 | 1.86E+07 | 8.72E+08 | 6.26E+08 | 0.42 |
| Com_6566_neg | N-Methylhydantoin | 3.68E+06 | 4.42E+06 | 5.74E+06 | 5.09E+06 | 7.47E+06 | 6.59E+06 | 9.09E+06 | 6.23E+06 | 1.14E+07 | 5.31E+06 | 0.08 |
| Com_65_neg | 7-Ketolithocholic acid | 4.23E+09 | 4.00E+09 | 4.28E+09 | 2.54E+09 | 2.02E+09 | 1.24E+09 | 2.27E+09 | 1.97E+08 | 2.62E+09 | 4.82E+09 | 0.23 |
| Com_6610_pos | N-Acetyl-L-methionine | 3.37E+07 | 3.95E+07 | 4.39E+07 | 2.68E+07 | 3.36E+07 | 2.28E+07 | 5.09E+07 | 3.89E+07 | 5.94E+07 | 4.68E+07 | 0.39 |
| Com_6615_neg | Guanosine monophosphate (GMP) | 8.17E+05 | 6.28E+05 | 2.17E+07 | 2.52E+06 | 1.10E+06 | 7.10E+05 | 1.25E+06 | 1.29E+06 | 5.70E+05 | 8.62E+05 | 0.29 |
| Com_6640_pos | 4-methyl-5-oxo-2-pentyl-2,5-dihydrofuran-3-carboxylic acid | 4.09E+07 | 3.93E+07 | 4.21E+07 | 1.08E+07 | 1.54E+07 | 5.72E+07 | 3.12E+07 | 2.75E+07 | 1.36E+07 | 1.54E+07 | 0.96 |
| Com_666_pos | N-Acetylneuraminic acid | 6.37E+07 | 1.30E+08 | 5.71E+07 | 1.27E+08 | 4.35E+07 | 9.21E+08 | 1.88E+08 | 1.34E+08 | 1.11E+08 | 1.60E+08 | 0.06 |
| Com_6674_pos | 2-(14,15-Epoxyeicosatrienoyl) glycerol | 1.75E+07 | 2.21E+07 | 2.99E+07 | 1.17E+07 | 2.36E+07 | 8.99E+06 | 1.98E+07 | 6.79E+07 | 1.36E+07 | 1.46E+07 | 0.89 |
| Com_66_neg | 2-Hydroxy-2-methyl-3-buten-1-yl beta-D-glucopyranoside | 9.36E+05 | 1.05E+06 | 3.87E+06 | 1.18E+07 | 2.58E+06 | 4.14E+09 | 6.75E+07 | 2.36E+07 | 2.42E+06 | 3.81E+08 | 0.04 |
| Com_66_pos | MAG (18:4) | 2.94E+09 | 2.10E+09 | 7.88E+08 | 3.10E+08 | 2.78E+09 | 1.04E+10 | 7.56E+08 | 3.60E+09 | 3.83E+09 | 2.52E+09 | 0.21 |
| Com_6701_pos | RLK | 1.59E+06 | 1.64E+06 | 6.84E+07 | 1.45E+06 | 1.39E+06 | 1.71E+07 | 1.24E+06 | 1.83E+06 | 1.46E+06 | 1.70E+06 | 0.78 |
| Com_6706_pos | Hydroquinone | 3.04E+07 | 4.59E+07 | 2.29E+07 | 2.85E+07 | 1.86E+07 | 1.28E+07 | 3.43E+07 | 3.61E+07 | 5.81E+07 | 2.05E+07 | 0.95 |
| Com_6725_neg | LPC 22:4 | 2.12E+06 | 1.12E+07 | 3.98E+06 | 2.95E+06 | 2.44E+06 | 3.75E+06 | 4.50E+06 | 1.84E+06 | 1.51E+06 | 1.91E+06 | 0.30 |
| Com_6748_neg | Gallic acid | 1.79E+06 | 4.08E+06 | 2.21E+06 | 1.78E+06 | 6.08E+06 | 8.35E+06 | 7.71E+06 | 1.91E+07 | 1.41E+06 | 5.91E+06 | 0.14 |
| Com_6757_neg | Lysopc 18:3 | 7.87E+06 | 8.71E+06 | 4.76E+06 | 2.43E+06 | 7.47E+06 | 1.17E+07 | 5.54E+06 | 1.91E+07 | 3.46E+06 | 4.88E+06 | 0.54 |
| Com_6757_pos | Argininosuccinic acid | 1.34E+07 | 1.94E+07 | 3.65E+07 | 3.39E+07 | 2.67E+07 | 5.57E+07 | 6.37E+07 | 3.73E+07 | 2.59E+07 | 3.52E+07 | 0.07 |
| Com_6766_pos | 3-Methyladenine | 5.26E+06 | 5.03E+06 | 2.87E+07 | 5.68E+07 | 4.04E+06 | 1.83E+07 | 2.97E+07 | 1.98E+07 | 8.25E+06 | 6.49E+07 | 0.31 |
| Com_6797_neg | 5beta-Androstane-3,17-dione | 1.37E+07 | 9.49E+06 | 7.19E+06 | 1.23E+07 | 1.47E+07 | 5.46E+06 | 2.06E+07 | 1.89E+07 | 7.93E+06 | 1.01E+07 | 1.00 |
| Com_67_pos | LPC 22:6 | 1.01E+09 | 3.80E+09 | 2.83E+08 | 2.98E+08 | 7.02E+09 | 3.51E+09 | 1.09E+09 | 1.24E+10 | 9.36E+07 | 2.24E+08 | 0.88 |
| Com_6801_pos | Biliverdin | 1.49E+06 | 5.47E+05 | 5.16E+06 | 3.20E+06 | 7.17E+05 | 5.52E+07 | 8.43E+06 | 2.35E+06 | 2.78E+06 | 2.81E+06 | 0.10 |
| Com_6827_pos | NPH | 3.81E+06 | 1.46E+07 | 2.90E+06 | 2.54E+06 | 6.74E+06 | 3.56E+07 | 2.01E+07 | 6.57E+07 | 2.71E+06 | 3.74E+06 | 0.19 |
| Com_6830_pos | N-α-L-Acetyl-arginine | 3.11E+07 | 2.14E+07 | 5.81E+07 | 4.21E+07 | 2.97E+07 | 4.43E+07 | 3.03E+07 | 5.76E+07 | 2.14E+07 | 6.39E+07 | 0.57 |
| Com_6842_pos | Thiazolidine-4-carboxylic acid | 6.43E+06 | 5.31E+06 | 5.61E+07 | 5.68E+07 | 2.05E+07 | 5.46E+07 | 1.93E+07 | 4.24E+07 | 5.37E+07 | 4.24E+07 | 0.22 |
| Com_685_pos | KPK | 6.41E+08 | 3.20E+08 | 3.44E+08 | 2.34E+08 | 3.44E+08 | 8.89E+08 | 2.24E+08 | 5.19E+08 | 5.67E+08 | 5.53E+08 | 0.25 |
| Com_6878_pos | trans-Cinnamaldehyde | 6.91E+07 | 5.04E+07 | 4.28E+06 | 4.64E+07 | 2.69E+07 | 1.67E+07 | 1.86E+07 | 1.06E+07 | 1.24E+07 | 1.21E+07 | 0.22 |
| Com_6880_pos | 16-Heptadecyne-1,2,4-triol | 2.08E+07 | 6.11E+06 | 6.57E+07 | 3.63E+07 | 4.60E+06 | 1.76E+07 | 1.85E+07 | 2.44E+07 | 1.91E+07 | 1.94E+07 | 0.78 |
| Com_6896_neg | Pro-Leu | 5.76E+05 | 5.77E+05 | 6.70E+05 | 1.26E+07 | 7.21E+05 | 3.67E+05 | 2.44E+07 | 4.18E+06 | 1.05E+07 | 6.16E+05 | 0.37 |
| Com_6897_pos | Oleoyl-L-alpha-lysophosphatidic acid | 1.70E+06 | 3.03E+06 | 2.19E+06 | 3.34E+05 | 1.66E+06 | 5.39E+07 | 5.88E+06 | 1.05E+07 | 7.43E+05 | 4.60E+06 | 0.10 |
| Com_6900_neg | N1-(3-amino-4-chlorophenyl)-2-[2,4-di(tert-pentyl)phenoxy]acetamide | 7.61E+06 | 1.07E+07 | 3.01E+06 | 2.00E+06 | 7.41E+06 | 6.27E+06 | 2.47E+06 | 2.09E+06 | 2.58E+06 | 9.39E+05 | 0.12 |
| Com_6929_pos | Stiripentol | 2.74E+06 | 2.56E+06 | 6.50E+07 | 1.98E+06 | 3.07E+06 | 5.82E+06 | 3.19E+06 | 2.48E+06 | 2.10E+06 | 2.98E+06 | 0.54 |
| Com_6938_pos | 3-(4-fluorophenoxy)-1-(1,4-thiazinan-4-yl)propan-1-one | 7.91E+06 | 1.50E+07 | 1.16E+07 | 3.05E+07 | 1.20E+07 | 1.86E+07 | 3.09E+07 | 1.07E+07 | 5.51E+07 | 3.78E+07 | 0.11 |
| Com_6942_pos | LPC 18:4 | 1.42E+07 | 2.71E+07 | 1.25E+07 | 9.15E+06 | 5.14E+07 | 1.32E+07 | 1.36E+07 | 4.38E+07 | 1.08E+07 | 1.42E+07 | 0.76 |
| Com_6951_pos | L-Saccharopine | 1.41E+07 | 2.32E+07 | 2.25E+07 | 2.01E+07 | 4.91E+07 | 4.50E+07 | 4.22E+07 | 3.98E+07 | 5.50E+07 | 5.55E+07 | 0.02 |
| Com_6953_pos | (S)-beta-Aminoisobutyric Acid | 3.08E+07 | 3.37E+07 | 2.17E+07 | 5.86E+07 | 2.52E+07 | 1.20E+07 | 2.39E+07 | 7.16E+06 | 3.52E+07 | 2.27E+07 | 0.11 |
| Com_6960_pos | 3-N-Methyl-L-histidine | 2.97E+07 | 3.07E+07 | 3.52E+07 | 2.41E+07 | 5.12E+07 | 1.82E+07 | 3.51E+07 | 4.04E+07 | 1.56E+07 | 4.01E+07 | 0.48 |
| Com_6961_pos | Chrysin | 1.19E+07 | 1.15E+07 | 1.15E+07 | 2.05E+07 | 1.78E+07 | 3.45E+07 | 6.86E+06 | 6.37E+07 | 3.71E+07 | 1.62E+07 | 0.23 |
| Com_6962_neg | N2-Methylguanosine | 1.94E+06 | 2.09E+06 | 4.77E+06 | 1.61E+07 | 2.75E+06 | 4.37E+06 | 5.93E+06 | 7.71E+06 | 2.56E+06 | 1.06E+07 | 0.45 |
| Com_6996_pos | 2-acetamido-3-(4-methoxyphenyl)propanoic acid | 1.36E+07 | 3.12E+07 | 6.40E+07 | 6.64E+06 | 9.74E+06 | 1.05E+07 | 1.33E+07 | 1.65E+07 | 1.24E+07 | 2.03E+07 | 0.63 |
| Com_699_pos | N-Ethylglycine | 7.50E+07 | 2.74E+08 | 1.75E+08 | 9.59E+08 | 9.43E+07 | 2.75E+08 | 2.61E+08 | 3.03E+08 | 3.86E+08 | 4.82E+08 | 0.33 |
| Com_69_pos | L-Valine | 1.47E+09 | 2.28E+09 | 2.33E+09 | 1.11E+10 | 2.70E+09 | 4.89E+09 | 5.46E+09 | 7.15E+09 | 2.70E+09 | 6.67E+09 | 0.21 |
| Com_700_pos | N-Acetylhistamine | 3.08E+07 | 1.80E+07 | 1.06E+09 | 8.30E+08 | 2.76E+08 | 1.12E+08 | 2.39E+08 | 2.00E+08 | 3.54E+08 | 2.23E+08 | 0.80 |
| Com_7032_pos | Linolelaidic Acid (C18:2N6T) | 3.09E+07 | 1.14E+07 | 2.70E+07 | 2.21E+07 | 5.04E+07 | 2.24E+07 | 1.61E+07 | 1.97E+07 | 2.26E+07 | 2.91E+07 | 0.56 |
| Com_7034_pos | Berberine | 1.46E+06 | 1.07E+07 | 2.86E+06 | 5.76E+07 | 1.08E+06 | 3.65E+06 | 2.56E+06 | 2.06E+06 | 2.18E+06 | 3.34E+06 | 0.46 |
| Com_7043_pos | XLR11 N-(4-hydroxypentyl) metabolite | 5.47E+05 | 1.31E+06 | 6.34E+07 | 4.31E+05 | 1.14E+06 | 9.02E+06 | 7.35E+05 | 5.32E+06 | 3.66E+05 | 7.40E+05 | 0.88 |
| Com_7049_pos | Cafestol | 6.66E+07 | 1.80E+07 | 1.14E+07 | 2.97E+07 | 2.05E+07 | 2.81E+07 | 2.95E+07 | 1.90E+07 | 2.92E+07 | 2.87E+07 | 0.78 |
| Com_704_neg | cis-2-Decenoic acid | 2.94E+07 | 1.76E+07 | 1.60E+07 | 2.13E+07 | 3.26E+08 | 2.32E+07 | 2.47E+07 | 1.78E+08 | 5.11E+07 | 2.73E+07 | 0.80 |
| Com_704_pos | 1-Methylnicotinamide | 2.68E+07 | 2.09E+07 | 1.05E+09 | 8.24E+08 | 2.72E+08 | 1.11E+08 | 2.37E+08 | 1.98E+08 | 3.51E+08 | 2.20E+08 | 0.80 |
| Com_7056_pos | (+/-)17(18)-EpETE methyl ester | 1.25E+07 | 2.64E+07 | 1.88E+07 | 3.21E+07 | 5.02E+07 | 6.71E+06 | 1.68E+07 | 3.99E+07 | 2.42E+06 | 1.94E+07 | 0.20 |
| Com_7064_pos | Thromoboxane B1 | 3.95E+07 | 4.91E+07 | 5.20E+07 | 3.47E+07 | 5.01E+07 | 4.09E+07 | 4.25E+07 | 2.55E+07 | 4.90E+07 | 4.87E+07 | 0.50 |
| Com_7066_pos | 3-Hydroxylidocaine | 5.28E+07 | 5.15E+07 | 2.64E+06 | 8.20E+06 | 4.23E+06 | 1.52E+06 | 1.73E+06 | 1.98E+06 | 1.69E+06 | 1.68E+07 | 0.09 |
| Com_7121_neg | 4-oxo-4,5,6,7-tetrahydrobenzo[b]furan-3-carboxylic acid | 1.70E+06 | 1.58E+06 | 3.75E+06 | 1.82E+06 | 2.18E+06 | 1.07E+07 | 5.32E+06 | 3.03E+06 | 1.16E+06 | 3.36E+06 | 0.21 |
| Com_7131_neg | Sorbitan monopalmitate | 3.75E+06 | 1.00E+06 | 1.37E+06 | 9.20E+05 | 1.33E+07 | 6.52E+06 | 9.28E+06 | 1.74E+07 | 2.74E+06 | 1.83E+06 | 0.21 |
| Com_713_pos | 5,6-dimethoxy-2-(2-methoxyphenyl)-4H-chromen-4-one | 7.88E+08 | 8.44E+08 | 1.90E+07 | 1.90E+08 | 6.82E+08 | 1.60E+08 | 3.78E+08 | 1.42E+08 | 7.36E+08 | 2.87E+08 | 0.98 |
| Com_7147_pos | Carbaprostacyclin | 3.25E+07 | 1.32E+07 | 1.07E+07 | 2.37E+07 | 7.15E+06 | 2.96E+07 | 2.54E+07 | 6.14E+07 | 1.57E+07 | 1.45E+07 | 0.20 |
| Com_7181_neg | N-Butylbenzenesulfonamide | 1.38E+07 | 4.53E+06 | 8.89E+06 | 1.46E+07 | 6.84E+06 | 4.13E+06 | 1.13E+07 | 7.92E+06 | 4.59E+06 | 6.30E+06 | 0.28 |
| Com_7196_neg | 4-Hydroxybenzylalcohol | 9.51E+05 | 7.49E+05 | 1.12E+06 | 6.30E+06 | 1.13E+06 | 6.41E+06 | 1.54E+07 | 3.91E+06 | 9.80E+06 | 8.83E+06 | 0.01 |
| Com_7201_pos | 15-OxoEDE | 5.34E+07 | 5.01E+07 | 2.46E+07 | 2.29E+07 | 4.72E+07 | 4.15E+07 | 3.10E+07 | 2.94E+07 | 2.45E+07 | 4.69E+07 | 0.67 |
| Com_720_pos | Naringeninchalcone | 3.02E+08 | 9.53E+07 | 9.65E+07 | 8.84E+07 | 2.01E+08 | 8.41E+08 | 1.60E+08 | 9.98E+08 | 5.15E+07 | 5.65E+07 | 0.57 |
| Com_7219_pos | (3R)-8-hydroxy-3-(4-hydroxyphenyl)-3,4-dihydro-1H-2-benzopyran-1-one | 4.50E+07 | 4.99E+07 | 2.09E+06 | 7.49E+06 | 1.54E+07 | 5.47E+06 | 9.77E+06 | 5.40E+06 | 2.01E+07 | 1.13E+07 | 0.54 |
| Com_7226_pos | 3-Methyl-2-oxobutanoic acid | 2.90E+07 | 2.60E+07 | 3.96E+07 | 3.06E+07 | 4.85E+07 | 3.07E+07 | 3.77E+07 | 4.71E+07 | 2.39E+07 | 3.83E+07 | 0.88 |
| Com_723_pos | N-Acetylmethionine | 8.65E+07 | 1.19E+08 | 1.41E+08 | 1.16E+08 | 3.29E+08 | 7.66E+07 | 2.69E+08 | 1.01E+09 | 6.99E+07 | 1.62E+08 | 0.61 |
| Com_7249_neg | Neopterin | 6.34E+06 | 2.66E+06 | 1.90E+06 | 1.31E+07 | 9.72E+05 | 2.55E+06 | 1.15E+06 | 1.40E+06 | 9.68E+06 | 1.16E+07 | 0.97 |
| Com_725_pos | Kanosamine | 1.41E+08 | 1.52E+08 | 3.37E+08 | 3.47E+08 | 1.52E+08 | 8.38E+08 | 1.53E+08 | 8.24E+08 | 6.11E+08 | 3.29E+08 | 0.08 |
| Com_7264_pos | Epinephrine | 1.91E+07 | 3.56E+07 | 1.73E+07 | 2.50E+07 | 5.12E+06 | 1.09E+07 | 1.85E+07 | 5.34E+06 | 5.15E+07 | 1.75E+07 | 0.86 |
| Com_7269_neg | FAHFA (5:0/18:1) | 9.01E+05 | 2.83E+06 | 5.81E+06 | 1.50E+07 | 1.36E+06 | 5.80E+05 | 1.65E+06 | 1.39E+06 | 1.06E+06 | 1.78E+06 | 0.14 |
| Com_7271_pos | Deoxyinosine | 6.44E+06 | 1.21E+07 | 4.21E+07 | 1.40E+07 | 1.38E+07 | 8.15E+06 | 1.02E+07 | 9.46E+06 | 3.38E+07 | 5.82E+07 | 0.73 |
| Com_7308_neg | Anacardic acid | 6.78E+06 | 9.79E+06 | 1.03E+07 | 7.39E+06 | 5.38E+06 | 3.33E+06 | 8.59E+06 | 2.52E+06 | 8.28E+06 | 9.77E+06 | 0.35 |
| Com_7314_pos | ACar 13:0 | 4.18E+06 | 3.98E+06 | 5.99E+07 | 4.37E+06 | 3.98E+06 | 1.26E+07 | 3.51E+06 | 9.41E+06 | 1.73E+06 | 3.03E+06 | 0.54 |
| Com_735_pos | LPE 16:1 | 7.44E+06 | 8.19E+08 | 7.31E+06 | 1.77E+07 | 1.62E+08 | 2.63E+07 | 6.78E+07 | 3.29E+08 | 6.25E+06 | 6.23E+06 | 0.78 |
| Com_737_neg | (2R)-2,3-Dihydroxypropanoic acid | 8.53E+07 | 9.90E+07 | 9.40E+07 | 1.85E+08 | 1.24E+08 | 6.39E+07 | 4.94E+08 | 2.08E+08 | 9.36E+07 | 1.75E+08 | 0.39 |
| Com_738_neg | 5-Methoxysalicylic acid | 3.34E+06 | 1.65E+07 | 7.53E+06 | 5.28E+07 | 1.13E+07 | 8.49E+07 | 4.94E+08 | 1.81E+08 | 9.40E+07 | 9.73E+07 | 0.00 |
| Com_7404_neg | DL-α-Methoxyphenylacetic acid | 2.64E+05 | 2.67E+05 | 5.48E+05 | 5.12E+05 | 4.55E+06 | 3.02E+05 | 1.65E+07 | 1.57E+07 | 9.36E+06 | 5.55E+05 | 0.14 |
| Com_7408_pos | L-Cystine | 4.84E+06 | 3.45E+06 | 1.53E+07 | 2.59E+07 | 2.05E+07 | 2.50E+07 | 1.55E+07 | 5.80E+07 | 1.04E+07 | 1.86E+07 | 0.20 |
| Com_7412_neg | 4-Hydroxy-6-methyl-2-pyrone | 3.69E+06 | 2.74E+06 | 1.80E+07 | 4.10E+06 | 4.67E+06 | 5.18E+06 | 6.76E+06 | 7.86E+06 | 2.07E+06 | 4.88E+06 | 0.91 |
| Com_7414_pos | 3,4-dihydroxy-4-(4-methoxyphenyl)-1,2,3,4-tetrahydroquinolin-2-one | 4.70E+07 | 4.28E+07 | 3.98E+07 | 1.66E+07 | 3.96E+07 | 3.07E+07 | 3.89E+07 | 2.37E+07 | 4.31E+07 | 2.48E+07 | 0.63 |
| Com_7425_neg | Trehalose 6-phosphate | 1.51E+06 | 3.75E+06 | 4.80E+06 | 1.57E+06 | 1.36E+07 | 1.70E+06 | 2.33E+06 | 3.25E+06 | 1.29E+06 | 1.89E+06 | 0.24 |
| Com_7471_pos | Cinnamoylglycine | 1.26E+07 | 1.75E+07 | 2.21E+06 | 2.50E+07 | 2.24E+07 | 4.79E+07 | 2.17E+07 | 3.62E+07 | 8.96E+06 | 4.14E+07 | 0.19 |
| Com_7500_neg | Phe-Phe | 7.23E+05 | 1.74E+06 | 3.07E+05 | 2.88E+06 | 3.46E+06 | 3.76E+06 | 1.38E+06 | 1.61E+07 | 6.03E+05 | 2.07E+06 | 0.38 |
| Com_7506_neg | 3-(4,5-diphenyl-1H-imidazol-2-yl)pyridine | 1.36E+06 | 2.45E+06 | 3.45E+06 | 5.66E+06 | 3.42E+06 | 7.89E+06 | 3.85E+06 | 1.61E+07 | 2.82E+06 | 5.25E+06 | 0.11 |
| Com_7521_pos | L-Ergothioneine | 1.69E+07 | 1.04E+07 | 5.77E+07 | 1.73E+07 | 1.05E+07 | 3.46E+07 | 1.28E+07 | 1.13E+07 | 1.91E+07 | 2.00E+07 | 0.98 |
| Com_753_pos | N-Acetyl-DL-glutamic acid | 2.15E+08 | 3.33E+08 | 9.79E+08 | 3.72E+08 | 1.26E+08 | 3.51E+08 | 2.00E+08 | 1.07E+08 | 4.26E+08 | 4.13E+08 | 0.68 |
| Com_75_pos | Oleanolic acid | 9.73E+08 | 9.03E+08 | 3.69E+08 | 1.00E+10 | 3.75E+09 | 1.36E+09 | 3.05E+09 | 1.99E+09 | 2.30E+09 | 1.17E+09 | 0.85 |
| Com_7622_pos | (1E)-5-hydroxy-1,7-diphenylhept-1-en-3-one | 3.91E+07 | 4.38E+07 | 1.31E+07 | 2.22E+07 | 7.23E+06 | 4.68E+07 | 1.63E+07 | 4.93E+06 | 2.93E+07 | 3.59E+07 | 0.97 |
| Com_7625_neg | Dl-3,4-Dihydroxymandelic Acid | 1.86E+06 | 1.93E+06 | 1.48E+06 | 1.39E+07 | 8.41E+06 | 6.30E+06 | 1.35E+07 | 8.56E+06 | 1.23E+06 | 1.69E+06 | 0.79 |
| Com_762_pos | p-Mentha-1,3,8-triene | 5.12E+08 | 5.29E+08 | 5.59E+08 | 3.88E+08 | 5.58E+08 | 3.23E+08 | 4.07E+08 | 9.53E+07 | 8.23E+08 | 6.04E+08 | 0.43 |
| Com_7647_pos | 2-Methoxyresorcinol | 9.26E+06 | 1.30E+07 | 1.07E+07 | 7.45E+06 | 4.56E+06 | 4.99E+06 | 6.84E+07 | 4.77E+06 | 1.21E+07 | 1.57E+07 | 0.48 |
| Com_7658_neg | Porphobilinogen | 2.47E+06 | 3.76E+06 | 7.27E+06 | 3.41E+06 | 8.62E+06 | 3.65E+06 | 6.50E+06 | 8.66E+06 | 2.85E+06 | 1.54E+07 | 0.45 |
| Com_7689_pos | Azaspiracid-1 | 8.05E+05 | 8.41E+05 | 5.54E+05 | 7.71E+05 | 8.01E+05 | 5.87E+05 | 7.68E+05 | 6.71E+05 | 4.77E+07 | 8.58E+05 | 0.40 |
| Com_7701_neg | Glucuronic acid-3,6-lactone | 1.78E+06 | 1.02E+06 | 8.64E+06 | 2.68E+06 | 6.50E+06 | 4.32E+06 | 1.50E+07 | 1.54E+07 | 2.40E+06 | 4.67E+06 | 0.21 |
| Com_7717_pos | PC (7:0/8:0) | 1.65E+07 | 1.70E+07 | 5.09E+07 | 2.75E+07 | 3.09E+07 | 4.61E+07 | 5.51E+07 | 4.12E+07 | 1.48E+07 | 1.36E+07 | 0.77 |
| Com_7720_neg | NSI-189 | 1.66E+06 | 2.53E+06 | 1.11E+06 | 2.64E+06 | 1.45E+06 | 9.41E+06 | 3.13E+06 | 2.65E+06 | 2.21E+06 | 1.47E+06 | 0.18 |
| Com_7728_neg | 3-[(methoxycarbonyl)amino]-2,2,3-trimethylbutanoic acid | 1.73E+06 | 3.84E+06 | 3.18E+06 | 9.20E+06 | 1.30E+06 | 8.14E+05 | 8.92E+06 | 5.89E+05 | 1.45E+06 | 1.52E+07 | 0.80 |
| Com_7748_pos | Roquefortine C | 3.88E+06 | 4.79E+06 | 1.94E+06 | 1.86E+07 | 2.12E+06 | 4.59E+07 | 4.35E+06 | 3.24E+07 | 2.72E+06 | 6.68E+06 | 0.24 |
| Com_7754_pos | 3-[2-(3-Hydroxyphenyl)ethyl]-5-methoxyphenol | 5.84E+07 | 3.70E+07 | 7.57E+06 | 1.69E+07 | 1.92E+07 | 4.17E+07 | 3.32E+07 | 5.94E+06 | 1.43E+07 | 2.19E+07 | 0.78 |
| Com_7756_pos | R-1 Methanandamide phosphate | 1.90E+05 | 9.39E+05 | 5.55E+07 | 2.40E+05 | 3.93E+06 | 3.50E+06 | 3.85E+06 | 1.11E+07 | 3.92E+05 | 2.73E+05 | 0.93 |
| Com_7768_pos | 3-phenyl-5-(1,2,3-thiadiazol-4-yl)-1,2,4-oxadiazole | 1.53E+07 | 1.51E+07 | 2.68E+06 | 1.42E+07 | 4.39E+07 | 9.16E+06 | 2.81E+06 | 7.63E+06 | 2.02E+07 | 1.32E+07 | 0.50 |
| Com_7771_neg | LPE 15:0 | 1.28E+06 | 8.83E+06 | 5.50E+06 | 4.08E+06 | 4.76E+06 | 1.18E+06 | 4.84E+06 | 2.82E+06 | 2.23E+06 | 2.64E+06 | 0.23 |
| Com_7776_neg | (±)9(10)-DiHOME | 5.59E+06 | 3.16E+06 | 5.14E+06 | 3.07E+06 | 5.60E+06 | 1.51E+06 | 8.11E+06 | 7.55E+06 | 8.62E+06 | 2.60E+06 | 0.89 |
| Com_7780_pos | PDMP | 5.81E+07 | 1.90E+07 | 3.09E+06 | 1.51E+06 | 1.56E+07 | 1.86E+07 | 2.67E+07 | 1.50E+07 | 2.59E+07 | 2.95E+07 | 0.27 |
| Com_778_pos | 10-Undecenoic acid | 5.60E+08 | 5.24E+08 | 7.45E+08 | 4.34E+08 | 4.96E+08 | 2.16E+08 | 6.28E+08 | 8.36E+07 | 8.09E+08 | 5.63E+08 | 0.36 |
| Com_7795_neg | 1-benzyl-3-(2-methylphenyl)-3,7-dihydro-1H-purine-2,6-dione | 7.36E+06 | 8.80E+06 | 1.19E+06 | 3.62E+06 | 4.71E+06 | 4.00E+06 | 3.24E+06 | 9.18E+05 | 3.42E+06 | 4.74E+06 | 0.43 |
| Com_7803_neg | Resorcinol | 3.73E+06 | 3.59E+06 | 3.55E+06 | 1.96E+06 | 1.26E+07 | 1.50E+06 | 3.79E+06 | 2.51E+06 | 1.63E+06 | 2.62E+06 | 0.14 |
| Com_780_neg | LPC 16:1 | 3.43E+06 | 2.01E+08 | 1.96E+06 | 1.55E+07 | 3.84E+07 | 6.01E+07 | 2.75E+06 | 1.61E+08 | 1.26E+06 | 1.23E+07 | 0.92 |
| Com_7821_neg | Glyceraldehyde 3-phosphate | 1.20E+06 | 1.05E+06 | 1.65E+07 | 2.87E+06 | 3.52E+06 | 4.41E+06 | 5.14E+06 | 8.19E+06 | 1.28E+06 | 3.42E+06 | 0.66 |
| Com_782_pos | PC (14:1e/2:0) | 3.70E+08 | 2.96E+08 | 2.09E+08 | 5.13E+07 | 4.23E+08 | 7.84E+08 | 1.60E+08 | 2.98E+08 | 1.66E+08 | 1.91E+08 | 0.73 |
| Com_7840_neg | Riboflavin-5-phosphate | 7.70E+05 | 5.21E+05 | 7.22E+05 | 5.39E+05 | 5.92E+05 | 9.18E+06 | 8.57E+05 | 2.61E+06 | 1.13E+06 | 1.89E+06 | 0.04 |
| Com_784_neg | Taurochenodeoxycholic Acid (sodium salt) | 2.30E+08 | 2.43E+07 | 2.22E+06 | 4.27E+06 | 1.31E+08 | 7.27E+07 | 6.46E+05 | 3.42E+08 | 9.53E+07 | 1.08E+06 | 0.86 |
| Com_786_neg | 23-Nordeoxycholic acid | 1.14E+08 | 1.20E+08 | 1.69E+08 | 1.75E+08 | 8.33E+07 | 3.09E+07 | 1.07E+08 | 2.65E+07 | 1.95E+08 | 2.43E+08 | 0.43 |
| Com_7874_neg | Salvinorin A | 1.01E+06 | 9.06E+05 | 5.75E+05 | 1.09E+06 | 1.24E+07 | 8.41E+05 | 4.96E+05 | 1.36E+06 | 3.05E+05 | 3.72E+05 | 0.17 |
| Com_7876_pos | N-Acetyl-L-tyrosine | 1.08E+07 | 1.10E+07 | 2.45E+07 | 4.92E+07 | 1.59E+07 | 1.66E+07 | 8.59E+06 | 3.40E+07 | 3.69E+07 | 2.23E+07 | 0.78 |
| Com_788_pos | N-Acetyl-D-galactosamine | 5.41E+08 | 6.84E+08 | 1.63E+08 | 5.09E+08 | 3.65E+08 | 5.24E+08 | 5.97E+08 | 2.91E+08 | 3.37E+08 | 9.03E+08 | 0.59 |
| Com_7913_pos | S-Adenosylmethionine | 8.68E+06 | 1.28E+07 | 9.97E+06 | 3.55E+07 | 2.31E+07 | 2.69E+07 | 1.63E+07 | 1.50E+07 | 4.59E+07 | 3.00E+07 | 0.21 |
| Com_7933_pos | 2-(cyclopropylcarbonyl)-3-(4-fluoroanilino)acrylonitrile | 4.35E+07 | 4.06E+07 | 1.68E+07 | 3.92E+06 | 4.27E+07 | 3.94E+06 | 9.91E+06 | 9.72E+06 | 7.74E+06 | 7.55E+06 | 0.08 |
| Com_7954_neg | PG (5:0/16:0) | 1.98E+06 | 8.47E+06 | 1.82E+06 | 4.05E+06 | 7.24E+06 | 6.87E+05 | 3.78E+06 | 1.14E+07 | 3.09E+06 | 1.45E+06 | 0.52 |
| Com_7960_pos | 4-(2,3-dihydro-1,4-benzodioxin-6-yl)butanoic acid | 1.04E+07 | 9.17E+06 | 3.82E+06 | 2.73E+06 | 5.09E+06 | 2.91E+07 | 3.87E+06 | 5.28E+07 | 2.59E+06 | 3.04E+06 | 0.54 |
| Com_7_neg | Cholic acid | 6.35E+10 | 5.66E+10 | 1.93E+10 | 2.11E+10 | 2.79E+10 | 5.49E+10 | 1.60E+10 | 3.99E+10 | 5.17E+10 | 6.82E+10 | 0.55 |
| Com_800_neg | O-Acetyl-L-homoserine | 5.18E+07 | 5.50E+07 | 2.02E+08 | 1.16E+08 | 6.31E+07 | 2.06E+08 | 9.17E+07 | 1.56E+08 | 9.22E+07 | 1.14E+08 | 0.24 |
| Com_802_pos | LPE 18:1 | 8.71E+07 | 1.34E+08 | 3.40E+08 | 1.59E+08 | 7.26E+08 | 2.83E+08 | 4.00E+08 | 9.09E+08 | 9.61E+07 | 1.45E+08 | 0.69 |
| Com_8034_pos | LPE 22:4 | 8.95E+06 | 4.31E+07 | 1.53E+06 | 2.03E+06 | 1.98E+07 | 1.61E+07 | 2.07E+07 | 2.78E+07 | 1.18E+06 | 1.01E+06 | 0.88 |
| Com_8035_pos | ACar 12:0 | 1.62E+06 | 5.88E+06 | 8.65E+06 | 1.55E+06 | 3.37E+06 | 4.36E+07 | 1.44E+06 | 1.21E+07 | 1.31E+06 | 1.82E+06 | 0.72 |
| Com_8054_neg | trans-Petroselinic acid | 1.39E+06 | 3.78E+06 | 1.86E+06 | 5.27E+06 | 4.52E+06 | 9.98E+05 | 2.56E+06 | 3.44E+06 | 8.10E+06 | 5.76E+06 | 0.80 |
| Com_8078_neg | Homovanillic acid | 2.10E+06 | 2.24E+06 | 3.82E+06 | 3.39E+06 | 2.04E+06 | 5.10E+06 | 1.91E+07 | 3.26E+06 | 2.78E+06 | 4.00E+06 | 0.13 |
| Com_8089_pos | N,N-Dimethyldecylamine N-oxide | 1.07E+07 | 3.24E+07 | 4.08E+06 | 1.76E+07 | 8.87E+06 | 4.32E+07 | 1.84E+07 | 1.35E+07 | 4.49E+06 | 1.95E+07 | 0.58 |
| Com_8101_pos | 3-(3,4-Dihydroxyphenyl)-2-Methylalanine | 4.45E+07 | 4.26E+07 | 2.68E+06 | 1.89E+07 | 2.99E+06 | 3.44E+06 | 3.67E+06 | 2.23E+06 | 4.11E+06 | 1.19E+07 | 0.17 |
| Com_8123_pos | Homogentisic Acid | 1.50E+07 | 1.59E+07 | 1.52E+07 | 1.79E+07 | 1.36E+07 | 4.29E+07 | 2.50E+07 | 2.43E+07 | 1.59E+07 | 2.08E+07 | 0.05 |
| Com_8135_neg | LPE 18:4 | 2.61E+06 | 8.16E+06 | 2.89E+06 | 1.81E+06 | 5.74E+06 | 8.80E+05 | 8.66E+05 | 1.83E+06 | 1.18E+06 | 1.55E+06 | 0.01 |
| Com_8148_neg | 1,5,8-Trihydroxy-9-oxo-9H-xanthen-3-yl beta-D-glucopyranoside | 7.48E+05 | 3.85E+05 | 1.54E+07 | 2.79E+06 | 9.49E+06 | 1.17E+06 | 4.79E+06 | 2.09E+06 | 9.96E+05 | 3.12E+06 | 0.77 |
| Com_814_pos | Nicotinamide N-oxide | 6.27E+07 | 1.01E+08 | 3.36E+07 | 9.46E+07 | 2.80E+08 | 3.38E+08 | 4.04E+07 | 8.94E+08 | 2.78E+07 | 9.31E+07 | 0.66 |
| Com_8161_neg | LPA 16:1 | 1.24E+06 | 5.78E+06 | 1.83E+06 | 2.16E+06 | 5.56E+06 | 8.56E+06 | 2.22E+06 | 1.38E+07 | 1.53E+06 | 1.78E+06 | 0.60 |
| Com_8174_pos | N-Acetyl-D-lactosamine | 1.48E+07 | 2.76E+07 | 1.37E+07 | 3.44E+07 | 1.67E+07 | 3.20E+07 | 3.37E+07 | 2.28E+07 | 1.53E+07 | 3.13E+07 | 0.30 |
| Com_81_pos | L-(+)-Citrulline | 2.60E+09 | 3.15E+09 | 4.26E+09 | 7.29E+09 | 3.42E+09 | 3.08E+09 | 6.42E+09 | 3.44E+09 | 4.46E+09 | 9.36E+09 | 0.41 |
| Com_8213_neg | 8-Isoprostaglandin F1α | 1.10E+07 | 4.68E+06 | 3.69E+06 | 5.55E+06 | 6.26E+06 | 7.75E+06 | 6.62E+06 | 3.82E+06 | 4.84E+06 | 6.57E+06 | 0.96 |
| Com_8222_pos | 2-(3,4-dimethoxyphenyl)ethanamine | 3.12E+06 | 2.71E+06 | 2.48E+06 | 3.13E+06 | 2.51E+07 | 1.29E+07 | 1.32E+07 | 5.06E+07 | 1.71E+06 | 1.78E+06 | 0.50 |
| Com_822_pos | DL-Norvaline | 5.16E+08 | 3.45E+08 | 5.15E+08 | 4.06E+08 | 1.42E+08 | 7.41E+08 | 2.24E+08 | 4.81E+08 | 2.47E+08 | 4.61E+08 | 0.75 |
| Com_8253_pos | (1E,4Z,6E)-5-hydroxy-1,7-bis(4-hydroxyphenyl)hepta-1,4,6-trien-3-one | 4.33E+07 | 3.76E+07 | 2.03E+06 | 4.62E+07 | 1.31E+07 | 7.64E+06 | 2.10E+07 | 3.25E+06 | 3.64E+07 | 1.45E+07 | 0.60 |
| Com_828_pos | 3-(2-Hydroxyethyl)indole | 2.02E+07 | 2.04E+07 | 9.21E+06 | 9.73E+06 | 7.07E+08 | 1.02E+07 | 1.89E+07 | 1.06E+07 | 7.77E+06 | 1.79E+07 | 0.33 |
| Com_829_neg | Daidzein | 1.71E+05 | 3.33E+06 | 5.88E+06 | 6.91E+06 | 4.87E+07 | 1.65E+07 | 8.00E+06 | 3.20E+08 | 1.28E+05 | 2.30E+05 | 0.99 |
| Com_829_pos | Norsufentanil | 1.19E+07 | 6.87E+07 | 1.41E+07 | 3.99E+06 | 8.43E+07 | 1.25E+08 | 2.45E+07 | 8.79E+08 | 2.95E+06 | 1.54E+07 | 0.56 |
| Com_82_neg | Hydrocinnamic acid | 2.89E+09 | 3.10E+09 | 1.03E+09 | 2.16E+08 | 2.89E+09 | 1.91E+08 | 6.89E+08 | 3.97E+08 | 3.36E+08 | 3.67E+08 | 0.05 |
| Com_8301_neg | Noradrenaline | 6.89E+06 | 6.65E+06 | 6.22E+06 | 3.66E+06 | 1.02E+07 | 5.15E+06 | 6.28E+06 | 6.94E+06 | 7.71E+06 | 6.95E+06 | 0.90 |
| Com_835_neg | 1-Methylhistidine | 4.94E+07 | 9.88E+07 | 4.14E+07 | 2.80E+08 | 5.58E+07 | 6.68E+07 | 5.96E+07 | 5.44E+07 | 3.74E+07 | 2.54E+08 | 0.86 |
| Com_8363_neg | 4-Hydroxy-3- methoxyphenylglycol sulfate | 3.48E+05 | 1.19E+06 | 3.44E+05 | 3.24E+06 | 5.09E+06 | 4.37E+06 | 5.95E+06 | 1.34E+07 | 3.46E+06 | 4.34E+06 | 0.05 |
| Com_8367_pos | N-Carbamoyl-L-aspartate | 1.34E+06 | 1.26E+06 | 5.73E+06 | 4.50E+07 | 2.64E+06 | 4.58E+06 | 1.96E+07 | 2.63E+06 | 9.16E+06 | 5.10E+06 | 0.56 |
| Com_8375_pos | Benzoic acid | 7.72E+06 | 6.10E+06 | 8.95E+06 | 2.67E+07 | 7.52E+06 | 2.73E+07 | 5.05E+07 | 2.36E+07 | 4.21E+07 | 3.31E+07 | 0.01 |
| Com_8379_neg | Aflatoxin G2 | 5.04E+06 | 6.82E+06 | 9.53E+06 | 5.04E+06 | 5.40E+06 | 2.29E+06 | 7.08E+06 | 3.52E+05 | 7.60E+06 | 6.61E+06 | 0.31 |
| Com_8384_pos | Kahweol | 2.50E+07 | 1.81E+07 | 1.62E+07 | 4.48E+07 | 7.42E+06 | 1.35E+07 | 1.51E+07 | 6.40E+06 | 2.98E+07 | 2.32E+07 | 0.63 |
| Com_8388_pos | 5-hydroxy-6,7-dimethoxy-2-phenyl-4H-chromen-4-one | 3.65E+07 | 2.52E+07 | 4.51E+07 | 2.65E+07 | 2.03E+07 | 2.64E+07 | 2.76E+07 | 1.66E+07 | 4.19E+07 | 2.65E+07 | 0.63 |
| Com_8392_pos | 5,6-dihydroxyindole | 2.53E+07 | 7.00E+06 | 2.21E+07 | 1.98E+07 | 1.77E+07 | 2.86E+07 | 5.95E+07 | 1.90E+07 | 2.24E+07 | 2.42E+07 | 0.13 |
| Com_8403_pos | N-Acetyl-L-histidine | 2.94E+07 | 2.10E+07 | 2.11E+07 | 1.50E+07 | 3.90E+07 | 1.96E+07 | 3.48E+07 | 2.27E+07 | 2.20E+07 | 3.04E+07 | 0.77 |
| Com_8405_pos | N1-(4-methylidene-3-thia-1-azaspiro[4.5]dec-2-yliden)-2-chloroaniline | 1.07E+07 | 1.74E+07 | 4.00E+06 | 8.42E+06 | 9.45E+06 | 2.12E+07 | 3.04E+07 | 4.86E+07 | 5.35E+06 | 6.72E+06 | 0.27 |
| Com_8406_pos | L-(+)-Arginine | 1.28E+07 | 3.51E+07 | 9.06E+06 | 1.26E+07 | 1.52E+07 | 4.05E+07 | 9.67E+06 | 2.71E+07 | 5.04E+06 | 2.47E+07 | 0.82 |
| Com_842_neg | N-Acetylaspartic acid | 1.18E+08 | 1.22E+08 | 3.44E+08 | 1.17E+08 | 5.93E+07 | 1.34E+08 | 2.60E+08 | 3.76E+07 | 1.73E+08 | 2.22E+08 | 0.87 |
| Com_8447_pos | Phenethyl isothiocyanate | 9.49E+05 | 8.63E+05 | 1.80E+06 | 2.66E+06 | 5.66E+06 | 6.98E+06 | 1.69E+07 | 4.82E+07 | 5.51E+06 | 2.08E+06 | 0.04 |
| Com_8451_pos | 3-[(5-nitropyridin-2-yl)oxy]-1H-indazole | 6.14E+06 | 6.86E+06 | 5.78E+06 | 1.77E+07 | 3.87E+07 | 1.90E+07 | 4.05E+07 | 3.87E+06 | 1.01E+07 | 1.26E+07 | 0.77 |
| Com_8458_pos | ACar 12:1 | 1.63E+06 | 1.39E+06 | 4.87E+07 | 2.06E+06 | 1.44E+06 | 1.39E+07 | 9.70E+05 | 2.35E+06 | 9.07E+05 | 1.79E+06 | 0.67 |
| Com_8460_pos | 1-Aminocyclohexanecarboxylic acid | 1.22E+07 | 1.62E+07 | 1.98E+07 | 1.44E+07 | 1.26E+07 | 1.11E+07 | 1.94E+07 | 9.88E+06 | 4.14E+07 | 2.12E+07 | 0.51 |
| Com_8482_pos | (1E,4E)-1,5-bis(4-methoxyphenyl)penta-1,4-dien-3-one | 2.99E+07 | 2.46E+07 | 3.72E+07 | 2.88E+07 | 2.64E+07 | 1.30E+07 | 2.19E+07 | 2.46E+07 | 2.73E+07 | 4.67E+07 | 0.47 |
| Com_8483_pos | Choline Chloride | 2.99E+07 | 2.46E+07 | 3.72E+07 | 2.88E+07 | 2.64E+07 | 1.30E+07 | 2.19E+07 | 2.46E+07 | 2.73E+07 | 4.67E+07 | 0.47 |
| Com_8496_neg | gamma-Glutamylglutamic acid | 3.06E+06 | 5.78E+06 | 4.28E+06 | 7.05E+06 | 9.36E+06 | 4.80E+06 | 6.01E+06 | 1.30E+07 | 3.85E+06 | 8.83E+06 | 0.54 |
| Com_8501_pos | 1-(4-Methoxyphenyl)-2-propanone | 2.17E+07 | 2.08E+07 | 2.44E+07 | 1.81E+07 | 2.39E+07 | 2.60E+07 | 3.28E+07 | 2.02E+07 | 4.11E+07 | 2.09E+07 | 0.18 |
| Com_8515_neg | Spiculisporic Acid | 7.21E+06 | 7.56E+06 | 7.24E+06 | 7.33E+06 | 5.50E+06 | 2.10E+06 | 4.39E+06 | 1.97E+06 | 7.37E+06 | 6.80E+06 | 0.11 |
| Com_8519_pos | 1,4-Dihydro-1-Methyl-4-Oxo-3-Pyridinecarboxamide | 1.02E+07 | 1.06E+07 | 5.99E+06 | 2.30E+07 | 3.36E+07 | 3.96E+07 | 2.83E+07 | 4.76E+07 | 2.03E+07 | 2.31E+07 | 0.07 |
| Com_852_neg | Verbascose | 1.53E+07 | 3.93E+07 | 2.42E+07 | 8.28E+06 | 2.58E+08 | 6.21E+07 | 9.02E+07 | 1.89E+08 | 7.49E+06 | 1.50E+07 | 0.75 |
| Com_8535_neg | LPG 20:5 | 5.46E+06 | 4.89E+06 | 6.32E+05 | 1.53E+06 | 6.97E+06 | 5.54E+05 | 1.15E+06 | 1.29E+07 | 4.76E+05 | 1.11E+06 | 0.35 |
| Com_8548_pos | 2'-Deoxyguanosine | 9.44E+06 | 3.07E+07 | 9.09E+06 | 1.06E+07 | 1.83E+07 | 1.36E+07 | 5.46E+06 | 1.51E+07 | 1.56E+07 | 4.62E+07 | 0.83 |
| Com_8567_pos | Androsterone | 2.28E+07 | 2.51E+07 | 2.30E+07 | 2.02E+07 | 2.17E+07 | 3.94E+07 | 2.14E+07 | 1.93E+07 | 3.56E+07 | 4.17E+07 | 0.15 |
| Com_8576_pos | 1-Methyladenosine | 1.78E+07 | 1.69E+07 | 3.01E+07 | 2.11E+07 | 1.98E+07 | 3.94E+07 | 1.64E+07 | 3.40E+07 | 2.36E+07 | 4.49E+07 | 0.13 |
| Com_8582_pos | 4-[1-(acetyloxy)prop-2-en-1-yl]-2-methoxyphenyl 2-methylpropanoate | 7.22E+06 | 6.92E+06 | 3.00E+07 | 9.33E+06 | 8.95E+06 | 3.93E+07 | 1.21E+07 | 3.41E+07 | 2.66E+06 | 5.46E+06 | 0.84 |
| Com_8592_neg | 3-Hydroxydecanoic acid | 3.79E+06 | 7.45E+06 | 3.50E+06 | 2.30E+06 | 6.63E+06 | 2.62E+06 | 4.54E+06 | 3.43E+06 | 1.72E+06 | 3.08E+06 | 0.19 |
| Com_860_pos | Sebacic acid | 3.46E+08 | 4.71E+08 | 5.03E+08 | 3.77E+08 | 3.05E+08 | 2.67E+08 | 4.54E+08 | 6.53E+07 | 7.20E+08 | 5.47E+08 | 0.64 |
| Com_8616_pos | 1,7-bis(3,4-dihydroxyphenyl)heptan-3-one | 5.37E+06 | 4.52E+06 | 7.47E+06 | 8.46E+06 | 3.77E+07 | 1.08E+07 | 8.29E+06 | 9.95E+06 | 8.08E+06 | 8.33E+06 | 0.99 |
| Com_8628_neg | 2-(1H-1,2,3-benzotriazol-1-yl)-N-(2,3-dihydro-1H-inden-2-yl)acetamide | 3.38E+06 | 4.93E+06 | 1.08E+06 | 3.07E+06 | 3.16E+05 | 7.82E+06 | 1.11E+06 | 7.62E+05 | 8.20E+05 | 1.86E+06 | 0.87 |
| Com_8679_neg | Pantetheine | 6.12E+05 | 6.15E+05 | 7.68E+05 | 1.07E+06 | 4.58E+06 | 7.75E+06 | 3.57E+05 | 1.20E+06 | 1.59E+06 | 1.57E+06 | 0.58 |
| Com_8683_neg | 3'-Hydroxystanozolol | 2.32E+06 | 7.19E+06 | 1.22E+06 | 7.33E+06 | 1.79E+06 | 6.32E+06 | 3.79E+06 | 2.75E+06 | 8.90E+05 | 1.25E+07 | 0.73 |
| Com_868_pos | Acetyl-L-carnitine | 1.71E+08 | 1.37E+08 | 8.38E+08 | 9.52E+07 | 1.22E+08 | 2.07E+08 | 5.37E+07 | 3.06E+08 | 1.82E+08 | 5.97E+07 | 0.51 |
| Com_8691_pos | 19(R)-HETE | 4.93E+07 | 2.05E+07 | 1.56E+07 | 2.61E+07 | 1.82E+07 | 3.04E+07 | 2.53E+07 | 2.68E+07 | 2.51E+07 | 3.79E+07 | 0.41 |
| Com_8692_pos | Phylloquinone | 6.00E+06 | 6.47E+06 | 6.44E+06 | 4.25E+07 | 2.53E+07 | 1.38E+07 | 2.52E+07 | 2.42E+07 | 2.17E+07 | 1.40E+07 | 0.35 |
| Com_870_neg | Azelaic acid | 2.05E+08 | 1.58E+08 | 2.83E+08 | 1.28E+08 | 1.48E+08 | 1.32E+08 | 2.49E+08 | 7.89E+07 | 1.69E+08 | 1.45E+08 | 0.41 |
| Com_8740_pos | 5-(6-hydroxy-6-methyloctyl)-2,5-dihydrofuran-2-one | 4.10E+07 | 3.78E+07 | 6.46E+06 | 1.36E+07 | 3.47E+07 | 2.16E+07 | 2.43E+07 | 1.22E+07 | 3.07E+07 | 2.65E+07 | 0.96 |
| Com_8746_pos | 3,3,5-trimethyl-3H,11H-pyrano[3,2-a]carbazole | 4.87E+07 | 2.06E+07 | 5.49E+06 | 1.82E+07 | 2.17E+07 | 3.62E+07 | 1.10E+07 | 1.12E+06 | 1.67E+07 | 1.95E+07 | 0.46 |
| Com_876_pos | Ferulic acid | 2.25E+08 | 3.55E+08 | 1.84E+08 | 1.03E+08 | 1.91E+08 | 4.33E+08 | 4.53E+08 | 8.19E+08 | 8.50E+07 | 2.25E+08 | 0.32 |
| Com_8773_neg | 2-(3-methylbenzoyl)benzoic acid | 4.18E+06 | 4.79E+06 | 5.81E+06 | 3.77E+06 | 3.58E+06 | 7.63E+06 | 6.58E+06 | 5.20E+06 | 3.87E+06 | 2.46E+06 | 0.69 |
| Com_8796_pos | 4-(2-furyl)-2-oxo-6-(2-thienyl)-1,2-dihydro-3-pyridinecarbonitrile | 1.77E+07 | 4.99E+06 | 4.60E+07 | 3.66E+07 | 1.36E+07 | 1.22E+07 | 4.30E+07 | 1.40E+07 | 2.40E+07 | 2.22E+07 | 0.78 |
| Com_882_neg | Adenine | 8.39E+07 | 1.70E+08 | 1.32E+08 | 1.96E+08 | 3.23E+07 | 7.85E+07 | 2.27E+08 | 1.45E+08 | 1.28E+07 | 6.00E+07 | 0.56 |
| Com_882_pos | gamma-Glutamyltyrosine | 2.64E+07 | 5.88E+07 | 6.44E+07 | 1.63E+08 | 9.66E+07 | 6.80E+08 | 4.03E+07 | 6.64E+08 | 3.86E+07 | 1.08E+08 | 0.32 |
| Com_8846_pos | Spectinomycin | 2.98E+07 | 3.73E+07 | 2.76E+07 | 2.08E+07 | 2.62E+07 | 2.32E+07 | 2.58E+07 | 2.18E+07 | 1.87E+07 | 2.69E+07 | 0.15 |
| Com_8871_neg | PC (2:0/13:1) | 4.97E+06 | 3.85E+06 | 1.98E+06 | 1.08E+07 | 9.63E+06 | 8.85E+05 | 1.55E+06 | 4.21E+06 | 3.68E+06 | 1.83E+06 | 0.06 |
| Com_8879_pos | ACar 13:1 | 1.60E+06 | 1.38E+06 | 4.54E+07 | 1.18E+06 | 1.60E+06 | 7.57E+06 | 1.38E+06 | 1.78E+06 | 1.53E+06 | 1.37E+06 | 0.70 |
| Com_8893_neg | 2-(2-carboxy-2-methylpropyl)-4,6-dimethylbenzoic acid | 3.69E+06 | 3.15E+06 | 5.39E+05 | 7.96E+05 | 1.01E+07 | 3.62E+06 | 8.92E+05 | 5.81E+05 | 8.32E+05 | 8.99E+05 | 0.29 |
| Com_889_pos | N3,N4-Dimethyl-L-arginine | 2.31E+08 | 2.30E+08 | 3.79E+08 | 7.44E+08 | 3.59E+08 | 3.13E+08 | 6.76E+08 | 7.37E+08 | 2.16E+08 | 4.17E+08 | 0.56 |
| Com_894_neg | Taurochenodeoxycholic acid | 2.30E+08 | 4.09E+07 | 4.78E+06 | 9.31E+06 | 7.42E+07 | 3.82E+07 | 3.74E+06 | 5.52E+07 | 1.45E+08 | 8.75E+06 | 0.82 |
| Com_8951_pos | Metanephrine | 1.20E+06 | 1.18E+06 | 9.15E+05 | 3.55E+06 | 9.91E+05 | 2.63E+06 | 2.05E+07 | 4.42E+06 | 3.82E+07 | 2.46E+07 | 0.01 |
| Com_8952_pos | Fexofenadine | 2.01E+06 | 9.50E+06 | 3.46E+07 | 4.77E+06 | 1.57E+07 | 9.94E+06 | 3.36E+07 | 4.43E+07 | 3.97E+06 | 5.72E+06 | 0.59 |
| Com_8966_pos | N-P-Coumaroyl Spermidine | 3.05E+06 | 2.14E+06 | 1.71E+06 | 1.59E+06 | 3.96E+06 | 3.69E+07 | 5.13E+06 | 5.14E+06 | 1.71E+06 | 2.02E+06 | 0.24 |
| Com_8971_neg | (+/-)-Equol | 7.96E+06 | 5.15E+06 | 5.84E+06 | 5.00E+06 | 6.20E+06 | 7.31E+06 | 4.42E+06 | 4.28E+06 | 3.96E+06 | 5.99E+06 | 0.29 |
| Com_904_pos | Prolylleucine | 3.33E+07 | 1.35E+08 | 1.93E+07 | 4.43E+07 | 3.76E+08 | 3.61E+08 | 9.45E+08 | 8.00E+08 | 2.23E+07 | 1.02E+08 | 0.21 |
| Com_9053_neg | Piceatannol | 8.58E+06 | 4.67E+06 | 3.38E+06 | 4.37E+06 | 8.05E+06 | 7.19E+06 | 4.34E+06 | 4.82E+06 | 2.06E+06 | 3.24E+06 | 0.29 |
| Com_9072_pos | LPE 17:1 | 1.84E+07 | 3.13E+07 | 2.30E+06 | 1.66E+07 | 3.50E+07 | 1.77E+07 | 1.32E+07 | 1.50E+07 | 2.27E+07 | 1.92E+07 | 0.80 |
| Com_9074_pos | Serotonin | 2.80E+06 | 3.56E+06 | 1.83E+06 | 1.44E+06 | 3.49E+07 | 1.79E+06 | 2.87E+06 | 2.74E+06 | 1.57E+06 | 1.79E+06 | 0.34 |
| Com_9113_pos | SKK | 6.07E+06 | 3.61E+06 | 5.38E+06 | 4.59E+06 | 3.47E+07 | 5.13E+06 | 1.30E+07 | 2.35E+07 | 2.46E+07 | 4.32E+06 | 0.45 |
| Com_911_neg | FAHFA (18:2/20:4) | 1.02E+08 | 9.00E+07 | 5.38E+07 | 1.44E+08 | 1.23E+08 | 7.32E+06 | 1.45E+08 | 8.66E+07 | 1.59E+08 | 6.07E+07 | 0.47 |
| Com_911_pos | Uric acid | 2.68E+08 | 2.18E+08 | 6.34E+08 | 2.95E+07 | 6.40E+08 | 6.90E+07 | 4.61E+07 | 4.10E+08 | 3.07E+07 | 3.71E+07 | 0.13 |
| Com_9129_pos | 3-(3,4,5-trimethoxyphenyl)propanoic acid | 1.85E+07 | 2.86E+07 | 7.49E+06 | 1.95E+07 | 3.45E+07 | 2.34E+07 | 4.82E+07 | 3.10E+07 | 1.83E+07 | 1.38E+07 | 0.50 |
| Com_9133_pos | α-Aspartylphenylalanine | 3.48E+06 | 1.28E+07 | 2.28E+06 | 3.75E+06 | 1.02E+07 | 3.59E+07 | 6.23E+06 | 3.66E+07 | 2.71E+06 | 4.33E+06 | 0.35 |
| Com_9138_neg | FAHFA (16:0/18:0) | 1.50E+06 | 2.27E+06 | 9.19E+06 | 1.02E+07 | 1.29E+06 | 5.12E+05 | 4.18E+06 | 1.84E+06 | 4.41E+06 | 2.12E+06 | 0.44 |
| Com_9168_pos | Propionyl-L-carnitine | 9.61E+06 | 1.10E+07 | 4.32E+07 | 9.09E+06 | 2.60E+07 | 1.01E+07 | 1.23E+07 | 1.28E+07 | 1.11E+07 | 1.05E+07 | 0.32 |
| Com_9186_pos | N-glycyl-L-proline | 1.56E+07 | 1.11E+07 | 7.52E+06 | 2.00E+07 | 3.42E+07 | 2.35E+07 | 2.02E+07 | 3.88E+07 | 1.47E+07 | 2.84E+07 | 0.20 |
| Com_918_neg | Calcitriol | 1.29E+08 | 1.33E+08 | 2.46E+07 | 2.27E+08 | 1.23E+08 | 1.69E+08 | 1.80E+08 | 7.69E+07 | 1.26E+08 | 1.44E+08 | 0.56 |
| Com_918_pos | (-)-Camphor | 4.39E+08 | 6.48E+08 | 8.00E+08 | 4.19E+08 | 5.00E+08 | 2.98E+08 | 6.97E+08 | 5.97E+08 | 3.80E+08 | 3.88E+08 | 0.37 |
| Com_9190_neg | 4-Butylresorcinol | 2.76E+06 | 3.66E+06 | 2.59E+06 | 2.97E+06 | 3.78E+06 | 7.02E+06 | 7.69E+06 | 6.14E+06 | 1.47E+06 | 4.68E+06 | 0.25 |
| Com_9211_pos | methyl 2-[(2-acetyl-3-oxo-1-butenyl)amino]acetate | 2.76E+07 | 3.50E+07 | 2.61E+07 | 1.63E+07 | 2.73E+07 | 3.00E+07 | 2.65E+07 | 1.83E+07 | 2.96E+07 | 2.35E+07 | 0.90 |
| Com_9234_neg | N-(2-oxo-3-azepanyl)benzenesulfonamide | 2.89E+06 | 6.60E+06 | 3.35E+06 | 1.54E+06 | 5.34E+06 | 3.24E+06 | 2.99E+06 | 2.62E+06 | 2.27E+06 | 1.06E+07 | 0.94 |
| Com_9238_pos | N2,N2-Dimethylguanosine | 9.26E+06 | 1.50E+07 | 2.10E+07 | 3.88E+07 | 1.97E+07 | 7.77E+06 | 4.37E+07 | 3.60E+07 | 5.33E+06 | 2.42E+07 | 0.89 |
| Com_923_pos | octadec-9-ynoic acid | 4.43E+08 | 3.12E+08 | 5.55E+08 | 5.74E+08 | 2.92E+08 | 6.56E+08 | 4.93E+08 | 3.94E+08 | 2.51E+08 | 5.18E+08 | 0.82 |
| Com_9248_pos | 2-methyl-2,3,4,5-tetrahydro-1,5-benzoxazepin-4-one | 1.15E+07 | 9.29E+06 | 4.27E+07 | 7.15E+06 | 1.34E+07 | 1.77E+07 | 9.53E+06 | 2.18E+07 | 5.53E+06 | 6.11E+06 | 0.56 |
| Com_9261_neg | L-Carnitine | 3.92E+06 | 5.79E+06 | 9.62E+06 | 3.13E+06 | 9.42E+06 | 5.11E+06 | 5.82E+06 | 6.18E+06 | 3.24E+06 | 4.42E+06 | 0.51 |
| Com_928_pos | LPE 14:0 | 1.04E+08 | 6.45E+08 | 2.04E+07 | 6.02E+07 | 3.12E+08 | 1.06E+08 | 4.21E+07 | 4.35E+08 | 3.10E+07 | 5.21E+07 | 0.60 |
| Com_9291_neg | 2,5-Dimethylphenol | 3.63E+06 | 4.34E+06 | 1.23E+07 | 6.43E+05 | 3.46E+06 | 7.85E+05 | 1.11E+06 | 6.89E+05 | 2.14E+06 | 1.13E+06 | 0.07 |
| Com_9292_pos | GPK | 1.46E+07 | 2.33E+07 | 1.28E+07 | 3.85E+07 | 2.01E+07 | 7.30E+06 | 2.01E+07 | 2.24E+07 | 5.79E+06 | 1.72E+07 | 0.21 |
| Com_929_pos | Jasmone | 1.15E+08 | 1.00E+08 | 8.45E+07 | 7.15E+08 | 1.58E+08 | 1.02E+08 | 1.95E+08 | 1.54E+08 | 1.45E+08 | 1.14E+08 | 0.72 |
| Com_92_pos | D-(+)-Proline | 2.39E+09 | 2.69E+09 | 6.19E+09 | 4.69E+09 | 4.88E+09 | 3.67E+09 | 6.54E+09 | 7.95E+09 | 3.73E+09 | 5.27E+09 | 0.27 |
| Com_9301_pos | (5E)-7-methylidene-10-oxo-4-(propan-2-yl)undec-5-enoic acid | 3.77E+07 | 3.46E+07 | 2.16E+07 | 2.51E+07 | 2.49E+07 | 3.46E+07 | 3.11E+07 | 3.46E+07 | 2.44E+07 | 3.06E+07 | 0.48 |
| Com_9306_pos | Ureidoisobutyric Acid | 5.73E+06 | 1.13E+07 | 2.49E+07 | 3.91E+06 | 1.34E+07 | 3.49E+07 | 5.12E+06 | 7.13E+06 | 3.28E+06 | 7.35E+06 | 0.71 |
| Com_930_pos | Phenylpyruvic acid | 1.75E+07 | 5.96E+07 | 7.86E+08 | 5.66E+06 | 1.28E+08 | 1.66E+07 | 9.66E+06 | 1.08E+07 | 1.18E+07 | 1.24E+07 | 0.14 |
| Com_9318_neg | 1-(2,4-dihydroxyphenyl)-2-(3,5-dimethoxyphenyl)propan-1-one | 8.13E+06 | 6.50E+06 | 4.77E+05 | 1.56E+06 | 1.52E+06 | 7.72E+05 | 4.53E+05 | 2.42E+05 | 4.09E+06 | 3.98E+06 | 0.36 |
| Com_931_neg | 4-Methoxycinnamic Acid | 2.18E+08 | 4.62E+07 | 1.49E+08 | 8.36E+07 | 1.38E+08 | 5.22E+07 | 2.22E+08 | 4.60E+07 | 1.18E+08 | 8.45E+07 | 0.56 |
| Com_932_neg | LPI 18:2 | 1.02E+06 | 1.58E+08 | 1.14E+06 | 1.23E+06 | 8.36E+06 | 8.85E+05 | 1.74E+06 | 5.21E+06 | 9.02E+05 | 1.09E+06 | 0.33 |
| Com_9341_pos | L-Hydroxylysine | 9.18E+06 | 1.06E+07 | 5.86E+06 | 9.27E+06 | 1.91E+07 | 1.77E+07 | 9.65E+06 | 4.16E+07 | 9.96E+06 | 1.36E+07 | 0.21 |
| Com_934_pos | (5-L-Glutamyl)-L-Amino Acid | 5.00E+07 | 6.03E+07 | 8.58E+07 | 3.23E+08 | 6.85E+07 | 4.78E+08 | 1.22E+08 | 7.71E+08 | 7.73E+07 | 1.83E+08 | 0.12 |
| Com_9357_pos | LPE 19:1 | 4.35E+06 | 3.43E+07 | 7.63E+06 | 6.28E+06 | 6.02E+06 | 1.76E+07 | 5.68E+06 | 3.40E+07 | 3.50E+06 | 4.26E+06 | 0.96 |
| Com_9370_pos | PC (14:1e/3:0) | 1.77E+06 | 2.45E+07 | 1.54E+06 | 1.52E+06 | 3.33E+07 | 1.29E+07 | 2.45E+06 | 2.50E+07 | 8.97E+05 | 1.11E+06 | 0.77 |
| Com_9389_pos | (3,4-Dimethoxyphenyl)acetic acid | 2.54E+07 | 3.41E+07 | 1.29E+07 | 2.53E+07 | 2.03E+07 | 2.61E+07 | 1.81E+07 | 1.95E+07 | 3.44E+07 | 2.69E+07 | 0.70 |
| Com_938_pos | Lysopg 18:1 | 8.18E+08 | 4.65E+08 | 1.24E+08 | 4.99E+08 | 4.07E+08 | 1.85E+08 | 3.42E+08 | 1.29E+08 | 3.89E+08 | 2.95E+08 | 0.26 |
| Com_9398_neg | PG (3:0/18:0) | 5.67E+05 | 6.42E+06 | 9.16E+05 | 8.72E+05 | 2.56E+06 | 4.20E+05 | 1.34E+06 | 7.26E+05 | 5.40E+05 | 8.10E+05 | 0.18 |
| Com_9409_pos | Dihydrothymine | 5.94E+06 | 1.19E+07 | 2.45E+07 | 4.52E+06 | 1.42E+07 | 3.44E+07 | 5.10E+06 | 7.16E+06 | 3.78E+06 | 6.95E+06 | 0.63 |
| Com_941_neg | Orsellinic acid ethyl ester | 2.39E+06 | 3.89E+06 | 8.84E+06 | 5.45E+07 | 1.41E+07 | 2.15E+07 | 2.08E+08 | 1.26E+07 | 8.12E+07 | 2.66E+08 | 0.04 |
| Com_9427_neg | Gibberellin A7 | 8.79E+06 | 7.50E+05 | 6.59E+05 | 4.48E+05 | 3.30E+05 | 5.56E+05 | 1.37E+07 | 2.86E+05 | 4.11E+05 | 4.01E+05 | 0.90 |
| Com_9440_pos | Methanandamide | 1.38E+06 | 2.68E+06 | 4.14E+07 | 1.19E+06 | 1.96E+06 | 1.42E+06 | 1.82E+06 | 4.93E+06 | 2.88E+07 | 1.08E+06 | 0.98 |
| Com_9443_neg | 5-OxoETE | 5.93E+06 | 6.01E+06 | 9.66E+06 | 8.01E+06 | 9.13E+06 | 5.64E+06 | 1.11E+07 | 6.89E+06 | 5.30E+06 | 8.62E+06 | 0.78 |
| Com_944_pos | Ergocalciferol | 6.09E+07 | 3.59E+08 | 1.15E+07 | 1.31E+07 | 9.07E+06 | 1.36E+07 | 9.27E+07 | 1.05E+07 | 8.61E+07 | 7.43E+08 | 0.54 |
| Com_9456_pos | Folinic acid | 7.31E+05 | 7.20E+06 | 7.51E+05 | 1.51E+06 | 7.55E+06 | 3.11E+07 | 1.35E+07 | 4.09E+07 | 8.87E+05 | 4.71E+06 | 0.13 |
| Com_9466_neg | Lysopc 14:0 | 5.37E+06 | 1.31E+06 | 3.62E+05 | 3.00E+05 | 8.55E+05 | 1.30E+06 | 6.65E+05 | 1.09E+07 | 3.65E+05 | 2.56E+05 | 0.95 |
| Com_9469_neg | L-Methionine sulfone | 1.96E+06 | 2.21E+06 | 2.16E+06 | 2.21E+06 | 9.09E+06 | 3.96E+06 | 3.13E+06 | 5.84E+06 | 2.05E+06 | 1.71E+06 | 0.88 |
| Com_9473_pos | Methyl cinnamate | 8.77E+06 | 7.94E+06 | 6.53E+06 | 1.49E+07 | 5.21E+06 | 1.53E+07 | 3.41E+07 | 1.58E+07 | 3.51E+07 | 2.95E+07 | 0.00 |
| Com_947_neg | Sucrose | 2.46E+07 | 5.46E+07 | 4.48E+07 | 1.33E+07 | 2.23E+08 | 1.10E+08 | 8.68E+07 | 1.27E+08 | 1.51E+07 | 2.74E+07 | 0.75 |
| Com_9502_pos | Verrucarol | 2.26E+07 | 2.66E+07 | 1.67E+07 | 8.36E+06 | 1.62E+07 | 3.38E+07 | 4.74E+07 | 2.15E+07 | 1.57E+07 | 1.06E+07 | 0.41 |
| Com_9524_pos | 4-{3-[(3,4-dihydroxyphenyl)methyl]-2-methylbutyl}benzene-1,2-diol | 4.30E+07 | 1.65E+07 | 3.60E+06 | 6.37E+06 | 2.24E+07 | 6.79E+06 | 5.07E+06 | 3.18E+06 | 1.27E+07 | 6.61E+06 | 0.19 |
| Com_9528_neg | 1-O-(3,4,5-Trimethoxybenzoyl)-beta-L-galactopyranose | 1.43E+05 | 2.03E+05 | 2.28E+05 | 1.89E+05 | 1.78E+05 | 6.62E+06 | 6.55E+05 | 1.51E+06 | 1.16E+05 | 2.36E+05 | 0.13 |
| Com_952_neg | 4-Anisic acid | 8.46E+07 | 1.24E+07 | 2.60E+07 | 2.74E+07 | 2.38E+07 | 2.97E+07 | 3.58E+08 | 7.79E+07 | 2.99E+07 | 3.21E+07 | 0.23 |
| Com_953_neg | N-Acetylalanine | 3.48E+07 | 6.00E+07 | 1.15E+08 | 5.64E+07 | 1.80E+08 | 6.14E+07 | 1.10E+08 | 2.65E+08 | 3.23E+07 | 5.48E+07 | 0.91 |
| Com_9544_pos | PC (22:6e/2:0) | 4.19E+06 | 1.33E+07 | 2.25E+06 | 2.11E+06 | 1.22E+07 | 1.71E+06 | 6.14E+06 | 4.01E+07 | 1.04E+06 | 1.14E+06 | 0.65 |
| Com_956_neg | 3-(1,1,2,3,3,3-hexafluoropropyl)adamantane-1-carboxylic acid | 8.50E+07 | 1.06E+08 | 1.29E+07 | 1.17E+08 | 1.32E+08 | 9.50E+07 | 5.78E+07 | 1.62E+07 | 1.51E+08 | 1.94E+07 | 0.55 |
| Com_9592_pos | 2-Methoxyestradiol (2-MeOE2) | 3.57E+07 | 3.29E+07 | 2.94E+07 | 1.73E+07 | 2.89E+07 | 1.48E+07 | 1.88E+07 | 1.12E+07 | 2.16E+07 | 2.16E+07 | 0.02 |
| Com_95_pos | Xanthine | 3.19E+09 | 4.98E+09 | 6.73E+09 | 5.94E+09 | 2.57E+09 | 1.67E+09 | 4.93E+09 | 1.56E+09 | 2.39E+09 | 7.53E+09 | 0.32 |
| Com_9601_neg | 5,6-dimethyl-3-[5-(trifluoromethyl)pyridin-2-yl]-1,2,4-triazine | 8.33E+05 | 2.15E+06 | 6.47E+05 | 2.59E+05 | 8.89E+06 | 2.15E+05 | 6.44E+05 | 3.82E+05 | 1.95E+05 | 1.91E+05 | 0.07 |
| Com_9613_neg | 2,3-Dinor-8-epi-prostaglandin F2α | 3.84E+05 | 1.71E+06 | 4.28E+05 | 4.17E+05 | 1.80E+06 | 6.52E+06 | 1.79E+06 | 2.46E+06 | 3.28E+05 | 6.51E+05 | 0.32 |
| Com_9623_pos | 2-Phenylglycine | 1.28E+07 | 1.25E+07 | 2.63E+07 | 2.65E+07 | 1.24E+07 | 7.15E+06 | 1.82E+07 | 9.96E+06 | 3.08E+07 | 3.87E+07 | 0.95 |
| Com_9634_neg | Isoproterenol | 3.95E+06 | 6.16E+06 | 4.95E+06 | 2.39E+06 | 4.21E+06 | 3.09E+06 | 3.76E+06 | 6.70E+05 | 2.64E+06 | 3.19E+06 | 0.15 |
| Com_965_pos | Nicotinamide | 2.35E+07 | 4.64E+07 | 2.37E+07 | 9.60E+07 | 6.01E+07 | 4.12E+08 | 3.30E+08 | 4.74E+08 | 6.45E+08 | 1.05E+08 | 0.00 |
| Com_9666_pos | Astaxanthin | 1.17E+07 | 3.26E+07 | 1.10E+06 | 1.48E+06 | 1.16E+07 | 1.58E+06 | 9.83E+05 | 9.28E+05 | 2.22E+06 | 1.24E+06 | 0.08 |
| Com_9692_pos | α-Lapachone | 3.40E+07 | 3.25E+07 | 1.90E+06 | 8.53E+06 | 3.03E+07 | 1.34E+07 | 1.36E+07 | 4.08E+06 | 1.96E+07 | 1.67E+07 | 0.81 |
| Com_9704_neg | 2'-Deoxycytidine | 1.80E+06 | 2.87E+06 | 4.84E+06 | 1.50E+06 | 1.59E+06 | 3.50E+06 | 3.77E+06 | 2.99E+06 | 5.91E+06 | 1.04E+07 | 0.05 |
| Com_9729_pos | H-Gly-Pro-OH | 1.00E+07 | 1.53E+07 | 3.95E+07 | 1.99E+07 | 1.14E+07 | 1.19E+07 | 1.71E+07 | 1.22E+07 | 2.18E+07 | 2.75E+07 | 0.96 |
| Com_972_neg | 2-Oxobutyric acid | 4.88E+07 | 9.39E+07 | 5.95E+07 | 1.15E+08 | 2.18E+08 | 6.65E+07 | 2.00E+08 | 2.19E+08 | 3.23E+07 | 9.30E+07 | 0.91 |
| Com_9762_pos | 3-hydroxy-1,5-diphenylpentan-1-one | 1.37E+07 | 2.56E+07 | 1.85E+07 | 9.41E+06 | 3.11E+07 | 2.57E+07 | 1.51E+07 | 9.72E+06 | 1.35E+07 | 3.41E+06 | 0.27 |
| Com_9783_pos | Ala-trp | 6.65E+06 | 8.18E+06 | 1.74E+06 | 1.02E+07 | 3.11E+07 | 6.82E+06 | 8.56E+06 | 1.04E+07 | 3.94E+06 | 3.10E+06 | 0.61 |
| Com_9784_neg | 2-Phenylpropionic acid | 8.27E+06 | 5.82E+06 | 3.61E+06 | 2.57E+06 | 5.51E+06 | 6.07E+06 | 3.57E+06 | 1.96E+06 | 2.67E+06 | 2.82E+06 | 0.18 |
| Com_9799_neg | α,α-Trehalose | 5.53E+05 | 7.65E+05 | 8.74E+05 | 4.71E+06 | 8.89E+05 | 2.71E+06 | 5.05E+06 | 4.16E+05 | 5.86E+06 | 4.20E+06 | 0.18 |
| Com_979_neg | Asp-Phe methyl ester | 4.82E+06 | 1.54E+07 | 1.79E+07 | 1.60E+08 | 1.65E+07 | 1.58E+08 | 1.30E+07 | 1.53E+08 | 7.40E+06 | 4.56E+07 | 0.44 |
| Com_97_neg | Phenylacetaldehyde | 1.95E+08 | 5.90E+08 | 1.23E+08 | 3.13E+07 | 3.27E+09 | 3.63E+07 | 4.65E+07 | 1.06E+08 | 1.94E+07 | 2.25E+07 | 0.06 |
| Com_9843_neg | Ascorbyl stearate | 7.15E+05 | 7.53E+05 | 8.87E+05 | 1.20E+06 | 7.29E+05 | 6.28E+06 | 9.52E+05 | 8.98E+05 | 7.13E+05 | 9.60E+05 | 0.34 |
| Com_9848_pos | Deoxycorticosterone | 3.54E+07 | 2.98E+07 | 3.80E+07 | 1.66E+07 | 1.73E+07 | 6.52E+06 | 3.18E+07 | 1.74E+07 | 3.29E+07 | 2.09E+07 | 0.40 |
| Com_986_pos | trans-3-Indoleacrylic acid | 3.44E+07 | 2.44E+07 | 2.03E+06 | 5.73E+08 | 4.25E+07 | 2.21E+08 | 2.79E+08 | 7.31E+08 | 1.96E+07 | 2.73E+07 | 0.30 |
| Com_9873_pos | ACar 18:0 | 9.40E+06 | 2.50E+07 | 1.13E+07 | 1.53E+06 | 2.08E+07 | 3.18E+07 | 1.53E+06 | 3.74E+07 | 2.54E+06 | 3.47E+06 | 0.70 |
| Com_9881_neg | Desthiobiotin | 1.66E+06 | 3.62E+06 | 2.45E+06 | 5.61E+06 | 8.48E+06 | 2.66E+06 | 3.23E+06 | 2.41E+06 | 1.66E+06 | 4.33E+06 | 0.38 |
| Com_9882_pos | N-Formylkynurenine | 4.58E+06 | 6.58E+06 | 5.21E+06 | 8.81E+06 | 1.05E+07 | 9.38E+06 | 9.27E+06 | 3.80E+07 | 4.89E+06 | 6.22E+06 | 0.36 |
| Com_98_pos | L-(-)-Methionine | 2.23E+09 | 4.37E+09 | 2.42E+09 | 4.61E+09 | 3.35E+09 | 3.13E+09 | 4.34E+09 | 6.09E+09 | 2.24E+09 | 7.24E+09 | 0.35 |
| Com_9905_pos | Pleuromutilin | 1.13E+07 | 3.13E+07 | 1.64E+06 | 1.69E+06 | 5.11E+06 | 1.17E+06 | 2.18E+06 | 1.06E+06 | 1.32E+06 | 1.19E+06 | 0.07 |
| Com_9914_pos | Bialaphos | 3.42E+07 | 2.38E+07 | 3.83E+07 | 2.09E+07 | 3.95E+06 | 2.03E+07 | 1.36E+07 | 9.51E+05 | 2.59E+07 | 3.33E+07 | 0.55 |
| Com_9917_neg | 15(R)-Prostaglandin E2 | 8.09E+06 | 1.11E+06 | 1.17E+06 | 6.56E+05 | 1.27E+06 | 6.46E+05 | 1.43E+06 | 4.22E+05 | 1.27E+06 | 8.81E+05 | 0.26 |
