# Supplementary material for: Chronic alcohol-induced dysbiosis of the gut microbiota and gut metabolites impairs sperm quality in mice
Source: Front Microbiol. 2022 Dec 1;13:1042923. doi: 10.3389/fmicb.2022.1042923 (PMC9751024; doi:10.3389/fmicb.2022.1042923)
Supplement: Supplementary file 2 [file Data_Sheet_2.ZIP › supplemental/Supplemental Table 6.docx]

**Supplemental Table 6. Differential enrichment of metabolites between Alcohol-FMT and Control-FMT groups**

| **MapID** | **MapTitle** | **MetaIDs** | **P-value** | |
| --- | --- | --- | --- | --- |
| map00040 | Pentose and glucuronate interconversions | Ribitol; alpha-Ketoglutaric acid | 0.0150 |  |
| map00020 | Citrate cycle (TCA cycle) | Oxaloacetic acid; alpha-Ketoglutaric acid | 0.0287 |  |
| map00740 | Riboflavin metabolism | Ribitol; Riboflavin-5-phosphate | 0.0287 |  |
| map00232 | Caffeine metabolism | 1-Methylxanthine; 1-Methyluric acid | 0.0457 |  |
| map00630 | Glyoxylate and dicarboxylate metabolism | Oxaloacetic acid; alpha-Ketoglutaric acid | 0.0457 |  |
| map05032 | Morphine addiction | Adenosine | 0.0740 |  |
| map04927 | Cortisol synthesis and secretion | Cortisol; Cortodoxone | 0.1116 |  |
| map04934 | Cushing's syndrome | Cortisol; Cortodoxone | 0.1116 |  |
| map00250 | Alanine, aspartate and glutamate metabolism | Oxaloacetic acid; alpha-Ketoglutaric acid | 0.1371 |  |
| map00010 | Glycolysis / Gluconeogenesis | Oxaloacetic acid | 0.1427 |  |
| map00471 | D-Glutamine and D-glutamate metabolism | alpha-Ketoglutaric acid | 0.1427 |  |
| map04022 | cGMP-PKG signaling pathway | Adenosine | 0.1427 |  |
| map00310 | Lysine degradation | N6,N6,N6-Trimethyl-L-lysine; L-Saccharopine | 0.1638 |  |
| map00760 | Nicotinate and nicotinamide metabolism | Trigonelline; Nicotinamide | 0.1638 |  |
| map00053 | Ascorbate and aldarate metabolism | alpha-Ketoglutaric acid | 0.2065 |  |
| map00190 | Oxidative phosphorylation | Riboflavin-5-phosphate | 0.2065 |  |
| map00620 | Pyruvate metabolism | Oxaloacetic acid | 0.2065 |  |
| map04071 | Sphingolipid signaling pathway | Adenosine | 0.2065 |  |
| map04270 | Vascular smooth muscle contraction | Adenosine | 0.2065 |  |
| map05034 | Alcoholism | Adenosine | 0.2065 |  |
| map00340 | Histidine metabolism | Carnosine; alpha-Ketoglutaric acid | 0.2483 |  |
| map00650 | Butanoate metabolism | alpha-Ketoglutaric acid | 0.2657 |  |
| map04960 | Aldosterone-regulated sodium reabsorption | Cortisol | 0.2657 |  |
| map05012 | Parkinson's disease | Adenosine | 0.2657 |  |
| map04750 | Inflammatory mediator regulation of TRP channels | Resiniferatoxin | 0.3207 |  |
| map04924 | Renin secretion | Adenosine | 0.3207 |  |
| map00360 | Phenylalanine metabolism | Phenylglyoxylic acid; Benzoic acid | 0.3348 |  |
| map04977 | Vitamin digestion and absorption | Riboflavin-5-phosphate; Nicotinamide | 0.3348 |  |
| map04024 | cAMP signaling pathway | Adenosine | 0.3718 |  |
| map04923 | Regulation of lipolysis in adipocytes | Adenosine | 0.3718 |  |
| map00130 | Ubiquinone and other terpenoid-quinone biosynthesis | Homogentisic Acid | 0.4192 |  |
| map00430 | Taurine and hypotaurine metabolism | alpha-Ketoglutaric acid | 0.4192 |  |
| map00220 | Arginine biosynthesis | alpha-Ketoglutaric acid | 0.4631 |  |
| map04742 | Taste transduction | Saccharin | 0.4631 |  |
| map05215 | Prostate cancer | Cortisol | 0.4631 |  |
| map05200 | Pathways in cancer | Cortisol | 0.5039 |  |
| map00410 | beta-Alanine metabolism | Carnosine | 0.5417 |  |
| map00240 | Pyrimidine metabolism | Deoxycytidine; 2'-Deoxycytidine | 0.6322 |  |
| map04080 | Neuroactive ligand-receptor interaction | Cortisol; Adenosine | 0.6322 |  |
| map00140 | Steroid hormone biosynthesis | Cortisol; Cortodoxone | 1 |  |
